# Analysis of university students’ perception of mental health

**DOI:** 10.1186/s12889-025-25213-7

**Published:** 2025-11-10

**Authors:** Iveta Vrabková, Ivana Vaňková

**Affiliations:** Department of Management, Faculty of Economics, VSB – Technical University of Ostrava, 17. listopadu 2172/15, Ostrava , 708 00 Czech Republic

**Keywords:** Mental health, University students, Factor analysis, Gender, Urban environment, Prevention

## Abstract

The mental health of university students has become an increasingly important public health issue, especially after the COVID-19 pandemic. Despite the high prevalence of mental illness, little is known about how students themselves perceive their mental health and which contextual and institutional factors influence these perceptions. This study examined mental health perceptions among university students in the Czech Republic, focusing on their views of the urban environment and preferred forms of support. A cross-sectional online survey was conducted among 767 students from four public universities. The 15-item questionnaire covered internal experiences, social background, and institutional conditions. Data were analysed using principal component analysis (PCA) and Welch’s t-test to assess gender differences. Three components initially emerged: (1) subjective mental well-being, (2) contextual and interpersonal support, and (3) institutional determinants. However, due to low internal consistency (Cronbach’s α = 0.285), Component 2 was excluded. The final interpretation therefore focused on two dimensions: subjective self-perception of mental health and social/institutional determinants. Gender differences were statistically significant for the latter, with women scoring higher. Students most often reported workload, social media, and family relations as stressors, while they valued cultural and educational opportunities of cities. The most frequently preferred forms of support included strengthening psychological services at universities, preventive care, and easier access to professional help. The findings indicate that students’ mental health perceptions are shaped by individual, social, and institutional dimensions. Public health strategies should therefore extend beyond clinical treatment, strengthen preventive and community-based approaches, address gender differences, and enhance the role of universities as key sites of early intervention.

**Supplementary Information:**

The online version contains supplementary material available at 10.1186/s12889-025-25213-7.

## Background

The mental health of young adults has become a major public health priority both globally and in the Czech Republic. This priority is reflected in the National Mental Health Action Plan 2020–2030, which emphasises prevention, early intervention, and accessible care for vulnerable groups, including university students [1]. The university period is characterised by psychosocial pressures—transition to independence, academic demands, and uncertainty about the future—that create conditions for mental health difficulties.

In this study, the terms mental health and mental well-being are both relevant. While mental health refers to psychological functioning and coping ability, mental well-being reflects subjective balance and satisfaction. The two overlap considerably; for clarity, the term mental health is used consistently.

Global reports such as the World Mental Health Report [2] highlight a growing crisis among young people, linked to digital exposure, socioeconomic uncertainty, and limited service access. The COVID-19 pandemic accelerated these risks, increasing anxiety and depression prevalence by more than 25%, particularly among young adults and women.

Social determinants strongly influence young people’s mental health. Tew et al. (2012) [3] documented the impact of unstable jobs and socioeconomic pressures, while Stewart & Vigod (2016) [4] underlined gender disparities, with women facing a heavier burden. Urban environments also matter—Peen et al. (2010) [5] found that density, anonymity, and noise increase mental health risks. System-level reforms in psychiatric care further illustrate how access to appropriate services shapes mental health outcomes, as shown by Vrabková et al. (2023) [6], who identified gaps in the dynamics and performance of deinstitutionalization processes in the Czech and Slovak Republics.

Despite high prevalence, help-seeking remains low due to stigma and limited mental health literacy [7], and mental disorders represent a major cause of disability comparable to chronic somatic diseases [8]. While many studies focus on determinants such as family or socioeconomic status [9], fewer explore students’ subjective perceptions in relation to urban environments and support systems, particularly with respect to gender. This study aims to address this gap by examining Czech university students’ perceptions of mental health in relation to the urban environment and preferred support systems, with a focus on gender differences.

## Methodology

The objective was to identify latent variables shaping university students’ mental health perceptions and to explore how they view urban environments and preferred interventions. Two sub-objectives (SO) and research questions (RQ) were defined:

SO1 Identify latent components of students’ mental health perception using factor analysis.

SO2 Assess perceptions of urban environment and preferred interventions.

RQ1 What latent components shape students’ mental health perceptions?

RQ2 How do these components reflect awareness and education about mental health?

RQ3 How are urban environment features perceived in relation to mental health?

RQ4 Do gender differences shape perceptions?

RQ5 Which interventions are considered most effective?

### Questionnaire and data collection

The questionnaire was developed based on previous research focusing on the prevalence of mental disorders in the population aged 0–25 years in the Czech Republic [10], as well as consultations with experts. It comprised 15 items addressing internal experiences, social background, and institutional conditions. The questionnaire included ordinal (O), nominal (N), and binary (B) variables (see Table 1). It was pilot-tested on a sample of 27 students to ensure clarity and relevance, and was subsequently administered online in February 2025 to students from four major Czech universities in the field of social sciences. According to Czech Statistical Office data, a total of 5,700 students in economics were enrolled in this field at these universities in 2024 [11]. Information about the study was disseminated to students at all institutions via the internal university system Edison and through the official social media channels of the universities, where a link to the Google Forms questionnaire was also provided. In total, 767 valid responses were obtained.

Ethics approval was obtained from the Ethics Committee of VSB-Technical University of Ostrava, reference no. VSB/25/044396. The study was conducted in accordance with the Declaration of Helsinki. All participants provided informed consent prior to participation. Incomplete responses were excluded using listwise deletion, and only cases with valid answers to all relevant items were included in the statistical analysis.

The questions are introduced in Table 1 below.

**Table 1:** Questions for the quantitative research

	Q	Type of variable
Q1	How would you rate your current mental state?	O
Q2	If you sought professional help for your mental health (e.g., psychologist, psychiatrist), how would you rate the quality of these services?	O
Q3	Do you think mental health care is easily accessible in your city/region?	O
Q4	Which factors do you think affect the mental health of young people aged 18–25 the most? (select the 3 most important)	B
Q5	Do you live in an urban or rural environment?	N
Q6	What benefits of living in the city do you think have a positive impact on mental health? (select the 3 most important)	B
Q7	What disadvantages of living in the city do you think deteriorate mental health? (select the 3 most important)	B
Q8	If you could choose, where would you prefer to live in terms of mental health?	N
Q9	How important is support from family and friends to you in dealing with mental health problems?	O
Q10	Do you think the age at which people enter parenthood can affect the mental health of their children?	O
Q11	How much of an impact do you think crime in your region has on your mental health?	O
Q12	Do you think the age of the first-time mother can affect her mental health?	O
Q13	How would you rate the level of general awareness and education about mental health in secondary schools and colleges?	O
Q14	Which of the following do you think would be most helpful in improving mental health care for young people in your region? (select up to 2 options)	B
Q15	How often do you feel you have to look after your mental health on your own, without the help of professionals or family?	O

The research (including questionnaire design) was grounded in the assumption of a two-factor model, where latent dimensions of mental health perception include both aspects of well-being (e.g., subjective state, support) and threat factors (e.g., loneliness, lack of access to care). The two-factor model [12, 13] conceptualises mental health as two independent yet co-existing domains. The first domain includes psychopathological symptoms and difficulties, while the second comprises psychological functioning and well-being. In line with Bernanke et al. (2017) [14], who applied latent class analysis to identify groups of students with different mental health risks, this study also seeks to uncover hidden patterns in students’ perceptions of their well-being. The methodological approach was further inspired by Zhou et al. (2020) [13], who used latent profile analysis to distinguish different types of adolescent mental health, supporting the value of person-centred approaches for identifying latent patterns in subjective perception.

### Analytical strategy

Data were analysed using principal component analysis (PCA). Suitability of the data was confirmed by a Kaiser–Meyer–Olkin measure (KMO = 0.523) and Bartlett’s test of sphericity (χ²(28) = 289.0; *p* < 0.001). Although PCA is primarily a data reduction technique, in this study it was employed as an exploratory tool to reveal interpretable structures underlying students’ perceptions of mental health.

PCA transforms the original variables X₁, X₂, …, Xₚ into new, orthogonal components Zₖ that maximise variance in the data (1):

1 $$Z_{k}\;=\;a_{k1} X_{1}\;+\;a_{k2} X_{2}\;+\;\ldots\;+\;a_{kp} X_{p}\;=\;{a_{k}}^{t} X$$

where *aₖ* is the eigenvector of correlation matrix Σ. The components are independent of each other and maintain order according to the explained variance.

The number of components retained was determined by the Kaiser criterion (eigenvalue > 1) and the scree plot. To increase interpretability, varimax rotation was applied, which maximises high loadings and minimises low loadings, thereby clarifying the structure of latent dimensions. Reliability was assessed using Cronbach’s alpha.

Although PCA is not equivalent to a latent variable model, its application in this study enabled identification of patterns of perception. The extracted components should therefore be interpreted as exploratory perception patterns rather than validated latent constructs.

### Factor scores and statistical testing

Factor scores were calculated as weighted averages of items, with weights corresponding to the factor loadings. To assess gender differences, Welch’s t-test for independent samples was employed, as it does not assume equality of variances. Effect sizes were calculated using Cohen’s d to estimate the magnitude of observed differences.

All statistical analyses were conducted using IBM SPSS Statistics (Version 29).

### Results of the questionnaire survey

The online questionnaire was completed by 767 respondents aged 18–26 years. The most frequent ages were 20 (20.7%), 22 (19.8%), and 21 years (18.4%). Women accounted for 60.5% of the sample and men for 39.5%. Regarding place of residence, almost half of respondents reported living in urban areas (46.2%), while 29.9% lived in mixed urban–rural environments and 24.0% in rural areas.

### Latent variables: principal component analysis

**Fig. 1:**
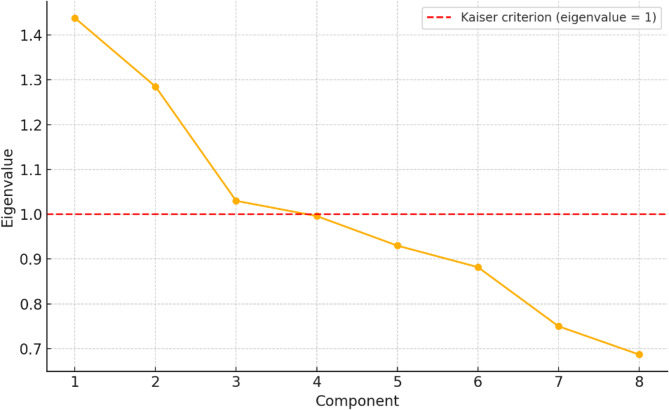
Scree plot PCA

Principal component analysis (PCA) initially identified three components with eigenvalues greater than 1, explaining 46.9% of the total variance (Table 2; Fig. 1). However, the second component (“Contextual and interpersonal support”) contained only two items (Q9 and Q11) and showed very low internal consistency (Cronbach’s α = 0.285). As this value fell well below the accepted threshold, only two components were retained for interpretation (PC1 and PC3).

**Table 2:** Total variance explained

Component	Initial Eigenvalues			Extraction Sums of Squared Loadings			Rotation Sums of Squared Loadings		
	Total	% of Variance	Cumulative %	Total	% of Variance	Cumulative %	Total	% of Variance	Cumulative %
1	1.438	17.975	17.975	1.438	17.975	17.975	1.428	17.855	17.855
2	1.285	16.069	34.044	1.285	16.069	34.044	1.181	14.761	32.616
3	1.030	12.881	46.925	1.030	12.881	46.925	1.145	14.309	46.925
4	0.996	12.453	59.378						
5	0.930	11.627	71.005						
6	0.882	11.030	82.035						
7	0.750	9.380	91.415						
8	0.687	8.585	100.000						

Extraction Method: Principal Component Analysis, SPSS

Although the three-component solution explained 46.9% of the variance, only PC1 and PC3 were retained. Together, they accounted for 32.2% of the variance (PC1 = 17.9%, PC3 = 14.3%). The scree plot (Fig. 1) illustrates the eigenvalue distribution and confirms the importance of the first three components, with a clear drop after the third.

**Fig. 2:**
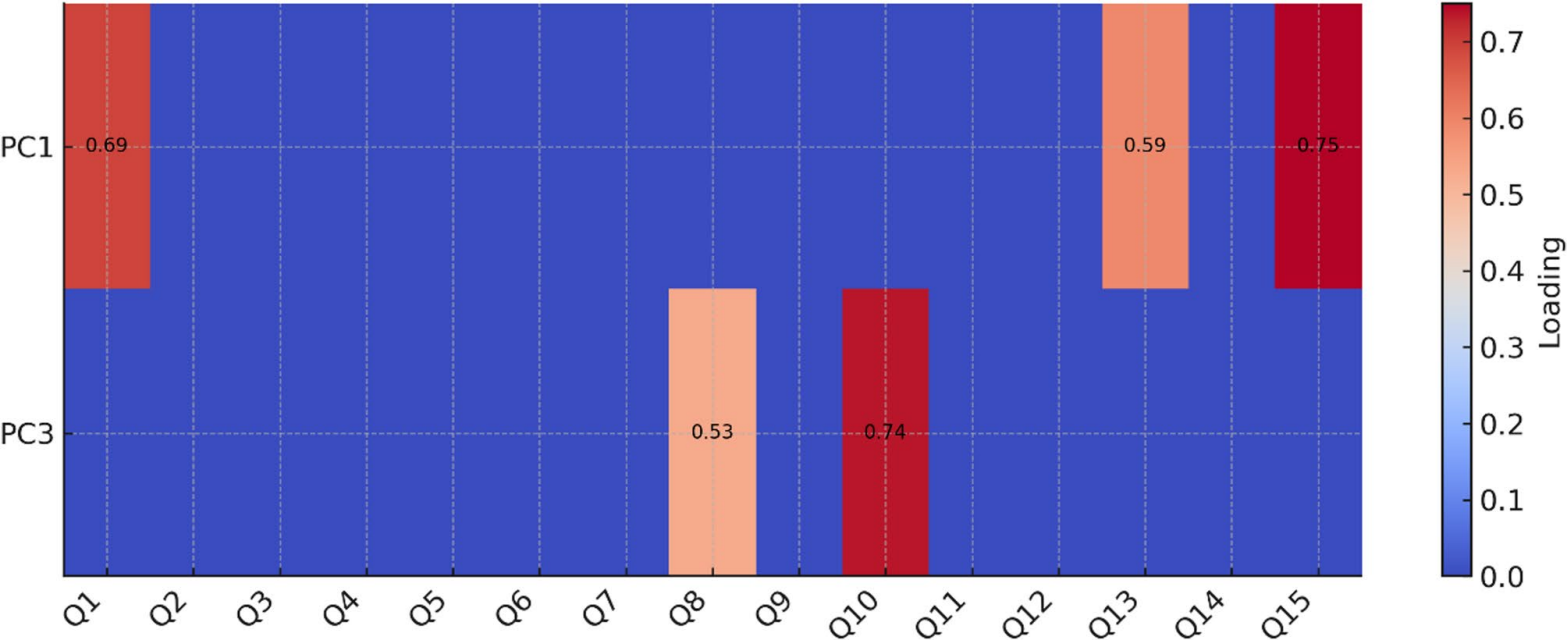
Factor loadings heat map

Varimax rotation redistributed the explained variance and highlighted the dominant loadings of the items. Figure 2 presents a heat map of factor loadings, showing contributions of each questionnaire item (Q1–Q15) to PC1 and PC3.

Component 1: Subjective perspective on mental health.

This factor included items relating to individual experiences and self-assessment: loneliness in dealing with mental health (loading = 0.750), evaluation of current mental state (0.693), and perception of mental health awareness in schools (0.590). It can be interpreted as students’ self-perception and awareness of their own mental health.

Component 3: Social and institutional determinants.

This factor grouped items on broader social and institutional aspects: perceived impact of parents’ age on children’s mental health (0.736), preferred place of living in terms of mental health (0.528), and availability of mental health care (0.508). It can be understood as the institutional and community dimension of mental health.

### Gender differences

Factor scores were calculated as a weighted average of items using the factor loadings. Welch’s t-test for independent samples was applied. The results showed that for Component PC1, there were no statistically significant differences between men and women (t = − 1.31; *p* = 0.192; Cohen’s d = − 0.14). For Component PC3, statistically significant gender differences were observed (t = − 3.49; *p* < 0.001; Cohen’s d = 0.27), with women scoring higher on average (F = 1.65) compared to men (M = 1.49).

### Results of the perception of mental health influences and interventions

Which factors do you think affect the mental health of young people aged 18–25 the most?

**Fig. 3:**
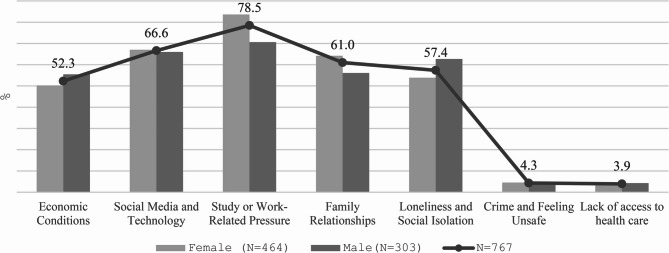
Factors affecting university students’ mental health

Figure 3 illustrates the distribution of respondents’ perceptions of external factors influencing mental health, broken down by gender (women *N* = 464, men *N* = 303; overall *N* = 767). Each respondent selected up to three of seven predefined factors perceived as having a negative impact on mental health.

The most common determinant was workload related to study or employment, reported by 78.5% of respondents, with women emphasising this factor more strongly. Social media and technology (66.6%) ranked second, and family relationships (61.0%) third, both more frequently mentioned by women. Loneliness and social isolation were relevant for 57.4% of respondents, again more often among women. About half of the respondents (52.3%) cited economic conditions as a stressor, with no significant gender differences.

By contrast, factors such as crime and feelings of insecurity (4.3%) and lack of access to healthcare (3.9%) were reported only marginally, suggesting lower subjective relevance in relation to mental well-being.

Overall, women more frequently identified stressors connected with the social environment, interpersonal relationships, and digital technologies.

**Fig. 4:**
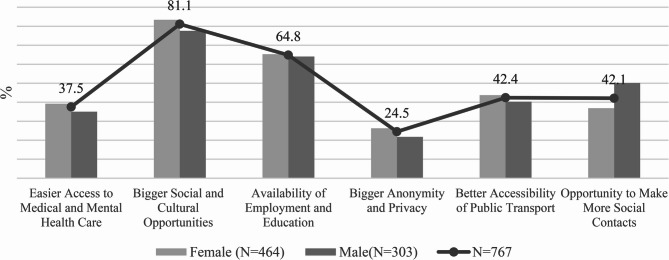
Positive factors City life on mental health

Figure 4 shows the frequency of positively perceived aspects of the urban environment. Respondents selected up to three of six predefined factors.

The most significant benefit was the broader social and cultural offer (81.1%), underscoring the role of cities as places of cultural stimulation and social engagement. Access to employment and education (64.8%) ranked second, reflecting the city’s socio-economic opportunities. Wider social contacts (42.1%) and better accessibility of public transport (42.4%) were also reported, with minor gender differences. Easier access to healthcare (37.5%) was more frequently mentioned by women, indicating higher sensitivity to institutional support.

Greater anonymity and privacy were mentioned by only 24.5% of respondents, suggesting low importance of individualisation in this context. Overall, urban environments were primarily valued for cultural, educational, and economic opportunities, while privacy, anonymity, and healthcare availability played a secondary role.

**Fig. 5:**
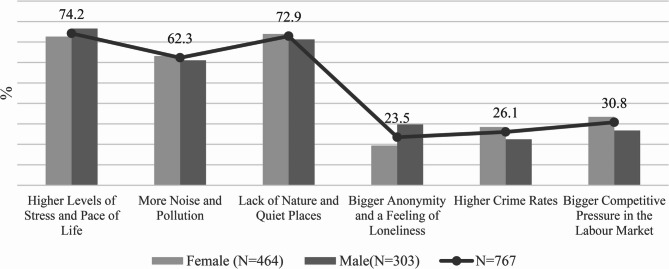
Negative impact of life in the City on mental health

Figure 5 displays perceptions of urban disadvantages. Respondents again selected up to three of six predefined factors.

The most frequent issue was increased stress and fast pace of life (74.2%), more strongly reported by women. Almost as many respondents (72.9%) emphasised the lack of natural environments and quiet areas, confirming the role of nature in psychological well-being. Noise and pollution (62.3%) ranked third, again highlighted by women.

Less frequently mentioned, but still notable, were competitive working environments (30.8%), higher crime rates (26.1%), and anonymity associated with loneliness (23.5%). These factors, although less common, may still significantly affect certain subgroups.

In summary, urban disadvantages were most often associated with stress, environmental deficits, and sensory overload, while crime and social isolation were less prominent.

**Fig. 6:**
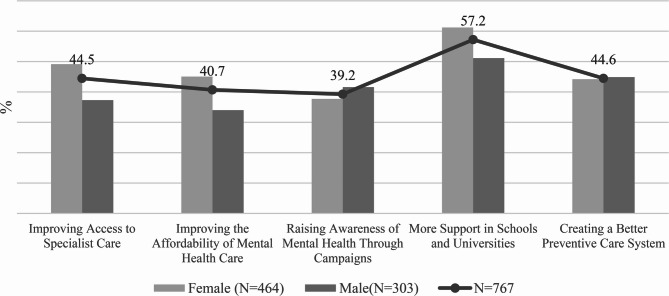
What can improve mental health of young people?

Figure 6 presents the respondents’ preferred areas of improvement in mental health care (five options available).

The most common priority was strengthening support in schools and universities (57.2%), highlighting educational institutions as key points for prevention and early intervention. This was followed by better preventive care systems (44.6%) and improved availability of specialist care (44.5%), both more frequently mentioned by women.

Other relevant areas included reducing financial barriers to care (40.7%) and awareness campaigns (39.2%).

Overall, respondents prioritized systemic changes with an emphasis on prevention, the university environment, and access to specialized care, with gender differences remaining relatively consistent across categories. Similar conclusions were reached by the authors in [15].

## Discussion

This study identified latent variables shaping university students’ perceptions of mental health, their views of the urban environment, and preferred interventions.

Three components initially emerged from the PCA: subjective perspective on mental well-being, contextual factors and interpersonal support, and institutional determinants. However, due to low internal consistency, the second component was excluded from interpretation. The results thus highlight two reliable dimensions: subjective perception of mental health and institutional/social determinants.

Urban environments were perceived ambivalently: cultural and educational opportunities were valued, but stress, noise, and lack of nature were major concerns. These findings support earlier evidence that cities may simultaneously act as sources of support and risk [5, 16].

Gender differences were observed. For Component 1 (subjective perspective on mental health), men scored slightly higher, but the difference was not statistically significant (t = − 1.31; *p* = 0.192). For Component 3 (institutional and community determinants), women scored significantly higher (t = − 3.49; *p* < 0.001), indicating greater sensitivity to institutional and community factors. This nuance aligns with earlier findings that women tend to report stronger responses to social stressors [4] and underscores the need for gender-sensitive interventions.

Students most often recommended strengthening psychological support at universities, prevention, and psychoeducation. These findings confirm the potential of educational institutions as key points for early detection and intervention.

From a practical perspective, interventions should extend beyond acute problems to building resilience and overall well-being. Expanding preventive programmes at universities is therefore recommended.

The study also has several limitations:

- voluntary participation led to higher female representation,
- cross-sectional design prevents causal inference,
- contextual and interpersonal support (Component 2) lacked reliability,
- the instrument was exploratory rather than psychometrically validated.

Future studies should expand the item set, combine quantitative and qualitative methods, and validate scales specifically for students’ perceptions of mental health.

In the Czech context, cultural specifics such as persistent stigma, regional disparities in service availability, and low mental health literacy remain barriers to care [1]. According to the Institute of Health Information and Statistics [17], over 50,000 young adults (18–26) were treated in outpatient psychiatric care in 2022, with women comprising more than 60%. The most frequent diagnoses were mood disorders and stress-related conditions. These data highlight the importance of preventive, accessible, and gender-sensitive approaches.

## Conclusion

The research identified two main latent variables shaping students’ perceptions of mental health: subjective perspective on well-being and social/institutional determinants. Perceptions of urban life were ambivalent—valuing opportunities while pointing to stress, lack of nature, and sensory overload.

Preferred interventions focused on preventive and university-based care, supplemented by awareness and specialist services. Gender patterns showed women more strongly emphasising institutional and community aspects, while men reported slightly higher—but not statistically significant—scores in subjective well-being.

These findings may inform targeted interventions and systemic changes in education and healthcare to better address the needs of young people in contemporary urban contexts.

## Supplementary Information

Supplementary Material 1.

Supplementary Material 2.

Supplementary Material 3.

Supplementary Material 4.

## Data Availability

All data generated or analyzed during this study are included in this article.
