# Supplementary material for: Analysis of university students’ perception of mental health
Source: BMC Public Health. 2025 Nov 10;25:3868. doi: 10.1186/s12889-025-25213-7 (PMC12599076; doi:10.1186/s12889-025-25213-7)
Supplement: Supplementary file 1 — Supplementary Material 1. [file 12889_2025_25213_MOESM1_ESM.docx]

**Analysis of University Students’ Perception of Mental Health**

**Iveta Vrabková^1^**(corresponding author), **Ivana Vaňková^2^**

^1^ [iveta.vrabkova@vsb.cz](mailto:iveta.vrabkova@vsb.cz), Department of Management, VSB – Technical University of Ostrava, Ostrava City, Czech Republic

^2^ [ivana.vankova@vsb.cz](mailto:ivana.vankova@vsb.cz), Department of Management, VSB – Technical University of Ostrava, Ostrava City, Czech Republic

**Abstract**

Mental health of university students has become an increasingly important public health issue, especially following the COVID-19 pandemic. Despite the high prevalence of mental illness, there still lacks a deeper understanding of how students themselves perceive their mental health and what contextual and institutional factors influence this perception. This research focused on the perception of mental health among university students in the Czech Republic and analysed their attitudes towards the urban environment as well as the preferred forms of support.

The cross-sectional questionnaire survey, conducted online, involved 767 students from five public universities. The fifteen questions covered the areas of internal experiences, social background, and institutional conditions. Data were analysed using the principal component analysis (PCA) and Welch’s t-test to compare gender differences.

Analysis revealed three latent components shaping the perception of mental health: (1) subjective mental well-being (e.g., loneliness, self-assessment of the mental state); (2) contextual and interpersonal factors (e.g., feeling of security, family support); and (3) institutional determinants (e.g., availability of services, family background). Gender differences were statistically significant in the second and third components – women showed higher sensitivity to institutional and environmental factors. Social networks, study demands, and family relationships were perceived as the main stressors by the students, while cultural and educational possibilities of the cities were valued positively by them. Availability of services at the universities, prevention, and easier access to care were considered by them as the most suitable forms of support.

The findings show that students’ perception of mental health is multilayered – influenced by individual, social, as well as institutional aspects. Efficient public health strategies should therefore not be limited to clinical intervention, but they should corroborate the university environment, reflect gender differences, and improve the systemic availability of support.

**Keywords:** mental health, university students, factor analysis, gender, urban environment, prevention

**Background**

Mental health of young adults has become a major public health policy priority in the last decade, not only in the Czech Republic, but also globally. In the Czech Republic, this priority is reflected in the National Mental Health Action Plan 2020–2030, which accentuates the need for prevention, early intervention, and accessible care for vulnerable groups, among whom university students feature prominently (MoH CR, 2025) [1]. The period of university studies is characterised by increased psychosocial pressure – transition to independence, academic demands, and uncertainty about the future – which creates the conditions for mental health problems. In this study, two terms appear—“mental health” and “mental well-being”—which are partially overlapping in the academic literature. Mental health typically refers to psychological functioning and the ability to cope with stress, while mental well-being reflects a subjective sense of balance and satisfaction. These dimensions are closely related. **For the purposes of this study, we will consistently use the term mental health throughout the text.**

Global reports, including the World Mental Health Report (WHO, 2022) [2], point to the growing crisis in the mental health of young people. The main risk factors include the increased digital burden, socioeconomic instability, and limited access to quality mental health services, a fact reflected in more than 25% increase in the prevalence of anxiety and depressive disorders after the COVID-19 pandemic – primarily among young adults and women.

There is long-standing evidence that young people’s mental health is significantly influenced by social determinants (Anthony, W. A.) [3]. Tew et al. (2012) [4] identified the influence of socioeconomic uncertainty, temporary jobs, and loss of identity on psychological well-being. Stewart and Vigod (2016) [5] pointed out gender inequality where women face a higher burden due to the combination of care, work, and study. The environment in which young people live also plays an important role – according to Peen et al. (2010) [6], urban environment with a high density of inhabitants, anonymity, and noise is an independent risk factor for mental disorders.

According to WHO (2017) [7], anxiety, depression, and sleep disorders are among the most common diagnoses in this age group, and they are the main causes of disability, comparable to chronic somatic diseases (Vigo et al., 2016) [8]. Despite the high prevalence of mental disorders, the rate of seeking professional help remains low, especially due to stigmatisation and low mental health literacy.

Although there are already studies examining the individual determinants of mental health (Gatdula et al., 2022) [9], a deeper understanding of how university students subjectively perceive their mental health in the context of the urban environment and what forms of support they find most effective is still lacking. At the same time, it has not been sufficiently mapped whether and how these perceptions differ by gender.

This article is divided into four parts. Methodology describes the research design, questionnaire design, and analytical approach including factor analysis. The part Results presents the main outputs including latent components and gender differences identified. Discussion interprets the results in the light of contemporary literature and proposes implications for the creation of mental health policies. The article concludes with a summary of key findings and recommendations for further research and intervention practice.

**Methodology**

The methodology was selected in order to achieve the research objective – to identify latent variables behind university students’ perception of mental health and to inquire into their perception of the urban environment and preferred interventions to support mental well-being. This objective is divided into two sub-objectives (SO), supplemented by research questions (RQ).

SO1: Identify the main latent components of university students’ perception of mental health using the factor analysis.

SO2: Evaluate how students reflect on the positive and negative dimensions of the urban environment in relation to mental health, and what types of intervention they perceive as the most beneficial to its promotion.

RQ1: What major components shape students’ perception of mental health based on their attitudes and experiences?

RQ2: How the main components identified (latent variables) reflect the general awareness and education about mental health?

RQ3: How students perceive positive and negative aspects of the urban environment in relation to their mental health?

RQ4: Does perception of factors affecting mental health differ by gender?

RQ5: Which types of interventions do students find most effective in supporting their mental health?

The research (including the questionnaire design) is based on the assumption of a two-factor model, meaning that the latent dimensions of the perception of mental health contain both the aspects of well-being (e.g., subjective state, support) and the threat factors (such as loneliness, unavailability of care). The two-factor model (Keyes, 2005, Zhou et al., 2020) [10, 11] construes mental health as two independent, but co-existing areas. The first area includes psychopathological symptoms and difficulties, the second includes psychological functioning and well-being. Similarly to Bernanke et al. (2017) [12], who used latent analysis of classes to identify groups of students with different mental health risks, this research also focuses on identifying hidden patterns in how students perceive their mental well-being. The methodological approach was also inspired by the study of Zhou et al. (2020) [11] that used latent profile analysis to identify different types of mental health among adolescents, thus supporting the suitability of person-centred approaches for revealing latent patterns in subjective perception.

The research builds on the findings of previous secondary research, which shows that the prevalence of mental illnesses in the young population increases in time and is also influenced by socioeconomic factors like the urban and rural environment, availability of care, criminality, and the age of first-time mothers [13].

Formulation of the questions was consulted with experts in psychological and psychiatric care and then tested in the pilot preliminary research in a group of 27 students (15 women and 12 men). The final set of 15 questions was distributed online to students at four of the largest public universities in the Czech Republic (VSB – Technical University of Ostrava, Masaryk University, Charles University, and Palacký University), in order to ensure theoretical geographical coverage of the country. The questionnaire was circulated via social media during February 2025.

The Ethics Committee of VSB – Technical University of Ostrava reviewed the questionnaire and the accompanying cover letter, which explained the scientific objectives of the survey to the participants. The review ensured that the research design met ethical standards for research involving human participants. Ethics approval was granted under the reference number VSB/25/044396. The study was conducted in accordance with the Declaration of Helsinki.

The questionnaire included ordinal (O), nominal (N), and binary (B) types of variables (indicated in the table). Incomplete responses were excluded from the analysis using listwise deletion. Only cases with valid answers to all relevant items were included in the principal component analysis (PCA) and statistical testing. Despite including binary and nominal items, all variables were treated as numeric inputs in the PCA. While this approach is common in exploratory social research, it may violate statistical assumptions. Future studies may employ methods that account for variable type more accurately.

The questions are introduced in Table 1 below.

Table 1 Questions for the Quantitative Research

|  | Q | Type of variable | |  |
| --- | --- | --- | --- | --- |
| Q1 | How would you rate your current mental state? | | O | |
| Q2 | If you sought professional help for your mental health (e.g., psychologist, psychiatrist), how would you rate the quality of these services? | | O | |
| Q3 | Do you think mental health care is easily accessible in your city/region? | | O | |
| Q4 | Which factors do you think affect the mental health of young people aged 18–25 the most? (select the 3 most important) | | B | |
| Q5 | Do you live in an urban or rural environment? | | N | |
| Q6 | What benefits of living in the city do you think have a positive impact on mental health? (select the 3 most important) | | B | |
| Q7 | What disadvantages of living in the city do you think deteriorate mental health? (select the 3 most important) | | B | |
| Q8 | If you could choose, where would you prefer to live in terms of mental health? | | N | |
| Q9 | How important is support from family and friends to you in dealing with mental health problems? | | O | |
| Q10 | Do you think the age at which people enter parenthood can affect the mental health of their children? | | O | |
| Q11 | How much of an impact do you think crime in your region has on your mental health? | | O | |
| Q12 | Do you think the age of the first-time mother can affect her mental health? | | O | |
| Q13 | How would you rate the level of general awareness and education about mental health in secondary schools and colleges? | | O | |
| Q14 | Which of the following do you think would be most helpful in improving mental health care for young people in your region? (select up to 2 options) | | B | |
| Q15 | How often do you feel you have to look after your mental health on your own, without the help of professionals or family? | | O | |

Identification of latent dimensions in the perception of mental health was performed using the principal component analysis (PCA), designed to reduce the data set dimension and reveal hidden structures in the data. PCA transforms the original variables *X₁, X₂, ..., Xₚ* into new, orthogonal components *Zₖ,* which maximise variance on data (1):

*Zₖ = aₖ₁X₁ + aₖ₂X₂ + ... + aₖₚXₚ = aₖᵗX* (1)

where *aₖ* is the eigenvector of correlation matrix Σ. The components are independent of each other and maintain order according to the explained variance.

Although PCA is primarily a data reduction technique rather than a latent variable model, it was used in this study to identify interpretable components underlying students’ perceptions of mental health. Therefore, the component loadings are interpreted as coefficients of observed variables rather than as reflections of latent constructs.

To verify the suitability of the data for PCA, the Kaiser-Meyer-Olkin (KMO) measure of sampling adequacy was applied (KMO = 0.523), along with Bartlett’s test of sphericity (χ²(28) = 289.0; p < 0.001), both of which confirmed that the data were adequate for factor analysis.

To increase factor interpretability, the varimax rotation was used, which maximises high loadings and minimises low loadings. The number of components was determined by Kaiser criterion (eigenvalue higher than 1) and scree plot. Comparative analysis of factor scores was used to analyse differences in the perception of mental health among students. The factor scores for the individual components were calculated as a weighted average of items included in the given latent variable, while the weights corresponded to the sizes of factor loadings. To test gender differences, Welch’s t-test for independent samples was used, which is suitable when variance equality cannot be assumed. In addition to statistical significance testing, effect sizes were computed using Cohen’s d.

Calculations were performed in IBM SPSS Statistics (Version 29).

**Results of the Questionnaire Survey**

The number of respondents of the on-line questionnaire aged 18–26 years was 767 (N = 767). The most numerous were respondents aged 20 (20.7%), 22 (19.8%), and 21 years (18.4%). The sample included 60.5% of women and 39.5% of men. The respondent structure according to the place of residence shows that almost a half of the respondents come from the urban environment (46.2%), a third live in areas with combined features of urban and rural environments (29.9%), and a quarter of respondents indicated that they lived in rural areas (24.0%).

**Latent Variables: Principal Component Analysis**

The principal component analysis (PCA) identified three components with latent values (eigenvalues) higher than 1, which corresponds to the classic Kaiser criterion (see Table 2 and Figure 1). These three components combined explain 46.93% of the total variance in data. The first component has the eigenvalue of 1.438 and explains 17.98% of the total variance. The second component with the value of 1.285 contributes 16.07% of variance, thus the cumulative variability explained reaches 34.04%. The third component has the eigenvalue of 1.030, adding further 12.88%, which leads to the cumulative share of 46.93% of variance explained.

Table 2 Total Variance Explained

| Component | Initial Eigenvalues | |  | Extraction Sums of Squared Loadings | | | Rotation Sums of Squared Loadings | | |
| --- | --- | --- | --- | --- | --- | --- | --- | --- | --- |
|  | Total | % of Variance | Cumulative % | Total | % of Variance | Cumulative % | Total | % of Variance | Cumulative % |
| 1 | 1.438 | 17.975 | 17.975 | 1.438 | 17.975 | 17.975 | 1.428 | 17.855 | 17.855 |
| 2 | 1.285 | 16.069 | 34.044 | 1.285 | 16.069 | 34.044 | 1.181 | 14.761 | 32.616 |
| 3 | 1.030 | 12.881 | 46.925 | 1.030 | 12.881 | 46.925 | 1.145 | 14.309 | 46.925 |
| 4 | 0.996 | 12.453 | 59.378 |  |  |  |  |  |  |
| 5 | 0.930 | 11.627 | 71.005 |  |  |  |  |  |  |
| 6 | 0.882 | 11.030 | 82.035 |  |  |  |  |  |  |
| 7 | 0.750 | 9.380 | 91.415 |  |  |  |  |  |  |
| 8 | 0.687 | 8.585 | 100.000 |  |  |  |  |  |  |

Extraction Method: Principal Component Analysis, SPSS.

The scree plot in Figure 1 visualises the distribution of eigenvalues across the eight components. The number of retained components was based on the Kaiser criterion and the scree plot. Although this is a standard approach, we recognize that more robust methods such as parallel analysis or exploratory graph analysis could offer improved precision.

The decline following the third component, clearly shows that the first three factors are of greatest importance. The red line shows the limit of the Kaiser criterion (eigenvalue = 1), separating the components with higher values.

Figure 1 Scree Plot PCA


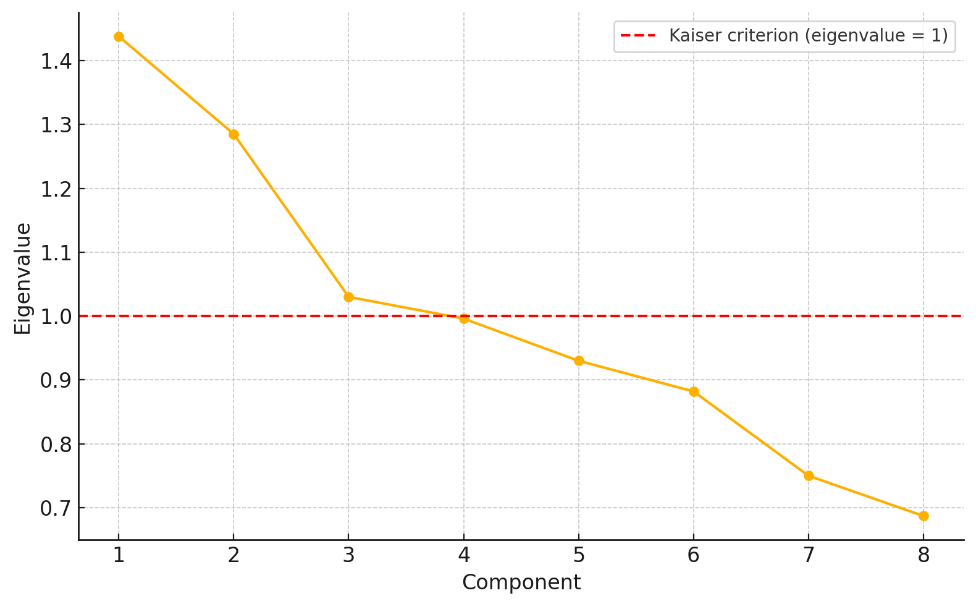


The varimax rotation (Table 2) caused a slight redistribution of the variance explained among these three components, but the cumulative total remained unchanged. The first component continues to have the largest share (17.86%), followed by the second (14.76%) and third (14.31%) components. The rotation ensures a more precise definition of the factor structure, as it highlights the dominant loadings of the variables to the individual components (latent variables). The loadings are illustrated by the factor loadings heat map, which shows how each manifest variable (Q1–Q15) contributes to the individual components, see Figure 2. Red shades indicate stronger positive loadings, grey shades weaker or no loadings.

Figure 2 Factor Loadings Heat Map


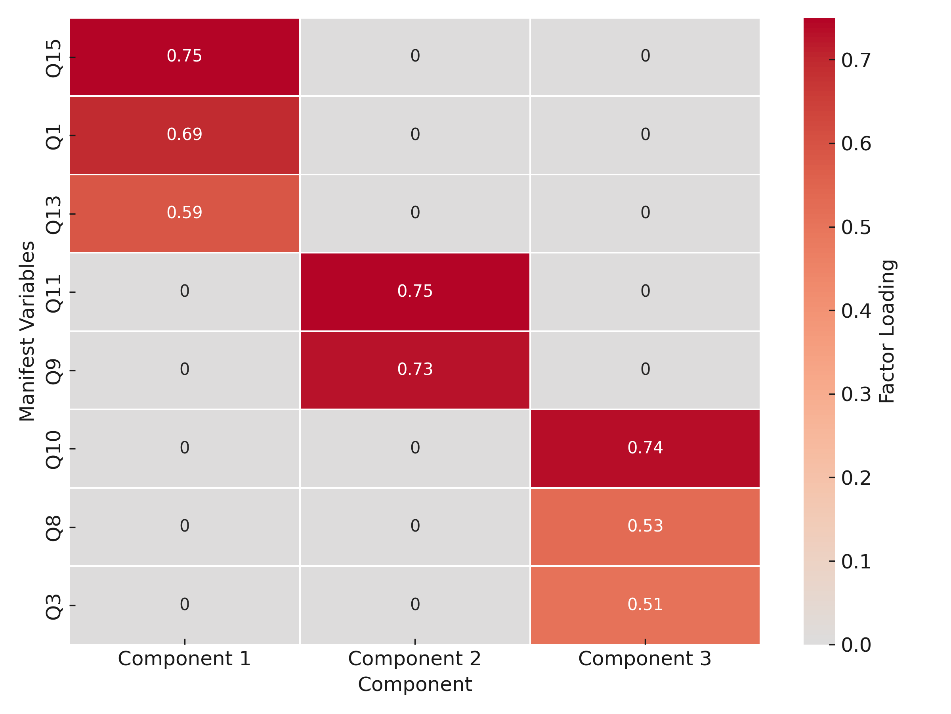


Component 1: Subjective perspective on mental well-being. This component comprises items related to personal experience of mental health – especially the feeling of loneliness in caring for mental health (0.750), evaluation of one’s own mental state (0.693), and perception of mental health awareness in the school environment (0.590). This dimension may be interpreted as self-perception and awareness of one’s own mental health.

Component 2: Contextual factors and interpersonal support. The second component captures the impact of crime in the area (0.750) and the importance of support from family and friends (0.730). It is a dimension that reflects the external context and availability of social support in coping with mental health problems. However, Component 2 (contextual and interpersonal factors) includes only two items (Q9 and Q11), and its internal consistency is low (Cronbach’s α = 0.285). This indicates limited reliability of this dimension, and its interpretation should therefore be approached with caution. Future studies should consider including additional items to improve factor stability.

Component 3: Social and institutional determinants of mental health. The third component includes items focusing on institutional approaches and broader social context: influence of parent’s age on the mental health of children (0.736), preferences for a place to live in terms of mental health (0.528), and availability of mental health care (0.508). This dimension can be perceived as institutional and community context of mental health.

These three components together explain approximately 47% of the total variance (see Table 2 Total Variance Explained). The results indicate that attitudes towards mental health are not uniform, but rather structured in different, yet interconnected dimensions.

Factor scores were calculated as a weighted average of items using the factor loadings. This was followed by the Welch’s t-test for independent samples that takes into account possible unequal variances.

In terms of gender differences, the results showed that for Component PC1, there were no statistically significant differences between men and women (t = –1.31; p = 0.192). In Component PC2, which includes the perceived impact of crime in the area and the importance of support from family and friends, statistically significant gender differences were found (t = –5.57; p < 0.001). Women reported higher scores (F = 3.25) than men (M = 2.92), indicating greater sensitivity to these contextual and social influences. Statistically significant differences were also observed for Component PC3, which reflects institutional and community determinants of mental health (t = –3.49; p < 0.001); again, women scored higher on average (F = 1.65) compared to men (M = 1.49), suggesting that they place greater importance on the availability of care and the surrounding environment.

In addition to statistical significance, effect sizes were also calculated using Cohen’s d. The results showed a very small effect for Component 1 (d = –0.14), a moderate effect for Component 2 (d = 0.43), and a small effect for Component 3 (d = 0.27). However, it should be noted that Component 2 includes only two items and has low internal consistency (Cronbach’s α = 0.285), which limits the interpretability of this dimension. Therefore, the results related to this component should be interpreted with caution, and future studies are advised to include additional items to strengthen its validity.

**Results of the Perception of Mental Health Influences and Interventions**

**Which factors do you think affect the mental health of young people aged 18-25 the most?**

Chart in Figure 3 shows the distribution of perception of external factors that affect mental health, broken down by gender (women N = 464, men N = 303; overall data set N = 767). Respondents had the opportunity to select 3 of the 7 predefined factors, which in their opinion negatively affect mental health.

The most common determinant was the workload associated with study or employment, identified as a significant factor by 78.5% of respondents. This area was more strongly emphasised among women. Social media and technology ranked seconds (66.6%) and family relationships ranked third (61.0%) – here too, the response rate was higher for women than for men.

Loneliness and social isolation were relevant for 57.4% of the respondents, again more common among women. Approximately a half of the respondents (52.3%), with no significant gender differences, indicated economic conditions as a potential stressor.

In contrast, factors like crime and feeling unsafe (4.3%) and lack of access to healthcare (3.9%) were entirely marginal, a fact that may reflect their lower subjective relevance in relation to mental well-being.

In summary, the perception of mental health determinants shows a gender-specific pattern where women more frequently identify stressors associated with the social environment, interpersonal relations, and digital technologies.

Figure 3 Factors Affecting University Students’ Mental Health

Chart in Figure 4 shows the frequency of occurrence of positively rated aspects of the urban environment in relation to their perceived impact on mental health. Respondents had the opportunity to select 3 of the 6 predefined factors, which in their opinion most strongly represent the supportive or beneficial dimensions of urban life.

The most significant factor was the greater social and cultural offer, identified by 81.1% of the respondents, a fact that underlines the importance of urban environment as a place of cultural stimulation and social engagement. The second most frequently cited area was access to employment and education (64.8%), which reflects the importance of the city as an area of socio-economic opportunity.

The possibility to establish wider social contacts (42.1%) and better accessibility of public transport (42.4%) were assessed as positive benefits to a comparable extent, with only minor difference between women and men. Easier access to medical and mental healthcare (37.5%) was slightly more often reported by women, which probably reflects their higher sensitivity to the institutional infrastructure of care.

In contrast, greater anonymity and privacy was identified as an advantage by mere 24.5% of the respondents, possibly indicating a lower importance of individualisation of the environment in relation to the perception of mental health.

Overall, urban environment is primarily perceived as a space of cultural, educational, and employment opportunities, while the aspects of privacy, anonymity, and access to healthcare play a minor role in the respondents’ perception.

Figure 4 Positive Factors City Life on Mental Health

Chart in Figure 5 shows the perception of negatively rated aspects of the urban environment that may adversely impact mental health. Respondents had the opportunity to select 3 of the 6 predefined factors, which in their opinion represent the main burdens of urban life.

The most frequently cited problem was increased stress levels and fast pace of life, identified by 74.2% of respondents, while women reflected this fact more strongly women. Almost the same percentage of respondents (72.9%) pointed out the lack of natural environment and quiet areas, which confirms the importance of the natural environment for perceived psychological well-being. The third most common area was noise and pollution (62.3%), again more accentuated by women.

In contrast, less prevalent, but not insignificant factors included higher crime rate (26.1%), more competitive working environment (30.8%), and anonymity associated with feelings of loneliness (23.5%). These aspects were less frequent in the answers, yet can significantly affect mental health in certain populations.

It is arguable that negative aspects of the urban environment are perceived by the respondents primarily in connection with stress, lack of natural environment, and sensory overload, while fear of crime and social isolation are less frequent.

Figure 5 Negative Impact of Life in the City on Mental Health

Chart in Figure 6 shows preferred areas of improvement in mental health care as perceived by the respondents, who had the opportunity to select from five possible interventions.

The most commonly stated priority was strengthening the support in schools and universities (57.2%), a fact that points out the perceived significance of prevention and intervention within educational institutions as key points for early detection of psychological problems. This factor was more strongly accentuated by women.

The second most frequently cited area was ensuring a better preventive care system (44.6%), indicating the respondents’ emphasis on early intervention and better systemic readiness of healthcare services. This was followed by improved availability of specialist care (44.5%), more often accentuated by women.

Key areas identified by relatively fewer respondents were the increased access to care in terms of financial demands (40.7%) and awareness campaigns (39.2%). Still, these areas remain relevant components of the comprehensive strategy to improve mental health care.

In general, respondents prefer systemic changes focusing on prevention, university environment, and availability of specialised care, while gender differences remain relatively consistent across all the categories.

Figure 6 What Can Improve Mental Health of Young People?

**Discussion**

This study was aimed at identifying latent variables that shape the perception of mental health of university students and understanding their attitudes towards urban environment and preferred interventions. The results yielded several important findings, which correspond to the research questions.

Three key latent components were identified using the factor analysis – subjective perspective on mental well-being, contextual factors and interpersonal support, and institutional determinants. These dimensions show that perception of mental health is not a matter of individual experience, but is rather shaped by a broader social and environmental context. Especially significant was the component reflecting subjective perception and independence in taking care of one’s own mental health, suggesting a certain level of individualisation of experience and coping with mental health problems.

Relationship between the urban environment and mental well-being was evaluated ambivalently by the respondents. Cultural and educational opportunities were perceived positively by them, while the most common negative factors cited were stress, noise, and lack of natural environment. These results confirm that city can be both a source of support and a risky environment, which is consistent with previous findings (e.g., Peen et al., 2010; Srivarathan et al., 2023) [6, 14].

Gender differences observed indicate that women perceive mental health more comprehensively in relation to the institutional and community factors, while men had higher scores in the area of subjective assessment of mental well-being. This supports earlier findings regarding higher sensitivity in women towards social stressors (Stewart & Vigod, 2016) [5] and indicates the need for gender-sensitive interventions.

Preferred forms of support most commonly indicated by the students included strengthening mental health within educational institutions, including psychological services at universities, prevention, and psychoeducation. These findings show the potential of university environments as key points of early detection of mental problems.

In terms of practical implications for mental health in general, it is important that interventions are not only focused on acute problems, but also on supporting students’ psychological resilience and overall well-being. It is therefore recommended to improve and expand the system of support at universities by preventive programmes.

However, this research has, logically, several limitations due to the questionnaire survey in the form of voluntary participation, as this is likely to create selection bias – especially in terms of higher representation of women. Data were collected at a single point in time, which does not allow for causal relationships to be observed. Last, but not least, the research did not involve the entire diversity of university population, such as students with migration background or from marginalised communities.

One of the limitations of the factor structure is the low internal consistency of the second component, which included only two items and yielded a Cronbach’s alpha of 0.285. Although this dimension is conceptually coherent, its statistical reliability is weak. Future research should consider expanding the item set to better capture the contextual and interpersonal factors affecting students’ mental health and thereby improve the interpretability and robustness of this component.

In the Czech context, the field of mental health is strongly influenced by several culturally specific factors. Above all, the persistent stigmatization of mental illness remains a significant barrier to seeking professional help, particularly among young adults. According to a government expert report [1], on quality-of-life indicators, health inequalities are further exacerbated by limited access to care, regional disparities in the availability of services, and the still low level of mental health literacy in the population.

According to data from the Institute of Health Information and Statistics [16], more than 50,000 clients aged 18–26 were recorded in outpatient psychiatric care in 2022, with women making up over 60% of this group. The most frequent diagnoses included mood disorders and neurotic, stress-related, and somatoform disorders, which together accounted for more than half of the cases in this age group. The Czech Republic 2030 strategy emphasizes the need to promote healthy lifestyles, prevention, and the reduction of health inequalities, including those related to mental health. These factors should be taken into account when interpreting the research findings—e.g., the lower willingness of young people to openly evaluate their psychological difficulties may lead to biased self-assessments of mental well-being.

At the same time, we highlight the methodological limitations of quantitative approaches – as shown by Laidlaw et al. (2016) [15], students can perceive the terms “mental health” and “mental well-being” differently and this may influence interpretation of the answers. An issue to be addressed is to what extent efficiency of preventive programmes can be influenced by this different understanding. It also seems valid to ask if and how the urban environment can be transformed to minimise stressors and corroborate mental health resilience of university students. Results of this study, supplemented by the findings of Srivarathan et al. (2023) [14], indicate that stability of social ties, availability of green zones, and community support seem to be key factors in mental comfort of young population living in cities.

Future research should take these limitations into consideration and combine quantitative approaches with qualitative methods that would allow deeper understanding of subjective implications associated with mental health.

**Conclusion**

The aim of the research was to identify latent variables that shape the perception of mental health of university students and understanding their attitudes towards urban environment and preferred interventions. Three components were found using the factor analysis: subjective perspective on mental well-being, contextual factors and interpersonal support, and social and institutional determinants of mental health. These dimensions show that perception of mental health is not reducible to individual symptoms, but is rather shaped by a social support, environment, and institutional framework.

The results also showed that students reflect the urban environment ambivalently – they value its cultural and economic opportunities, but at the same time point to stress, absence of nature, and sensory overload. Preferred interventions thus include available, prevention-oriented, and community-based care, with an emphasis on the university environment as a key place of support.

Gender differences ascertained indicate that women perceive external factors (such as crime rate, institutional availability of care) as being more significant, while men reach higher scores in subjective assessment of mental health. These differences should be taken into account when designing targeted interventions and preventive programmes.

The results of this study can serve as a basis for targeted interventions and systemic changes in educational and medical institutions to better meet the needs of young people in the changing social and urban context.

**References**

1. Ministry of Health Czech Republic. National Action Plan for Mental Health 2020-2030. <https://mzd.gov.cz/narodni-akcni-plan-pro-dusevni-zdravi-2020-2030/>. Accessed 10 Jan 2025.
2. World mental health report: Transforming mental health for all. (2022). <https://www.who.int/publications/i/item/9789240049338>. Accessed 5 Feb 2025.
3. Anthony, W.A. (1993). *Recovery from mental illness: the guiding vision of the mental health service system in the 1990s*. Psychosocial Rehabilitation Journal, 16(4), pp.11–23. <https://doi.org/10.1037/h0095655>.
4. Tew, J., Ramon, S., Slade, M., Bird, V., Melton, J. and Le Boutillier, C. (2012). *Social factors and recovery from mental health difficulties: a review of the evidence*. British Journal of Social Work, 42(3), pp.443–460. <https://doi.org/10.1093/bjsw/bcr076>.
5. Stewart, D.E. and Vigod, S.N. (2016). *Developing a gender-sensitive women’s mental health policy: lessons from Canada*. Health Care for Women International, 37(2), pp.134–148. <https://doi.org/10.1080/07399332.2015.1085312>.
6. Peen, J., Schoevers, R.A., Beekman, A.T.F. and Dekker, J. (2010). *The current status of urban-rural differences in psychiatric disorders*. Acta Psychiatrica Scandinavica, 121(2), pp.84–93. <https://doi.org/10.1111/j.1600-0447.2009.01438.x.5>.
7. World Health Organization (WHO). (2017). *Depression and Other Common Mental Disorders: Global Health Estimates*. Geneva: World Health Organization. <https://apps.who.int/iris/handle/10665/254610>. Accessed 10 Jan 2025.
8. Vigo, D., Thornicroft, G. and Atun, R. (2016). *Estimating the true global burden of mental illness*. The Lancet Psychiatry, 3(2), pp.171–178. <https://doi.org/10.1016/S2215-0366(15)00505-2>.
9. Gatdula, N., Costa, C. B., Rascón, M. S., Deckers, C. M., & Bird, M. (2022). College students’ perceptions of telemental health to address their mental health needs. Journal of American College Health, 72(2), 515–521. <https://doi.org/10.1080/07448481.2022.2047697>.
10. Keyes, C. L. M. (2005). Mental Illness and/or Mental Health? Investigating Axioms of the Complete State Model of Health. *Journal of Consulting and Clinical Psychology, 73*(3), 539–548. [https://doi.org/10.1037/0022-006X.73.3.539](https://psycnet.apa.org/doi/10.1037/0022-006X.73.3.539).
11. Zhou, J., Jiang, S., Zhu, X. *et al.* Profiles and Transitions of Dual-Factor Mental Health among Chinese Early Adolescents: The Predictive Roles of Perceived Psychological Need Satisfaction and Stress in School. *J Youth Adolescence* 49, 2090–2108 (2020). <https://doi.org/10.1007/s10964-020-01253-7>.
12. Bernanke, J., Stanley, B., Oquendo, M., & Posner, J. (2017). Toward fine-grained phenotyping of suicidal behavior: The role of suicidal subtypes. *Molecular Psychiatry, 22*(8), 1080–1081. DOI: <https://doi.org/10.1038/mp.2017.123>.
13. Vrabková, I., Vaňková, I. (2024). Socioeconomic Factors in the Prevalence of Mental Disorders in the Population Aged 0–25 Years: Regions in the Czech Republic. Preprint, <https://www.researchsquare.com/article/rs-5349124/v1>.
14. Srivarathan, A., Jørgensen, T. S. H., Lund, R., Nygaard, S. S., and Kristiansen, M. (2023). They are breaking us into pieces: A longitudinal multi-method study on urban regeneration and place-based social relations among social housing residents in Denmark. Health & Place, 79, 102965. <https://doi.org/10.1016/j.healthplace.2023.102965>.
15. Laidlaw, P., McLellan, A. and Ozakinci, G. (2016). Understanding undergraduate student perceptions of mental health, mental well-being and help-seeking behaviour. *Studies in Higher Education*, 41(12), pp.2156–2168. <https://doi.org/10.1080/03075079.2015.1026890>
16. Institute of Health Information and Statistics of the Czech Republic (2023). Psychiatrická ročenka 2022. https://www.uzis.cz/res/f/008442/psych2022.pdf

**Acknowledgements**

This paper was supported by project NO. SP2025/069 „Factors of Efficiency in the Distribution and Accessibility of Healthcare Services“, VSB – Technical University of Ostrava and it has been produced whith the financial support of the European Union under the REFRESH – Research Excellence For Region Sustainability and High-tech Industries project number CZ.10.03.01/00/22_003/0000048 via the Operational Programme Just Transition.

**Funding**

This study was supported by:

- SP2025/069 „Factors of Efficiency in the Distribution and Accessibility of Healthcare Services“, funded by VSB – Technical University of Ostrava.

REFRESH – Research Excellence For Region Sustainability and High-tech Industries project number CZ.10.03.01/00/22_003/0000048, funded by the European Union.

**Availability of data and materials**

All data generated or analyzed during this study are included in this article.

**Declarations**

Ethics approval and consent to participate: Ethics approval was obtained from the Ethics Committee of VSB-Technical University of Ostrava, reference no. VSB/25/044396. All participants provided informed consent prior to participation.

**Consent for publication.**

Not applicable.

**Competing interests**

The authors declare that they have no known competing financial interests or personal relationships that could have appeared to influence the work reported in this paper.

**Author details**

**Authors and Affiliations**

Department of Management, VSB – Technical University of Ostrava, 17. listopadu St. 2172/15, 708 00, Ostrava-Poruba, Czech Republic
