# Supplementary material for: Analysis of university students’ perception of mental health
Source: BMC Public Health. 2025 Nov 10;25:3868. doi: 10.1186/s12889-025-25213-7 (PMC12599076; doi:10.1186/s12889-025-25213-7)
Supplement: Supplementary file 2 — Supplementary Material 2. [file 12889_2025_25213_MOESM2_ESM.docx]

**References**

1. Ministry of Health Czech Republic. National Action Plan for Mental Health 2020-2030. <https://mzd.gov.cz/narodni-akcni-plan-pro-dusevni-zdravi-2020-2030/>. Accessed 10 Jan 2025.
2. World mental health report: Transforming mental health for all. 2022. <https://www.who.int/publications/i/item/9789240049338>. Accessed 5 Feb 2025.
3. Tew J, Ramon S, Slade M, Bird V, Melton J, Le Boutillier C. Social factors and recovery from mental health difficulties: a review of the evidence. British Journal of Social Work. 2012; 42(3): 443–460. <https://doi.org/10.1093/bjsw/bcr076>.
4. Stewart DE, Vigod SN. Developing a gender-sensitive women’s mental health policy: lessons from Canada. Health Care for Women International. 2016; 37(2): 134–148. <https://doi.org/10.1080/07399332.2015.1085312>.
5. Peen J, Schoevers RA, Beekman ATF, Dekker J. The current status of urban-rural differences in psychiatric disorders. Acta Psychiatrica Scandinavica. 2010; 121(2): 84–93. <https://doi.org/10.1111/j.1600-0447.2009.01438.x.5>.
6. Vrabková I, Vaňková I, Moškořová K. Gaps in the dynamics and performance of the psychiatric care deinstitutionalization. INQUIRY: The Journal of Health Care Organization, Provision, and Financing. 2023; 60, 1–10. <https://doi.org/10.1177/00469580231170727>
7. World Health Organization, WHO. Depression and Other Common Mental Disorders: Global Health Estimates. Geneva: World Health Organization. 2017. <https://apps.who.int/iris/handle/10665/254610>. Accessed 10 Jan 2025.
8. Vigo D, Thornicroft G, Atun R. *Estimating the true global burden of mental illness*. The Lancet Psychiatry. 2016; 3(2): 171–178. <https://doi.org/10.1016/S2215-0366(15)00505-2>.
9. Gatdula N, Costa CB, Rascón MS, Deckers CM, Bird M. College students’ perceptions of telemental health to address their mental health needs. Journal of American College Health. 2022; 72(2):515–521. <https://doi.org/10.1080/07448481.2022.2047697>.
10. Vrabková I, Vaňková I. Socioeconomic Factors in the Prevalence of Mental Disorders in the Population Aged 0–25 Years: Regions in the Czech Republic. Preprint, 2024. DOI: [10.21203/rs.3.rs-5349124/v1](https://urldefense.com/v3/__https:/doi.org/10.21203/rs.3.rs-5349124/v1__;!!NLFGqXoFfo8MMQ!px52Vzbbwghh3_HizcC1xphxsqMwJAa13axcBi_pRjt0hc-xpipr5YIUWV7XN2BJ1CJSmfMKeOkI0CrlwQixMihc5hGcOjI%24)
11. Czech Statistical Office. (2025). Studenti oborů ze skupiny Společenské vědy, žurnalistika a informační vědy ve veřejných a soukromých vysokých školách v Česku. Czech Statistical Office. <https://csu.gov.cz/open-data>
12. Keyes CLM. Mental Illness and/or Mental Health? Investigating Axioms of the Complete State Model of Health. Journal of Consulting and Clinical Psychology. 2005*; 73*(3): 539–548. [https://doi.org/10.1037/0022-006X.73.3.539](https://psycnet.apa.org/doi/10.1037/0022-006X.73.3.539).
13. Zhou J, Jiang S, Zhu X et al. Profiles and Transitions of Dual-Factor Mental Health among Chinese Early Adolescents: The Predictive Roles of Perceived Psychological Need Satisfaction and Stress in School. J Youth Adolescence. 2020; 49: 2090–2108. <https://doi.org/10.1007/s10964-020-01253-7>.
14. Bernanke J, Stanley B, Oquendo M, Posner J. Toward fine-grained phenotyping of suicidal behavior: The role of suicidal subtypes. Molecular Psychiatry. 2017; *22*(8): 1080–1081. <https://doi.org/10.1038/mp.2017.123>.
15. Laidlaw P, McLellan A, Ozakinci G. Understanding undergraduate student perceptions of mental health, mental well-being and help-seeking behaviour. Studies in Higher Education. 2016; 41(12): 2156–2168. https://doi.org/10.1080/03075079.2015.1026890.
16. Srivarathan A, Jørgensen TSH, Lund R, Nygaard SS, Kristiansen M. They are breaking us into pieces: A longitudinal multi-method study on urban regeneration and place-based social relations among social housing residents in Denmark. Health & Place. 2023; 79, 102965. <https://doi.org/10.1016/j.healthplace.2023.102965>.
17. Institute of Health Information and Statistics of the Czech Republic. Psychiatric Yearbook 2022. 2022. <https://www.uzis.cz/res/f/008442/psych2022.pdf>. Accessed 25 Jan 2025.
