# Supplementary material for: Analysis of university students’ perception of mental health
Source: BMC Public Health. 2025 Nov 10;25:3868. doi: 10.1186/s12889-025-25213-7 (PMC12599076; doi:10.1186/s12889-025-25213-7)
Supplement: Supplementary file 3 — Supplementary Material 3. [file 12889_2025_25213_MOESM3_ESM.pdf]

Questionnaire: Mental Health Care and Factors Influencing Mental State (Ages 18–25), University Students

Dear Respondents,

Thank you for participating in this questionnaire, which aims to explore how young people perceive mental health care and which factors they believe influence their mental well-being.

Respondent Information:

- **Age:** Please select your age from the dropdown menu (18–25, over 25)
- **Gender:** Please select your gender from the dropdown menu (male, female)
- **Region of Permanent Residence:** Please select your region from the dropdown menu

1. How would you rate your current mental state?
(1 = Very poor, 5 = Very good)

- 1
- 2
- 3
- 4
- 5

2. If you sought professional help for your mental health (e.g., psychologist, psychiatrist), how would you rate the quality of these services? *(1 = Very dissatisfied, 5 = Very satisfied)*

- 1
- 2
- 3
- 4
- 5
- I have never sought professional help

3. Do you think mental health care is easily accessible in your city/region?

- Yes, without any problems
- Yes, but with some difficulties (e.g., waiting times)
- No, it is difficult to access professional care
- I don't know / I have no experience

4. Which factors do you think affect the mental health of young people aged 18–25 the most? *(select the 3 most important)*

- Economic conditions (e.g., financial stress)
- Social media and technology
- Academic or work-related pressure
- Family relationships
- Loneliness and social isolation
- Crime and sense of insecurity
- Limited access to health care
- Other (please specify)

5. Do you live in an urban or rural environment?

- Urban environment
- Rural environment
- Mixed (on the outskirts of a city or in small towns)

6. What benefits of living in the city do you think have a positive impact on mental health? *(select the 3 most important)*

- Easier access to medical and mental health care
- More social and cultural opportunities (cinemas, concerts, cafés, etc.)
- Access to jobs and education
- Greater anonymity and privacy
- Better access to public transportation
- Opportunities to build more social connections
- Other (please specify)

7. What disadvantages of living in the city do you think deteriorate mental health? *(select the 3 most important)*

- Higher levels of stress and fast-paced lifestyle
- More noise and pollution
- Lack of nature and peaceful spaces
- Greater anonymity and feelings of loneliness
- Higher crime rates
- More competitive job market
- Other (please specify)

8. If you could choose, where would you prefer to live in terms of mental health?

- In a city
- In the countryside
- A combination of urban and rural living
- I have no preference

9. How important is support from family and friends to you in dealing with mental health problems?
1 = Not important, 5 = Very important

- 1
- 2
- 3
- 4
- 5

10. Do you think the age at which people enter parenthood can affect the mental health of their children?

- Yes, older parental age improves children's mental health
- Yes, younger parental age improves children's mental health
- No, parental age has no effect
- I don't know

11. How much of an impact do you think crime in your region has on your mental health?
1 = No effect, 5 = Very strong effect

- 1
- 2
- 3
- 4
- 5

12. Do you think the age of the first-time mother can affect her mental health?

- Yes, a younger age (up to 21) may negatively affect her mental health
- Yes, an older age (over 21) may positively affect her mental health
- No, maternal age has no effect on her mental health
- I don't know

13. How would you rate the level of general awareness and education about mental health in secondary schools and colleges?
1 = Very poor, 5 = Very good

- 1
- 2
- 3
- 4
- 5

14. Which of the following do you think would be most helpful in improving mental health care for young people in your region? *(select up to 2 options)*

- Improving access to professional care (more therapists, shorter waiting times)
- Making mental health care more affordable
- Raising awareness through mental health campaigns
- Providing more support at schools and universities
- Creating a better preventive care system
- Other (please specify)

15. How often do you feel you have to look after your mental health on your own, without the help of professionals or family?

- Never
- Occasionally
- Often
- Always
