# Supplementary material for: Analysis of university students’ perception of mental health
Source: BMC Public Health. 2025 Nov 10;25:3868. doi: 10.1186/s12889-025-25213-7 (PMC12599076; doi:10.1186/s12889-025-25213-7)
Supplement: Supplementary file 4 — Supplementary Material 4. [file 12889_2025_25213_MOESM4_ESM.pdf]

Age	Gender	Region of Permanent Residence:	Q1. How would you rate your current mental state? (1 = Very poor, 5 = Very good)	Q2. If you sought professional help for your mental health (e.g., psychologist, psychiatrist), how would you rate the quality of these services? (1 = Very dissatisfied, 5 = Very satisfied)	Q3. Do you think mental health care is easily accessible in your city/region?	Q4. Which factors do you think affect the mental health of young people aged 18–25 the most? (select the 3 most important)	Q5. Do you live in an urban or rural environment?	Q6. What benefits of living in the city do you think have a positive impact on mental health? (select the 3 most important)	Q7. What disadvantages of living in the city do you think deteriorate mental health? (select the 3 most important)	Q8. If you could choose, where would you prefer to live in terms of mental health?	Q9. How important is support from family and friends to you in dealing with mental health problems? (1 = Not important, 5 = Very important)	Q10. Do you think the age at which people enter parenthood can affect the mental health of their children?	Q11. How much of an impact do you think crime in your region has on your mental health? (1 = No effect, 5 = Very strong effect)	Q12. Do you think the age of the first-time mother can affect her mental health?	Q13.How would you rate the level of general awareness and education about mental health in secondary schools and colleges? (1 = Very poor, 5 = Very good)	Q14. Which of the following do you think would be most helpful in improving mental health care for young people in your region? (select up to 2 options)	Q15. How often do you feel you have to look after your mental health on your own, without the help of professionals or family?
25	2	Ústecký kraj	4	0	2		Urban environment	Easier access to medical and mental health care	Higher levels of stress and pace of life	In the countryside	5	Yes, older parental age improves children's mental health	4	Yes, a younger age (up to 21) may negatively affect her mental health	3	Improved access to specialist care (more therapists, shorter waiting times)	Occasionally
19	2	Pardubice Region	4	4	1	Economic conditions (e.g., financial hardship), social media and technology, loneliness, and social isolation	Mixed (on the outskirts of a city or in small towns)		Higher levels of stress and pace of life, More noise and pollution, Greater anonymity and feelings of loneliness	In the countryside	5	Yes, younger parental age improves children's mental health	2	Yes, an older age (over 21) may positively affect her mental health	2	Greater support in schools and universities, Creation of a better preventive care system	Often
26	1	Karlovarský kraj	5	4	0	Economic conditions (e.g., financial hardship), social media and technology, loneliness, and social isolation	Rural environment	Greater social and cultural opportunities (cinemas, concerts, cafés, etc.), Greater anonymity and privacy, Opportunity to establish more social contacts	Higher levels of stress and pace of life, More noise and pollution, Lack of nature and quiet places	A combination of urban and rural living	3	I don't know	2	Yes, a younger age (up to 21) may negatively affect her mental health	3	Greater support in schools and universities, Creation of a better preventive care system	Never
20	2	Moravian-Silesian Region	3	0	0	Economic conditions (e.g., financial hardship), social media and technology, loneliness, and social isolation	Mixed (on the outskirts of a city or in small towns)	Greater social and cultural opportunities (cinemas, concerts, cafés, etc.), better access to public transport, opportunity to make more social contacts	Higher levels of stress and pace of life, More noise and pollution, Lack of nature and quiet places	In the countryside	4	Yes, younger parental age improves children's mental health	4	Yes, a younger age (up to 21) may negatively affect her mental health	3	Improving the affordability of mental health care, Greater support in schools and universities	Often
22	2	Moravian-Silesian Region	4	3	1	Economic conditions (e.g., financial hardship), social media and technology, loneliness, and social isolation	Urban environment	Easier access to medical and mental health care, Greater social and cultural opportunities (cinemas, concerts, cafés, etc.), Greater choice of shops, restaurants, entertainment	Higher levels of stress and pace of life, Lack of nature and quiet places, Greater anonymity and feelings of loneliness	A combination of urban and rural living	2	I don't know	2	Yes, a younger age (up to 21) may negatively affect her mental health	2	Improved access to specialist care (more therapists, shorter waiting times), Improved affordability of mental health care	Often
25	2	Moravian-Silesian Region	4	0	2	Economic conditions (e.g., financial hardship), social media and technology, loneliness, and social isolation	Urban environment		Higher levels of stress and pace of life, Lack of nature and quiet places, Greater anonymity and feelings of loneliness	A combination of urban and rural living	5	No, parental age has no effect	3	Yes, an older age (over 21) may positively affect her mental health	3	Improving access to specialist care (more therapists, shorter waiting times), creating a better preventive care system	Often
22	2	Moravian-Silesian Region	3	0	2	Economic conditions (e.g., financial hardship), social media and technology, loneliness, and social isolation	Mixed (on the outskirts of a city or in small towns)	Greater social and cultural opportunities (cinemas, concerts, cafés, etc.), Greater anonymity and privacy, Opportunity to establish more social contacts	Lack of nature and quiet places, lots of people, light pollution at night	A combination of urban and rural living	4	No, parental age has no effect	1	No, maternal age has no effect on her mental health	3	Improving the affordability of mental health care, Creating a better preventive care system	Always
23	2	Moravian-Silesian Region	1	2	2	Economic conditions (e.g., financial hardship), social media and technology, loneliness, and social isolation	Urban environment	Easier access to medical and mental health care, Accessibility of employment and education, Greater anonymity and privacy	Higher levels of stress and pace of life, More noise and pollution, Lack of nature and quiet places	A combination of urban and rural living	4	I don't know	1	I don't know	1	Improved access to specialist care (more therapists, shorter waiting times), Improved affordability of mental health care	Always
23	2	Moravian-Silesian Region	4	0	0	Economic conditions (e.g., financial hardship), social media and technology, loneliness, and social isolation	Mixed (on the outskirts of a city or in small towns)	Greater social and cultural opportunities (cinemas, concerts, cafés, etc.), Opportunity to establish more social contacts	More noise and pollution, Lack of nature and quiet places	A combination of urban and rural living	3	Yes, older parental age improves children's mental health	2	Yes, a younger age (up to 21) may negatively affect her mental health	3	Greater support in schools and universities, Creation of a better preventive care system	Occasionally
23	2	Zlín Region	4	0	0	Economic conditions (e.g., financial hardship), social media and technology, loneliness, and social isolation	Rural environment	Greater social and cultural opportunities (cinemas, concerts, cafés, etc.), Accessibility of employment and education, Better accessibility of public transport		A combination of urban and rural living	4	Yes, younger parental age improves children's mental health	3	Yes, a younger age (up to 21) may negatively affect her mental health	2	Improving access to specialist care (more therapists, shorter waiting times), creating a better preventive care system	Occasionally
24	2	Central Bohemian Region	2	0	0	Economic conditions (e.g., financial hardship), social media and technology, loneliness, and social isolation	Urban environment	Accessibility of employment and education, Better accessibility of public transport	Higher levels of stress and pace of life, Lack of nature and quiet places, Greater competitive pressure in the job market	A combination of urban and rural living	2	No, parental age has no effect	2	No, maternal age has no effect on her mental health	2	zpomalení pokroku doby - rodiče kteří ví která bije, každý by ale měl být schopen se postarat sám o svoje zdraví	Always
22	2	Moravian-Silesian Region	3	0	0	Economic conditions (e.g., financial hardship), social media and technology, loneliness, and social isolation	Mixed (on the outskirts of a city or in small towns)	Greater social and cultural opportunities (cinemas, concerts, cafés, etc.), Accessibility of employment and education, Opportunity to establish more social contacts	Higher levels of stress and pace of life, Lack of nature and quiet places, Greater competitive pressure in the job market	In the countryside	5	Yes, older parental age improves children's mental health	1	Yes, a younger age (up to 21) may negatively affect her mental health	2	Raising awareness of mental health through campaigns, Creating a better preventive care system	Always
23	2	Moravian-Silesian Region	2	0	0	Economic conditions (e.g., financial hardship), social media and technology, loneliness, and social isolation	Mixed (on the outskirts of a city or in small towns)	Greater social and cultural opportunities (cinemas, concerts, cafés, etc.), Accessibility of employment and education, Better accessibility of public transport	Higher levels of stress and pace of life, More noise and pollution, Lack of nature and quiet places	In the countryside	3	No, parental age has no effect	1	Yes, a younger age (up to 21) may negatively affect her mental health	1	Raising awareness of mental health through campaigns, Creating a better preventive care system	Always
20	1	Vysočina Region	4	0	2	Economic conditions (e.g., financial hardship), social media and technology, loneliness, and social isolation	Mixed (on the outskirts of a city or in small towns)	Easier access to medical and mental health care, Greater social and cultural opportunities (cinemas, concerts, cafés, etc.), Accessibility of employment and education, Better accessibility of public transport	Greater competitive pressure in the labor market	In a city	4	Yes, older parental age improves children's mental health	2	Yes, a younger age (up to 21) may negatively affect her mental health	2	Improving the affordability of mental health care, Raising awareness of mental health through campaigns	Often
22	2	Zlín Region	4	0	0	Economic conditions (e.g., financial hardship), social media and technology, loneliness, and social isolation	Mixed (on the outskirts of a city or in small towns)	Greater social and cultural opportunities (cinemas, concerts, cafés, etc.), Accessibility of employment and education, Opportunity to establish more social contacts	Higher levels of stress and pace of life, More noise and pollution, Lack of nature and quiet places	A combination of urban and rural living	3	Yes, older parental age improves children's mental health	1	Yes, a younger age (up to 21) may negatively affect her mental health	3	Greater support in schools and universities, Creation of a better preventive care system	Often
20	1	Zlín Region	3	0	2	Economic conditions (e.g., financial hardship), social media and technology, loneliness, and social isolation	Rural environment	Accessibility of employment and education, Better accessibility of public transport, Opportunity to establish more social contacts	Higher levels of stress and pace of life, More noise and pollution, Lack of nature and quiet places	In the countryside	5	Yes, older parental age improves children's mental health	1	I don't know	2	Greater support in schools and universities	Occasionally
20	1	Moravian-Silesian Region	2	1	1	Economic conditions (e.g., financial hardship), social media and technology, loneliness, and social isolation	Mixed (on the outskirts of a city or in small towns)	Easier access to medical and mental health care, Greater social and cultural opportunities (cinemas, concerts, cafés, etc.), Better public transport accessibility	Higher levels of stress and pace of life, More noise and pollution, Lack of nature and quiet places	In the countryside	3	Yes, older parental age improves children's mental health	5	Yes, a younger age (up to 21) may negatively affect her mental health	1		Often
20	2	Moravian-Silesian Region	3	0	0	Economic conditions (e.g., financial hardship), social media and technology, loneliness, and social isolation	Urban environment	Easier access to medical and mental health care, Accessibility of employment and education, Opportunity to establish more social contacts	Higher levels of stress and pace of life, Greater anonymity and feelings of loneliness, Greater competitive pressure in the job market	A combination of urban and rural living	2	Yes, older parental age improves children's mental health	2	Yes, an older age (over 21) may positively affect her mental health	3	Improving access to specialist care (more therapists, shorter waiting times), creating a better preventive care system	Often
21	2	Olomouc Region	4	0	3	Economic conditions (e.g., financial hardship), social media and technology, loneliness, and social isolation	Rural environment			In the countryside	4	No, parental age has no effect	1	Yes, an older age (over 21) may positively affect her mental health	2	Greater support in schools and universities, Creation of a better preventive care system	Often
22	1	Central Bohemian Region	4	0	1	Economic conditions (e.g., financial hardship), social media and technology, loneliness, and social isolation	Rural environment	Easier access to medical and mental health care, Accessibility of employment and education, Better accessibility of public transport		A combination of urban and rural living	4	I don't know	2	Yes, a younger age (up to 21) may negatively affect her mental health	2	Improved access to specialist care (more therapists, shorter waiting times), Improved affordability of mental health care	Often
20	2	South Moravian Region	1	4	0	Economic conditions (e.g., financial hardship), social media and technology, loneliness, and social isolation	Urban environment	Accessibility of employment and education, Greater anonymity and privacy, Better accessibility of public transport	Higher levels of stress and pace of life, More noise and pollution, Lack of nature and quiet places	In a city	3	Yes, older parental age improves children's mental health	1	I don't know	2	Improving access to professional care (more therapists, shorter waiting times), Raising awareness of mental health through campaigns	Always
20	1	South Moravian Region	5	0	0	Economic conditions (e.g., financial hardship), social media and technology, loneliness, and social isolation	Urban environment		More noise and pollution, Lack of nature and quiet places, Greater competitive pressure in the job market	A combination of urban and rural living	4	Yes, older parental age improves children's mental health	1	Yes, a younger age (up to 21) may negatively affect her mental health	3	Raising awareness of mental health through campaigns, Greater support in schools and universities	Occasionally
20	1	Ústí nad Labem Region	3	0	0	Economic conditions (e.g., financial hardship), social media and technology, loneliness, and social isolation	Urban environment	Easier access to medical and mental health care, Greater social and cultural opportunities (cinemas, concerts, cafés, etc.), Opportunity to establish more social contacts	More noise and pollution, Lack of nature and quiet places, Greater competitive pressure in the job market	In a city	4	I don't know	2	Yes, a younger age (up to 21) may negatively affect her mental health	3	Improved access to specialist care (more therapists, shorter waiting times), Improved affordability of mental health care	Always
22	2	Pardubice Region	4	0	0	Economic conditions (e.g., financial hardship), social media and technology, loneliness, and social isolation	Mixed (on the outskirts of a city or in small towns)	Greater social and cultural opportunities (cinemas, concerts, cafés, etc.), Greater anonymity and privacy, Opportunity to establish more social contacts		A combination of urban and rural living	5	I don't know	2	Yes, an older age (over 21) may positively affect her mental health	3	Improving access to professional care (more therapists, shorter waiting times), Raising awareness of mental health through campaigns	Never
21	2	Capital City of Prague	3	0	3	Economic conditions (e.g., financial hardship), social media and technology, loneliness, and social isolation	Urban environment	Easier access to medical and mental health care, Accessibility of employment and education, Opportunity to establish more social contacts	Higher levels of stress and pace of life, Greater anonymity and feelings of loneliness, Greater competitive pressure in the job market	In a city	4	Yes, older parental age improves children's mental health	1	Yes, a younger age (up to 21) may negatively affect her mental health	2	Improving the affordability of mental health care, Raising awareness of mental health through campaigns	Often
20	1	Vysočina Region	2	0	0	Economic conditions (e.g., financial hardship), social media and technology, loneliness, and social isolation	Urban environment			A combination of urban and rural living	5	I don't know	2	Yes, a younger age (up to 21) may negatively affect her mental health	2	Improving the affordability of mental health care, Raising awareness of mental health through campaigns	Occasionally
25	1	Moravian-Silesian Region	3	4	2	Economic conditions (e.g., financial hardship), social media and technology, loneliness, and social isolation	Mixed (on the outskirts of a city or in small towns)	Easier access to medical and mental health care, Greater social and cultural opportunities (cinemas, concerts, cafés, etc.), Accessibility of employment and education		A combination of urban and rural living	5	I don't know	3	I don't know	2	Greater support in schools and universities, Creation of a better preventive care system	Often
21	1	Moravian-Silesian Region	2	3	2	Economic conditions (e.g., financial hardship), social media and technology, loneliness, and social isolation	Mixed (on the outskirts of a city or in small towns)	Greater social and cultural opportunities (cinemas, concerts, cafés, etc.), Accessibility of employment and education, Greater anonymity and privacy	Higher levels of stress and pace of life, Lack of nature and quiet places, Greater competitive pressure in the job market	In a city	5	Yes, younger parental age improves children's mental health	2	Yes, a younger age (up to 21) may negatively affect her mental health	3	Improving access to specialist care (more therapists, shorter waiting times), creating a better preventive care system	Often
21	1	Central Bohemian Region	4	0	2	Economic conditions (e.g., financial hardship), social media and technology, loneliness, and social isolation	Mixed (on the outskirts of a city or in small towns)	Greater social and cultural opportunities (cinemas, concerts, cafés, etc.), Greater anonymity and privacy, Better access to public transport	Lack of nature and quiet places, Higher crime rate	A combination of urban and rural living	5	Yes, older parental age improves children's mental health	3	Yes, a younger age (up to 21) may negatively affect her mental health	1	Improved access to professional care (more therapists, shorter waiting times), Greater support in schools and universities	Always
24	1	Moravian-Silesian Region	3	0	0	Economic conditions (e.g., financial hardship), social media and technology, loneliness, and social isolation	Urban environment	Easier access to medical and mental health care, Greater social and cultural opportunities (cinemas, concerts, cafés, etc.), Accessibility of employment and education	Higher levels of stress and pace of life, Lack of nature and quiet places, Greater anonymity and feelings of loneliness	A combination of urban and rural living	5	I don't know	1	I don't know	3	Improved access to specialist care (more therapists, shorter waiting times), Improved affordability of mental health care	Often
23	2	Moravian-Silesian Region	2	2	2	Economic conditions (e.g., financial pressure), social media and technology, family relationships	Rural environment	Greater social and cultural opportunities (cinemas, concerts, cafés, etc.), Accessibility of employment and education, Opportunity to establish more social contacts	More noise and pollution, greater anonymity and feelings of loneliness	In the countryside	4	Yes, younger parental age improves children's mental health	2	Yes, an older age (over 21) may positively affect her mental health	2	Raising awareness of mental health through campaigns, Greater support in schools and universities, Creating a better preventive care system	Often
20	1	Capital City of Prague	4	0	2	Economic conditions (e.g., financial pressure), social media and technology, family relationships	Urban environment	Greater social and cultural opportunities (cinemas, concerts, cafés, etc.), Accessibility of employment and education, Opportunity to establish more social contacts	Higher levels of stress and pace of life, More noise and pollution, Greater competitive pressure in the job market	A combination of urban and rural living	5	Yes, older parental age improves children's mental health	1	Yes, a younger age (up to 21) may negatively affect her mental health	2	Improving access to specialist care (more therapists, shorter waiting times), improving the affordability of mental health care, raising awareness of mental health through campaigns	Often
23	1	Moravian-Silesian Region	5	0	0	Economic conditions (e.g., financial pressure), social media and technology, family relationships	Rural environment	Greater social and cultural opportunities (cinemas, concerts, cafés, etc.), Accessibility of employment and education, Better accessibility of public transport	Higher levels of stress and pace of life, More noise and pollution, Lack of nature and quiet places	In the countryside	5	No, parental age has no effect	3	I don't know	3	Greater support in schools and universities, Creation of a better preventive care system	Never
24	2	Moravian-Silesian Region	5	3	1	Economic conditions (e.g., financial pressure), social media and technology, family relationships	Urban environment	Greater social and cultural opportunities (cinemas, concerts, cafés, etc.), Accessibility of employment and education, Better accessibility of public transport	Higher levels of stress and pace of life, Lack of nature and quiet places, Higher crime rates	A combination of urban and rural living	1	Yes, older parental age improves children's mental health	5	Yes, a younger age (up to 21) may negatively affect her mental health	1	Improving the affordability of mental health care, Raising awareness of mental health through campaigns	Always
26	2	Moravian-Silesian Region	5	0	0	Economic conditions (e.g., financial pressure), social media and technology, family relationships	Mixed (on the outskirts of a city or in small towns)	Easier access to medical and mental health care, Greater social and cultural opportunities (cinemas, concerts, cafés, etc.), Better public transport accessibility		A combination of urban and rural living	4	Yes, younger parental age improves children's mental health	3	Yes, a younger age (up to 21) may negatively affect her mental health	3	Creating a better preventive care system	Never
20	2	Hradec Králové Region	5	0	0	Economic conditions (e.g., financial pressure), social media and technology, family relationships	Urban environment	Greater social and cultural opportunities (cinemas, concerts, cafés, etc.), better access to public transport, opportunity to make more social contacts	Higher levels of stress and pace of life, More noise and pollution, Lack of nature and quiet places	In the countryside	5	Yes, older parental age improves children's mental health	3	No, maternal age has no effect on her mental health	2	Greater support in schools and universities, Creation of a better preventive care system	Often
25	1	Moravian-Silesian Region	4	0	1	Economic conditions (e.g., financial pressure), social media and technology, family relationships	Mixed (on the outskirts of a city or in small towns)	Accessibility of employment and education, Better accessibility of public transport, Opportunity to establish more social contacts	Higher levels of stress and pace of life, More noise and pollution, Lack of nature and quiet places	A combination of urban and rural living	5	Yes, younger parental age improves children's mental health	3	Yes, an older age (over 21) may positively affect her mental health	2	Improved access to specialist care (more therapists, shorter waiting times), Improved affordability of mental health care	Often
20	1	Olomouc Region	3	0	0	Economic conditions (e.g., financial pressure), social media and technology, family relationships	Rural environment	Greater anonymity and privacy, Better access to public transport	Higher crime rate	A combination of urban and rural living	2	I don't know	1	I don't know	1	Improved access to specialist care (more therapists, shorter waiting times), Improved affordability of mental health care	Often
19	2	Zlín Region	4	0	0	Economic conditions (e.g., financial pressure), social media and technology, family relationships	Urban environment	Greater social and cultural opportunities (cinemas, concerts, cafés, etc.), Accessibility of employment and education, Better accessibility of public transport	Higher levels of stress and pace of life, Lack of nature and quiet places	A combination of urban and rural living	2	I don't know	3	Yes, a younger age (up to 21) may negatively affect her mental health	2	Improving the affordability of mental health care, Raising awareness of mental health through campaigns	Always
19	1	Moravian-Silesian Region	5	0	1	Economic conditions (e.g., financial pressure), social media and technology, family relationships	Rural environment	Greater social and cultural opportunities (cinemas, concerts, cafés, etc.), better access to public transport, opportunity to make more social contacts	Higher levels of stress and pace of life, More noise and pollution, Lack of nature and quiet places	In the countryside	4	No, parental age has no effect	2	No, maternal age has no effect on her mental health	3	Improving the affordability of mental health care, Creating a better preventive care system	Always
26	1	Zlín Region	2	3	2	Economic conditions (e.g., financial pressure), social media and technology, family relationships	Urban environment	Easier access to medical and mental health care, Greater social and cultural opportunities (cinemas, concerts, cafés, etc.), Accessibility of employment and education	Higher levels of stress and pace of life, More noise and pollution, Lack of nature and quiet places	A combination of urban and rural living	4	Yes, older parental age improves children's mental health	2	Yes, a younger age (up to 21) may negatively affect her mental health	2	Improving the affordability of mental health care, Greater support in schools and universities	Always
21	1	South Moravian Region	3	4	2	Economic conditions (e.g., financial pressure), social media and technology, family relationships	Rural environment	Easier access to medical and mental health care, Greater social and cultural opportunities (cinemas, concerts, cafés, etc.), Accessibility of employment and education	Higher levels of stress and pace of life, More noise and pollution, Lack of nature and quiet places	A combination of urban and rural living	5	Yes, older parental age improves children's mental health	3	Yes, a younger age (up to 21) may negatively affect her mental health	3	Improved access to specialist care (more therapists, shorter waiting times), improved affordability of mental health care, greater support in schools and universities	Occasionally
22	1	South Moravian Region	3	3	1	Economic conditions (e.g., financial pressure), social media and technology, family relationships	Mixed (on the outskirts of a city or in small towns)	Easier access to medical and mental health care, Greater social and cultural opportunities (cinemas, concerts, cafés, etc.), Accessibility of employment and education	Lack of nature and quiet places, Greater anonymity and feelings of loneliness, Higher crime rates	A combination of urban and rural living	3	Yes, older parental age improves children's mental health	3	Yes, an older age (over 21) may positively affect her mental health	3	Improving the affordability of mental health care, Raising awareness of mental health through campaigns	Occasionally
23	2	Vysočina Region	4	0	3	Economic conditions (e.g., financial pressure), social media and technology, family relationships	Urban environment	Greater social and cultural opportunities (cinemas, concerts, cafés, etc.), better access to public transport, opportunity to make more social contacts	Higher levels of stress and pace of life, Greater anonymity and feelings of loneliness, Higher crime rates	I have no preference	5	No, parental age has no effect	1	No, maternal age has no effect on her mental health	3	Raising awareness of mental health through campaigns, creating a better preventive care system, increasing parents' awareness of the issue.	Occasionally
22	1	Liberecký kraj	4	0	0	Economic conditions (e.g., financial pressure), social media and technology, family relationships	Urban environment	Easier access to medical and mental health care, Greater social and cultural opportunities (cinemas, concerts, cafés, etc.), Accessibility of employment and education, Better accessibility of public transport	Higher levels of stress and pace of life, Higher crime rates	A combination of urban and rural living	4	No, parental age has no effect	4	No, maternal age has no effect on her mental health	4	Improving the affordability of mental health care, Greater support in schools and universities, Creating a better preventive care system	Often
23	1	Moravian-Silesian Region	4	0	2	Economic conditions (e.g., financial pressure), social media and technology, family relationships	Urban environment	Easier access to medical and mental health care, Greater social and cultural opportunities (cinemas, concerts, cafés, etc.), Greater anonymity and privacy	Higher levels of stress and pace of life, Higher crime rates, Greater competitive pressure in the labor market	A combination of urban and rural living	5	I don't know	2	Yes, a younger age (up to 21) may negatively affect her mental health	2	Greater support in schools and universities, Creation of a better preventive care system	Often
25	1	Moravian-Silesian Region	4	4	2	Economic conditions (e.g., financial pressure), social media and technology, family relationships, crime, and feelings of insecurity	Mixed (on the outskirts of a city or in small towns)	Easier access to medical and mental health care, Accessibility of employment and education	More noise and pollution, higher crime rates	In the countryside	3	Yes, older parental age improves children's mental health	4	Yes, a younger age (up to 21) may negatively affect her mental health	3	Improved access to specialist care (more therapists, shorter waiting times), Improved affordability of mental health care	Often
22	2	Moravian-Silesian Region	2	0	0	Economic conditions (e.g., financial pressure), social media and technology, family relationships, drug intoxication	Urban environment			In a city	2	I don't know	3	Yes, a younger age (up to 21) may negatively affect her mental health	5	Greater support in schools and universities, Creation of a better preventive care system	Always

Age	Gender	Region of Permanent Residence:	Q1. How would you rate your current mental state? (1 = Very poor, 5 = Very good)	Q2. If you sought professional help for your mental health (e.g., psychologist, psychiatrist), how would you rate the quality of these services? (1 = Very dissatisfied, 5 = Very satisfied)	Q3. Do you think mental health care is easily accessible in your city/region?	Q4: Which factors do you think affect the mental health of young people aged 18–25 the most? (select the 3 most important)	Q5. Do you live in an urban or rural environment?	Q6. What benefits of living in the city do you think have a positive impact on mental health? (select the 3 most important)	Q7. What disadvantages of living in the city do you think deteriorate mental health? (select the 3 most important)	Q8. If you could choose, where would you prefer to live in terms of mental health?	Q9. How important is support from family and friends to you in dealing with mental health problems? (1 = Not important, 5 = Very important)	Q10. Do you think the age at which people enter parenthood can affect the mental health of their children?	Q11. How much of an impact do you think crime in your region has on your mental health? (1 = No effect, 5 = Very strong effect)	Q12. Do you think the age of the first-time mother can affect her mental health?	Q13.How would you rate the level of general awareness and education about mental health in secondary schools and colleges? (1 = Very poor, 5 = Very good)	Q14. Which of the following do you think would be most helpful in improving mental health care for young people in your region? (select up to 2 options)	Q15. How often do you feel you have to look after your mental health on your own, without the help of professionals or family?
22	2	Moravian-Silesian Region	4	0	0	Economic conditions (e.g., financial pressure), social media and technology, family relationships, Insufficient access to healthcare	Urban environment	Greater social and cultural opportunities (cinemas, concerts, cafés, etc.), Accessibility of employment and education, Better accessibility of public transport	Higher levels of stress and pace of life, More noise and pollution, Greater anonymity and feelings of loneliness	I have no proference	5	Yes, older parental age improves children's mental health	1	Yes, a younger age (up to 21) may negatively affect her mental health	2	Improving the affordability of mental health care, Greater support in schools and universities	Always
26	2	Capital City of Prague	3	4	3	Economic conditions (e.g., financial pressure), social media and technology, family relationships, loneliness and social isolation	Urban environment	Greater social and cultural opportunities (cinemas, concerts, cafés, etc.), Accessibility of employment and education, Better accessibility of public transport	Higher levels of stress and pace of life, Greater anonymity and feelings of loneliness, Higher crime rates	In a city	2	Yes, older parental age improves children's mental health	3	Yes, a younger age (up to 21) may negatively affect her mental health	1	Improving the affordability of mental health care, Greater support in schools and universities	Often
24	2	Moravian-Silesian Region	4	0	2	Economic conditions (e.g., financial pressure), social media and technology, family relationships, loneliness and social isolation	Urban environment	Easier access to medical and mental health care, Greater social and cultural opportunities (cinemas, concerts, cafés, etc.)	Higher levels of stress and pace of life, More noise and pollution, Lack of nature and quiet places	A combination of urban and rural living	4	Yes, older parental age improves children's mental health	2	Yes, a younger age (up to 21) may negatively affect her mental health	3	Improving access to professional care (more therapists, shorter waiting times), Raising awareness of mental health through campaigns, Greater support in schools and universities	Often
25	2	Moravian-Silesian Region	4	5	2	Economic conditions (e.g., financial pressure), social media and technology, family relationships, loneliness and social isolation	Urban environment		Higher levels of stress and pace of life, More noise and pollution, Lack of nature and quiet places	A combination of urban and rural living	5	I don't know	1	I don't know	3	Improving the affordability of mental health care, Creating a better preventive care system	Often
20	1	Moravian-Silesian Region	5	0	0	Economic conditions (e.g., financial pressure), social media and technology, family relationships, loneliness and social isolation	Mixed (on the outskirts of a city or in small towns)	Easier access to medical and mental health care, Greater social and cultural opportunities (cinemas, concerts, cafés, etc.), Accessibility of employment and education	Higher levels of stress and pace of life, More noise and pollution, Lack of nature and quiet places	A combination of urban and rural living	5	No, parental age has no effect	1	I don't know	2	Improving access to professional care (more therapists, shorter waiting times), Raising awareness of mental health through campaigns, Greater support in schools and universities	Occasionally
22	2	Zlín Region	4	0	1	Economic conditions (e.g., financial pressure), social media and technology, family relationships, loneliness and social isolation	Mixed (on the outskirts of a city or in small towns)	Easier access to medical and mental health care, Greater anonymity and privacy, Better public transport accessibility, Opportunity to establish more social contacts	Higher levels of stress and pace of life, More noise and pollution, Lack of nature and quiet places	In a city	2	Yes, younger parental age improves children's mental health	2	Yes, a younger age (up to 21) may negatively affect her mental health	2	Improved access to specialist care (more therapists, shorter waiting times), greater support in schools and universities, creation of a better preventive care system.	Often
22	2	South Moravian Region	4	0	1	Economic conditions (e.g., financial pressure), social media and technology, family relationships, loneliness and social isolation	Urban environment	Greater social and cultural opportunities (cinemas, concerts, cafés, etc.), Greater anonymity and privacy, Opportunity to establish more social contacts	More noise and pollution, Lack of nature and quiet places, Greater anonymity and feelings of loneliness	In the countryside	4	Yes, older parental age improves children's mental health	2	Yes, a younger age (up to 21) may negatively affect her mental health	2	Improved access to specialist care (more therapists, shorter waiting times), Improved affordability of mental health care	Occasionally
22	1	Zlín Region	4	4	2	Economic conditions (e.g., financial pressure), social media and technology, family relationships, loneliness and social isolation	Rural environment	Greater social and cultural opportunities (cinemas, concerts, cafés, etc.), Accessibility of employment and education, Greater anonymity and privacy	Higher levels of stress and pace of life, More noise and pollution, Lack of nature and quiet places	In the countryside	5	I don't know	1	Yes, a younger age (up to 21) may negatively affect her mental health	3	Improving the affordability of mental health care, Creating a better preventive care system	Occasionally
21	2	South Moravian Region	1	0	1	Economic conditions (e.g., financial pressure), social media and technology, family relationships, loneliness and social isolation, appearance and social standards	Urban environment	Easier access to medical and mental health care, Greater social and cultural opportunities (cinemas, concerts, cafés, etc.), Accessibility of employment and education, Greater anonymity and privacy, Better accessibility of public transport, Opportunity to establish more social contacts	More noise and pollution, Lack of nature and quiet places, Higher crime rates, Greater competitive pressure in the job market	A combination of urban and rural living	5	Yes, older parental age improves children's mental health	3	Yes, a younger age (up to 21) may negatively affect her mental health	1	Improving the affordability of mental health care, Greater support in schools and universities	Always
26	2	Ústí nad Labem Region	5	0	0	Economic conditions (e.g., financial stress)	Urban environment	Access to employment and education		A combination of urban and rural living	5	Yes, older parental age improves children's mental health	3	Yes, a younger age (up to 21) may negatively affect her mental health	1	Improving access to specialist care (more therapists, shorter waiting times), improving the affordability of mental health care, raising awareness of mental health through campaigns, greater support in schools and universities, creating a better preventive care system.	Never
24	2	Olomouc Region	3	0	0	Economic conditions (e.g., financial stress)	Urban environment	Easier access to medical and mental health care	Higher levels of stress and pace of life, More noise and pollution	In the countryside	2	Yes, older parental age improves children's mental health	2	No, maternal age has no effect on her mental health	5	Improved access to specialist care (more therapists, shorter waiting times)	Occasionally
19	1	Moravian-Silesian Region	5	2	2	Economic conditions (e.g., financial stress), Family relationships, Crime and feelings of insecurity	Urban environment		Higher levels of stress and pace of life, Higher crime rates, Greater competitive pressure in the labor market	I have no proference	5	No, parental age has no effect	3	Yes, a younger age (up to 21) may negatively affect her mental health	3		Often
26	2	Moravian-Silesian Region	4	0	0	Economic conditions (e.g., financial stress), Family relationships, Inadequate access to healthcare	Urban environment	Greater social and cultural opportunities (cinemas, concerts, cafés, etc.), Accessibility of employment and education, Better accessibility of public transport	Higher levels of stress and pace of life, More noise and pollution, Higher crime rates	In the countryside	5	Yes, older parental age improves children's mental health	3	Yes, a younger age (up to 21) may negatively affect her mental health	3	Raising awareness of mental health through campaigns, Creating a better preventive care system	Occasionally
23	1	Moravian-Silesian Region	3	0	2	Economic conditions (e.g., financial stress), Family relationships, Loneliness and social isolation	Mixed (on the outskirts of a city or in small towns)	Greater social and cultural opportunities (cinemas, concerts, cafés, etc.)	More noise and pollution, Lack of nature and quiet places, Higher crime rate	In the countryside	4	No, parental age has no effect	1	No, maternal age has no effect on her mental health	1	Improved access to professional care (more therapists, shorter waiting times), Greater support in schools and universities	Always
21	2	Moravian-Silesian Region	1	0	0	Economic conditions (e.g., financial stress), Family relationships, Loneliness and social isolation	Urban environment	Greater social and cultural opportunities (cinemas, concerts, cafés, etc.), better access to public transport, opportunity to make more social contacts	More noise and pollution, Greater anonymity and feelings of loneliness, Higher crime rates	A combination of urban and rural living	4	Yes, older parental age improves children's mental health	1	I don't know	3	Greater support in schools and universities, Creation of a better preventive care system	Always
25	2	Moravian-Silesian Region	5	4	2	Economic conditions (e.g., financial stress), Family relationships, Loneliness and social isolation	Mixed (on the outskirts of a city or in small towns)	Easier access to medical and mental health care, Greater social and cultural opportunities (cinemas, concerts, cafés, etc.), Accessibility of employment and education	More noise and pollution, Lack of nature and quiet places, Pressure on housing	A combination of urban and rural living	4	No, parental age has no effect	2	I don't know	1	Improved access to specialist care (more therapists, shorter waiting times), Improved affordability of mental health care	Always
23	1	Moravian-Silesian Region	3	4	1	Economic conditions (e.g., financial stress), Family relationships, Loneliness and social isolation	Mixed (on the outskirts of a city or in small towns)	Greater social and cultural opportunities (cinemas, concerts, cafés, etc.), Accessibility of employment and education, Opportunity to establish more social contacts	Higher levels of stress and pace of life, Greater anonymity and feelings of loneliness, Higher crime rates	In the countryside	5	Yes, older parental age improves children's mental health	3	Yes, a younger age (up to 21) may negatively affect her mental health	3	Greater support in schools and universities, Creation of a better preventive care system	Occasionally
20	1	Zlín Region	4	0	1	Economic conditions (e.g., financial stress), Family relationships, Loneliness and social isolation	Mixed (on the outskirts of a city or in small towns)	Greater social and cultural opportunities (cinemas, concerts, cafés, etc.), better access to public transport, opportunity to make more social contacts	Higher levels of stress and pace of life, More noise and pollution, Greater competitive pressure in the job market	In the countryside	4	I don't know	1	Yes, a younger age (up to 21) may negatively affect her mental health	1	Zlepšení dostupnosti odborné péče (více terapeutů, kratší čekací doby), Zvýšení povědomí o duševním zdraví prostřednictvím kampaní, Větší podpora na školách a univerzitách, Vytvoření lepšího systému preventivní péče	Always
20	2	Moravian-Silesian Region	3	0	0	Economic conditions (e.g., financial stress), Family relationships, Loneliness and social isolation	Urban environment	Easier access to medical and mental health care, Greater social and cultural opportunities (cinemas, concerts, cafés, etc.), Opportunity to establish more social contacts	Higher levels of stress and pace of life, Lack of nature and quiet places, Greater competitive pressure in the job market	A combination of urban and rural living	4	I don't know	1	Yes, a younger age (up to 21) may negatively affect her mental health	3	Improving access to specialist care (more therapists, shorter waiting times), creating a better preventive care system	Often
25	1	Olomouc Region	1	0	2	Economic conditions (e.g., financial stress), Family relationships, Loneliness and social isolation	Urban environment	Greater social and cultural opportunities (cinemas, concerts, cafés, etc.), Accessibility of employment and education, Greater anonymity and privacy	Higher levels of stress and pace of life, Higher crime rates, Greater competitive pressure in the labor market	In a city	3	I don't know	1	I don't know	1	Improved access to specialist care (more therapists, shorter waiting times), Improved affordability of mental health care	Always
22	2	Moravian-Silesian Region	4	0	0	Economic conditions (e.g., financial stress), Family relationships, Loneliness and social isolation	Urban environment	Greater social and cultural opportunities (cinemas, concerts, cafés, etc.), Accessibility of employment and education, Opportunity to establish more social contacts	Higher levels of stress and pace of life, Lack of nature and quiet places, Greater anonymity and feelings of loneliness	A combination of urban and rural living	5	Yes, older parental age improves children's mental health	2	No, maternal age has no effect on her mental health	1	Raising awareness of mental health through campaigns, Creating a better preventive care system	Always
22	2	Moravian-Silesian Region	3	0	0	Economic conditions (e.g., financial stress), Family relationships, Loneliness and social isolation	Urban environment	Greater social and cultural opportunities (cinemas, concerts, cafés, etc.), Accessibility of employment and education, Better accessibility of public transport	Higher levels of stress and pace of life, Lack of nature and quiet places, Greater competitive pressure in the job market	In a city	4	Yes, older parental age improves children's mental health	1	Yes, a younger age (up to 21) may negatively affect her mental health	2	Improving the affordability of mental health care, Greater support in schools and universities	Always
19	1	Olomouc Region	2	3	2	Economic conditions (e.g., financial stress), Family relationships, Loneliness and social isolation	Rural environment	Access to employment and education, Opportunity to establish more social contacts	Lack of nature and quiet places, Greater competitive pressure in the job market	A combination of urban and rural living	5	Yes, older parental age improves children's mental health	2	Yes, a younger age (up to 21) may negatively affect her mental health	4	Improving the affordability of mental health care, Greater support in schools and universities, Creating a better preventive care system	Always
26	1	Pardubice Region	2	5	1	Economic conditions (e.g., financial stress), Family relationships, Loneliness and social isolation	Rural environment	Accessibility of employment and education, Better accessibility of public transport, Opportunity to establish more social contacts	More noise and pollution, Lack of nature and quiet places, Greater anonymity and feelings of loneliness	In the countryside	5	Yes, older parental age improves children's mental health	1	I don't know	1	Improving access to professional care (more therapists, shorter waiting times), Raising awareness of mental health through campaigns, Greater support in schools and universities	Often
20	1	South Moravian Region	5	0	0	Economic conditions (e.g., financial stress), Family relationships, Loneliness and social isolation	Rural environment	Greater social and cultural opportunities (cinemas, concerts, cafés, etc.), Accessibility of employment and education, Better accessibility of public transport	Higher levels of stress and pace of life, More noise and pollution, Lack of nature and quiet places	In the countryside	5	Yes, older parental age improves children's mental health	1	Yes, a younger age (up to 21) may negatively affect her mental health	2	Improving the affordability of mental health care, Greater support in schools and universities	Occasionally
23	1	Olomouc Region	4	0	0	Economic conditions (e.g., financial stress), Family relationships, Loneliness and social isolation	Mixed (on the outskirts of a city or in small towns)	Greater social and cultural opportunities (cinemas, concerts, cafés, etc.), Accessibility of employment and education, Opportunity to establish more social contacts	Higher levels of stress and pace of life, More noise and pollution, Lack of nature and quiet places	A combination of urban and rural living	5	I don't know	2	I don't know	3	Raising awareness of mental health through campaigns, Greater support in schools and universities, Creating a better preventive care system, Addendum to question 15—my goal is to take care of my mental health myself; the contribution of family or professionals is only a welcome addition.	Often
21	1	South Moravian Region	3	0	0	Economic conditions (e.g., financial stress), Family relationships, Loneliness and social isolation	Mixed (on the outskirts of a city or in small towns)	Better accessibility of public transport	Lack of nature and quiet places, Higher crime rate	In a city	3	I don't know	3	I don't know	2		Often
21	2	Pardubice Region	3	0	0	Economic conditions (e.g., financial stress), Family relationships, Loneliness and social isolation	Mixed (on the outskirts of a city or in small towns)	Greater social and cultural opportunities (cinemas, concerts, cafés, etc.), Accessibility of employment and education, Opportunity to establish more social contacts	Higher levels of stress and pace of life, Lack of nature and quiet places, Greater competitive pressure in the job market	In the countryside	5	Yes, older parental age improves children's mental health	1	I don't know	4	Improving the affordability of mental health care, Creating a better preventive care system	Often
21	2	Hradec Králové Region	3	0	0	Economic conditions (e.g., financial stress), Family relationships, Loneliness and social isolation	Urban environment	Easier access to medical and mental health care, Greater social and cultural opportunities (cinemas, concerts, cafés, etc.), Accessibility of employment and education	Higher levels of stress and pace of life, Lack of nature and quiet places, Greater competitive pressure in the job market	I have no proference	3	No, parental age has no effect	2	No, maternal age has no effect on her mental health	2	Improving access to professional care (more therapists, shorter waiting times), Raising awareness of mental health through campaigns	Often
20	2	Moravian-Silesian Region	1	0	0	Economic conditions (e.g., financial stress), Family relationships, Loneliness and social isolation	Mixed (on the outskirts of a city or in small towns)	Access to employment and education, Greater anonymity and privacy	Higher levels of stress and pace of life, greater anonymity and feelings of loneliness	A combination of urban and rural living	3	No, parental age has no effect	1	Yes, a younger age (up to 21) may negatively affect her mental health	1	Improved access to specialist care (more therapists, shorter waiting times), improved affordability of mental health care, greater support in schools and universities	Always
20	2	South Moravian Region	5	0	2	Economic conditions (e.g., financial stress), Family relationships, Loneliness and social isolation	Urban environment	Greater social and cultural opportunities (cinemas, concerts, cafés, etc.), Accessibility of employment and education, Opportunity to establish more social contacts	Higher levels of stress and pace of life, Greater anonymity and feelings of loneliness, Greater competitive pressure in the job market	A combination of urban and rural living	4	Yes, younger parental age improves children's mental health	2	Yes, an older age (over 21) may positively affect her mental health	2	Improving access to specialist care (more therapists, shorter waiting times), creating a better preventive care system	Occasionally
24	2	Zlín Region	3	0	3	Economic conditions (e.g., financial stress), girlfriend	Urban environment	Greater social and cultural opportunities (cinemas, concerts, cafés, etc.), Accessibility of employment and education, Better accessibility of public transport, Opportunity to establish more social contacts		In a city	1	I don't know	1	I don't know	5	longer opening hours in pubs, especially for beers	Always
24	1	Moravian-Silesian Region	4	0	2	Economic conditions (e.g., financial stress), Social media and technology	Urban environment	Greater social and cultural opportunities (cinemas, concerts, cafés, etc.), better access to public transport	More noise and pollution, Lack of nature and quiet places	A combination of urban and rural living	5	Yes, older parental age improves children's mental health	2	No, maternal age has no effect on her mental health	3	Improving the affordability of mental health care, Raising awareness of mental health through campaigns, Greater support in schools and universities	Occasionally
23	2	South Moravian Region	4	5	2	Economic conditions (e.g., financial stress), Social media and technology	Urban environment	Easier access to medical and mental health care, Greater social and cultural opportunities (cinemas, concerts, cafés, etc.), Greater anonymity and privacy		In a city	3	Yes, younger parental age improves children's mental health	2	I don't know	2	Improving access to specialist care (more therapists, shorter waiting times), improving the affordability of mental health care, raising awareness of mental health through campaigns	Often
25	1	Zlín Region	3	3	1	Economic conditions (e.g., financial stress), Social media and technology, Study or work pressure	Mixed (on the outskirts of a city or in small towns)	Easier access to medical and mental health care, Greater social and cultural opportunities (cinemas, concerts, cafés, etc.), Better public transport accessibility	Higher levels of stress and pace of life, Lack of nature and quiet places, Greater anonymity and feelings of loneliness	In the countryside	5	Yes, older parental age improves children's mental health	2	Yes, a younger age (up to 21) may negatively affect her mental health	3	Improving access to specialist care (more therapists, shorter waiting times), improving the affordability of mental health care, creating a better preventive care system	Occasionally
24	2	Pardubice Region	4	0	0	Economic conditions (e.g., financial stress), Social media and technology, Study or work pressure	Urban environment	Greater social and cultural opportunities (cinemas, concerts, cafés, etc.), Accessibility of employment and education, Opportunity to establish more social contacts	Higher levels of stress and pace of life, More noise and pollution, Lack of nature and quiet places	A combination of urban and rural living	5	No, parental age has no effect	1	No, maternal age has no effect on her mental health	2	Improving access to professional care (more therapists, shorter waiting times), Raising awareness of mental health through campaigns	Occasionally
24	1	Liberecký kraj	5	0	2	Economic conditions (e.g., financial stress), Social media and technology, Study or work pressure	Urban environment	Greater social and cultural opportunities (cinemas, concerts, cafés, etc.), Accessibility of employment and education, Opportunity to establish more social contacts	Higher levels of stress and pace of life, More noise and pollution, Greater anonymity and feelings of loneliness	A combination of urban and rural living	3	I don't know	2	I don't know	3	Improving access to specialist care (more therapists, shorter waiting times), creating a better preventive care system	Often
26	2	Zlín Region	3	5	3	Economic conditions (e.g., financial stress), Social media and technology, Study or work pressure	Urban environment	Access to employment and education	Higher levels of stress and pace of life, More noise and pollution, Lack of nature and quiet places, Greater competitive pressure in the job market	In the countryside	5	No, parental age has no effect	1	No, maternal age has no effect on her mental health	1	Greater support in schools and universities, Creation of a better preventive care system	Always
19	1	Moravian-Silesian Region	4	1	2	Economic conditions (e.g., financial stress), Social media and technology, Study or work pressure	Urban environment	Greater social and cultural opportunities (cinemas, concerts, cafés, etc.), Accessibility of employment and education, Better accessibility of public transport	Higher levels of stress and pace of life, More noise and pollution, Lack of nature and quiet places	A combination of urban and rural living	4	Yes, older parental age improves children's mental health	2	Yes, a younger age (up to 21) may negatively affect her mental health	2	Improved access to specialist care (more therapists, shorter waiting times), Improved affordability of mental health care	Often
23	1	Moravian-Silesian Region	4	0	2	Economic conditions (e.g., financial stress), Social media and technology, Study or work pressure	Mixed (on the outskirts of a city or in small towns)	Easier access to medical and mental health care, Greater anonymity and privacy	More noise and pollution, Lack of nature and quiet places	A combination of urban and rural living	5	Yes, older parental age improves children's mental health	4	I don't know	1	Improved access to professional care (more therapists, shorter waiting times), Greater support in schools and universities	Always
21	2	Moravian-Silesian Region	3	0	3	Economic conditions (e.g., financial stress), Social media and technology, Study or work pressure	Urban environment	Greater social and cultural opportunities (cinemas, concerts, cafés, etc.), Accessibility of employment and education, Better accessibility of public transport		A combination of urban and rural living	4	I don't know	1	Yes, a younger age (up to 21) may negatively affect her mental health	3	Raising awareness of mental health through campaigns, Greater support in schools and universities, Creating a better preventive care system	Occasionally
26	2	Hradec Králové Region	4	0	0	Economic conditions (e.g., financial stress), Social media and technology, Study or work pressure	Mixed (on the outskirts of a city or in small towns)	Easier access to medical and mental health care, Greater social and cultural opportunities (cinemas, concerts, cafés, etc.), Accessibility of employment and education	Higher levels of stress and pace of life, More noise and pollution, Greater anonymity and feelings of loneliness	A combination of urban and rural living	5	No, parental age has no effect	2	Yes, a younger age (up to 21) may negatively affect her mental health	4	Improved access to specialist care (more therapists, shorter waiting times), greater support in schools and universities, creation of a better preventive care system.	Occasionally
21	2	Moravian-Silesian Region	4	0	0	Economic conditions (e.g., financial stress), Social media and technology, Study or work pressure	Mixed (on the outskirts of a city or in small towns)	Easier access to medical and mental health care, Greater social and cultural opportunities (cinemas, concerts, cafés, etc.), Accessibility of employment and education	More noise and pollution, Lack of nature and quiet places, Greater anonymity and feelings of loneliness	A combination of urban and rural living	4	I don't know	4	I don't know	3	Improved access to specialist care (more therapists, shorter waiting times), Improved affordability of mental health care	Often
24	1	Olomouc Region	3	0	2	Economic conditions (e.g., financial stress), Social media and technology, Study or work pressure	Urban environment	Greater social and cultural opportunities (cinemas, concerts, cafés, etc.), Accessibility of employment and education, Opportunity to establish more social contacts	Higher levels of stress and pace of life, Greater anonymity and feelings of loneliness, Greater competitive pressure in the job market	A combination of urban and rural living	2	Yes, younger parental age improves children's mental health	1	Yes, a younger age (up to 21) may negatively affect her mental health	1	Improving access to specialist care (more therapists, shorter waiting times), creating a better preventive care system	Occasionally
26	2	Moravian-Silesian Region	3	4	1	Economic conditions (e.g., financial stress), Social media and technology, Study or work pressure	Urban environment	Greater social and cultural opportunities (cinemas, concerts, cafés, etc.), Accessibility of employment and education, Better accessibility of public transport, Opportunity to establish more social contacts		A combination of urban and rural living	3	Yes, older parental age improves children's mental health	3	Yes, an older age (over 21) may positively affect her mental health	3	Improving access to professional care (more therapists, shorter waiting times), Raising awareness of mental health through campaigns, Greater support in schools and universities, Creating a better preventive care system	Often
24	2	Moravian-Silesian Region	3	4	2	Economic conditions (e.g., financial stress), Social media and technology, Study or work pressure	Mixed (on the outskirts of a city or in small towns)	Greater social and cultural opportunities (cinemas, concerts, cafés, etc.), Accessibility of employment and education, Opportunity to establish more social contacts	Higher levels of stress and pace of life, More noise and pollution, Lack of nature and quiet places	In the countryside	4	No, parental age has no effect	1	I don't know	2		Often
22	1	Moravian-Silesian Region	3	0	0	Economic conditions (e.g., financial stress), Social media and technology, Study or work pressure	Mixed (on the outskirts of a city or in small towns)	Easier access to medical and mental health care, Greater social and cultural opportunities (cinemas, concerts, cafés, etc.), Accessibility of employment and education	Higher levels of stress and pace of life, Lack of nature and quiet places, Greater competitive pressure in the job market	A combination of urban and rural living	4	Yes, younger parental age improves children's mental health	1	Yes, a younger age (up to 21) may negatively affect her mental health	1	Improved access to specialist care (more therapists, shorter waiting times), Improved affordability of mental health care	Often
19	1	Moravian-Silesian Region	3	0	0	Economic conditions (e.g., financial stress), Social media and technology, Study or work pressure	Rural environment	Easier access to medical and mental health care, Greater social and cultural opportunities (cinemas, concerts, cafés, etc.), Accessibility of employment and education, Greater anonymity and privacy, Better accessibility of public transport, Opportunity to establish more social contacts	Higher levels of stress and pace of life, Higher crime rates	A combination of urban and rural living	2	I don't know	1	I don't know	1	Greater support in schools and universities	Often

Age	Gender	Region of Permanent Residence:	Q1. How would you rate your current mental state? (1 = Very poor, 5 = Very good)	Q2. If you sought professional help for your mental health (e.g., psychologist, psychiatrist), how would you rate the quality of these services? (1 = Very dissatisfied, 5 = Very satisfied)	Q3. Do you think mental health care is easily accessible in your city/region?	Q4: Which factors do you think affect the mental health of young people aged 18–25 the most? (select the 3 most important)	Q5. Do you live in an urban or rural environment?	Q6. What benefits of living in the city do you think have a positive impact on mental health? (select the 3 most important)	Q7. What disadvantages of living in the city do you think deteriorate mental health? (select the 3 most important)	Q8. If you could choose, where would you prefer to live in terms of mental health?	Q9. How important is support from family and friends to you in dealing with mental health problems? (1 = Not important, 5 = Very important)	Q10. Do you think the age at which people enter parenthood can affect the mental health of their children?	Q11. How much of an impact do you think crime in your region has on your mental health? (1 = No effect, 5 = Very strong effect)	Q12. Do you think the age of the first-time mother can affect her mental health?	Q13.How would you rate the level of general awareness and education about mental health in secondary schools and colleges? (1 = Very poor, 5 = Very good)	Q14. Which of the following do you think would be most helpful in improving mental health care for young people in your region? (select up to 2 options)	Q15. How often do you feel you have to look after your mental health on your own, without the help of professionals or family?
21	1	Olomouc Region	3	0	0	Economic conditions (e.g., financial stress), Social media and technology, Study or work pressure	Rural environment	Easier access to medical and mental health care, Greater social and cultural opportunities (cinemas, concerts, cafés, etc.), Accessibility of employment and education	Higher levels of stress and pace of life, More noise and pollution, Lack of nature and quiet places	In the countryside	5	Yes, older parental age improves children's mental health	1	Yes, a younger age (up to 21) may negatively affect her mental health	1	Raising awareness of mental health through campaigns, Greater support in schools and universities, Creating a better preventive care system	Occasionally
20	1	Moravian-Silesian Region	2	0	0	Economic conditions (e.g., financial stress), Social media and technology, Study or work pressure	Mixed (on the outskirts of a city or in small towns)	Easier access to medical and mental health care, Accessibility of employment and education, Better accessibility of public transport	Higher levels of stress and pace of life, More noise and pollution, Lack of nature and quiet places, Higher crime rates	A combination of urban and rural living	5	Yes, younger parental age improves children's mental health	4	Yes, a younger age (up to 21) may negatively affect her mental health	1	Improving the affordability of mental health care, Raising awareness of mental health through campaigns, Greater support in schools and universities	Always
26	1	Olomouc Region	4	0	3	Economic conditions (e.g., financial stress), Social media and technology, Study or work pressure	Rural environment	Greater social and cultural opportunities (cinemas, concerts, cafés, etc.), Accessibility of employment and education, Opportunity to establish more social contacts	Higher levels of stress and pace of life, Lack of nature and quiet places, Greater anonymity and feelings of loneliness	A combination of urban and rural living	4	No, parental age has no effect	3	Yes, an older age (over 21) may positively affect her mental health	3	Raising awareness of mental health through campaigns, Creating a better preventive care system	Often
23	1	Moravian-Silesian Region	4	1	1	Economic conditions (e.g., financial stress), Social media and technology, Study or work pressure	Mixed (on the outskirts of a city or in small towns)	Greater social and cultural opportunities (cinemas, concerts, cafés, etc.), Accessibility of employment and education, Opportunity to establish more social contacts	Higher levels of stress and pace of life, More noise and pollution, Lack of nature and quiet places	A combination of urban and rural living	5	Yes, older parental age improves children's mental health	4	Yes, a younger age (up to 21) may negatively affect her mental health	1	Improved access to professional care (more therapists, shorter waiting times), Greater support in schools and universities	Always
26	2	Moravian-Silesian Region	3	0	0	Economic conditions (e.g., financial stress), Social media and technology, Study or work pressure	Urban environment	More social and cultural opportunities (cinemas, concerts, cafés, etc.), Opportunity to make more social contacts, Many grocery stores and discounts nearby.	Higher levels of stress and pace of life, More noise and pollution, Greater competitive pressure in the job market	In the countryside	5	No, parental age has no effect	1	No, maternal age has no effect on her mental health	2	Improved access to specialist care (more therapists, shorter waiting times)	Always
23	1	Moravian-Silesian Region	4	0	2	Economic conditions (e.g., financial stress), Social media and technology, Study or work pressure	Urban environment	Easier access to medical and mental health care, Greater social and cultural opportunities (cinemas, concerts, cafés, etc.), Accessibility of employment and education	Higher levels of stress and pace of life, More noise and pollution, Lack of nature and quiet places	A combination of urban and rural living	4	Yes, older parental age improves children's mental health	2	Yes, a younger age (up to 21) may negatively affect her mental health	3		Occasionally
23	2	Moravian-Silesian Region	3	4	2	Economic conditions (e.g., financial stress), Social media and technology, Study or work pressure	Urban environment	Greater social and cultural opportunities (cinemas, concerts, cafés, etc.), Accessibility of employment and education, Opportunity to establish more social contacts	Higher levels of stress and pace of life, More noise and pollution, Greater competitive pressure in the job market	In a city	4	I don't know	2	I don't know	1	Improving the affordability of mental health care, Creating a better preventive care system	Often
22	2	Moravian-Silesian Region	3	0	2	Economic conditions (e.g., financial stress), Social media and technology, Study or work pressure	Urban environment	Greater social and cultural opportunities (cinemas, concerts, cafés, etc.), better access to public transport, opportunity to make more social contacts	Greater competitive pressure in the labor market	In a city	4	I don't know	3	Yes, a younger age (up to 21) may negatively affect her mental health	2	Raising awareness of mental health through campaigns, Greater support in schools and universities, Creating a better preventive care system	Often
23	1	Moravian-Silesian Region	3	0	3	Economic conditions (e.g., financial stress), Social media and technology, Study or work pressure	Urban environment	Easier access to medical and mental health care, Greater social and cultural opportunities (cinemas, concerts, cafés, etc.), Accessibility of employment and education	Higher levels of stress and pace of life, Lack of nature and quiet places, Greater anonymity and feelings of loneliness	In the countryside	2	Yes, older parental age improves children's mental health	1	Yes, a younger age (up to 21) may negatively affect her mental health	2	Improving the affordability of mental health care, Greater support in schools and universities	Always
23	1	Moravian-Silesian Region	3	0	0	Economic conditions (e.g., financial stress), Social media and technology, Study or work pressure	Mixed (on the outskirts of a city or in small towns)	Greater social and cultural opportunities (cinemas, concerts, cafés, etc.), Accessibility of employment and education, Better accessibility of public transport	Higher levels of stress and pace of life, More noise and pollution, Greater competitive pressure in the job market	A combination of urban and rural living	4	Yes, older parental age improves children's mental health	3	I don't know	1	Raising awareness of mental health through campaigns, Creating a better preventive care system	Always
23	1	Moravian-Silesian Region	4	0	2	Economic conditions (e.g., financial stress), Social media and technology, Study or work pressure	Urban environment	Easier access to medical and mental health care, Greater social and cultural opportunities (cinemas, concerts, cafés, etc.), Accessibility of employment and education	Higher levels of stress and pace of life, Lack of nature and quiet places, Greater competitive pressure in the job market	In the countryside	5	No, parental age has no effect	3	Yes, an older age (over 21) may positively affect her mental health	1	Improved access to professional care (more therapists, shorter waiting times), Greater support in schools and universities	Often
23	1	Moravian-Silesian Region	2	4	2	Economic conditions (e.g., financial stress), Social media and technology, Study or work pressure	Mixed (on the outskirts of a city or in small towns)	Greater social and cultural opportunities (cinemas, concerts, cafés, etc.), Accessibility of employment and education, Better accessibility of public transport	Higher levels of stress and pace of life, More noise and pollution, Lack of nature and quiet places, Higher crime rates	In the countryside	4	No, parental age has no effect	3	No, maternal age has no effect on her mental health	2	Improved access to professional care (more therapists, shorter waiting times), Greater support in schools and universities	Often
22	2	Moravian-Silesian Region	4	3	2	Economic conditions (e.g., financial stress), Social media and technology, Study or work pressure	Rural environment	Greater social and cultural opportunities (cinemas, concerts, cafés, etc.), Accessibility of employment and education, Opportunity to establish more social contacts	Higher levels of stress and pace of life, Greater anonymity and feelings of loneliness, Greater competitive pressure in the job market	In the countryside	4	No, parental age has no effect	3	Yes, a younger age (up to 21) may negatively affect her mental health	3	Raising awareness of mental health through campaigns, Greater support in schools and universities	Always
24	2	Moravian-Silesian Region	2	3	3	Economic conditions (e.g., financial stress), Social media and technology, Study or work pressure	Urban environment	Accessibility of employment and education, Greater anonymity and privacy, Opportunity to establish more social contacts		A combination of urban and rural living	2	Yes, younger parental age improves children's mental health	3	Yes, an older age (over 21) may positively affect her mental health	2	Improving the affordability of mental health care, Creating a better preventive care system	Always
26	1	Moravian-Silesian Region	3	0	0	Economic conditions (e.g., financial stress), Social media and technology, Study or work pressure	Urban environment	Greater social and cultural opportunities (cinemas, concerts, cafés, etc.), Accessibility of employment and education, Better accessibility of public transport	More noise and pollution, higher crime rates	In the countryside	5	No, parental age has no effect	3	Yes, a younger age (up to 21) may negatively affect her mental health	3	Raising awareness of mental health through campaigns, Greater support in schools and universities, Creating a better preventive care system	Often
22	2	Moravian-Silesian Region	3	0	0	Economic conditions (e.g., financial stress), Social media and technology, Study or work pressure	Rural environment	Greater social and cultural opportunities (cinemas, concerts, cafés, etc.), Opportunity to establish more social contacts		In the countryside	5	Yes, older parental age improves children's mental health	4	Yes, a younger age (up to 21) may negatively affect her mental health	2	Improved access to professional care (more therapists, shorter waiting times), Greater support in schools and universities	Often
21	1	Moravian-Silesian Region	3	0	1	Economic conditions (e.g., financial stress), Social media and technology, Study or work pressure	Mixed (on the outskirts of a city or in small towns)	Greater social and cultural opportunities (cinemas, concerts, cafés, etc.), Accessibility of employment and education, Better accessibility of public transport		A combination of urban and rural living	4	No, parental age has no effect	3	Yes, an older age (over 21) may positively affect her mental health	3	Raising awareness of mental health through campaigns, Greater support in schools and universities	Occasionally
21	1	Moravian-Silesian Region	4	0	0	Economic conditions (e.g., financial stress), Social media and technology, Study or work pressure	Mixed (on the outskirts of a city or in small towns)	Greater social and cultural opportunities (cinemas, concerts, cafés, etc.), Accessibility of employment and education, Opportunity to establish more social contacts	Higher levels of stress and pace of life, Lack of nature and quiet places, Higher crime rates	A combination of urban and rural living	5	Yes, older parental age improves children's mental health	2	Yes, a younger age (up to 21) may negatively affect her mental health	3	Greater support in schools and universities, Creation of a better preventive care system	Occasionally
19	2	Pardubice Region	5	0	0	Economic conditions (e.g., financial stress), Social media and technology, Study or work pressure	Mixed (on the outskirts of a city or in small towns)	Greater social and cultural opportunities (cinemas, concerts, cafés, etc.), Accessibility of employment and education, Greater anonymity and privacy		A combination of urban and rural living	5	Yes, older parental age improves children's mental health	2	Yes, a younger age (up to 21) may negatively affect her mental health	3	Raising awareness of mental health through campaigns, Greater support in schools and universities	Occasionally
20	2	Moravian-Silesian Region	3	0	0	Economic conditions (e.g., financial stress), Social media and technology, Study or work pressure	Mixed (on the outskirts of a city or in small towns)	Greater social and cultural opportunities (cinemas, concerts, cafés, etc.), Opportunity to establish more social contacts	Higher levels of stress and pace of life, More noise and pollution	A combination of urban and rural living	5	I don't know	3	I don't know	3	Raising awareness of mental health through campaigns, Greater support in schools and universities, Creating a better preventive care system	Often
20	1	Olomouc Region	3	0	1	Economic conditions (e.g., financial stress), Social media and technology, Study or work pressure	Mixed (on the outskirts of a city or in small towns)	Greater social and cultural opportunities (cinemas, concerts, cafés, etc.), greater anonymity and privacy	More noise and pollution, Lack of nature and quiet places, Higher crime rate	In the countryside	5	No, parental age has no effect	3	Yes, a younger age (up to 21) may negatively affect her mental health	2	Improved access to specialist care (more therapists, shorter waiting times), Improved affordability of mental health care	Often
22	1	Zlín Region	3	0	2	Economic conditions (e.g., financial stress), Social media and technology, Study or work pressure	Mixed (on the outskirts of a city or in small towns)		More noise and pollution, Lack of nature and quiet places, Higher crime rate	In the countryside	5	I don't know	2	Yes, a younger age (up to 21) may negatively affect her mental health	2	Improving access to specialist care (more therapists, shorter waiting times), improving the affordability of mental health care, raising awareness of mental health through campaigns, greater support in schools and universities	Occasionally
21	1	Moravian-Silesian Region	2	0	1	Economic conditions (e.g., financial stress), Social media and technology, Study or work pressure	Urban environment	Easier access to medical and mental health care, Greater social and cultural opportunities (cinemas, concerts, cafés, etc.), Better public transport accessibility	More noise and pollution, Greater anonymity and feelings of loneliness, Greater competitive pressure in the job market	A combination of urban and rural living	5	No, parental age has no effect	2	Yes, a younger age (up to 21) may negatively affect her mental health	1	Improved access to professional care (more therapists, shorter waiting times), Greater support in schools and universities	Always
20	1	Moravian-Silesian Region	2	0	0	Economic conditions (e.g., financial stress), Social media and technology, Study or work pressure	Urban environment	Greater social and cultural opportunities (cinemas, concerts, cafés, etc.), Accessibility of employment and education, Opportunity to establish more social contacts		A combination of urban and rural living	5	Yes, older parental age improves children's mental health	2	Yes, a younger age (up to 21) may negatively affect her mental health	2	Improved access to specialist care (more therapists, shorter waiting times), Improved affordability of mental health care	Often
21	1	Moravian-Silesian Region	3	0	1	Economic conditions (e.g., financial stress), Social media and technology, Study or work pressure	Urban environment	Easier access to medical and mental health care, Greater social and cultural opportunities (cinemas, concerts, cafés, etc.), Greater anonymity and privacy	Higher levels of stress and pace of life, Lack of nature and quiet places, High concentration of people associated with the evaluation of others (pressure to conform to norms)	A combination of urban and rural living	4	I don't know	4	I don't know	2	Improving access to specialist care (more therapists, shorter waiting times), creating a better preventive care system	Often
24	1	Moravian-Silesian Region	4	0	0	Economic conditions (e.g., financial stress), Social media and technology, Study or work pressure	Urban environment	Greater social and cultural opportunities (cinemas, concerts, cafés, etc.), greater anonymity and privacy	Higher levels of stress and pace of life, More noise and pollution, Lack of nature and quiet places	A combination of urban and rural living	5	Yes, older parental age improves children's mental health	2	Yes, a younger age (up to 21) may negatively affect her mental health	1	Improved access to specialist care (more therapists, shorter waiting times), improved affordability of mental health care, greater support in schools and universities	Never
25	2	Moravian-Silesian Region	4	0	0	Economic conditions (e.g., financial stress), Social media and technology, Study or work pressure	Mixed (on the outskirts of a city or in small towns)		Higher levels of stress and pace of life, More noise and pollution, Lack of nature and quiet places	A combination of urban and rural living	5	I don't know	2	I don't know	2	Raising awareness of mental health through campaigns, Creating a better preventive care system	Never
21	1	Pardubice Region	3	2	2	Economic conditions (e.g., financial stress), Social media and technology, Study or work pressure	Rural environment	Greater social and cultural opportunities (cinemas, concerts, cafés, etc.), Accessibility of employment and education, Opportunity to establish more social contacts	Higher levels of stress and pace of life, More noise and pollution, Higher crime rates	A combination of urban and rural living	4	Yes, older parental age improves children's mental health	3	Yes, a younger age (up to 21) may negatively affect her mental health	4	Improving access to specialist care (more therapists, shorter waiting times), creating a better preventive care system	Often
21	1	South Moravian Region	4	0	0	Economic conditions (e.g., financial stress), Social media and technology, Study or work pressure	Rural environment	Greater social and cultural opportunities (cinemas, concerts, cafés, etc.), Accessibility of employment and education, Better accessibility of public transport	More noise and pollution, Lack of nature and quiet places, Greater anonymity and feelings of loneliness, Higher crime rates	In the countryside	3	No, parental age has no effect	4	I don't know	2	Improved access to specialist care (more therapists, shorter waiting times), greater support in schools and universities, creation of a better preventive care system.	Occasionally
21	1	Vysočina Region	2	3	2	Economic conditions (e.g., financial stress), Social media and technology, Study or work pressure	Urban environment	Easier access to medical and mental health care, Greater social and cultural opportunities (cinemas, concerts, cafés, etc.), Accessibility of employment and education	Higher levels of stress and pace of life, Lack of nature and quiet places, Higher crime rates	A combination of urban and rural living	5	No, parental age has no effect	3	No, maternal age has no effect on her mental health	3	Improving the affordability of mental health care, Greater support in schools and universities	Often
20	1	Moravian-Silesian Region	2	0	2	Economic conditions (e.g., financial stress), Social media and technology, Study or work pressure	Mixed (on the outskirts of a city or in small towns)	Greater social and cultural opportunities (cinemas, concerts, cafés, etc.), Accessibility of employment and education, Opportunity to establish more social contacts		A combination of urban and rural living	1	Yes, younger parental age improves children's mental health	4	I don't know	1	Raising awareness of mental health through campaigns, Greater support in schools and universities	Occasionally
20	1	South Moravian Region	5	0	0	Economic conditions (e.g., financial stress), Social media and technology, Study or work pressure	Mixed (on the outskirts of a city or in small towns)	Easier access to medical and mental health care, Greater social and cultural opportunities (cinemas, concerts, cafés, etc.), Accessibility of employment and education	Higher levels of stress and pace of life, More noise and pollution, Greater competitive pressure in the job market	A combination of urban and rural living	5	I don't know	2	Yes, a younger age (up to 21) may negatively affect her mental health	3	Improving the affordability of mental health care, Greater support in schools and universities	Never
19	1	South Moravian Region	4	5	1	Economic conditions (e.g., financial stress), Social media and technology, Study or work pressure	Urban environment	Easier access to medical and mental health care, Greater social and cultural opportunities (cinemas, concerts, cafés, etc.), Opportunity to establish more social contacts	Higher levels of stress and pace of life, Lack of nature and quiet places, Greater anonymity and feelings of loneliness	A combination of urban and rural living	5	No, parental age has no effect	3	Yes, a younger age (up to 21) may negatively affect her mental health	4	Improved access to specialist care (more therapists, shorter waiting times), Improved affordability of mental health care	Never
22	1	Hradec Králové Region	3	0	2	Economic conditions (e.g., financial stress), Social media and technology, Study or work pressure	Urban environment	Greater social and cultural opportunities (cinemas, concerts, cafés, etc.), Accessibility of employment and education, Greater anonymity and privacy	Higher levels of stress and pace of life, More noise and pollution, Lack of nature and quiet places	A combination of urban and rural living	5	Yes, older parental age improves children's mental health	4	Yes, an older age (over 21) may positively affect her mental health	4	Raising awareness of mental health through campaigns, Greater support in schools and universities	Always
24	2	Olomouc Region	2	5	1	Economic conditions (e.g., financial stress), Social media and technology, Study or work pressure	Urban environment	Greater social and cultural opportunities (cinemas, concerts, cafés, etc.), better access to public transport	Higher levels of stress and pace of life, Lack of nature and quiet places, Higher crime rates	In the countryside	4	Yes, older parental age improves children's mental health	3	Yes, a younger age (up to 21) may negatively affect her mental health	3	Improved access to specialist care (more therapists, shorter waiting times)	Often
21	2	South Moravian Region	5	0	2	Economic conditions (e.g., financial stress), Social media and technology, Study or work pressure	Urban environment	Easier access to medical and mental health care, Greater social and cultural opportunities (cinemas, concerts, cafés, etc.), Opportunity to establish more social contacts	Higher levels of stress and pace of life, More noise and pollution, Lack of nature and quiet places	A combination of urban and rural living	5	Yes, older parental age improves children's mental health	4	Yes, a younger age (up to 21) may negatively affect her mental health	3		Often
22	1	South Moravian Region	3	5	2	Economic conditions (e.g., financial stress), Social media and technology, Study or work pressure	Urban environment	Easier access to medical and mental health care, Accessibility of employment and education, Opportunity to establish more social contacts	Higher levels of stress and pace of life, Lack of nature and quiet places, Greater competitive pressure in the job market	A combination of urban and rural living	5	I don't know	1	I don't know	3	Improving access to specialist care (more therapists, shorter waiting times), creating a better preventive care system	Often
21	1	South Moravian Region	4	0	3	Economic conditions (e.g., financial stress), Social media and technology, Study or work pressure	Rural environment		Higher levels of stress and pace of life, More noise and pollution, Lack of nature and quiet places	In the countryside	5	Yes, younger parental age improves children's mental health	3	No, maternal age has no effect on her mental health	3	Raising awareness of mental health through campaigns, Creating a better preventive care system	Occasionally
20	1	South Moravian Region	4	0	2	Economic conditions (e.g., financial stress), Social media and technology, Study or work pressure	Urban environment	Easier access to medical and mental health care, Greater social and cultural opportunities (cinemas, concerts, cafés, etc.), Opportunity to establish more social contacts	Higher levels of stress and pace of life, More noise and pollution, Lack of nature and quiet places	A combination of urban and rural living	5	Yes, older parental age improves children's mental health	1	Yes, a younger age (up to 21) may negatively affect her mental health	3	Greater support in schools and universities, Creation of a better preventive care system	Occasionally
20	2	Moravian-Silesian Region	4	0	2	Economic conditions (e.g., financial stress), Social media and technology, Study or work pressure	Urban environment	Easier access to medical and mental health care, Greater social and cultural opportunities (cinemas, concerts, cafés, etc.), Greater anonymity and privacy	Higher levels of stress and pace of life, Greater anonymity and feelings of loneliness, Greater competitive pressure in the job market	A combination of urban and rural living	5	Yes, younger parental age improves children's mental health	1	No, maternal age has no effect on her mental health	2	Raising awareness of mental health through campaigns, Creating a better preventive care system	Always
20	1	South Moravian Region	4	2	1	Economic conditions (e.g., financial stress), Social media and technology, Study or work pressure	Urban environment	Greater social and cultural opportunities (cinemas, concerts, cafés, etc.), Accessibility of employment and education, Better accessibility of public transport		In the countryside	2	I don't know	3	Yes, a younger age (up to 21) may negatively affect her mental health	5	Improved access to specialist care (more therapists, shorter waiting times), Improved affordability of mental health care	Always
21	1	Capital City of Prague	4	2	2	Economic conditions (e.g., financial stress), Social media and technology, Study or work pressure	Urban environment	Easier access to medical and mental health care, Accessibility of employment and education, Better accessibility of public transport	Higher levels of stress and pace of life, More noise and pollution, Lack of nature and quiet places	In the countryside	4	No, parental age has no effect	2	I don't know	3	Improving access to specialist care (more therapists, shorter waiting times), creating a better preventive care system	Often
21	2	Central Bohemian Region	2	0	0	Economic conditions (e.g., financial stress), Social media and technology, Study or work pressure	Urban environment	Greater social and cultural opportunities (cinemas, concerts, cafés, etc.), Greater anonymity and privacy, Opportunity to establish more social contacts	Higher levels of stress and pace of life, Lack of nature and quiet places, Greater competitive pressure in the job market	In a city	3	Yes, younger parental age improves children's mental health	2	I don't know	3	Improved access to specialist care (more therapists, shorter waiting times), Improved affordability of mental health care	Always
22	1	South Moravian Region	4	0	0	Economic conditions (e.g., financial stress), Social media and technology, Study or work pressure	Urban environment	Easier access to medical and mental health care, Better public transport accessibility, Opportunity to establish more social contacts	Higher levels of stress and pace of life, More noise and pollution, Greater competitive pressure in the job market	In a city	5	I don't know	1	Yes, a younger age (up to 21) may negatively affect her mental health	1	Raising awareness of mental health through campaigns, Greater support in schools and universities, Creating a better preventive care system	Never
21	1	Moravian-Silesian Region	3	0	2	Economic conditions (e.g., financial stress), Social media and technology, Study or work pressure	Rural environment	Greater social and cultural opportunities (cinemas, concerts, cafés, etc.), better access to public transport	Higher levels of stress and pace of life, More noise and pollution, Lack of nature and quiet places	In the countryside	5	No, parental age has no effect	3	I don't know	2	Raising awareness of mental health through campaigns, Greater support in schools and universities	Often
22	1	Moravian-Silesian Region	4	4	2	Economic conditions (e.g., financial stress), Social media and technology, Study or work pressure	Mixed (on the outskirts of a city or in small towns)	Greater social and cultural opportunities (cinemas, concerts, cafés, etc.), better access to public transport, opportunity to make more social contacts	Higher levels of stress and pace of life, Lack of nature and quiet places, Greater competitive pressure in the job market	A combination of urban and rural living	5	No, parental age has no effect	2	No, maternal age has no effect on her mental health	2	Improved access to specialist care (more therapists, shorter waiting times), greater support in schools and universities, creation of a better preventive care system.	Often
20	1	Moravian-Silesian Region	4	0	1	Economic conditions (e.g., financial stress), Social media and technology, Study or work pressure	Rural environment	Greater social and cultural opportunities (cinemas, concerts, cafés, etc.), Accessibility of employment and education, Opportunity to establish more social contacts	Higher levels of stress and pace of life, More noise and pollution, Lack of nature and quiet places	In the countryside	5	Yes, younger parental age improves children's mental health	2	Yes, a younger age (up to 21) may negatively affect her mental health	2	Improving the affordability of mental health care, Greater support in schools and universities	Often
20	2	South Moravian Region	3	0	0	Economic conditions (e.g., financial stress), Social media and technology, Study or work pressure	Urban environment	Greater social and cultural opportunities (cinemas, concerts, cafés, etc.), Accessibility of employment and education, Opportunity to establish more social contacts	Higher levels of stress and pace of life, Lack of nature and quiet places, Greater competitive pressure in the job market	I have no proference	5	Yes, older parental age improves children's mental health	2	Yes, a younger age (up to 21) may negatively affect her mental health	2	Raising awareness of mental health through campaigns, Greater support in schools and universities	Occasionally

Age	Gender	Region of Permanent Residence:	Q1. How would you rate your current mental state? (1 = Very poor, 5 = Very good)	Q2. If you sought professional help for your mental health (e.g., psychologist, psychiatrist), how would you rate the quality of these services? (1 = Very dissatisfied, 5 = Very satisfied)	Q3. Do you think mental health care is easily accessible in your city/region?	Q4: Which factors do you think affect the mental health of young people aged 18–25 the most? (select the 3 most important)	Q5. Do you live in an urban or rural environment?	Q6. What benefits of living in the city do you think have a positive impact on mental health? (select the 3 most important)	Q7. What disadvantages of living in the city do you think deteriorate mental health? (select the 3 most important)	Q8. If you could choose, where would you prefer to live in terms of mental health?	Q9. How important is support from family and friends to you in dealing with mental health problems? (1 = Not important, 5 = Very important)	Q10. Do you think the age at which people enter parenthood can affect the mental health of their children?	Q11. How much of an impact do you think crime in your region has on your mental health? (1 = No effect, 5 = Very strong effect)	Q12. Do you think the age of the first-time mother can affect her mental health?	Q13.How would you rate the level of general awareness and education about mental health in secondary schools and colleges? (1 = Very poor, 5 = Very good)	Q14. Which of the following do you think would be most helpful in improving mental health care for young people in your region? (select up to 2 options)	Q15. How often do you feel you have to look after your mental health on your own, without the help of professionals or family?
22	1	Moravian-Silesian Region	3	0	0	Economic conditions (e.g., financial stress), Social media and technology, Study or work pressure	Mixed (on the outskirts of a city or in small towns)	Greater social and cultural opportunities (cinemas, concerts, cafés, etc.), Accessibility of employment and education, Opportunity to establish more social contacts	Higher levels of stress and pace of life, More noise and pollution, Lack of nature and quiet places	A combination of urban and rural living	5	I don't know	2	Yes, an older age (over 21) may positively affect her mental health	2	Improved access to professional care (more therapists, shorter waiting times), Greater support in schools and universities	Always
21	1	South Moravian Region	2	4	2	Economic conditions (e.g., financial stress), Social media and technology, Study or work pressure, Crime and feeling of danger	Mixed (on the outskirts of a city or in small towns)	Greater social and cultural opportunities (cinemas, concerts, cafés, etc.), Accessibility of employment and education, Better accessibility of public transport	More noise and pollution, Greater anonymity and feelings of loneliness, Higher crime rates	In the countryside	4	I don't know	5	Yes, a younger age (up to 21) may negatively affect her mental health	3	Improving access to specialist care (more therapists, shorter waiting times), improving the affordability of mental health care, creating a better preventive care system	Often
23	1	Moravian-Silesian Region	3	0	1	Economic conditions (e.g., financial stress), Social media and technology, Study or work pressure, Insufficient access to healthcare	Urban environment	Easier access to medical and mental health care, Greater social and cultural opportunities (cinemas, concerts, cafés, etc.), Accessibility of employment and education, Better accessibility of public transport	Higher levels of stress and pace of life, More noise and pollution, Lack of nature and quiet places, Higher crime rates, Greater competitive pressure in the job market	A combination of urban and rural living	5	No, parental age has no effect	2	No, maternal age has no effect on her mental health	2	Improving access to specialist care (more therapists, shorter waiting times), improving the affordability of mental health care, raising awareness of mental health through campaigns, greater support in schools and universities, creating a better preventive care system.	Often
22	1	Pardubice Region	3	3	2	Economic conditions (e.g., financial stress), Social media and technology, Study or work pressure, Insufficient access to healthcare	Mixed (on the outskirts of a city or in small towns)	Easier access to medical and mental health care, Greater social and cultural opportunities (cinemas, concerts, cafés, etc.), Accessibility of employment and education, Better accessibility of public transport, Opportunity to establish more social contacts	Higher levels of stress and pace of life, More noise and pollution, Lack of nature and quiet places, Greater competitive pressure in the job market	A combination of urban and rural living	4	I don't know	1	Yes, a younger age (up to 21) may negatively affect her mental health	3	Improving access to specialist care (more therapists, shorter waiting times), improving the affordability of mental health care, raising awareness of mental health through campaigns, greater support in schools and universities, creating a better preventive care system.	Occasionally
22	1	Zlín Region	3	0	2	Economic conditions (e.g., financial stress), Social media and technology, Studijní nebo pracovní tlak, Osamělost a sociální izolace	Urban environment	Greater social and cultural opportunities (cinemas, concerts, cafés, etc.), Accessibility of employment and education, Opportunity to establish more social contacts	Higher levels of stress and pace of life, Lack of nature and quiet places, Greater competitive pressure in the job market	A combination of urban and rural living	5	Yes, younger parental age improves children's mental health	1	Yes, a younger age (up to 21) may negatively affect her mental health	1	Raising awareness of mental health through campaigns, Greater support in schools and universities	Always
25	1	Central Bohemian Region	4	3	1	Economic conditions (e.g., financial stress), Social media and technology, Studijní nebo pracovní tlak, Osamělost a sociální izolace	Urban environment	Greater social and cultural opportunities (cinemas, concerts, cafés, etc.), Accessibility of employment and education, Greater anonymity and privacy	Higher levels of stress and pace of life, More noise and pollution, Lack of nature and quiet places, Higher crime rates, Greater competitive pressure in the job market	A combination of urban and rural living	5	I don't know	2	Yes, a younger age (up to 21) may negatively affect her mental health	3	Improved access to specialist care (more therapists, shorter waiting times), improved affordability of mental health care, greater support in schools and universities, creation of a better preventive care system.	Occasionally
22	1	Olomouc Region	2	0	0	Economic conditions (e.g., financial stress), Social media and technology, Studijní nebo pracovní tlak, Osamělost a sociální izolace	Urban environment	Greater social and cultural opportunities (cinemas, concerts, cafés, etc.), Accessibility of employment and education, Better accessibility of public transport	More noise and pollution, Lack of nature and quiet places	A combination of urban and rural living	4	No, parental age has no effect	2	No, maternal age has no effect on her mental health	1	Improving access to specialist care (more therapists, shorter waiting times), improving the affordability of mental health care, raising awareness of mental health through campaigns, greater support in schools and universities	Often
24	1	Moravian-Silesian Region	3	0	0	Economic conditions (e.g., financial stress), Social media and technology, Studijní nebo pracovní tlak, Osamělost a sociální izolace	Urban environment	Accessibility of employment and education, Better accessibility of public transport		A combination of urban and rural living	4	No, parental age has no effect	2	No, maternal age has no effect on her mental health	1	Improving the affordability of mental health care, Raising awareness of mental health through campaigns, Creating a better preventive care system	Occasionally
22	1	Moravian-Silesian Region	4	2	2	Economic conditions (e.g., financial stress), Social media and technology, Studijní nebo pracovní tlak, Osamělost a sociální izolace	Urban environment	Greater social and cultural opportunities (cinemas, concerts, cafés, etc.), Accessibility of employment and education, Better accessibility of public transport	More noise and pollution, Lack of nature and quiet places, Higher crime rate	A combination of urban and rural living	4	Yes, older parental age improves children's mental health	2	Yes, a younger age (up to 21) may negatively affect her mental health	2	Greater support in schools and universities, Creation of a better preventive care system	Occasionally
24	1	Karlovy Vary Region	4	0	2	Economic conditions (e.g., financial stress), Social media and technology, Studijní nebo pracovní tlak, Osamělost a sociální izolace	Urban environment	Accessibility of employment and education, Greater anonymity and privacy, Opportunity to establish more social contacts	Higher levels of stress and pace of life, More noise and pollution, Higher crime rates	In the countryside	5	No, parental age has no effect	4	No, maternal age has no effect on her mental health	2	Improving the affordability of mental health care, Greater support in schools and universities	Often
24	2	Moravian-Silesian Region	4	3	1	Economic conditions (e.g., financial stress), Social media and technology, Studijní nebo pracovní tlak, Osamělost a sociální izolace	Rural environment	Greater social and cultural opportunities (cinemas, concerts, cafés, etc.), Accessibility of employment and education, Opportunity to establish more social contacts	Higher levels of stress and pace of life, Lack of nature and quiet places, Greater anonymity and feelings of loneliness	In the countryside	5	I don't know	2	Yes, a younger age (up to 21) may negatively affect her mental health	3	Improving access to specialist care (more therapists, shorter waiting times), creating a better preventive care system	Occasionally
24	2	Moravian-Silesian Region	4	0	0	Economic conditions (e.g., financial stress), Social media and technology, Studijní nebo pracovní tlak, Osamělost a sociální izolace	Urban environment	Greater social and cultural opportunities (cinemas, concerts, cafés, etc.), Accessibility of employment and education, Opportunity to establish more social contacts	Higher levels of stress and pace of life, More noise and pollution, Greater anonymity and feelings of loneliness	A combination of urban and rural living	5	Yes, older parental age improves children's mental health	2	Yes, a younger age (up to 21) may negatively affect her mental health	3	Raising awareness of mental health through campaigns	Always
20	1	Moravian-Silesian Region	1	4	1	Economic conditions (e.g., financial stress), Social media and technology, Studijní nebo pracovní tlak, Osamělost a sociální izolace	Mixed (on the outskirts of a city or in small towns)	Easier access to medical and mental health care, Greater social and cultural opportunities (cinemas, concerts, cafés, etc.), Accessibility of employment and education	Higher levels of stress and pace of life, Lack of nature and quiet places, Greater anonymity and feelings of loneliness	In a city	5	I don't know	1	Yes, a younger age (up to 21) may negatively affect her mental health	1	Improved access to professional care (more therapists, shorter waiting times), Greater support in schools and universities	Always
22	2	Moravian-Silesian Region	4	0	2	Economic conditions (e.g., financial stress), Social media and technology, Studijní nebo pracovní tlak, Osamělost a sociální izolace	Rural environment	Accessibility of employment and education, Better accessibility of public transport, Opportunity to establish more social contacts	Higher levels of stress and pace of life, More noise and pollution, Lack of nature and quiet places	In the countryside	5	I don't know	3	I don't know	2	Raising awareness of mental health through campaigns, Creating a better preventive care system	Occasionally
19	1	Moravian-Silesian Region	4	0	1	Economic conditions (e.g., financial stress), Social media and technology, Studijní nebo pracovní tlak, Osamělost a sociální izolace	Mixed (on the outskirts of a city or in small towns)	Greater social and cultural opportunities (cinemas, concerts, cafés, etc.), Greater anonymity and privacy, Opportunity to establish more social contacts	Higher levels of stress and pace of life, Lack of nature and quiet places, Greater competitive pressure in the job market	I have no proference	4	I don't know	1	I don't know	3	Improving access to specialist care (more therapists, shorter waiting times), improving the affordability of mental health care, raising awareness of mental health through campaigns, greater support in schools and universities, creating a better preventive care system.	Often
24	2	Olomouc Region	2	0	2	Economic conditions (e.g., financial stress), Social media and technology, Studijní nebo pracovní tlak, Osamělost a sociální izolace	Urban environment	Opportunity to establish more social contacts	Higher levels of stress and pace of life, More noise and pollution, Lack of nature and quiet places, Greater anonymity and feelings of loneliness, Higher crime rates, Greater competitive pressure in the job market	A combination of urban and rural living	5	I don't know	1	I don't know	1	Raising awareness of mental health through campaigns, Greater support in schools and universities, Creating a better preventive care system	Always
22	1	Vysočina Region	3	4	2	Economic conditions (e.g., financial stress), Social media and technology, Studijní nebo pracovní tlak, Osamělost a sociální izolace	Mixed (on the outskirts of a city or in small towns)	Greater social and cultural opportunities (cinemas, concerts, cafés, etc.), Accessibility of employment and education, Better accessibility of public transport		A combination of urban and rural living	3	Yes, younger parental age improves children's mental health	1	Yes, an older age (over 21) may positively affect her mental health	3	Improving the affordability of mental health care, Greater support in schools and universities, Creating a better preventive care system	Often
21	2	Moravian-Silesian Region	4	0	0	Economic conditions (e.g., financial stress), Social media and technology, Studijní nebo pracovní tlak, Osamělost a sociální izolace	Urban environment	Easier access to medical and mental health care, Greater social and cultural opportunities (cinemas, concerts, cafés, etc.), Accessibility of employment and education	Higher levels of stress and pace of life, Lack of nature and quiet places, Greater competitive pressure in the job market	A combination of urban and rural living	5	I don't know	3	I don't know	2	Greater support in schools and universities, Creation of a better preventive care system	Occasionally
21	1	South Moravian Region	3	5	1	Economic conditions (e.g., financial stress), Social media and technology, Studijní nebo pracovní tlak, Osamělost a sociální izolace, Nedostatečná dostupnost zdravotní péče	Urban environment	Greater social and cultural opportunities (cinemas, concerts, cafés, etc.), Accessibility of employment and education, Better accessibility of public transport	Higher levels of stress and pace of life, Lack of nature and quiet places	A combination of urban and rural living	5	I don't know	3	I don't know	3	Improved access to professional care (more therapists, shorter waiting times), Greater support in schools and universities	Always
22	1	Moravian-Silesian Region	3	0	1	Economic conditions (e.g., financial stress), Social media and technology, Studijní nebo pracovní tlak, Osamělost a sociální izolace, Závislosti	Mixed (on the outskirts of a city or in small towns)	Greater anonymity and privacy	Higher levels of stress and pace of life, More noise and pollution, Greater anonymity and feelings of loneliness	A combination of urban and rural living	5	I don't know	2	No, maternal age has no effect on her mental health	3	Improved access to specialist care (more therapists, shorter waiting times), Improved affordability of mental health care	Always
20	1	Zlín Region	4	4	2	Economic conditions (e.g., financial stress), Social media and technology, Studijní nebo pracovní tlak, Rodinné vztahy	Mixed (on the outskirts of a city or in small towns)	Greater social and cultural opportunities (cinemas, concerts, cafés, etc.), Accessibility of employment and education	More noise and pollution, Lack of nature and quiet places	In the countryside	5	No, parental age has no effect	4	I don't know	2	Improved access to professional care (more therapists, shorter waiting times), Greater support in schools and universities	Occasionally
22	1	Ústí nad Labem Region	3	0	0	Economic conditions (e.g., financial stress), Social media and technology, Studijní nebo pracovní tlak, Rodinné vztahy	Urban environment	Greater social and cultural opportunities (cinemas, concerts, cafés, etc.), Opportunity to establish more social contacts		A combination of urban and rural living	5	Yes, older parental age improves children's mental health	3	Yes, a younger age (up to 21) may negatively affect her mental health	1	Improved access to specialist care (more therapists, shorter waiting times), improved affordability of mental health care, greater support in schools and universities	Always
22	1	Moravian-Silesian Region	4	0	2	Economic conditions (e.g., financial stress), Social media and technology, Studijní nebo pracovní tlak, Rodinné vztahy	Mixed (on the outskirts of a city or in small towns)	Easier access to medical and mental health care, Greater social and cultural opportunities (cinemas, concerts, cafés, etc.), Accessibility of employment and education	More noise and pollution, Lack of nature and quiet places	A combination of urban and rural living	5	Yes, younger parental age improves children's mental health	3	Yes, a younger age (up to 21) may negatively affect her mental health	3	Improving access to specialist care (more therapists, shorter waiting times), improving the affordability of mental health care, raising awareness of mental health through campaigns	Occasionally
23	2	Moravian-Silesian Region	2	0	0	Economic conditions (e.g., financial stress), Social media and technology, Studijní nebo pracovní tlak, Rodinné vztahy	Rural environment	Easier access to medical and mental health care, Greater social and cultural opportunities (cinemas, concerts, cafés, etc.), Accessibility of employment and education		A combination of urban and rural living	4	I don't know	2	I don't know	3	Improving access to professional care (more therapists, shorter waiting times), Raising awareness of mental health through campaigns	Never
26	1	Olomouc Region	3	0	1	Economic conditions (e.g., financial stress), Social media and technology, Studijní nebo pracovní tlak, Rodinné vztahy	Urban environment	Greater social and cultural opportunities (cinemas, concerts, cafés, etc.), better access to public transport	Higher levels of stress and pace of life, Lack of nature and quiet places, Greater competitive pressure in the job market	In a city	3	No, parental age has no effect	1	I don't know	1	Improving access to specialist care (more therapists, shorter waiting times), creating a better preventive care system	Always
22	1	Moravian-Silesian Region	5	0	2	Economic conditions (e.g., financial stress), Social media and technology, Studijní nebo pracovní tlak, Rodinné vztahy	Urban environment	Greater social and cultural opportunities (cinemas, concerts, cafés, etc.), Accessibility of employment and education, Opportunity to establish more social contacts	Higher levels of stress and pace of life, Lack of nature and quiet places, Greater competitive pressure in the job market	A combination of urban and rural living	5	No, parental age has no effect	1	Yes, a younger age (up to 21) may negatively affect her mental health	2	Improving the affordability of mental health care, Greater support in schools and universities	Occasionally
21	2	Moravian-Silesian Region	3	0	0	Economic conditions (e.g., financial stress), Social media and technology, Studijní nebo pracovní tlak, Rodinné vztahy	Urban environment	Greater social and cultural opportunities (cinemas, concerts, cafés, etc.), Accessibility of employment and education, Better accessibility of public transport, Opportunity to establish more social contacts	Lack of nature and quiet places, Greater competitive pressure in the job market	A combination of urban and rural living	5	I don't know	1	I don't know	1	Improving access to specialist care (more therapists, shorter waiting times), improving the affordability of mental health care, raising awareness of mental health through campaigns, greater support in schools and universities	Often
18	1	Olomouc Region	3	2	2	Economic conditions (e.g., financial stress), Social media and technology, Studijní nebo pracovní tlak, Rodinné vztahy	Urban environment	Better accessibility of public transport	Higher levels of stress and pace of life, More noise and pollution, Lack of nature and quiet places, Higher crime rates	In a city	5	Yes, older parental age improves children's mental health	4	Yes, a younger age (up to 21) may negatively affect her mental health	1	Improved access to specialist care (more therapists, shorter waiting times), improved affordability of mental health care, greater support in schools and universities	Often
23	1	Moravian-Silesian Region	4	2	1	Economic conditions (e.g., financial stress), Social media and technology, Studijní nebo pracovní tlak, Rodinné vztahy	Urban environment	Greater social and cultural opportunities (cinemas, concerts, cafés, etc.), Accessibility of employment and education, Opportunity to establish more social contacts	More noise and pollution, Lack of nature and quiet places	In the countryside	5	Yes, younger parental age improves children's mental health	2	Yes, a younger age (up to 21) may negatively affect her mental health	2	Improving access to specialist care (more therapists, shorter waiting times), improving the affordability of mental health care, creating a better preventive care system	Always
19	1	Olomouc Region	4	0	0	Economic conditions (e.g., financial stress), Social media and technology, Studijní nebo pracovní tlak, Rodinné vztahy	Rural environment	Easier access to medical and mental health care, Greater anonymity and privacy		A combination of urban and rural living	5	No, parental age has no effect	2	Yes, a younger age (up to 21) may negatively affect her mental health	2	Raising awareness of mental health through campaigns, Greater support in schools and universities, Creating a better preventive care system	Occasionally
25	2	Moravian-Silesian Region	2	0	1	Economic conditions (e.g., financial stress), Social media and technology, Studijní nebo pracovní tlak, Rodinné vztahy	Mixed (on the outskirts of a city or in small towns)	Greater social and cultural opportunities (cinemas, concerts, cafés, etc.), Accessibility of employment and education, Better accessibility of public transport, Opportunity to establish more social contacts	Higher levels of stress and pace of life, More noise and pollution, Lack of nature and quiet places, Greater anonymity and feelings of loneliness, Higher crime rates	A combination of urban and rural living	5	Yes, younger parental age improves children's mental health	3	Yes, an older age (over 21) may positively affect her mental health	2	Improving access to specialist care (more therapists, shorter waiting times), creating a better preventive care system	Occasionally
20	2	Moravian-Silesian Region	3	0	2	Economic conditions (e.g., financial stress), Social media and technology, Studijní nebo pracovní tlak, Rodinné vztahy	Urban environment	Easier access to medical and mental health care, Greater social and cultural opportunities (cinemas, concerts, cafés, etc.), Greater anonymity and privacy, Better public transport accessibility	Higher levels of stress and pace of life, More noise and pollution, Lack of nature and quiet places	A combination of urban and rural living	4	Yes, younger parental age improves children's mental health	2	Yes, a younger age (up to 21) may negatively affect her mental health	2	Improved access to professional care (more therapists, shorter waiting times), Greater support in schools and universities	Occasionally
21	1	Moravian-Silesian Region	4	0	1	Economic conditions (e.g., financial stress), Social media and technology, Studijní nebo pracovní tlak, Rodinné vztahy	Mixed (on the outskirts of a city or in small towns)	Greater social and cultural opportunities (cinemas, concerts, cafés, etc.), Accessibility of employment and education, Opportunity to establish more social contacts	Higher levels of stress and pace of life, More noise and pollution, Greater competitive pressure in the job market	In the countryside	5	Yes, younger parental age improves children's mental health	2	Yes, an older age (over 21) may positively affect her mental health	1	Improved access to specialist care (more therapists, shorter waiting times), Improved affordability of mental health care	Always
25	2	Moravian-Silesian Region	4	0	0	Economic conditions (e.g., financial stress), Social media and technology, Studijní nebo pracovní tlak, Rodinné vztahy	Urban environment	Accessibility of employment and education, Greater anonymity and privacy, Opportunity to establish more social contacts	Higher levels of stress and	In a city	3	Yes, older parental age improves children's mental health	2	Yes, a younger age (up to 21) may negatively affect her mental health	3	Raising awareness of mental health through campaigns, Creating a better preventive care system	Never
23	1	Moravian-Silesian Region	4	0	0	Economic conditions (e.g., financial stress), Social media and technology, Studijní nebo pracovní tlak, Rodinné vztahy	Urban environment	Greater social and cultural opportunities (cinemas, concerts, cafés, etc.), Accessibility of employment and education, Greater anonymity and privacy	Higher levels of stress and pace of life, Lack of nature and quiet places, Greater competitive pressure in the job market	A combination of urban and rural living	2	No, parental age has no effect	1	No, maternal age has no effect on her mental health	2	Improving access to specialist care (more therapists, shorter waiting times), improving the affordability of mental health care, raising awareness of mental health through campaigns	Often
26	1	Ústí nad Labem Region	3	0	1	Economic conditions (e.g., financial stress), Social media and technology, Studijní nebo pracovní tlak, Rodinné vztahy	Mixed (on the outskirts of a city or in small towns)	Easier access to medical and mental health care, Greater social and cultural opportunities (cinemas, concerts, cafés, etc.), Accessibility of employment and education, Greater anonymity and privacy, Better accessibility of public transport, Opportunity to establish more social contacts	Higher levels of stress and pace of life, Lack of nature and quiet places, Greater anonymity and feelings of loneliness, Higher crime rates	In a city	4	No, parental age has no effect	1	Yes, a younger age (up to 21) may negatively affect her mental health	1	Improving access to specialist care (more therapists, shorter waiting times), improving the affordability of mental health care, raising awareness of mental health through campaigns, greater support in schools and universities	Often
21	1	Zlín Region	1	3	1	Economic conditions (e.g., financial stress), Social media and technology, Studijní nebo pracovní tlak, Rodinné vztahy	Rural environment	Easier access to medical and mental health care, Greater social and cultural opportunities (cinemas, concerts, cafés, etc.), Accessibility of employment and education		In the countryside	5	No, parental age has no effect	1	Yes, a younger age (up to 21) may negatively affect her mental health	3	Improved access to professional care (more therapists, shorter waiting times), Greater support in schools and universities	Often
21	2	Moravian-Silesian Region	4	0	0	Economic conditions (e.g., financial stress), Social media and technology, Studijní nebo pracovní tlak, Rodinné vztahy	Urban environment	Easier access to medical and mental health care, Accessibility of employment and education, Opportunity to establish more social contacts	Higher levels of stress and pace of life, More noise and pollution, Lack of nature and quiet places	A combination of urban and rural living	5	Yes, older parental age improves children's mental health	4	Yes, a younger age (up to 21) may negatively affect her mental health	1	Raising awareness of mental health through campaigns, Greater support in schools and universities	Always
24	1	Moravian-Silesian Region	5	0	2	Economic conditions (e.g., financial stress), Social media and technology, Studijní nebo pracovní tlak, Rodinné vztahy	Urban environment		Higher levels of stress and pace of life, Greater anonymity and feelings of loneliness, Greater competitive pressure in the job market	A combination of urban and rural living	5	Yes, younger parental age improves children's mental health	1	Yes, a younger age (up to 21) may negatively affect her mental health	3	Greater support in schools and universities, Creation of a better preventive care system	Occasionally
22	2	Moravian-Silesian Region	4	0	0	Economic conditions (e.g., financial stress), Social media and technology, Studijní nebo pracovní tlak, Rodinné vztahy	Urban environment	Easier access to medical and mental health care, greater anonymity and privacy, the overall concept of the city—everything is more accessible. Some people see this as a negative, but I see it as a positive aspect of the city.		A combination of urban and rural living	4	Yes, older parental age improves children's mental health	1	I don't know	2	Improving access to specialist care (more therapists, shorter waiting times), creating a better preventive care system	Occasionally
22	1	Capital City of Prague	3	0	0	Economic conditions (e.g., financial stress), Social media and technology, Studijní nebo pracovní tlak, Rodinné vztahy	Urban environment		Higher levels of stress and pace of life, More noise and pollution, Greater anonymity and feelings of loneliness	A combination of urban and rural living	2	No, parental age has no effect	1	I don't know	2	Raising awareness of mental health through campaigns, Greater support in schools and universities, Creating a better preventive care system	Occasionally
20	2	South Moravian Region	5	0	0	Economic conditions (e.g., financial stress), Social media and technology, Studijní nebo pracovní tlak, Rodinné vztahy	Urban environment	Easier access to medical and mental health care, Greater social and cultural opportunities (cinemas, concerts, cafés, etc.), Better public transport accessibility	Higher levels of stress and pace of life, More noise and pollution, Lack of nature and quiet places	A combination of urban and rural living	3	I don't know	3	Yes, a younger age (up to 21) may negatively affect her mental health	2	Improving the affordability of mental health care, Raising awareness of mental health through campaigns	Occasionally
19	2	Moravian-Silesian Region	4	0	0	Economic conditions (e.g., financial stress), Social media and technology, Studijní nebo pracovní tlak, Rodinné vztahy	Mixed (on the outskirts of a city or in small towns)	Greater social and cultural opportunities (cinemas, concerts, cafés, etc.), Greater anonymity and privacy, Better access to public transport, Opportunity to make more social contacts	More noise and pollution, Lack of nature and quiet places, Higher crime rate	A combination of urban and rural living	2	I don't know	1	I don't know	1	Improved access to specialist care (more therapists, shorter waiting times), Improved affordability of mental health care	Always
22	1	South Moravian Region	4	0	2	Economic conditions (e.g., financial stress), Social media and technology, Studijní nebo pracovní tlak, Rodinné vztahy	Urban environment		Higher crime rates, greater competitive pressure in the labor market	In a city	4	Yes, older parental age improves children's mental health	3	Yes, a younger age (up to 21) may negatively affect her mental health	3	Improving the affordability of mental health care, Greater support in schools and universities	Often
24	2	South Moravian Region	4	0	0	Economic conditions (e.g., financial stress), Social media and technology, Studijní nebo pracovní tlak, Rodinné vztahy	Urban environment	Easier access to medical and mental health care, Greater social and cultural opportunities (cinemas, concerts, cafés, etc.), Accessibility of employment and education, Opportunity to establish more social contacts	Higher levels of stress and pace of life, More noise and pollution, Lack of nature and quiet places, Greater anonymity and feelings of loneliness	A combination of urban and rural living	4	I don't know	2	Yes, a younger age (up to 21) may negatively affect her mental health	3	Improving access to specialist care (more therapists, shorter waiting times), improving the affordability of mental health care, creating a better preventive care system	Occasionally
22	2	Moravian-Silesian Region	4	0	3	Economic conditions (e.g., financial stress), Social media and technology, Studijní nebo pracovní tlak, Rodinné vztahy, Kriminalita a pocit nebezpečí	Rural environment	Easier access to medical and mental health care, Greater social and cultural opportunities (cinemas, concerts, cafés, etc.), Accessibility of employment and education	Higher levels of stress and pace of life, More noise and pollution, Lack of nature and quiet places	In the countryside	4	Yes, older parental age improves children's mental health	5	Yes, a younger age (up to 21) may negatively affect her mental health	3	Improving access to specialist care (more therapists, shorter waiting times), improving the affordability of mental health care, raising awareness of mental health through campaigns, greater support in schools and universities	Occasionally
22	1	Moravian-Silesian Region	2	0	2	Economic conditions (e.g., financial stress), Social media and technology, Studijní nebo pracovní tlak, Rodinné vztahy, Kriminalita a pocit nebezpečí	Rural environment	I don't think these things contribute to mental health.	Higher levels of stress and pace of life, Lack of nature and quiet places, Greater anonymity and feelings of loneliness, Higher crime rates, Greater competitive pressure in the job market	In the countryside	5	Yes, older parental age improves children's mental health	2	I don't know	3	Improving access to professional care (more therapists, shorter waiting times), improving the financial accessibility of mental health care, Greater support in schools and universities, Young people are often influenced by their family and surroundings, and it is not always possible to have general financial support, so this can result in us having no personal life, and the stress of moving between school and work, for example in my case, has a negative impact on my mental state.	Always
26	1	South Bohemian Region	3	0	0	Economic conditions (e.g., financial stress), Social media and technology, Studijní nebo pracovní tlak, Rodinné vztahy, Kriminalita a pocit nebezpečí	Mixed (on the outskirts of a city or in small towns)			In the countryside	5	No, parental age has no effect	3	Yes, a younger age (up to 21) may negatively affect her mental health	2		Often

Age	Gender	Region of Permanent Residence:	Q1. How would you rate your current mental state? (1 = Very poor, 5 = Very good)	Q2. If you sought professional help for your mental health (e.g., psychologist, psychiatrist), how would you rate the quality of these services? (1 = Very dissatisfied, 5 = Very satisfied)	Q3. Do you think mental health care is easily accessible in your city/region?	Q4: Which factors do you think affect the mental health of young people aged 18–25 the most? (select the 3 most important)	Q5. Do you live in an urban or rural environment?	Q6. What benefits of living in the city do you think have a positive impact on mental health? (select the 3 most important)	Q7. What disadvantages of living in the city do you think deteriorate mental health? (select the 3 most important)	Q8. If you could choose, where would you prefer to live in terms of mental health?	Q9. How important is support from family and friends to you in dealing with mental health problems? (1 = Not important, 5 = Very important)	Q10. Do you think the age at which people enter parenthood can affect the mental health of their children?	Q11. How much of an impact do you think crime in your region has on your mental health? (1 = No effect, 5 = Very strong effect)	Q12. Do you think the age of the first-time mother can affect her mental health?	Q13.How would you rate the level of general awareness and education about mental health in secondary schools and colleges? (1 = Very poor, 5 = Very good)	Q14. Which of the following do you think would be most helpful in improving mental health care for young people in your region? (select up to 2 options)	Q15. How often do you feel you have to look after your mental health on your own, without the help of professionals or family?
20	1	Moravian-Silesian Region	3	0	0	Economic conditions (e.g., financial stress), Social media and technology, Studijní nebo pracovní tlak, Rodinné vztahy, Nedostatečná dostupnost zdravotní péče	Urban environment	Easier access to medical and mental health care, Greater social and cultural opportunities (cinemas, concerts, cafés, etc.), Opportunity to establish more social contacts	More noise and pollution, higher crime rates	A combination of urban and rural living	5	Yes, older parental age improves children's mental health	4	Yes, a younger age (up to 21) may negatively affect her mental health	3	Improving access to specialist care (more therapists, shorter waiting times), improving the affordability of mental health care, raising awareness of mental health through campaigns, greater support in schools and universities	Often
24	1	South Moravian Region	4	0	2	Economic conditions (e.g., financial stress), Social media and technology, Studijní nebo pracovní tlak, Rodinné vztahy, Osamělost a sociální izolace	Urban environment	Easier access to medical and mental health care, Greater social and cultural opportunities (cinemas, concerts, cafés, etc.), Accessibility of employment and education, Greater anonymity and privacy, Better accessibility of public transport		A combination of urban and rural living	4	No, parental age has no effect	2	Yes, a younger age (up to 21) may negatively affect her mental health	3	Improving access to specialist care (more therapists, shorter waiting times), improving the affordability of mental health care, raising awareness of mental health through campaigns	Often
26	1	Moravian-Silesian Region	5	5	2	Economic conditions (e.g., financial stress), Social media and technology, Studijní nebo pracovní tlak, Rodinné vztahy, Osamělost a sociální izolace	Mixed (on the outskirts of a city or in small towns)	Easier access to medical and mental health care, Accessibility of employment and education, Better accessibility of public transport	Higher levels of stress and pace of life, Lack of nature and quiet places, Greater anonymity and feelings of loneliness	A combination of urban and rural living	5	Yes, older parental age improves children's mental health	3	Yes, an older age (over 21) may positively affect her mental health	2	Improved access to professional care (more therapists, shorter waiting times), Greater support in schools and universities	Always
19	1	Moravian-Silesian Region	3	0	1	Economic conditions (e.g., financial stress), Social media and technology, Studijní nebo pracovní tlak, Rodinné vztahy, Osamělost a sociální izolace	Urban environment	Greater social and cultural opportunities (cinemas, concerts, cafés, etc.), Greater anonymity and privacy, Opportunity to establish more social contacts	More noise and pollution, Lack of nature and quiet places, Greater competitive pressure in the job market	A combination of urban and rural living	5	I don't know	3	Yes, a younger age (up to 21) may negatively affect her mental health	2	Improving the affordability of mental health care, Greater support in schools and universities	Often
20	2	Moravian-Silesian Region	5	0	0	Economic conditions (e.g., financial stress), Social media and technology, Studijní nebo pracovní tlak, Rodinné vztahy, Osamělost a sociální izolace	Rural environment	Access to employment and education		In the countryside	3	Yes, younger parental age improves children's mental health	2	No, maternal age has no effect on her mental health	2	Greater support in schools and universities	Always
23	2	Moravian-Silesian Region	2	0	0	Economic conditions (e.g., financial stress), Social media and technology, Studijní nebo pracovní tlak, Rodinné vztahy, Osamělost a sociální izolace	Mixed (on the outskirts of a city or in small towns)	Greater social and cultural opportunities (cinemas, concerts, cafés, etc.), Accessibility of employment and education, Greater anonymity and privacy, Better accessibility of public transport, Opportunity to establish more social contacts	Higher levels of stress and pace of life, More noise and pollution, Lack of nature and quiet places	In the countryside	5	No, parental age has no effect	2	No, maternal age has no effect on her mental health	2	Improving access to specialist care (more therapists, shorter waiting times), improving the affordability of mental health care, raising awareness of mental health through campaigns, greater support in schools and universities, creating a better preventive care system.	Never
23	2	Moravian-Silesian Region	5	0	0	Economic conditions (e.g., financial stress), Social media and technology, Studijní nebo pracovní tlak, Rodinné vztahy, Osamělost a sociální izolace	Rural environment	Easier access to medical and mental health care, Greater social and cultural opportunities (cinemas, concerts, cafés, etc.), Accessibility of employment and education, Better accessibility of public transport	More noise and pollution, Lack of nature and quiet places	A combination of urban and rural living	2	No, parental age has no effect	1	No, maternal age has no effect on her mental health	3	Zlepšení dostupnosti odborné péče (více terapeutů, kratší čekací doby), Zvýšení povědomí o duševním zdraví prostřednictvím kampaní, Větší podpora na školách a univerzitách, Vytvoření lepšího systému preventivní péče	Always
21	2	Moravian-Silesian Region	3	0	2	Economic conditions (e.g., financial stress), Social media and technology, Studijní nebo pracovní tlak, Rodinné vztahy, Osamělost a sociální izolace	Rural environment	Easier access to medical and mental health care, Accessibility of employment and education		In the countryside	4	Yes, older parental age improves children's mental health	2	Yes, a younger age (up to 21) may negatively affect her mental health	3	Raising awareness of mental health through campaigns, Creating a better preventive care system	Often
22	2	Zlín Region	3	0	1	Economic conditions (e.g., financial stress), Social media and technology, Studijní nebo pracovní tlak, Rodinné vztahy, Osamělost a sociální izolace	Mixed (on the outskirts of a city or in small towns)		Higher levels of stress and pace of life, More noise and pollution, Lack of nature and quiet places	I have no proference	4	I don't know	1	Yes, a younger age (up to 21) may negatively affect her mental health	2	Improved access to specialist care (more therapists, shorter waiting times), Improved affordability of mental health care	Often
22	2	Olomouc Region	3	0	0	Economic conditions (e.g., financial stress), Social media and technology, Studijní nebo pracovní tlak, Rodinné vztahy, Osamělost a sociální izolace	Mixed (on the outskirts of a city or in small towns)	Greater social and cultural opportunities (cinemas, concerts, cafés, etc.), Accessibility of employment and education, Greater anonymity and privacy, Opportunity to establish more social contacts	Higher levels of stress and pace of life, More noise and pollution, Lack of nature and quiet places	A combination of urban and rural living	2	Yes, younger parental age improves children's mental health	2	Yes, a younger age (up to 21) may negatively affect her mental health	1	Greater support in schools and universities, Creation of a better preventive care system	Always
22	2	South Moravian Region	4	0	0	Economic conditions (e.g., financial stress), Social media and technology, Studijní nebo pracovní tlak, Rodinné vztahy, Osamělost a sociální izolace	Urban environment	Leisure activities, friends, healthy climate, green environment	Higher levels of stress and pace of life, More noise and pollution, Lack of nature and quiet places, Higher crime rates	A combination of urban and rural living	5	I don't know	2	I don't know	1	Raising awareness of mental health through campaigns, Greater support in schools and universities, Creating a better preventive care system	Always
20	2	Moravian-Silesian Region	4	0	3	Economic conditions (e.g., financial stress), Social media and technology, Studijní nebo pracovní tlak, Rodinné vztahy, Osamělost a sociální izolace	Rural environment		Higher levels of stress and pace of life, More noise and pollution, Lack of nature and quiet places	In the countryside	5	No, parental age has no effect	2	I don't know	4	Improving the affordability of mental health care, Greater support in schools and universities	Often
21	1	Olomouc Region	3	3	1	Economic conditions (e.g., financial stress), Social media and technology, Studijní nebo pracovní tlak, Rodinné vztahy, Osamělost a sociální izolace	Urban environment	Easier access to medical and mental health care, Greater social and cultural opportunities (cinemas, concerts, cafés, etc.), Accessibility of employment and education	Higher levels of stress and pace of life, More noise and pollution, Lack of nature and quiet places	In the countryside	4	No, parental age has no effect	1	Yes, a younger age (up to 21) may negatively affect her mental health	3	Greater support in schools and universities, Creation of a better preventive care system	Often
23	2	Vysočina Region	5	0	0	Economic conditions (e.g., financial stress), Social media and technology, Studijní nebo pracovní tlak, Rodinné vztahy, Osamělost a sociální izolace	Mixed (on the outskirts of a city or in small towns)	Greater social and cultural opportunities (cinemas, concerts, cafés, etc.), Accessibility of employment and education, Opportunity to establish more social contacts	Higher levels of stress and pace of life, More noise and pollution, Lack of nature and quiet places, Greater anonymity and feelings of loneliness, Higher crime rates, Greater competitive pressure in the job market	A combination of urban and rural living	4	No, parental age has no effect	1	Yes, an older age (over 21) may positively affect her mental health	4	Improving access to specialist care (more therapists, shorter waiting times), improving the affordability of mental health care, raising awareness of mental health through campaigns, greater support in schools and universities, creating a better preventive care system.	Often
23	1	Olomouc Region	4	0	0	Economic conditions (e.g., financial stress), Social media and technology, Studijní nebo pracovní tlak, Rodinné vztahy, Osamělost a sociální izolace	Rural environment	Greater social and cultural opportunities (cinemas, concerts, cafés, etc.), better access to public transport, opportunity to make more social contacts		In the countryside	5	Yes, younger parental age improves children's mental health	4	No, maternal age has no effect on her mental health	2	Raising awareness of mental health through campaigns, Greater support in schools and universities	Often
20	1	Moravian-Silesian Region	4	0	0	Economic conditions (e.g., financial stress), Social media and technology, Studijní nebo pracovní tlak, Rodinné vztahy, Osamělost a sociální izolace	Rural environment	Greater social and cultural opportunities (cinemas, concerts, cafés, etc.), Accessibility of employment and education	Higher levels of stress and pace of life, Lack of nature and quiet places	In the countryside	3	No, parental age has no effect	2	Yes, a younger age (up to 21) may negatively affect her mental health	1	Greater support in schools and universities, Creation of a better preventive care system	Occasionally
22	1	Moravian-Silesian Region	4	0	0	Economic conditions (e.g., financial stress), Social media and technology, Studijní nebo pracovní tlak, Rodinné vztahy, Osamělost a sociální izolace	Rural environment	Greater social and cultural opportunities (cinemas, concerts, cafés, etc.)	Lack of nature and quiet places, Greater competitive pressure in the job market	In the countryside	5	Yes, older parental age improves children's mental health	1	Yes, an older age (over 21) may positively affect her mental health	2	Improving the affordability of mental health care, Raising awareness of mental health through campaigns, Greater support in schools and universities, Creating a better preventive care system	Often
19	1	Moravian-Silesian Region	3	3	2	Economic conditions (e.g., financial stress), Social media and technology, Studijní nebo pracovní tlak, Rodinné vztahy, Osamělost a sociální izolace	Mixed (on the outskirts of a city or in small towns)	Easier access to medical and mental health care, Greater social and cultural opportunities (cinemas, concerts, cafés, etc.), Better public transport accessibility	Higher levels of stress and pace of life, Lack of nature and quiet places, Greater competitive pressure in the job market	A combination of urban and rural living	5	No, parental age has no effect	1	I don't know	1	Improved access to professional care (more therapists, shorter waiting times), Greater support in schools and universities	Always
22	2	Moravian-Silesian Region	3	0	3	Economic conditions (e.g., financial stress), Social media and technology, Studijní nebo pracovní tlak, Rodinné vztahy, Osamělost a sociální izolace	Rural environment	Easier access to medical and mental health care, Greater social and cultural opportunities (cinemas, concerts, cafés, etc.), Accessibility of employment and education, Greater anonymity and privacy, Better accessibility of public transport, Opportunity to establish more social contacts	Higher levels of stress and pace of life, More noise and pollution, Lack of nature and quiet places, Higher crime rates, Greater competitive pressure in the job market	A combination of urban and rural living	5	No, parental age has no effect	3	I don't know	3	Improving the affordability of mental health care, Raising awareness of mental health through campaigns, Greater support in schools and universities, Creating a better preventive care system	Never
23	2	Moravian-Silesian Region	2	4	2	Economic conditions (e.g., financial stress), Social media and technology, Studijní nebo pracovní tlak, Rodinné vztahy, Osamělost a sociální izolace	Urban environment	Easier access to medical and mental health care, Accessibility of employment and education, Better accessibility of public transport	Higher levels of stress and pace of life, More noise and pollution, Lack of nature and quiet places	A combination of urban and rural living	4	Yes, older parental age improves children's mental health	1	Yes, a younger age (up to 21) may negatively affect her mental health	2	Raising awareness of mental health through campaigns, Greater support in schools and universities	Often
19	1	South Moravian Region	3	0	0	Economic conditions (e.g., financial stress), Social media and technology, Studijní nebo pracovní tlak, Rodinné vztahy, Osamělost a sociální izolace	Urban environment	Easier access to medical and mental health care, Greater social and cultural opportunities (cinemas, concerts, cafés, etc.), Better public transport accessibility	Higher levels of stress and pace of life, Lack of nature and quiet places, Greater anonymity and feelings of loneliness	A combination of urban and rural living	5	Yes, younger parental age improves children's mental health	1	Yes, a younger age (up to 21) may negatively affect her mental health	3	Improving the affordability of mental health care, Creating a better preventive care system	Often
21	1	Zlín Region	2	3	2	Economic conditions (e.g., financial stress), Social media and technology, Studijní nebo pracovní tlak, Rodinné vztahy, Osamělost a sociální izolace	Rural environment	Greater social and cultural opportunities (cinemas, concerts, cafés, etc.), Accessibility of employment and education, Better accessibility of public transport	Higher levels of stress and pace of life, Lack of nature and quiet places, Higher crime rates	In the countryside	4	No, parental age has no effect	3	I don't know	2	Improved access to specialist care (more therapists, shorter waiting times), Improved affordability of mental health care	Often
19	2	South Bohemian Region	3	0	0	Economic conditions (e.g., financial stress), Social media and technology, Studijní nebo pracovní tlak, Rodinné vztahy, Osamělost a sociální izolace	Mixed (on the outskirts of a city or in small towns)	Greater social and cultural opportunities (cinemas, concerts, cafés, etc.), availability of recreational activities – sports/classmates...		A combination of urban and rural living	1	Yes, older parental age improves children's mental health	1	Yes, a younger age (up to 21) may negatively affect her mental health	2	Improved access to professional care (more therapists, shorter waiting times), greater support in schools and universities, licensed therapists, not just membership of some kind of therapists' organization where all you need to do is put a sign on the door.	Always
22	2	South Moravian Region	3	0	0	Economic conditions (e.g., financial stress), Social media and technology, Studijní nebo pracovní tlak, Rodinné vztahy, Osamělost a sociální izolace	Mixed (on the outskirts of a city or in small towns)		Higher levels of stress and pace of life, More noise and pollution, Lack of nature and quiet places, Greater anonymity and feelings of loneliness, Higher crime rates, Greater competitive pressure in the job market	A combination of urban and rural living	4	I don't know	5	Yes, a younger age (up to 21) may negatively affect her mental health	1		Always
21	1	Zlín Region	4	0	0	Economic conditions (e.g., financial stress), Social media and technology, Studijní nebo pracovní tlak, Rodinné vztahy, Osamělost a sociální izolace	Mixed (on the outskirts of a city or in small towns)	Easier access to medical and mental health care, Greater social and cultural opportunities (cinemas, concerts, cafés, etc.), Greater anonymity and privacy, Better public transport accessibility		In a city	5	I don't know	3	No, maternal age has no effect on her mental health	4	Improving access to specialist care (more therapists, shorter waiting times), improving the affordability of mental health care, raising awareness of mental health through campaigns, greater support in schools and universities, creating a better preventive care system.	Often
20	1	Central Bohemian Region	3	4	2	Economic conditions (e.g., financial stress), Social media and technology, Studijní nebo pracovní tlak, Rodinné vztahy, Osamělost a sociální izolace	Rural environment	Easier access to medical and mental health care, Greater social and cultural opportunities (cinemas, concerts, cafés, etc.), Accessibility of employment and education, Better accessibility of public transport	More noise and pollution, Lack of nature and quiet places	A combination of urban and rural living	4	I don't know	2	Yes, a younger age (up to 21) may negatively affect her mental health	2	Improved access to specialist care (more therapists, shorter waiting times), improved affordability of mental health care, greater support in schools and universities, creation of a better preventive care system.	Always
23	2	Central Bohemian Region	3	4	2	Economic conditions (e.g., financial stress), Social media and technology, Studijní nebo pracovní tlak, Rodinné vztahy, Osamělost a sociální izolace	Urban environment	Accessibility of employment and education, Better accessibility of public transport		I have no proference	5	Yes, older parental age improves children's mental health	1	Yes, a younger age (up to 21) may negatively affect her mental health	2	Improved access to specialist care (more therapists, shorter waiting times), improved affordability of mental health care, greater support in schools and universities	Often
20	1	Moravian-Silesian Region	4	0	2	Economic conditions (e.g., financial stress), Social media and technology, Studijní nebo pracovní tlak, Rodinné vztahy, Osamělost a sociální izolace	Urban environment	Greater social and cultural opportunities (cinemas, concerts, cafés, etc.), Accessibility of employment and education	Higher levels of stress and pace of life, Lack of nature and quiet places, Greater competitive pressure in the job market	In the countryside	4	I don't know	1	Yes, a younger age (up to 21) may negatively affect her mental health	1	Raising awareness of mental health through campaigns, Greater support in schools and universities, Creating a better preventive care system	Always
21	1	South Moravian Region	2	0	2	Economic conditions (e.g., financial stress), Social media and technology, Studijní nebo pracovní tlak, Rodinné vztahy, Osamělost a sociální izolace	Urban environment	Greater social and cultural opportunities (cinemas, concerts, cafés, etc.), Accessibility of employment and education, Better accessibility of public transport, Opportunity to establish more social contacts	Higher levels of stress and pace of life, greater competitive pressure in the labor market	A combination of urban and rural living	5	Yes, younger parental age improves children's mental health	2	Yes, an older age (over 21) may positively affect her mental health	3	Improving access to specialist care (more therapists, shorter waiting times), improving the affordability of mental health care, raising awareness of mental health through campaigns, greater support in schools and universities, creating a better preventive care system.	Always
20	2	Pilsen Region	4	0	3	Economic conditions (e.g., financial stress), Social media and technology, Studijní nebo pracovní tlak, Rodinné vztahy, Osamělost a sociální izolace	Mixed (on the outskirts of a city or in small towns)	Easier access to medical and mental health care, Accessibility of employment and education	Higher levels of stress and pace of life, Lack of nature and quiet places	In the countryside	1	Yes, younger parental age improves children's mental health	1	Yes, a younger age (up to 21) may negatively affect her mental health	1	Improved access to specialist care (more therapists, shorter waiting times), greater support in schools and universities, creation of a better preventive care system.	Always
21	2	South Moravian Region	3	0	2	Economic conditions (e.g., financial stress), Social media and technology, Studijní nebo pracovní tlak, Rodinné vztahy, Osamělost a sociální izolace	Urban environment		Higher levels of stress and pace of life, Lack of nature and quiet places, Higher crime rates	A combination of urban and rural living	5	Yes, younger parental age improves children's mental health	1	Yes, a younger age (up to 21) may negatively affect her mental health	2	Improving the affordability of mental health care, greater support in schools and universities, creating a better preventive care system, reintroducing traditional values into society, and specifically limiting dependence on modern technologies. Young people in my region have little contact with each other and do not form good, long-term relationships.	Always
21	2	South Moravian Region	2	4	2	Economic conditions (e.g., financial stress), Social media and technology, Studijní nebo pracovní tlak, Rodinné vztahy, Osamělost a sociální izolace	Urban environment	Accessibility of employment and education, Greater anonymity and privacy, Opportunity to establish more social contacts	Higher levels of stress and pace of life, More noise and pollution, Lack of nature and quiet places	A combination of urban and rural living	3	I don't know	1	I don't know	1	Greater support in schools and universities, Creation of a better preventive care system	Often
20	1	Zlín Region	3	4	2	Economic conditions (e.g., financial stress), Social media and technology, Studijní nebo pracovní tlak, Rodinné vztahy, Osamělost a sociální izolace	Urban environment	Greater social and cultural opportunities (cinemas, concerts, cafés, etc.), Accessibility of employment and education, Better accessibility of public transport	Higher levels of stress and pace of life, greater competitive pressure in the labor market	I have no proference	5	I don't know	3	Yes, a younger age (up to 21) may negatively affect her mental health	2	Zlepšení dostupnosti odborné péče (více terapeutů, kratší čekací doby), Zvýšení povědomí o duševním zdraví prostřednictvím kampaní, Větší podpora na školách a univerzitách, Vytvoření lepšího systému preventivní péče	Often
22	2	Central Bohemian Region	3	0	0	Economic conditions (e.g., financial stress), Social media and technology, Studijní nebo pracovní tlak, Rodinné vztahy, Osamělost a sociální izolace	Urban environment	Greater social and cultural opportunities (cinemas, concerts, cafés, etc.), Accessibility of employment and education, Greater anonymity and privacy, Better accessibility of public transport, Opportunity to establish more social contacts	Higher levels of stress and pace of life, More noise and pollution, Lack of nature and quiet places, Greater competitive pressure in the job market	A combination of urban and rural living	4	Yes, younger parental age improves children's mental health	2	Yes, a younger age (up to 21) may negatively affect her mental health	3	Improving access to specialist care (more therapists, shorter waiting times), improving the affordability of mental health care, raising awareness of mental health through campaigns, greater support in schools and universities, creating a better preventive care system.	Often
22	2	South Moravian Region	3	0	0	Economic conditions (e.g., financial stress), Social media and technology, Studijní nebo pracovní tlak, Rodinné vztahy, Osamělost a sociální izolace	Mixed (on the outskirts of a city or in small towns)	Greater social and cultural opportunities (cinemas, concerts, cafés, etc.), Accessibility of employment and education, Better accessibility of public transport	Higher levels of stress and pace of life, Greater anonymity and feelings of loneliness, Greater competitive pressure in the job market	A combination of urban and rural living	4	No, parental age has no effect	1	Yes, a younger age (up to 21) may negatively affect her mental health	3	Greater support in schools and universities, Creation of a better preventive care system	Often
26	2	Moravian-Silesian Region	4	0	0	Economic conditions (e.g., financial stress), Social media and technology, Studijní nebo pracovní tlak, Rodinné vztahy, Osamělost a sociální izolace	Urban environment	Easier access to medical and mental health care, Greater social and cultural opportunities (cinemas, concerts, cafés, etc.), Accessibility of employment and education, Opportunity to establish more social contacts		A combination of urban and rural living	5	Yes, older parental age improves children's mental health	3	Yes, an older age (over 21) may positively affect her mental health	2	Raising awareness of mental health through campaigns, Greater support in schools and universities	Occasionally
20	1	Moravian-Silesian Region	4	0	2	Economic conditions (e.g., financial stress), Social media and technology, Studijní nebo pracovní tlak, Rodinné vztahy, Osamělost a sociální izolace	Mixed (on the outskirts of a city or in small towns)	Greater social and cultural opportunities (cinemas, concerts, cafés, etc.), Accessibility of employment and education, Opportunity to establish more social contacts	Higher levels of stress and pace of life, Lack of nature and quiet places, Greater competitive pressure in the job market	A combination of urban and rural living	5	No, parental age has no effect	4	I don't know	2	Greater support in schools and universities, Creation of a better preventive care system	Occasionally
20	1	Moravian-Silesian Region	2	4	2	Economic conditions (e.g., financial stress), Social media and technology, Studijní nebo pracovní tlak, Rodinné vztahy, Osamělost a sociální izolace	Mixed (on the outskirts of a city or in small towns)	Easier access to medical and mental health care, Greater social and cultural opportunities (cinemas, concerts, cafés, etc.), Greater anonymity and privacy	More noise and pollution, Lack of nature and quiet places, Higher crime rate	A combination of urban and rural living	5	Yes, younger parental age improves children's mental health	2	Yes, a younger age (up to 21) may negatively affect her mental health	2	Improved access to specialist care (more therapists, shorter waiting times), Improved affordability of mental health care	Often
22	1	Moravian-Silesian Region	2	3	2	Economic conditions (e.g., financial stress), Social media and technology, Studijní nebo pracovní tlak, Rodinné vztahy, Osamělost a sociální izolace	Mixed (on the outskirts of a city or in small towns)	Easier access to medical and mental health care, Greater social and cultural opportunities (cinemas, concerts, cafés, etc.), Accessibility of employment and education, Greater anonymity and privacy	Higher levels of stress and pace of life, More noise and pollution, Lack of nature and quiet places, Greater anonymity and feelings of loneliness, Higher crime rates, Greater competitive pressure in the job market	In the countryside	4	Yes, older parental age improves children's mental health	2	Yes, a younger age (up to 21) may negatively affect her mental health	2	Improving access to specialist care (more therapists, shorter waiting times), improving the affordability of mental health care, raising awareness of mental health through campaigns, greater support in schools and universities, creating a better preventive care system.	Always
22	1	Olomouc Region	1	2	2	Economic conditions (e.g., financial stress), Social media and technology, Studijní nebo pracovní tlak, Rodinné vztahy, Osamělost a sociální izolace	Mixed (on the outskirts of a city or in small towns)	Greater social and cultural opportunities (cinemas, concerts, cafés, etc.), Accessibility of employment and education, Better accessibility of public transport, Opportunity to establish more social contacts		A combination of urban and rural living	2	No, parental age has no effect	1	Yes, a younger age (up to 21) may negatively affect her mental health	3	Improving the affordability of mental health care, Greater support in schools and universities	Never
20	1	Moravian-Silesian Region	3	0	0	Economic conditions (e.g., financial stress), Social media and technology, Studijní nebo pracovní tlak, Rodinné vztahy, Osamělost a sociální izolace	Mixed (on the outskirts of a city or in small towns)	Greater social and cultural opportunities (cinemas, concerts, cafés, etc.), Accessibility of employment and education, Better accessibility of public transport		In the countryside	5	Yes, younger parental age improves children's mental health	3	Yes, a younger age (up to 21) may negatively affect her mental health	2	Raising awareness of mental health through campaigns, Greater support in schools and universities	Often
20	2	Moravian-Silesian Region	4	0	0	Economic conditions (e.g., financial stress), Social media and technology, Studijní nebo pracovní tlak, Rodinné vztahy, Osamělost a sociální izolace	Urban environment	Greater social and cultural opportunities (cinemas, concerts, cafés, etc.), Accessibility of employment and education, Opportunity to establish more social contacts	Higher levels of stress and pace of life, Lack of nature and quiet places, Greater competitive pressure in the job market	A combination of urban and rural living	5	I don't know	3	Yes, a younger age (up to 21) may negatively affect her mental health	2	Raising awareness of mental health through campaigns, Greater support in schools and universities	Always
22	1	Central Bohemian Region	3	2	1	Economic conditions (e.g., financial stress), Social media and technology, Studijní nebo pracovní tlak, Rodinné vztahy, Osamělost a sociální izolace	Urban environment	Easier access to medical and mental health care, Greater social and cultural opportunities (cinemas, concerts, cafés, etc.), Accessibility of employment and education, Opportunity to establish more social contacts	Higher levels of stress and pace of life, More noise and pollution, Lack of nature and quiet places	A combination of urban and rural living	5	No, parental age has no effect	1	I don't know	2	Raising awareness of mental health through campaigns, Greater support in schools and universities, Creating a better preventive care system	Often
23	2	Moravian-Silesian Region	3	1	1	Economic conditions (e.g., financial stress), Social media and technology, Studijní nebo pracovní tlak, Rodinné vztahy, Osamělost a sociální izolace	Rural environment	Greater social and cultural opportunities (cinemas, concerts, cafés, etc.), availability of employment and education, pubs (t2 bar)	Higher levels of stress and pace of life, More noise and pollution, Lack of nature and quiet places	In the countryside	4	Yes, older parental age improves children's mental health	1	Yes, a younger age (up to 21) may negatively affect her mental health	3	Improved access to professional care (more therapists, shorter waiting times), Greater support in schools and universities	Often
22	2	Central Bohemian Region	2	4	3	Economic conditions (e.g., financial stress), Social media and technology, Studijní nebo pracovní tlak, Rodinné vztahy, Osamělost a sociální izolace	Mixed (on the outskirts of a city or in small towns)	Easier access to medical and mental health care, Greater social and cultural opportunities (cinemas, concerts, cafés, etc.), Accessibility of employment and education, Opportunity to establish more social contacts	Higher levels of stress and pace of life, More noise and pollution, Lack of nature and quiet places, Greater anonymity and feelings of loneliness, Higher crime rates	A combination of urban and rural living	5	I don't know	4	I don't know	3	Improve access to professional care (more therapists, shorter waiting times), motivate parents to encourage their children to play sports and socialize.	Often
21	1	Moravian-Silesian Region	2	3	2	Economic conditions (e.g., financial stress), Social media and technology, Studijní nebo pracovní tlak, Rodinné vztahy, Osamělost a sociální izolace, Kriminalita a pocit nebezpečí	Rural environment	Greater social and cultural opportunities (cinemas, concerts, cafés, etc.), Accessibility of employment and education	Higher levels of stress and pace of life, More noise and pollution, Lack of nature and quiet places, Higher crime rates	A combination of urban and rural living	5	No, parental age has no effect	3	Yes, a younger age (up to 21) may negatively affect her mental health	3		Often
26	2	South Bohemian Region	3	3	1	Economic conditions (e.g., financial stress), Social media and technology, Studijní nebo pracovní tlak, Rodinné vztahy, Osamělost a sociální izolace, Kriminalita a pocit nebezpečí	Rural environment	Easier access to medical and mental health care, Accessibility of employment and education, Greater anonymity and privacy	Higher levels of stress and pace of life, More noise and pollution, Greater anonymity and feelings of loneliness, Higher crime rates, Greater competitive pressure in the job market	In the countryside	5	I don't know	5	I don't know	1	Improving the affordability of mental health care	Often
20	2	Moravian-Silesian Region	3	0	0	Economic conditions (e.g., financial stress), Social media and technology, Studijní nebo pracovní tlak, Rodinné vztahy, Osamělost a sociální izolace, Kriminalita a pocit nebezpečí	Mixed (on the outskirts of a city or in small towns)	Better accessibility of public transport	Higher levels of stress and pace of life, Lack of nature and quiet places, Higher crime rates	A combination of urban and rural living	5	Yes, younger parental age improves children's mental health	4	Yes, an older age (over 21) may positively affect her mental health	3	Raising awareness of mental health through campaigns, Greater support in schools and universities	Occasionally

Age	Gender	Region of Permanent Residence:	Q1. How would you rate your current mental state? (1 = Very poor, 5 = Very good)	Q2. If you sought professional help for your mental health (e.g., psychologist, psychiatrist), how would you rate the quality of these services? (1 = Very dissatisfied, 5 = Very satisfied)	Q3. Do you think mental health care is easily accessible in your city/region?	Q4: Which factors do you think affect the mental health of young people aged 18–25 the most? (select the 3 most important)	Q5. Do you live in an urban or rural environment?	Q6. What benefits of living in the city do you think have a positive impact on mental health? (select the 3 most important)	Q7. What disadvantages of living in the city do you think deteriorate mental health? (select the 3 most important)	Q8. If you could choose, where would you prefer to live in terms of mental health?	Q9. How important is support from family and friends to you in dealing with mental health problems? (1 = Not important, 5 = Very important)	Q10. Do you think the age at which people enter parenthood can affect the mental health of their children?	Q11. How much of an impact do you think crime in your region has on your mental health? (1 = No effect, 5 = Very strong effect)	Q12. Do you think the age of the first-time mother can affect her mental health?	Q13.How would you rate the level of general awareness and education about mental health in secondary schools and colleges? (1 = Very poor, 5 = Very good)	Q14. Which of the following do you think would be most helpful in improving mental health care for young people in your region? (select up to 2 options)	Q15. How often do you feel you have to look after your mental health on your own, without the help of professionals or family?
21	2	South Moravian Region	4	0	0	Economic conditions (e.g., financial stress), Social media and technology, Studijní nebo pracovní tlak, Rodinné vztahy, Osamělost a sociální izolace, Kriminalita a pocit nebezpečí	Urban environment	Accessibility of employment and education, Better accessibility of public transport, Opportunity to establish more social contacts		In the countryside	5	I don't know	2	Yes, an older age (over 21) may positively affect her mental health	4	Raising awareness of mental health through campaigns, Creating a better preventive care system	Occasionally
20	1	South Moravian Region	5	0	2	Economic conditions (e.g., financial stress), Social media and technology, Studijní nebo pracovní tlak, Rodinné vztahy, Osamělost a sociální izolace, Kriminalita a pocit nebezpečí	Mixed (on the outskirts of a city or in small towns)	Greater social and cultural opportunities (cinemas, concerts, cafés, etc.), Accessibility of employment and education, Better accessibility of public transport	Higher levels of stress and pace of life, More noise and pollution, Lack of nature and quiet places	In the countryside	4	No, parental age has no effect	2	No, maternal age has no effect on her mental health	1	Improving the affordability of mental health care, Raising awareness of mental health through campaigns	Occasionally
22	1	Moravian-Silesian Region	3	0	2	Economic conditions (e.g., financial stress), Social media and technology, Studijní nebo pracovní tlak, Rodinné vztahy, Osamělost a sociální izolace, Kriminalita a pocit nebezpečí	Urban environment	Greater social and cultural opportunities (cinemas, concerts, cafés, etc.), Accessibility of employment and education, Greater anonymity and privacy, Better accessibility of public transport	Higher levels of stress and pace of life, Lack of nature and quiet places, Higher crime rates	I have no proference	5	I don't know	4	I don't know	1	Improving access to specialist care (more therapists, shorter waiting times), improving the affordability of mental health care, raising awareness of mental health through campaigns, greater support in schools and universities, creating a better preventive care system.	Often
22	1	Moravian-Silesian Region	4	2	3	Economic conditions (e.g., financial stress), Social media and technology, Studijní nebo pracovní tlak, Rodinné vztahy, Osamělost a sociální izolace, Kriminalita a pocit nebezpečí	Rural environment	Greater social and cultural opportunities (cinemas, concerts, cafés, etc.), Accessibility of employment and education, Better accessibility of public transport		A combination of urban and rural living	5	No, parental age has no effect	1	Yes, a younger age (up to 21) may negatively affect her mental health	2	Improving access to specialist care (more therapists, shorter waiting times), creating a better preventive care system	Often
23	2	Moravian-Silesian Region	2	1	2	Economic conditions (e.g., financial stress), Social media and technology, Studijní nebo pracovní tlak, Rodinné vztahy, Osamělost a sociální izolace, Nedostatečná dostupnost zdravotní péče	Urban environment	Easier access to medical and mental health care, Greater social and cultural opportunities (cinemas, concerts, cafés, etc.), Accessibility of employment and education, Better accessibility of public transport, Opportunity to establish more social contacts, More free time	Higher levels of stress and	A combination of urban and rural living	3	I don't know	2	I don't know	1	Raising awareness of mental health through campaigns, Greater support in schools and universities, Creating a better preventive care system	Always
23	1	Moravian-Silesian Region	4	0	1	Economic conditions (e.g., financial stress), Social media and technology, Studijní nebo pracovní tlak, Rodinné vztahy, Osamělost a sociální izolace, Nedostatečná dostupnost zdravotní péče	Urban environment		More noise and pollution, Lack of nature and quiet places	In a city	4	No, parental age has no effect	2	Yes, a younger age (up to 21) may negatively affect her mental health	2	Improving access to specialist care (more therapists, shorter waiting times), improving the affordability of mental health care, raising awareness of mental health through campaigns, greater support in schools and universities, creating a better preventive care system.	Often
21	2	Moravian-Silesian Region	3	0	0	Economic conditions (e.g., financial stress), Social media and technology, Studijní nebo pracovní tlak, Rodinné vztahy, Osamělost a sociální izolace, Nedostatečná dostupnost zdravotní péče	Rural environment	Easier access to medical and mental health care, Greater social and cultural opportunities (cinemas, concerts, cafés, etc.), Accessibility of employment and education		In the countryside	5	I don't know	1	I don't know	2	Raising awareness of mental health through campaigns, Greater support in schools and universities	Often
20	1	Zlín Region	2	3	1	Economic conditions (e.g., financial stress), Social media and technology, Studijní nebo pracovní tlak, Rodinné vztahy, Osamělost a sociální izolace, Nedostatečná dostupnost zdravotní péče	Rural environment	Easier access to medical and mental health care, Accessibility of employment and education, Greater anonymity and privacy	Higher levels of stress and pace of life, More noise and pollution, Greater anonymity and feelings of loneliness	In the countryside	5	Yes, older parental age improves children's mental health	2	Yes, a younger age (up to 21) may negatively affect her mental health	3	Improved access to specialist care (more therapists, shorter waiting times), Improved affordability of mental health care	Always
20	1	Vysočina Region	5	5	1	Economic conditions (e.g., financial stress), Social media and technology, Studijní nebo pracovní tlak, Rodinné vztahy, Osamělost a sociální izolace, Nedostatečná dostupnost zdravotní péče	Urban environment	Accessibility of employment and education, Greater anonymity and privacy, Better accessibility of public transport, Opportunity to establish more social contacts	Higher levels of stress and pace of life, More noise and pollution, Lack of nature and quiet places, Greater competitive pressure in the job market	A combination of urban and rural living	5	Yes, older parental age improves children's mental health	2	I don't know	2	Improving access to specialist care (more therapists, shorter waiting times), improving the affordability of mental health care, raising awareness of mental health through campaigns, greater support in schools and universities, creating a better preventive care system.	Occasionally
21	1	Pardubice Region	4	5	2	Economic conditions (e.g., financial stress), Social media and technology, Studijní nebo pracovní tlak, Rodinné vztahy, Osamělost a sociální izolace, Nedostatečná dostupnost zdravotní péče	Urban environment	Easier access to medical and mental health care, Greater social and cultural opportunities (cinemas, concerts, cafés, etc.), Accessibility of employment and education, Greater anonymity and privacy, Better accessibility of public transport, Opportunity to establish more social contacts	Higher levels of stress and pace of life, greater competitive pressure in the labor market	In the countryside	5	I don't know	3	Yes, a younger age (up to 21) may negatively affect her mental health	2	Improving access to specialist care (more therapists, shorter waiting times), improving the affordability of mental health care, raising awareness of mental health through campaigns, greater support in schools and universities	Occasionally
20	1	Moravian-Silesian Region	3	0	2	Economic conditions (e.g., financial stress), Social media and technology, Studijní nebo pracovní tlak, Rodinné vztahy, Osamělost a sociální izolace, Nedostatečná dostupnost zdravotní péče, Zrychlená doba, obrovské [illegible]	Rural environment	Easier access to medical and mental health care, Greater social and cultural opportunities (cinemas, concerts, cafés, etc.), Accessibility of employment and education, Better accessibility of public transport, Opportunity to establish more social contacts	More noise and pollution, Lack of nature and quiet places	In the countryside	5	Yes, older parental age improves children's mental health	2	Yes, a younger age (up to 21) may negatively affect her mental health	1	Improving the affordability of mental health care, Raising awareness of mental health through campaigns	Often
21	2	Moravian-Silesian Region	3	0	0	Economic conditions (e.g., financial stress), Social media and technology, Studijní nebo pracovní tlak, Rodinné vztahy, Osamělost a sociální izolace, Vztahy obecně, nejen rodinné	Urban environment	Better accessibility of public transport		In the countryside	5	Yes, older parental age improves children's mental health	1	Yes, a younger age (up to 21) may negatively affect her mental health	3	Improving the affordability of mental health care, Greater support in schools and universities	Always
24	2	Moravian-Silesian Region	2	0	0	Economic conditions (e.g., financial stress), Study or work pressure	Urban environment	Greater social and cultural opportunities (cinemas, concerts, cafés, etc.), Accessibility of employment and education	Higher levels of stress and pace of life, More noise and pollution, Higher crime rates, Greater competitive pressure in the job market	I have no proference	3	Yes, older parental age improves children's mental health	2	I don't know	3	Improved access to specialist care (more therapists, shorter waiting times), Improved affordability of mental health care	Occasionally
23	1	Moravian-Silesian Region	1	0	0	Economic conditions (e.g., financial stress), Study or work pressure	Rural environment	Easier access to medical and mental health care, Greater social and cultural opportunities (cinemas, concerts, cafés, etc.), Better public transport accessibility	Higher levels of stress and pace of life, More noise and pollution, Lack of nature and quiet places	In the countryside	5	Yes, older parental age improves children's mental health	3	Yes, a younger age (up to 21) may negatively affect her mental health	1	Raising awareness of mental health through campaigns, Greater support in schools and universities	Always
19	1	Olomouc Region	4	0	0	Economic conditions (e.g., financial stress), Study or work pressure	Mixed (on the outskirts of a city or in small towns)	Access to employment and education, Greater anonymity and privacy	Higher levels of stress and pace of life, More noise and pollution	I have no proference	4	No, parental age has no effect	1	Yes, a younger age (up to 21) may negatively affect her mental health	2	Improving the affordability of mental health care, Greater support in schools and universities	Occasionally
21	1	Moravian-Silesian Region	4	0	0	Economic conditions (e.g., financial stress), Study or work pressure	Urban environment	Greater social and cultural opportunities (cinemas, concerts, cafés, etc.), Accessibility of employment and education	Higher levels of stress and pace of life, Lack of nature and quiet places, Higher crime rates	A combination of urban and rural living	5	No, parental age has no effect	2	I don't know	3	Creating a better preventive care system	Often
22	1	Olomouc Region	4	0	3	Economic conditions (e.g., financial stress), Study or work pressure	Urban environment	Accessibility of employment and education, Greater anonymity and privacy, Better accessibility of public transport		In a city	3	I don't know	2	Yes, a younger age (up to 21) may negatively affect her mental health	3	Creating a better preventive care system	Often
21	1	Moravian-Silesian Region	4	5	1	Economic conditions (e.g., financial stress), academic or work pressure, crime, and feelings of insecurity	Rural environment		Higher levels of stress and pace of life, Lack of nature and quiet places, Higher crime rates	A combination of urban and rural living	5	Yes, older parental age improves children's mental health	3	Yes, a younger age (up to 21) may negatively affect her mental health	2	Greater support in schools and universities, Creation of a better preventive care system	Often
23	2	Moravian-Silesian Region	1	1	0	Economic conditions (e.g., financial stress), academic or work pressure, crime, and feelings of insecurity	Rural environment	Accessibility of employment and education, Better accessibility of public transport		In a city	1	No, parental age has no effect	3	Yes, an older age (over 21) may positively affect her mental health	2	Raising awareness of mental health through campaigns	Never
22	1	Moravian-Silesian Region	4	4	1	Economic conditions (e.g., financial stress), Academic or work pressure, Inadequate access to healthcare	Mixed (on the outskirts of a city or in small towns)	Easier access to medical and mental health care, Greater social and cultural opportunities (cinemas, concerts, cafés, etc.), Accessibility of employment and education	Higher crime rate	In a city	1	Yes, older parental age improves children's mental health	3	Yes, a younger age (up to 21) may negatively affect her mental health	3	Improved access to specialist care (more therapists, shorter waiting times), Improved affordability of mental health care	Often
25	2	Moravian-Silesian Region	3	1	1	Economic conditions (e.g., financial stress), Academic or work pressure, Inadequate access to healthcare	Urban environment	Greater social and cultural opportunities (cinemas, concerts, cafés, etc.), Greater anonymity and privacy, Better access to public transport, Opportunity to make more social contacts	More noise and pollution, Lack of nature and quiet places, Greater competitive pressure in the job market	A combination of urban and rural living	2	No, parental age has no effect	1	Yes, a younger age (up to 21) may negatively affect her mental health	1	Improving access to specialist care (more therapists, shorter waiting times), improving the affordability of mental health care, raising awareness of mental health through campaigns, greater support in schools and universities, creating a better preventive care system.	Always
26	2	Zlín Region	4	5	2	Economic conditions (e.g., financial stress), Academic or work pressure, Inadequate access to healthcare	Urban environment			In a city	4	I don't know	1	I don't know	3	Improved access to specialist care (more therapists, shorter waiting times), Improved affordability of mental health care	Occasionally
23	2	South Moravian Region	2	3	1	Economic conditions (e.g., financial stress), Academic or work pressure, Loneliness and social isolation	Urban environment		Higher levels of stress and pace of life, More noise and pollution, Greater competitive pressure in the job market	A combination of urban and rural living	3	Yes, younger parental age improves children's mental health	2	Yes, an older age (over 21) may positively affect her mental health	2	Improving access to specialist care (more therapists, shorter waiting times), improving the affordability of mental health care, creating a better preventive care system	Always
26	2	Ústecký kraj	3	0	2	Economic conditions (e.g., financial stress), Academic or work pressure, Loneliness and social isolation	Urban environment	Greater social and cultural opportunities (cinemas, concerts, cafés, etc.), Accessibility of employment and education, Better accessibility of public transport	Higher levels of stress and pace of life, More noise and pollution, Lack of nature and quiet places	A combination of urban and rural living	5	Yes, younger parental age improves children's mental health	4	Yes, an older age (over 21) may positively affect her mental health	3	Raising awareness of mental health through campaigns, Creating a better preventive care system	Often
20	2	Moravian-Silesian Region	5	0	0	Economic conditions (e.g., financial stress), Academic or work pressure, Loneliness and social isolation	Rural environment	Easier access to medical and mental health care, Accessibility of employment and education, Better accessibility of public transport	More noise and pollution, Lack of nature and quiet places, Greater competitive pressure in the job market	In the countryside	5	I don't know	1	No, maternal age has no effect on her mental health	3	Improving access to specialist care (more therapists, shorter waiting times), creating a better preventive care system	Occasionally
22	1	Olomouc Region	4	0	0	Economic conditions (e.g., financial stress), Academic or work pressure, Loneliness and social isolation	Mixed (on the outskirts of a city or in small towns)	Greater social and cultural opportunities (cinemas, concerts, cafés, etc.), Accessibility of employment and education, Greater anonymity and privacy		A combination of urban and rural living	5	I don't know	1	Yes, a younger age (up to 21) may negatively affect her mental health	2	Improving the affordability of mental health care, Greater support in schools and universities	Occasionally
22	2	Moravian-Silesian Region	3	0	1	Economic conditions (e.g., financial stress), Academic or work pressure, Loneliness and social isolation	Urban environment	Greater social and cultural opportunities (cinemas, concerts, cafés, etc.), Accessibility of employment and education, Opportunity to establish more social contacts	Higher levels of stress and pace of life, More noise and pollution, Lack of nature and quiet places	A combination of urban and rural living	2	No, parental age has no effect	2	Yes, a younger age (up to 21) may negatively affect her mental health	1	Improved access to specialist care (more therapists, shorter waiting times), Improved affordability of mental health care	Always
21	1	Moravian-Silesian Region	3	0	1	Economic conditions (e.g., financial stress), Academic or work pressure, Loneliness and social isolation	Urban environment	Easier access to medical and mental health care, Greater social and cultural opportunities (cinemas, concerts, cafés, etc.), Better public transport accessibility	Higher levels of stress and pace of life, Lack of nature and quiet places, Higher crime rates	In the countryside	5	No, parental age has no effect	4	Yes, a younger age (up to 21) may negatively affect her mental health	1	Improved access to specialist care (more therapists, shorter waiting times), Improved affordability of mental health care	Often
20	1	Moravian-Silesian Region	3	0	2	Economic conditions (e.g., financial stress), Academic or work pressure, Loneliness and social isolation	Mixed (on the outskirts of a city or in small towns)	Greater social and cultural opportunities (cinemas, concerts, cafés, etc.), Accessibility of employment and education, Opportunity to establish more social contacts		A combination of urban and rural living	3	No, parental age has no effect	1	No, maternal age has no effect on her mental health	1	Improving the affordability of mental health care, Greater support in schools and universities	Often
22	1	Zlín Region	3	0	2	Economic conditions (e.g., financial stress), Academic or work pressure, Loneliness and social isolation	Mixed (on the outskirts of a city or in small towns)	Easier access to medical and mental health care, Greater social and cultural opportunities (cinemas, concerts, cafés, etc.), Better public transport accessibility	Higher levels of stress and pace of life, More noise and pollution, Lack of nature and quiet places	In the countryside	5	Yes, younger parental age improves children's mental health	2	Yes, a younger age (up to 21) may negatively affect her mental health	1	Raising awareness of mental health through campaigns, Greater support in schools and universities	Often
25	1	Moravian-Silesian Region	4	0	2	Economic conditions (e.g., financial stress), Academic or work pressure, Loneliness and social isolation	Mixed (on the outskirts of a city or in small towns)	Greater social and cultural opportunities (cinemas, concerts, cafés, etc.), better access to public transport, opportunity to make more social contacts	Higher levels of stress and pace of life, More noise and pollution, Lack of nature and quiet places, Greater anonymity and feelings of loneliness, Greater competitive pressure in the job market	A combination of urban and rural living	4	I don't know	2	Yes, a younger age (up to 21) may negatively affect her mental health	2	Improving the affordability of mental health care, Greater support in schools and universities	Always
26	2	Moravian-Silesian Region	3	0	0	Economic conditions (e.g., financial stress), Academic or work pressure, Loneliness and social isolation	Urban environment	Easier access to medical and mental health care, Accessibility of employment and education, Greater anonymity and privacy	Higher levels of stress and pace of life, More noise and pollution, Greater competitive pressure in the job market	In the countryside	3	Yes, older parental age improves children's mental health	1	Yes, a younger age (up to 21) may negatively affect her mental health	3	Improving the affordability of mental health care, Greater support in schools and universities	Always
26	2	Moravian-Silesian Region	4	0	2	Economic conditions (e.g., financial stress), Academic or work pressure, Loneliness and social isolation	Urban environment	Easier access to medical and mental health care, Greater social and cultural opportunities (cinemas, concerts, cafés, etc.), Accessibility of employment and education	Higher levels of stress and pace of life, More noise and pollution, Greater anonymity and feelings of loneliness	A combination of urban and rural living	5	Yes, older parental age improves children's mental health	1	Yes, a younger age (up to 21) may negatively affect her mental health	1	Zvýšení povědomí o duševním zdraví prostřednictvím kampaní, Vytvoření lepšího systému preventivní péče, Osvěta. Spousta jedinců především starší generace bagatelizuje psychické problémy a vůbec je nechápe jako plnohodnotnou zdravotní obtíž.	Never
19	2	Moravian-Silesian Region	2	0	0	Economic conditions (e.g., financial stress), Academic or work pressure, Loneliness and social isolation	Urban environment	Easier access to medical and mental health care, Greater anonymity and privacy, Better public transport accessibility	Lack of nature and quiet places, Higher crime rate, Greater competitive pressure in the labor market	A combination of urban and rural living	4	No, parental age has no effect	4	Yes, a younger age (up to 21) may negatively affect her mental health	3	Improving the affordability of mental health care, Creating a better preventive care system	Always
19	1	Moravian-Silesian Region	2	0	0	Economic conditions (e.g., financial stress), Academic or work pressure, Loneliness and social isolation	Urban environment	Accessibility of employment and education, Greater anonymity and privacy, Better accessibility of public transport	More noise and pollution, Lack of nature and quiet places, Greater competitive pressure in the job market	A combination of urban and rural living	3	Yes, older parental age improves children's mental health	2	Yes, a younger age (up to 21) may negatively affect her mental health	2	Improving access to professional care (more therapists, shorter waiting times), Raising awareness of mental health through campaigns, Greater support in schools and universities	Occasionally
19	2	Moravian-Silesian Region	5	0	0	Economic conditions (e.g., financial stress), Academic or work pressure, Loneliness and social isolation	Mixed (on the outskirts of a city or in small towns)			In the countryside	4	No, parental age has no effect	2	Yes, a younger age (up to 21) may negatively affect her mental health	3	Greater support in schools and universities, Creation of a better preventive care system	Never
21	2	Zlín Region	4	0	2	Economic conditions (e.g., financial stress), Academic or work pressure, Loneliness and social isolation	Rural environment	Greater social and cultural opportunities (cinemas, concerts, cafés, etc.), Greater anonymity and privacy, Nature	Higher levels of stress and pace of life, More noise and pollution, Lack of nature and quiet places	In the countryside	5	I don't know	1	Yes, an older age (over 21) may positively affect her mental health	1	Greater support in schools and universities, Creation of a better preventive care system	Always
24	1	Moravian-Silesian Region	3	0	0	Economic conditions (e.g., financial stress), Academic or work pressure, Loneliness and social isolation	Urban environment	Greater social and cultural opportunities (cinemas, concerts, cafés, etc.), better access to public transport, opportunity to make more social contacts	Higher levels of stress and pace of life, Higher crime rates, Greater competitive pressure in the labor market	A combination of urban and rural living	4	I don't know	2	I don't know	2	Improving access to specialist care (more therapists, shorter waiting times), creating a better preventive care system	Often
23	1	Moravian-Silesian Region	3	0	0	Economic conditions (e.g., financial stress), Academic or work pressure, Loneliness and social isolation	Rural environment	Easier access to medical and mental health care, Accessibility of employment and education, Better accessibility of public transport	Lack of nature and quiet places, Greater anonymity and feelings of loneliness, Higher crime rates	In the countryside	5	Yes, younger parental age improves children's mental health	2	Yes, a younger age (up to 21) may negatively affect her mental health	4	Improved access to specialist care (more therapists, shorter waiting times), Improved affordability of mental health care	Occasionally
19	2	Moravian-Silesian Region	4	0	3	Economic conditions (e.g., financial stress), Academic or work pressure, Loneliness and social isolation	Rural environment	Greater social and cultural opportunities (cinemas, concerts, cafés, etc.), Accessibility of employment and education, Opportunity to establish more social contacts	Higher levels of stress and pace of life, More noise and pollution, Lack of nature and quiet places	In the countryside	3	No, parental age has no effect	1	Yes, a younger age (up to 21) may negatively affect her mental health	4	Improving the affordability of mental health care, Greater support in schools and universities	Often
23	2	Zlín Region	3	0	0	Economic conditions (e.g., financial stress), Academic or work pressure, Loneliness and social isolation	Mixed (on the outskirts of a city or in small towns)	Accessibility of employment and education, Better accessibility of public transport	Higher levels of stress and pace of life, More noise and pollution, Lack of nature and quiet places	In the countryside	5	No, parental age has no effect	1	I don't know	2	Raising awareness of mental health through campaigns, Greater support in schools and universities	Often
21	1	Moravian-Silesian Region	3	0	0	Economic conditions (e.g., financial stress), Academic or work pressure, Loneliness and social isolation	Urban environment	Greater social and cultural opportunities (cinemas, concerts, cafés, etc.), Accessibility of employment and education, Opportunity to establish more social contacts	Higher levels of stress and pace of life, More noise and pollution, Lack of nature and quiet places	A combination of urban and rural living	5	I don't know	3	Yes, a younger age (up to 21) may negatively affect her mental health	1	Raising awareness of mental health through campaigns, Greater support in schools and universities	Occasionally
19	2	Moravian-Silesian Region	3	0	0	Economic conditions (e.g., financial stress), Academic or work pressure, Loneliness and social isolation	Urban environment	Greater social and cultural opportunities (cinemas, concerts, cafés, etc.), Accessibility of employment and education, Better accessibility of public transport	More noise and pollution, Lack of nature and quiet places, Greater competitive pressure in the job market	A combination of urban and rural living	4	Yes, older parental age improves children's mental health	2	Yes, a younger age (up to 21) may negatively affect her mental health	3	Improving the affordability of mental health care, Creating a better preventive care system	Often
23	2	Moravian-Silesian Region	3	3	1	Economic conditions (e.g., financial stress), Academic or work pressure, Loneliness and social isolation	Mixed (on the outskirts of a city or in small towns)	Accessibility of employment and education, Better accessibility of public transport, Opportunity to establish more social contacts		A combination of urban and rural living	5	Yes, younger parental age improves children's mental health	2	I don't know	2	Improved access to professional care (more therapists, shorter waiting times), Greater support in schools and universities	Always
26	1	Olomouc Region	4	0	1	Economic conditions (e.g., financial stress), Academic or work pressure, Loneliness and social isolation	Urban environment	Easier access to medical and mental health care, Greater social and cultural opportunities (cinemas, concerts, cafés, etc.), Opportunity to establish more social contacts	Higher levels of stress and pace of life, More noise and pollution, Greater competitive pressure in the job market	A combination of urban and rural living	5	Yes, older parental age improves children's mental health	3	Yes, a younger age (up to 21) may negatively affect her mental health	1	Improved access to specialist care (more therapists, shorter waiting times), Improved affordability of mental health care	Always
24	1	Moravian-Silesian Region	3	0	0	Economic conditions (e.g., financial stress), Academic or work pressure, Loneliness and social isolation	Urban environment	Easier access to medical and mental health care, Greater social and cultural opportunities (cinemas, concerts, cafés, etc.), Accessibility of employment and education	Higher levels of stress and pace of life, More noise and pollution, Greater competitive pressure in the job market	A combination of urban and rural living	5	I don't know	3	Yes, a younger age (up to 21) may negatively affect her mental health	2	Improving the affordability of mental health care, Greater support in schools and universities	Occasionally
22	1	Moravian-Silesian Region	3	0	0	Economic conditions (e.g., financial stress), Academic or work pressure, Loneliness and social isolation	Urban environment	Greater social and cultural opportunities (cinemas, concerts, cafés, etc.), Accessibility of employment and education, Better accessibility of public transport	Higher levels of stress and pace of life, More noise and pollution, Higher crime rates	A combination of urban and rural living	2	No, parental age has no effect	2	Yes, a younger age (up to 21) may negatively affect her mental health	1	Improved access to specialist care (more therapists, shorter waiting times), Improved affordability of mental health care	Often
20	1	Moravian-Silesian Region	3	0	0	Economic conditions (e.g., financial stress), Academic or work pressure, Loneliness and social isolation	Rural environment	Greater social and cultural opportunities (cinemas, concerts, cafés, etc.), Accessibility of employment and education, Better accessibility of public transport	More noise and pollution, Lack of nature and quiet places, Higher crime rate	A combination of urban and rural living	4	I don't know	3	Yes, a younger age (up to 21) may negatively affect her mental health	3	Improving the affordability of mental health care, Creating a better preventive care system	Always

Age	Gender	Region of Permanent Residence:	Q1. How would you rate your current mental state? (1 = Very poor, 5 = Very good)	Q2. If you sought professional help for your mental health (e.g., psychologist, psychiatrist), how would you rate the quality of these services? (1 = Very dissatisfied, 5 = Very satisfied)	Q3. Do you think mental health care is easily accessible in your city/region?	Q4: Which factors do you think affect the mental health of young people aged 18–25 the most? (select the 3 most important)	Q5. Do you live in an urban or rural environment?	Q6. What benefits of living in the city do you think have a positive impact on mental health? (select the 3 most important)	Q7. What disadvantages of living in the city do you think deteriorate mental health? (select the 3 most important)	Q8. If you could choose, where would you prefer to live in terms of mental health?	Q9. How important is support from family and friends to you in dealing with mental health problems? (1 = Not important, 5 = Very important)	Q10. Do you think the age at which people enter parenthood can affect the mental health of their children?	Q11. How much of an impact do you think crime in your region has on your mental health? (1 = No effect, 5 = Very strong effect)	Q12. Do you think the age of the first-time mother can affect her mental health?	Q13.How would you rate the level of general awareness and education about mental health in secondary schools and colleges? (1 = Very poor, 5 = Very good)	Q14. Which of the following do you think would be most helpful in improving mental health care for young people in your region? (select up to 2 options)	Q15. How often do you feel you have to look after your mental health on your own, without the help of professionals or family?
20	2	Moravian-Silesian Region	3	0	0	Economic conditions (e.g., financial stress), Academic or work pressure, Loneliness and social isolation	Urban environment	Greater social and cultural opportunities (cinemas, concerts, cafés, etc.), Greater anonymity and privacy, Better access to public transport	Higher levels of stress and pace of life, More noise and pollution, Greater competitive pressure in the job market	A combination of urban and rural living	2	Yes, older parental age improves children's mental health	2	Yes, a younger age (up to 21) may negatively affect her mental health	2	Improving the affordability of mental health care, Greater support in schools and universities, Creating a better preventive care system	Often
23	1	Moravian-Silesian Region	4	0	0	Economic conditions (e.g., financial stress), Academic or work pressure, Loneliness and social isolation	Rural environment	Greater social and cultural opportunities (cinemas, concerts, cafés, etc.), Accessibility of employment and education, Greater anonymity and privacy	Higher levels of stress and pace of life, More noise and pollution, Lack of nature and quiet places	In the countryside	5	Yes, younger parental age improves children's mental health	4	Yes, a younger age (up to 21) may negatively affect her mental health	1	Raising awareness of mental health through campaigns, Greater support in schools and universities	Occasionally
25	2	Olomouc Region	5	0	0	Economic conditions (e.g., financial stress), Academic or work pressure, Loneliness and social isolation	Urban environment	Easier access to medical and mental health care, Greater social and cultural opportunities (cinemas, concerts, cafés, etc.), Accessibility of employment and education	Higher levels of stress and pace of life, More noise and pollution, Higher crime rates	In the countryside	5	Yes, older parental age improves children's mental health	3	Yes, a younger age (up to 21) may negatively affect her mental health	3	Improving access to professional care (more therapists, shorter waiting times), Raising awareness of mental health through campaigns	Never
22	2	Moravian-Silesian Region	4	0	0	Economic conditions (e.g., financial stress), Academic or work pressure, Loneliness and social isolation	Rural environment	Easier access to medical and mental health care, Greater social and cultural opportunities (cinemas, concerts, cafés, etc.), Better public transport accessibility	Higher levels of stress and pace of life, More noise and pollution, Lack of nature and quiet places	In the countryside	4	Yes, older parental age improves children's mental health	2	Yes, a younger age (up to 21) may negatively affect her mental health	3	Improving the affordability of mental health care, Greater support in schools and universities	Often
26	1	Olomouc Region	3	4	1	Economic conditions (e.g., financial stress), Academic or work pressure, Loneliness and social isolation	Mixed (on the outskirts of a city or in small towns)	Greater social and cultural opportunities (cinemas, concerts, cafés, etc.), Accessibility of employment and education, Greater anonymity and privacy	Higher levels of stress and pace of life, Lack of nature and quiet places, Greater competitive pressure in the job market	A combination of urban and rural living	5	Yes, younger parental age improves children's mental health	3	Yes, an older age (over 21) may positively affect her mental health	2	Raising awareness of mental health through campaigns, Creating a better preventive care system	Often
22	2	Liberecký kraj	3	2	1	Economic conditions (e.g., financial stress), Academic or work pressure, Loneliness and social isolation	Urban environment	Greater social and cultural opportunities (cinemas, concerts, cafés, etc.), Accessibility of employment and education, Greater anonymity and privacy	Higher levels of stress and pace of life, More noise and pollution, Lack of nature and quiet places	In the countryside	2	No, parental age has no effect	1	No, maternal age has no effect on her mental health	1	Improved access to specialist care (more therapists, shorter waiting times), Improved affordability of mental health care	Occasionally
22	2	South Moravian Region	3	0	2	Economic conditions (e.g., financial stress), Academic or work pressure, Loneliness and social isolation	Mixed (on the outskirts of a city or in small towns)	Greater social and cultural opportunities (cinemas, concerts, cafés, etc.), Accessibility of employment and education, Opportunity to establish more social contacts	More noise and pollution, Lack of nature and quiet places, Greater anonymity and feelings of loneliness	In the countryside	5	Yes, younger parental age improves children's mental health	1	I don't know	3	Improving the affordability of mental health care, Creating a better preventive care system	Often
21	1	Vysočina Region	2	0	2	Economic conditions (e.g., financial stress), Academic or work pressure, Loneliness and social isolation	Mixed (on the outskirts of a city or in small towns)	Easier access to medical and mental health care, Accessibility of employment and education	Higher levels of stress and pace of life, Lack of nature and quiet places, Greater anonymity and feelings of loneliness	In a city	4	I don't know	1	Yes, a younger age (up to 21) may negatively affect her mental health	2	Improving access to specialist care (more therapists, shorter waiting times), creating a better preventive care system	Often
20	2	South Moravian Region	2	0	0	Economic conditions (e.g., financial stress), Academic or work pressure, Loneliness and social isolation	Mixed (on the outskirts of a city or in small towns)		Higher levels of stress and pace of life, More noise and pollution, Lack of nature and quiet places	In the countryside	4	I don't know	2	Yes, a younger age (up to 21) may negatively affect her mental health	2	Improving the affordability of mental health care, Greater support in schools and universities	Often
24	1	South Moravian Region	3	4	2	Economic conditions (e.g., financial stress), Academic or work pressure, Loneliness and social isolation	Urban environment	Easier access to medical and mental health care, Greater social and cultural opportunities (cinemas, concerts, cafés, etc.), Opportunity to establish more social contacts	Higher levels of stress and pace of life, Greater anonymity and feelings of loneliness, Greater competitive pressure in the job market	A combination of urban and rural living	5	No, parental age has no effect	1	Yes, a younger age (up to 21) may negatively affect her mental health	3	Improved access to professional care (more therapists, shorter waiting times), Greater support in schools and universities	Occasionally
20	1	South Moravian Region	4	5	2	Economic conditions (e.g., financial stress), Academic or work pressure, Loneliness and social isolation	Mixed (on the outskirts of a city or in small towns)	Accessibility of employment and education, Greater anonymity and privacy, Better accessibility of public transport		In the countryside	3	Yes, younger parental age improves children's mental health	1	Yes, an older age (over 21) may positively affect her mental health	2	Improved access to professional care (more therapists, shorter waiting times), Greater support in schools and universities	Occasionally
21	1	South Moravian Region	3	0	0	Economic conditions (e.g., financial stress), Academic or work pressure, Loneliness and social isolation	Rural environment		More noise and pollution, Lack of nature and quiet places, Greater competitive pressure in the job market	A combination of urban and rural living	3	I don't know	1	I don't know	2	Improved access to specialist care (more therapists, shorter waiting times), Improved affordability of mental health care	Always
22	2	South Moravian Region	2	0	2	Economic conditions (e.g., financial stress), Academic or work pressure, Loneliness and social isolation	Mixed (on the outskirts of a city or in small towns)	Greater social and cultural opportunities (cinemas, concerts, cafés, etc.), better access to public transport, opportunity to make more social contacts		In a city	4	I don't know	1	Yes, a younger age (up to 21) may negatively affect her mental health	1	Improved access to specialist care (more therapists, shorter waiting times), Improved affordability of mental health care	Always
20	2	Moravian-Silesian Region	4	3	1	Economic conditions (e.g., financial stress), Academic or work pressure, Loneliness and social isolation	Mixed (on the outskirts of a city or in small towns)	Easier access to medical and mental health care, Greater social and cultural opportunities (cinemas, concerts, cafés, etc.), Better public transport accessibility	Higher levels of stress and pace of life, More noise and pollution, Lack of nature and quiet places	In the countryside	5	I don't know	2	Yes, a younger age (up to 21) may negatively affect her mental health	2	Greater support in schools and universities, Creation of a better preventive care system	Occasionally
21	2	Moravian-Silesian Region	3	0	0	Economic conditions (e.g., financial stress), Academic or work pressure, Loneliness and social isolation	Urban environment	Greater social and cultural opportunities (cinemas, concerts, cafés, etc.), Accessibility of employment and education, Better accessibility of public transport	Higher levels of stress and pace of life, More noise and pollution, Higher crime rates	A combination of urban and rural living	4	I don't know	3	I don't know	2	Improving access to specialist care (more therapists, shorter waiting times), creating a better preventive care system	Often
24	1	Central Bohemian Region	3	0	2	Economic conditions (e.g., financial stress), Academic or work pressure, Loneliness and social isolation	Mixed (on the outskirts of a city or in small towns)	Easier access to medical and mental health care, Greater social and cultural opportunities (cinemas, concerts, cafés, etc.), Accessibility of employment and education, Better accessibility of public transport, Opportunity to establish more social contacts	Higher levels of stress and	A combination of urban and rural living	4	Yes, older parental age improves children's mental health	1	Yes, a younger age (up to 21) may negatively affect her mental health	3	Improved access to specialist care (more therapists, shorter waiting times), Improved affordability of mental health care	Often
23	1	Moravian-Silesian Region	2	3	1	Economic conditions (e.g., financial stress), academic or work pressure, loneliness and social isolation, romantic relationships	Mixed (on the outskirts of a city or in small towns)	Easier access to medical and mental health care, Accessibility of employment and education, Opportunity to establish more social contacts	Higher levels of stress and pace of life, Greater anonymity and feelings of loneliness, Greater competitive pressure in the job market	A combination of urban and rural living	4	I don't know	2	Yes, a younger age (up to 21) may negatively affect her mental health	1	Improved access to professional care (more therapists, shorter waiting times), Greater support in schools and universities	Often
20	2	Olomouc Region	2	2	1	Economic conditions (e.g., financial stress), Academic or work pressure, Loneliness and social isolation, Inadequate access to healthcare	Urban environment			In a city	4	No, parental age has no effect	4	No, maternal age has no effect on her mental health	1	Improved access to specialist care (more therapists, shorter waiting times), improved affordability of mental health care, greater support in schools and universities, creation of a better preventive care system.	Often
25	2	Central Bohemian Region	3	4	2	Economic conditions (e.g., financial stress), Academic or work pressure, Family relationships	Mixed (on the outskirts of a city or in small towns)	Easier access to medical and mental health care, Accessibility of employment and education, Better accessibility of public transport	Higher levels of stress and pace of life, More noise and pollution, Higher crime rates	A combination of urban and rural living	4	I don't know	1	I don't know	3	Greater support in schools and universities, Creation of a better preventive care system	Occasionally
23	1	Capital City of Prague	4	5	3	Economic conditions (e.g., financial stress), Academic or work pressure, Family relationships	Urban environment	Easier access to medical and mental health care, Greater social and cultural opportunities (cinemas, concerts, cafés, etc.), Accessibility of employment and education, Greater anonymity and privacy, Opportunity to establish more social contacts	Higher levels of stress and pace of life, greater competitive pressure in the labor market	A combination of urban and rural living	5	No, parental age has no effect	3	Yes, a younger age (up to 21) may negatively affect her mental health	3	Improving the affordability of mental health care, Raising awareness of mental health through campaigns, Greater support in schools and universities, Creating a better preventive care system	Occasionally
20	1	Zlín Region	3	0	2	Economic conditions (e.g., financial stress), Academic or work pressure, Family relationships	Rural environment	Greater social and cultural opportunities (cinemas, concerts, cafés, etc.), Greater anonymity and privacy, Opportunity to establish more social contacts	Higher levels of stress and pace of life, More noise and pollution, Higher crime rates	In the countryside	5	No, parental age has no effect	2	No, maternal age has no effect on her mental health	2	Improved access to professional care (more therapists, shorter waiting times), Greater support in schools and universities	Always
19	1	Moravian-Silesian Region	3	0	0	Economic conditions (e.g., financial stress), Academic or work pressure, Family relationships	Urban environment	Greater social and cultural opportunities (cinemas, concerts, cafés, etc.), Accessibility of employment and education, Opportunity to establish more social contacts	Higher levels of stress and pace of life, More noise and pollution, Lack of nature and quiet places	A combination of urban and rural living	4	I don't know	2	Yes, a younger age (up to 21) may negatively affect her mental health	1	Raising awareness of mental health through campaigns	Always
23	2	Ústí nad Labem Region	2	0	0	Economic conditions (e.g., financial stress), Academic or work pressure, Family relationships	Rural environment	Easier access to medical and mental health care, Greater social and cultural opportunities (cinemas, concerts, cafés, etc.), Greater anonymity and privacy	Higher levels of stress and pace of life, Lack of nature and quiet places, Greater competitive pressure in the job market	A combination of urban and rural living	4	I don't know	1	Yes, a younger age (up to 21) may negatively affect her mental health	3	Improved access to professional care (more therapists, shorter waiting times), Greater support in schools and universities	Often
20	2	Moravian-Silesian Region	2	0	0	Economic conditions (e.g., financial stress), Academic or work pressure, Family relationships	Urban environment	Greater social and cultural opportunities (cinemas, concerts, cafés, etc.), Greater anonymity and privacy, Opportunity to establish more social contacts	Higher levels of stress and pace of life, More noise and pollution, Lack of nature and quiet places	In a city	4	No, parental age has no effect	2	No, maternal age has no effect on her mental health	3	Raising awareness of mental health through campaigns, Greater support in schools and universities	Often
24	1	Olomouc Region	2	4	2	Economic conditions (e.g., financial stress), Academic or work pressure, Family relationships	Rural environment	Easier access to medical and mental health care, Greater social and cultural opportunities (cinemas, concerts, cafés, etc.), Accessibility of employment and education, Better accessibility of public transport	Lack of nature and quiet places, Higher crime rate	In the countryside	5	Yes, younger parental age improves children's mental health	2	Yes, a younger age (up to 21) may negatively affect her mental health	1	Improving the affordability of mental health care, Raising awareness of mental health through campaigns, Greater support in schools and universities, Creating a better preventive care system	Often
25	1	Moravian-Silesian Region	4	4	2	Economic conditions (e.g., financial stress), Academic or work pressure, Family relationships	Mixed (on the outskirts of a city or in small towns)	Easier access to medical and mental health care, Accessibility of employment and education, Better accessibility of public transport	Higher levels of stress and pace of life, More noise and pollution, Lack of nature and quiet places	A combination of urban and rural living	5	Yes, older parental age improves children's mental health	2	I don't know	1	Improved access to professional care (more therapists, shorter waiting times), Greater support in schools and universities	Occasionally
24	2	Moravian-Silesian Region	5	0	2	Economic conditions (e.g., financial stress), Academic or work pressure, Family relationships	Urban environment	Easier access to medical and mental health care, Greater social and cultural opportunities (cinemas, concerts, cafés, etc.), Accessibility of employment and education	More noise and pollution, Lack of nature and quiet places, Greater competitive pressure in the job market	I have no proference	2	No, parental age has no effect	1	I don't know	2	Greater support in schools and universities, Creation of a better preventive care system	Always
26	1	Moravian-Silesian Region	2	0	3	Economic conditions (e.g., financial stress), Academic or work pressure, Family relationships	Urban environment	Easier access to medical and mental health care, Accessibility of employment and education, Better accessibility of public transport		In the countryside	5	Yes, younger parental age improves children's mental health	4	Yes, an older age (over 21) may positively affect her mental health	2	Improving the affordability of mental health care, Raising awareness of mental health through campaigns, Greater support in schools and universities	Often
24	1	Moravian-Silesian Region	4	0	2	Economic conditions (e.g., financial stress), Academic or work pressure, Family relationships	Rural environment	Greater social and cultural opportunities (cinemas, concerts, cafés, etc.), Accessibility of employment and education, Greater anonymity and privacy	Higher levels of stress and pace of life, More noise and pollution, Higher crime rates	A combination of urban and rural living	4	No, parental age has no effect	3	Yes, a younger age (up to 21) may negatively affect her mental health	3	Improved access to specialist care (more therapists, shorter waiting times), Improved affordability of mental health care	Often
23	1	Moravian-Silesian Region	3	5	2	Economic conditions (e.g., financial stress), Academic or work pressure, Family relationships	Urban environment	Access to employment and education, Opportunity to establish more social contacts	Greater competitive pressure in the labor market	A combination of urban and rural living	5	Yes, older parental age improves children's mental health	2	Yes, a younger age (up to 21) may negatively affect her mental health	2	Zlepšení dostupnosti odborné péče (více terapeutů, kratší čekací doby), Zvýšení povědomí o duševním zdraví prostřednictvím kampaní, Větší podpora na školách a univerzitách, Vytvoření lepšího systému preventivní péče	Occasionally
19	1	Zlín Region	3	2	1	Economic conditions (e.g., financial stress), Academic or work pressure, Family relationships	Mixed (on the outskirts of a city or in small towns)	Greater anonymity and privacy, Better access to public transport, Opportunity to establish more social contacts	Higher levels of stress and pace of life, More noise and pollution, Higher crime rates	A combination of urban and rural living	2	I don't know	3	Yes, a younger age (up to 21) may negatively affect her mental health	2	Improved access to professional care (more therapists, shorter waiting times), Greater support in schools and universities	Always
21	1	Moravian-Silesian Region	1	5	1	Economic conditions (e.g., financial stress), Academic or work pressure, Family relationships	Urban environment		Higher levels of stress and pace of life, More noise and pollution, Lack of nature and quiet places, Higher crime rates, Greater competitive pressure in the job market	A combination of urban and rural living	3	I don't know	4	Yes, a younger age (up to 21) may negatively affect her mental health	1	Improving access to specialist care (more therapists, shorter waiting times), improving the affordability of mental health care, creating a better preventive care system	Often
21	1	Olomouc Region	3	2	2	Economic conditions (e.g., financial stress), Academic or work pressure, Family relationships	Rural environment	Easier access to medical and mental health care, Greater social and cultural opportunities (cinemas, concerts, cafés, etc.)	Higher levels of stress and pace of life, Higher crime rates	In the countryside	5	I don't know	3	Yes, a younger age (up to 21) may negatively affect her mental health	2	Improved access to specialist care (more therapists, shorter waiting times), improved affordability of mental health care, greater support in schools and universities, creation of a better preventive care system.	Occasionally
23	1	Moravian-Silesian Region	5	0	0	Economic conditions (e.g., financial stress), Academic or work pressure, Family relationships	Mixed (on the outskirts of a city or in small towns)		Higher levels of stress and pace of life, More noise and pollution, Lack of nature and quiet places	A combination of urban and rural living	4	I don't know	1	Yes, an older age (over 21) may positively affect her mental health	1	Raising awareness of mental health through campaigns, Greater support in schools and universities	Often
24	2	Moravian-Silesian Region	5	0	0	Economic conditions (e.g., financial stress), Academic or work pressure, Family relationships	Rural environment	Greater social and cultural opportunities (cinemas, concerts, cafés, etc.), Accessibility of employment and education, Greater anonymity and privacy	Higher levels of stress and pace of life, Greater anonymity and feelings of loneliness, Greater competitive pressure in the job market	In the countryside	3	Yes, older parental age improves children's mental health	1	Yes, a younger age (up to 21) may negatively affect her mental health	1	Raising awareness of mental health through campaigns, Greater support in schools and universities	Always
20	1	Moravian-Silesian Region	3	0	1	Economic conditions (e.g., financial stress), Academic or work pressure, Family relationships	Mixed (on the outskirts of a city or in small towns)	Easier access to medical and mental health care, Accessibility of employment and education, Opportunity to establish more social contacts		In the countryside	4	No, parental age has no effect	4	No, maternal age has no effect on her mental health	4	Improving access to specialist care (more therapists, shorter waiting times), creating a better preventive care system	Often
22	1	Moravian-Silesian Region	3	4	2	Economic conditions (e.g., financial stress), Academic or work pressure, Family relationships	Mixed (on the outskirts of a city or in small towns)		Higher levels of stress and pace of life, More noise and pollution, Lack of nature and quiet places	A combination of urban and rural living	4	No, parental age has no effect	1	Yes, a younger age (up to 21) may negatively affect her mental health	2	Improving access to specialist care (more therapists, shorter waiting times), improving the affordability of mental health care, raising awareness of mental health through campaigns, greater support in schools and universities	Often
25	1	Moravian-Silesian Region	3	0	0	Economic conditions (e.g., financial stress), Academic or work pressure, Family relationships	Rural environment	Greater social and cultural opportunities (cinemas, concerts, cafés, etc.), Accessibility of employment and education, Better accessibility of public transport	Higher levels of stress and pace of life, More noise and pollution, Lack of nature and quiet places	In the countryside	3	I don't know	1	I don't know	1	Improving the affordability of mental health care, Greater support in schools and universities	Often
19	2	Moravian-Silesian Region	4	4	0	Economic conditions (e.g., financial stress), Academic or work pressure, Family relationships	Urban environment	Easier access to medical and mental health care, Greater social and cultural opportunities (cinemas, concerts, cafés, etc.), Accessibility of employment and education	Higher levels of stress and pace of life, Lack of nature and quiet places, Greater competitive pressure in the job market	In a city	5	Yes, older parental age improves children's mental health	2	Yes, a younger age (up to 21) may negatively affect her mental health	3	Raising awareness of mental health through campaigns	Often
20	1	Vysočina Region	1	0	0	Economic conditions (e.g., financial stress), Academic or work pressure, Family relationships	Urban environment	Greater social and cultural opportunities (cinemas, concerts, cafés, etc.), better access to public transport, opportunity to make more social contacts	Higher levels of stress and pace of life, Lack of nature and quiet places, Greater competitive pressure in the job market	A combination of urban and rural living	5	No, parental age has no effect	1	Yes, a younger age (up to 21) may negatively affect her mental health	5	Greater support in schools and universities, Creation of a better preventive care system	Often
24	2	Moravian-Silesian Region	5	1	3	Economic conditions (e.g., financial stress), Academic or work pressure, Family relationships	Urban environment	Easier access to medical and mental health care, Greater social and cultural opportunities (cinemas, concerts, cafés, etc.), Accessibility of employment and education	Higher levels of stress and pace of life, greater anonymity and feelings of loneliness	In the countryside	3	No, parental age has no effect	1	Yes, an older age (over 21) may positively affect her mental health	5	Greater support in schools and universities, Creation of a better preventive care system	Always
19	2	Moravian-Silesian Region	4	0	2	Economic conditions (e.g., financial stress), Academic or work pressure, Family relationships	Mixed (on the outskirts of a city or in small towns)	Greater social and cultural opportunities (cinemas, concerts, cafés, etc.), Greater anonymity and privacy, Better access to public transport	Higher levels of stress and pace of life, More noise and pollution, Lack of nature and quiet places, Higher crime rates	In the countryside	5	Yes, older parental age improves children's mental health	4	I don't know	4	Improving the affordability of mental health care, Greater support in schools and universities	Often
23	1	Moravian-Silesian Region	3	1	0	Economic conditions (e.g., financial stress), Academic or work pressure, Family relationships	Urban environment	Easier access to medical and mental health care, Greater social and cultural opportunities (cinemas, concerts, cafés, etc.), Greater anonymity and privacy	Higher levels of stress and pace of life, More noise and pollution, Lack of nature and quiet places	In a city	3	I don't know	4	Yes, a younger age (up to 21) may negatively affect her mental health	2	Greater support in schools and universities, Creation of a better preventive care system	Often
26	1	Olomouc Region	2	0	0	Economic conditions (e.g., financial stress), Academic or work pressure, Family relationships	Rural environment	Greater social and cultural opportunities (cinemas, concerts, cafés, etc.), Accessibility of employment and education, Opportunity to establish more social contacts	More noise and pollution, Lack of nature and quiet places, Higher crime rate	In the countryside	1	Yes, younger parental age improves children's mental health	2	No, maternal age has no effect on her mental health	2	Raising awareness of mental health through campaigns	Occasionally
22	1	Moravian-Silesian Region	5	0	3	Economic conditions (e.g., financial stress), Academic or work pressure, Family relationships	Urban environment	Easier access to medical and mental health care, Greater social and cultural opportunities (cinemas, concerts, cafés, etc.), Opportunity to establish more social contacts	Higher levels of stress and pace of life, More noise and pollution, Lack of nature and quiet places	A combination of urban and rural living	5	I don't know	3	Yes, a younger age (up to 21) may negatively affect her mental health	4	Improving the affordability of mental health care, Greater support in schools and universities	Never
22	2	Moravian-Silesian Region	4	0	0	Economic conditions (e.g., financial stress), Academic or work pressure, Family relationships	Rural environment	Accessibility of employment and education, Better accessibility of public transport, Opportunity to establish more social contacts	Lack of nature and quiet places, Higher crime rate, Greater competitive pressure in the labor market	In the countryside	5	I don't know	3	Yes, an older age (over 21) may positively affect her mental health	4	Improving the affordability of mental health care, Greater support in schools and universities	Never
24	2	Olomouc Region	3	5	2	Economic conditions (e.g., financial stress), Academic or work pressure, Family relationships	Rural environment	Easier access to medical and mental health care, Greater social and cultural opportunities (cinemas, concerts, cafés, etc.), Accessibility of employment and education	Higher levels of stress and pace of life, More noise and pollution, Lack of nature and quiet places	In the countryside	5	Yes, older parental age improves children's mental health	5	Yes, a younger age (up to 21) may negatively affect her mental health	2	Improving access to professional care (more therapists, shorter waiting times), Raising awareness of mental health through campaigns	Always
23	1	Moravian-Silesian Region	3	0	1	Economic conditions (e.g., financial stress), Academic or work pressure, Family relationships	Urban environment	Greater social and cultural opportunities (cinemas, concerts, cafés, etc.), Accessibility of employment and education, Opportunity to establish more social contacts	More noise and pollution, Lack of nature and quiet places, Greater competitive pressure in the job market	In a city	4	No, parental age has no effect	1	No, maternal age has no effect on her mental health	1	Greater support in schools and universities, Creation of a better preventive care system	Often

Age	Gender	Region of Permanent Residence:	Q1. How would you rate your current mental state? (1 = Very poor, 5 = Very good)	Q2. If you sought professional help for your mental health (e.g., psychologist, psychiatrist), how would you rate the quality of these services? (1 = Very dissatisfied, 5 = Very satisfied)	Q3. Do you think mental health care is easily accessible in your city/region?	Q4: Which factors do you think affect the mental health of young people aged 18–25 the most? (select the 3 most important)	Q5. Do you live in an urban or rural environment?	Q6. What benefits of living in the city do you think have a positive impact on mental health? (select the 3 most important)	Q7. What disadvantages of living in the city do you think deteriorate mental health? (select the 3 most important)	Q8. If you could choose, where would you prefer to live in terms of mental health?	Q9. How important is support from family and friends to you in dealing with mental health problems? (1 = Not important, 5 = Very important)	Q10. Do you think the age at which people enter parenthood can affect the mental health of their children?	Q11. How much of an impact do you think crime in your region has on your mental health? (1 = No effect, 5 = Very strong effect)	Q12. Do you think the age of the first-time mother can affect her mental health?	Q13.How would you rate the level of general awareness and education about mental health in secondary schools and colleges? (1 = Very poor, 5 = Very good)	Q14. Which of the following do you think would be most helpful in improving mental health care for young people in your region? (select up to 2 options)	Q15. How often do you feel you have to look after your mental health on your own, without the help of professionals or family?
23	1	Moravian-Silesian Region	3	0	0	Economic conditions (e.g., financial stress), Academic or work pressure, Family relationships	Rural environment	Greater social and cultural opportunities (cinemas, concerts, cafés, etc.), Accessibility of employment and education		In the countryside	5	No, parental age has no effect	1	No, maternal age has no effect on her mental health	3	Improving the affordability of mental health care, Raising awareness of mental health through campaigns, Creating a better preventive care system	Occasionally
22	1	Olomouc Region	5	0	3	Economic conditions (e.g., financial stress), Academic or work pressure, Family relationships	Urban environment	Accessibility of employment and education, Better accessibility of public transport, Opportunity to establish more social contacts	Higher levels of stress and pace of life, More noise and pollution, Greater competitive pressure in the job market	In a city	5	Yes, younger parental age improves children's mental health	1	No, maternal age has no effect on her mental health	2	Improved access to specialist care (more therapists, shorter waiting times), Improved affordability of mental health care	Occasionally
20	1	Zlín Region	3	0	0	Economic conditions (e.g., financial stress), Academic or work pressure, Family relationships	Rural environment	Easier access to medical and mental health care, Greater social and cultural opportunities (cinemas, concerts, cafés, etc.), Opportunity to establish more social contacts	Higher levels of stress and pace of life, More noise and pollution, Greater competitive pressure in the job market	In the countryside	3	I don't know	1	Yes, a younger age (up to 21) may negatively affect her mental health	2	Raising awareness of mental health through campaigns, Greater support in schools and universities	Occasionally
24	1	South Moravian Region	4	0	2	Economic conditions (e.g., financial stress), Academic or work pressure, Family relationships	Rural environment	Accessibility of employment and education, Greater anonymity and privacy, Better accessibility of public transport	Higher levels of stress and pace of life, More noise and pollution, Lack of nature and quiet places, Higher crime rates	In the countryside	5	No, parental age has no effect	1	Yes, a younger age (up to 21) may negatively affect her mental health	3	Raising awareness of mental health through campaigns, Greater support in schools and universities	Often
22	2	Moravian-Silesian Region	3	0	2	Economic conditions (e.g., financial stress), Academic or work pressure, Family relationships	Urban environment		Higher levels of stress and pace of life, Lack of nature and quiet places, Greater competitive pressure in the job market	In a city	4	No, parental age has no effect	2	No, maternal age has no effect on her mental health	4	Creating a better preventive care system	Often
20	1	Moravian-Silesian Region	5	0	0	Economic conditions (e.g., financial stress), Academic or work pressure, Family relationships	Rural environment	Greater social and cultural opportunities (cinemas, concerts, cafés, etc.), Accessibility of employment and education, Better accessibility of public transport	More noise and pollution, Lack of nature and quiet places, Higher crime rate	A combination of urban and rural living	5	Yes, younger parental age improves children's mental health	3	Yes, a younger age (up to 21) may negatively affect her mental health	3	Improving the affordability of mental health care, Greater support in schools and universities	Never
22	1	Central Bohemian Region	4	0	0	Economic conditions (e.g., financial stress), Academic or work pressure, Family relationships	Urban environment	Easier access to medical and mental health care, Greater social and cultural opportunities (cinemas, concerts, cafés, etc.), Better public transport accessibility	Higher levels of stress and pace of life, More noise and pollution, Greater competitive pressure in the job market	I have no proference	5	I don't know	1	I don't know	2	Greater support in schools and universities, Creation of a better preventive care system	Often
20	1	Hradec Králové Region	3	0	2	Economic conditions (e.g., financial stress), Academic or work pressure, Family relationships	Mixed (on the outskirts of a city or in small towns)	Easier access to medical and mental health care, Greater social and cultural opportunities (cinemas, concerts, cafés, etc.), Opportunity to establish more social contacts	Higher levels of stress and pace of life, Lack of nature and quiet places, Greater competitive pressure in the job market	In the countryside	4	Yes, older parental age improves children's mental health	3	Yes, a younger age (up to 21) may negatively affect her mental health	1	Greater support in schools and universities, Creation of a better preventive care system	Often
20	1	Moravian-Silesian Region	3	0	2	Economic conditions (e.g., financial stress), Academic or work pressure, Family relationships	Mixed (on the outskirts of a city or in small towns)	Access to employment and education	More noise and pollution	In the countryside	4	No, parental age has no effect	3	Yes, a younger age (up to 21) may negatively affect her mental health	2	Improving the affordability of mental health care, Raising awareness of mental health through campaigns, Greater support in schools and universities	Occasionally
21	2	Moravian-Silesian Region	4	0	2	Economic conditions (e.g., financial stress), Academic or work pressure, Family relationships	Rural environment	Easier access to medical and mental health care, Greater social and cultural opportunities (cinemas, concerts, cafés, etc.), Accessibility of employment and education, Greater anonymity and privacy, Better accessibility of public transport, Opportunity to establish more social contacts	More noise and pollution, Lack of nature and quiet places, Higher crime rate	In the countryside	5	I don't know	2	Yes, a younger age (up to 21) may negatively affect her mental health	2	Improved access to specialist care (more therapists, shorter waiting times), Improved affordability of mental health care	Always
21	2	Pilsen Region	3	0	0	Economic conditions (e.g., financial stress), Academic or work pressure, Family relationships	Urban environment	Easier access to medical and mental health care, Greater social and cultural opportunities (cinemas, concerts, cafés, etc.), Accessibility of employment and education	Higher levels of stress and pace of life, More noise and pollution, Higher crime rates	A combination of urban and rural living	4	Yes, younger parental age improves children's mental health	3	Yes, a younger age (up to 21) may negatively affect her mental health	2	Improving the affordability of mental health care, Greater support in schools and universities	Often
20	1	Moravian-Silesian Region	3	0	0	Economic conditions (e.g., financial stress), Academic or work pressure, Family relationships	Urban environment	Greater social and cultural opportunities (cinemas, concerts, cafés, etc.), Accessibility of employment and education, Better accessibility of public transport	Higher levels of stress and pace of life, More noise and pollution, Lack of nature and quiet places	A combination of urban and rural living	4	No, parental age has no effect	2	Yes, a younger age (up to 21) may negatively affect her mental health	1	Improving the affordability of mental health care, Raising awareness of mental health through campaigns, Creating a better preventive care system	Occasionally
24	2	Moravian-Silesian Region	4	0	0	Economic conditions (e.g., financial stress), Academic or work pressure, Family relationships	Urban environment	-	Higher levels of stress and	A combination of urban and rural living	4	I don't know	2	I don't know	4	Improving the affordability of mental health care, Greater support in schools and universities	Never
21	2	South Moravian Region	3	0	0	Economic conditions (e.g., financial stress), Academic or work pressure, Family relationships	Rural environment	Easier access to medical and mental health care, Accessibility of employment and education, Opportunity to establish more social contacts	Higher levels of stress and pace of life, Lack of nature and quiet places, Greater competitive pressure in the job market	A combination of urban and rural living	3	Yes, younger parental age improves children's mental health	3	Yes, a younger age (up to 21) may negatively affect her mental health	1	Improved access to professional care (more therapists, shorter waiting times), Greater support in schools and universities	Often
23	2	Olomouc Region	3	0	0	Economic conditions (e.g., financial stress), Academic or work pressure, Family relationships	Rural environment		Higher levels of stress and pace of life, More noise and pollution	In the countryside	1	Yes, older parental age improves children's mental health	1	Yes, an older age (over 21) may positively affect her mental health	2	Improving the affordability of mental health care, Raising awareness of mental health through campaigns	Never
22	2	Moravian-Silesian Region	3	0	2	Economic conditions (e.g., financial stress), Academic or work pressure, Family relationships	Mixed (on the outskirts of a city or in small towns)	Easier access to medical and mental health care, Greater social and cultural opportunities (cinemas, concerts, cafés, etc.), Accessibility of employment and education	Lack of nature and quiet places, Greater anonymity and feelings of loneliness, Higher crime rates	In the countryside	4	I don't know	1	I don't know	2	Improved access to professional care (more therapists, shorter waiting times), Greater support in schools and universities	Often
21	1	Moravian-Silesian Region	4	0	1	Economic conditions (e.g., financial stress), Academic or work pressure, Family relationships	Mixed (on the outskirts of a city or in small towns)	Greater social and cultural opportunities (cinemas, concerts, cafés, etc.), Accessibility of employment and education, Opportunity to establish more social contacts	Higher levels of stress and pace of life, More noise and pollution, Lack of nature and quiet places	A combination of urban and rural living	5	No, parental age has no effect	1	No, maternal age has no effect on her mental health	2	Improving access to specialist care (more therapists, shorter waiting times), improving the affordability of mental health care, creating a better preventive care system	Never
20	1	Olomouc Region	4	0	0	Economic conditions (e.g., financial stress), Academic or work pressure, Family relationships	Mixed (on the outskirts of a city or in small towns)	Easier access to medical and mental health care, Accessibility of employment and education, Opportunity to establish more social contacts	Higher levels of stress and pace of life, More noise and pollution, Greater competitive pressure in the job market	A combination of urban and rural living	5	Yes, older parental age improves children's mental health	3	Yes, a younger age (up to 21) may negatively affect her mental health	3	Improved access to specialist care (more therapists, shorter waiting times), Improved affordability of mental health care	Often
21	1	South Moravian Region	4	5	2	Economic conditions (e.g., financial stress), Academic or work pressure, Family relationships	Urban environment		More noise and pollution, Lack of nature and quiet places, Higher crime rate	In the countryside	5	Yes, younger parental age improves children's mental health	2	Yes, a younger age (up to 21) may negatively affect her mental health	2	Improving access to specialist care (more therapists, shorter waiting times), improving the affordability of mental health care, raising awareness of mental health through campaigns, greater support in schools and universities, creating a better preventive care system.	Often
22	2	Moravian-Silesian Region	5	0	0	Economic conditions (e.g., financial stress), Academic or work pressure, Family relationships	Rural environment	Easier access to medical and mental health care, Greater social and cultural opportunities (cinemas, concerts, cafés, etc.), Accessibility of employment and education, Better accessibility of public transport, Opportunity to establish more social contacts	Higher levels of stress and pace of life, More noise and pollution, Higher crime rates	A combination of urban and rural living	5	Yes, older parental age improves children's mental health	1	Yes, a younger age (up to 21) may negatively affect her mental health	3	Improved access to specialist care (more therapists, shorter waiting times), improved affordability of mental health care, greater support in schools and universities	Often
19	1	Liberecký kraj	3	4	3	Economic conditions (e.g., financial stress), Academic or work pressure, Family relationships	Urban environment	Easier access to medical and mental health care, Greater social and cultural opportunities (cinemas, concerts, cafés, etc.), Accessibility of employment and education	Higher levels of stress and pace of life, More noise and pollution, Lack of nature and quiet places	In the countryside	5	Yes, older parental age improves children's mental health	1	Yes, an older age (over 21) may positively affect her mental health	4	Improving the affordability of mental health care, Greater support in schools and universities	Occasionally
20	2	South Moravian Region	4	0	2	Economic conditions (e.g., financial stress), Academic or work pressure, Family relationships	Urban environment	Accessibility of employment and education, Greater anonymity and privacy, Better accessibility of public transport	Higher levels of stress and pace of life, Greater anonymity and feelings of loneliness, Greater competitive pressure in the job market	In a city	2	I don't know	2	Yes, a younger age (up to 21) may negatively affect her mental health	3	Improving access to specialist care (more therapists, shorter waiting times), creating a better preventive care system	Always
19	2	South Moravian Region	5	0	0	Economic conditions (e.g., financial stress), Academic or work pressure, Family relationships	Urban environment			In a city	5	Yes, older parental age improves children's mental health	3	Yes, a younger age (up to 21) may negatively affect her mental health	2	Improving the affordability of mental health care, Greater support in schools and universities	Never
20	1	Moravian-Silesian Region	3	5	2	Economic conditions (e.g., financial stress), Academic or work pressure, Family relationships	Urban environment	Easier access to medical and mental health care, Greater social and cultural opportunities (cinemas, concerts, cafés, etc.), Accessibility of employment and education	More noise and pollution, Lack of nature and quiet places, Higher crime rate	In a city	4	No, parental age has no effect	2	Yes, an older age (over 21) may positively affect her mental health	3	Improving access to specialist care (more therapists, shorter waiting times), creating a better preventive care system	Often
19	1	South Moravian Region	4	0	0	Economic conditions (e.g., financial stress), Academic or work pressure, Family relationships	Urban environment	Greater social and cultural opportunities (cinemas, concerts, cafés, etc.), Accessibility of employment and education, Better accessibility of public transport	Higher levels of stress and pace of life, Lack of nature and quiet places, Greater competitive pressure in the job market	A combination of urban and rural living	4	I don't know	2	I don't know	5	Creating a better preventive care system	Often
20	1	Pardubice Region	3	0	0	Economic conditions (e.g., financial stress), Academic or work pressure, Family relationships	Rural environment	Accessibility of employment and education, Greater anonymity and privacy, Better accessibility of public transport	Higher levels of stress and pace of life, More noise and pollution, Lack of nature and quiet places	In the countryside	2	Yes, older parental age improves children's mental health	1	Yes, a younger age (up to 21) may negatively affect her mental health	3	Improving the affordability of mental health care, Greater support in schools and universities	Always
24	2	Moravian-Silesian Region	4	0	1	Economic conditions (e.g., financial stress), Academic or work pressure, Family relationships	Urban environment		Higher levels of stress and pace of life, More noise and pollution, Lack of nature and quiet places	In a city	5	I don't know	3	I don't know	2	Improving access to professional care (more therapists, shorter waiting times), Raising awareness of mental health through campaigns	Often
20	2	South Moravian Region	4	0	0	Economic conditions (e.g., financial stress), Academic or work pressure, Family relationships	Urban environment	Greater social and cultural opportunities (cinemas, concerts, cafés, etc.), Accessibility of employment and education, Opportunity to establish more social contacts	More noise and pollution, Lack of nature and quiet places, Greater anonymity and feelings of loneliness	A combination of urban and rural living	2	Yes, older parental age improves children's mental health	2	Yes, a younger age (up to 21) may negatively affect her mental health	3	Improving access to specialist care (more therapists, shorter waiting times), creating a better preventive care system	Always
21	1	Vysočina Region	3	0	0	Economic conditions (e.g., financial stress), Academic or work pressure, Family relationships	Urban environment	Greater social and cultural opportunities (cinemas, concerts, cafés, etc.), Accessibility of employment and education, Greater anonymity and privacy	More noise and pollution, Lack of nature and quiet places, Greater competitive pressure in the job market	In the countryside	5	I don't know	2	Yes, a younger age (up to 21) may negatively affect her mental health	1	Greater support in schools and universities, Creation of a better preventive care system	Often
20	2	South Moravian Region	4	0	2	Economic conditions (e.g., financial stress), Academic or work pressure, Family relationships	Mixed (on the outskirts of a city or in small towns)	Easier access to medical and mental health care, Accessibility of employment and education, Better accessibility of public transport		A combination of urban and rural living	4	No, parental age has no effect	1	No, maternal age has no effect on her mental health	3	Improving the affordability of mental health care, Creating a better preventive care system	Occasionally
21	1	Zlín Region	3	5	3	Economic conditions (e.g., financial stress), Academic or work pressure, Family relationships	Mixed (on the outskirts of a city or in small towns)	Easier access to medical and mental health care, Greater social and cultural opportunities (cinemas, concerts, cafés, etc.), Accessibility of employment and education	Higher levels of stress and pace of life, Lack of nature and quiet places, Greater competitive pressure in the job market	A combination of urban and rural living	5	Yes, younger parental age improves children's mental health	1	I don't know	1	Improving the affordability of mental health care, Raising awareness of mental health through campaigns	Never
22	1	South Moravian Region	3	4	1	Economic conditions (e.g., financial stress), Academic or work pressure, Family relationships	Rural environment	Accessibility of employment and education, Greater anonymity and privacy, Better accessibility of public transport	More noise and pollution, Lack of nature and quiet places, Greater competitive pressure in the job market	A combination of urban and rural living	4	No, parental age has no effect	1	No, maternal age has no effect on her mental health	3	Improved access to specialist care (more therapists, shorter waiting times)	Often
20	2	South Moravian Region	4	4	2	Economic conditions (e.g., financial stress), Academic or work pressure, Family relationships	Urban environment			In a city	4	No, parental age has no effect	1	I don't know	3		Never
22	1	South Moravian Region	3	0	0	Economic conditions (e.g., financial stress), Academic or work pressure, Family relationships	Urban environment	Greater social and cultural opportunities (cinemas, concerts, cafés, etc.), Accessibility of employment and education, Greater anonymity and privacy	Higher levels of stress and pace of life, Lack of nature and quiet places, Greater competitive pressure in the job market	A combination of urban and rural living	4	I don't know	3	Yes, a younger age (up to 21) may negatively affect her mental health	2	Creating a better preventive care system	Always
23	1	Olomouc Region	3	0	0	Economic conditions (e.g., financial stress), Academic or work pressure, Family relationships	Urban environment	Easier access to medical and mental health care, Accessibility of employment and education, Greater anonymity and privacy	Higher levels of stress and pace of life, More noise and pollution, Lack of nature and quiet places	A combination of urban and rural living	5	No, parental age has no effect	4	I don't know	3	Raising awareness of mental health through campaigns, Greater support in schools and universities	Often
22	1	Moravian-Silesian Region	4	0	0	Economic conditions (e.g., financial stress), Academic or work pressure, Family relationships	Mixed (on the outskirts of a city or in small towns)	Greater social and cultural opportunities (cinemas, concerts, cafés, etc.), better access to public transport, opportunity to make more social contacts	Lack of nature and quiet places, Greater anonymity and feelings of loneliness, Higher crime rates	In the countryside	5	No, parental age has no effect	5	Yes, a younger age (up to 21) may negatively affect her mental health	1	Improving the affordability of mental health care, Greater support in schools and universities	Often
22	1	Moravian-Silesian Region	3	2	1	Economic conditions (e.g., financial stress), Academic or work pressure, Family relationships	Urban environment	Greater social and cultural opportunities (cinemas, concerts, cafés, etc.), Accessibility of employment and education, Greater anonymity and privacy	More noise and pollution, Lack of nature and quiet places, Higher crime rate	A combination of urban and rural living	4	Yes, younger parental age improves children's mental health	2	Yes, a younger age (up to 21) may negatively affect her mental health	2	Improving the affordability of mental health care, Raising awareness of mental health through campaigns	Often
22	1	Moravian-Silesian Region	2	0	0	Economic conditions (e.g., financial stress), Academic or work pressure, Family relationships	Rural environment	Easier access to medical and mental health care, Greater social and cultural opportunities (cinemas, concerts, cafés, etc.), Better public transport accessibility	Higher levels of stress and pace of life, Lack of nature and quiet places, Greater competitive pressure in the job market	A combination of urban and rural living	4	I don't know	3	No, maternal age has no effect on her mental health	2	Improving the affordability of mental health care, Greater support in schools and universities	Often
22	1	Moravian-Silesian Region	2	0	1	Economic conditions (e.g., financial stress), Academic or work pressure, Family relationships	Urban environment	Greater social and cultural opportunities (cinemas, concerts, cafés, etc.), Greater anonymity and privacy, Better access to public transport	Higher levels of stress and pace of life, Lack of nature and quiet places, Greater competitive pressure in the job market	In the countryside	5	Yes, younger parental age improves children's mental health	2	No, maternal age has no effect on her mental health	1	Improved access to specialist care (more therapists, shorter waiting times), Improved affordability of mental health care	Often
24	1	Moravian-Silesian Region	3	0	2	Economic conditions (e.g., financial stress), Academic or work pressure, Family relationships	Rural environment	Greater social and cultural opportunities (cinemas, concerts, cafés, etc.), Accessibility of employment and education, Greater anonymity and privacy	More noise and pollution, Lack of nature and quiet places, Higher crime rate	A combination of urban and rural living	4	I don't know	4	I don't know	3	Greater support in schools and universities, Creation of a better preventive care system	Occasionally
24	1	Zlín Region	4	0	0	Economic conditions (e.g., financial stress), Academic or work pressure, Family relationships	Rural environment	Easier access to medical and mental health care, Greater social and cultural opportunities (cinemas, concerts, cafés, etc.), Accessibility of employment and education	Higher levels of stress and pace of life, More noise and pollution, Lack of nature and quiet places	In the countryside	5	No, parental age has no effect	1	Yes, a younger age (up to 21) may negatively affect her mental health	3	Improving the affordability of mental health care, Raising awareness of mental health through campaigns	Occasionally
19	1	Moravian-Silesian Region	4	0	2	Economic conditions (e.g., financial stress), academic or work pressure, family relationships, crime, and feelings of insecurity	Urban environment	Easier access to medical and mental health care, Greater social and cultural opportunities (cinemas, concerts, cafés, etc.), Accessibility of employment and education	Higher levels of stress and pace of life, Lack of nature and quiet places, Greater competitive pressure in the job market	In the countryside	5	Yes, younger parental age improves children's mental health	3	I don't know	4	Improved access to specialist care (more therapists, shorter waiting times), improved affordability of mental health care, greater support in schools and universities	Occasionally
22	1	South Moravian Region	4	4	2	Economic conditions (e.g., financial stress), academic or work pressure, family relationships, uncertainty about the future: global conflicts, climate crisis, real estate market, and housing affordability	Urban environment	Greater social and cultural opportunities (cinemas, concerts, cafés, etc.), Accessibility of employment and education, Greater anonymity and privacy	Higher levels of stress and pace of life, Lack of nature and quiet places, Greater competitive pressure in the job market	A combination of urban and rural living	5	Yes, older parental age improves children's mental health	2	Yes, an older age (over 21) may positively affect her mental health	3	Improving access to specialist care (more therapists, shorter waiting times), creating a better preventive care system	Often
24	1	Moravian-Silesian Region	3	0	1	Economic conditions (e.g., financial stress), Academic or work pressure, Family relationships, Loneliness and social isolation	Mixed (on the outskirts of a city or in small towns)	Greater social and cultural opportunities (cinemas, concerts, cafés, etc.), Accessibility of employment and education, Greater anonymity and privacy	Higher levels of stress and pace of life, Higher crime rates	A combination of urban and rural living	5	No, parental age has no effect	4	Yes, a younger age (up to 21) may negatively affect her mental health	2	Improved access to specialist care (more therapists, shorter waiting times), improved affordability of mental health care, greater support in schools and universities	Always
25	2	Moravian-Silesian Region	3	0	0	Economic conditions (e.g., financial stress), Academic or work pressure, Family relationships, Loneliness and social isolation	Mixed (on the outskirts of a city or in small towns)	Greater social and cultural opportunities (cinemas, concerts, cafés, etc.)	Higher levels of stress and pace of life, More noise and pollution, Higher crime rates	I have no proference	5	No, parental age has no effect	4	I don't know	1	Creating a better preventive care system	Always
25	1	Moravian-Silesian Region	2	2	1	Economic conditions (e.g., financial stress), Academic or work pressure, Family relationships, Loneliness and social isolation	Mixed (on the outskirts of a city or in small towns)	Greater social and cultural opportunities (cinemas, concerts, cafés, etc.), Accessibility of employment and education, Opportunity to establish more social contacts	More noise and pollution, Lack of nature and quiet places, Greater competitive pressure in the job market	In a city	3	Yes, older parental age improves children's mental health	3	Yes, a younger age (up to 21) may negatively affect her mental health	1	Improved access to specialist care (more therapists, shorter waiting times), Improved affordability of mental health care	Always
21	1	Olomouc Region	3	2	0	Economic conditions (e.g., financial stress), Academic or work pressure, Family relationships, Loneliness and social isolation	Mixed (on the outskirts of a city or in small towns)	Greater social and cultural opportunities (cinemas, concerts, cafés, etc.), Accessibility of employment and education		In a city	3	No, parental age has no effect	2	No, maternal age has no effect on her mental health	2	Raising awareness of mental health through campaigns, Greater support in schools and universities, Creating a better preventive care system	Often
21	1	South Moravian Region	3	0	2	Economic conditions (e.g., financial stress), Academic or work pressure, Family relationships, Loneliness and social isolation	Rural environment	Easier access to medical and mental health care, Greater social and cultural opportunities (cinemas, concerts, cafés, etc.)	More noise and pollution, Lack of nature and quiet places, Higher crime rate	In the countryside	5	No, parental age has no effect	2	Yes, an older age (over 21) may positively affect her mental health	3	Improved access to professional care (more therapists, shorter waiting times), Greater support in schools and universities	Often

Age	Gender	Region of Permanent Residence:	Q1. How would you rate your current mental state? (1 = Very poor, 5 = Very good)	Q2. If you sought professional help for your mental health (e.g., psychologist, psychiatrist), how would you rate the quality of these services? (1 = Very dissatisfied, 5 = Very satisfied)	Q3. Do you think mental health care is easily accessible in your city/region?	Q4: Which factors do you think affect the mental health of young people aged 18–25 the most? (select the 3 most important)	Q5. Do you live in an urban or rural environment?	Q6. What benefits of living in the city do you think have a positive impact on mental health? (select the 3 most important)	Q7. What disadvantages of living in the city do you think deteriorate mental health? (select the 3 most important)	Q8. If you could choose, where would you prefer to live in terms of mental health?	Q9. How important is support from family and friends to you in dealing with mental health problems? (1 = Not important, 5 = Very important)	Q10. Do you think the age at which people enter parenthood can affect the mental health of their children?	Q11. How much of an impact do you think crime in your region has on your mental health? (1 = No effect, 5 = Very strong effect)	Q12. Do you think the age of the first-time mother can affect her mental health?	Q13.How would you rate the level of general awareness and education about mental health in secondary schools and colleges? (1 = Very poor, 5 = Very good)	Q14. Which of the following do you think would be most helpful in improving mental health care for young people in your region? (select up to 2 options)	Q15. How often do you feel you have to look after your mental health on your own, without the help of professionals or family?
20	1	South Moravian Region	2	0	0	Economic conditions (e.g., financial stress), Academic or work pressure, Family relationships, Loneliness and social isolation	Rural environment		Lack of nature and quiet places, Greater anonymity and feelings of loneliness, Higher crime rates	A combination of urban and rural living	5	I don't know	3	Yes, a younger age (up to 21) may negatively affect her mental health	2	Improving the affordability of mental health care, Raising awareness of mental health through campaigns, Creating a better preventive care system	Always
22	1	South Moravian Region	4	0	3	Economic conditions (e.g., financial stress), Academic or work pressure, Family relationships, Loneliness and social isolation	Rural environment		More noise and pollution, Lack of nature and quiet places, Higher crime rate	In a city	3	I don't know	3	I don't know	5	Improving the affordability of mental health care, Greater support in schools and universities, Creating a better preventive care system	Always
20	1	South Moravian Region	2	0	1	Economic conditions (e.g., financial stress), Academic or work pressure, Family relationships, Loneliness and social isolation	Mixed (on the outskirts of a city or in small towns)	Greater social and cultural opportunities (cinemas, concerts, cafés, etc.), Accessibility of employment and education	Higher levels of stress and pace of life, More noise and pollution, Lack of nature and quiet places, Greater competitive pressure in the job market	In the countryside	2	Yes, older parental age improves children's mental health	1	Yes, a younger age (up to 21) may negatively affect her mental health	3	Improved access to specialist care (more therapists, shorter waiting times), improved affordability of mental health care, greater support in schools and universities, creation of a better preventive care system.	Always
20	1	Moravian-Silesian Region	3	0	2	Economic conditions (e.g., financial stress), Academic or work pressure, Family relationships, Loneliness and social isolation	Urban environment	Greater social and cultural opportunities (cinemas, concerts, cafés, etc.), better access to public transport, opportunity to make more social contacts	Higher levels of stress and pace of life, greater competitive pressure in the labor market	A combination of urban and rural living	2	Yes, older parental age improves children's mental health	2	Yes, a younger age (up to 21) may negatively affect her mental health	2	Improved access to specialist care (more therapists, shorter waiting times), greater support in schools and universities, creation of a better preventive care system.	Occasionally
25	1	Moravian-Silesian Region	4	0	2	Economic conditions (e.g., financial stress), Academic or work pressure, Family relationships, Loneliness and social isolation	Urban environment	Easier access to medical and mental health care, Greater social and cultural opportunities (cinemas, concerts, cafés, etc.), Accessibility of employment and education, Opportunity to establish more social contacts	Higher levels of stress and pace of life, More noise and pollution, Lack of nature and quiet places	A combination of urban and rural living	5	No, parental age has no effect	3	I don't know	4	Improved access to specialist care (more therapists, shorter waiting times), Improved affordability of mental health care	Often
21	1	South Moravian Region	3	3	0	Economic conditions (e.g., financial stress), Academic or work pressure, Family relationships, Loneliness and social isolation	Urban environment	Greater social and cultural opportunities (cinemas, concerts, cafés, etc.), Accessibility of employment and education, Opportunity to establish more social contacts	Higher levels of stress and pace of life, More noise and pollution, Higher crime rates	In a city	3	I don't know	2	Yes, a younger age (up to 21) may negatively affect her mental health	2	Greater support in schools and universities	Often
20	1	Zlín Region	3	0	1	Economic conditions (e.g., financial stress), Academic or work pressure, Family relationships, Loneliness and social isolation	Mixed (on the outskirts of a city or in small towns)	Greater social and cultural opportunities (cinemas, concerts, cafés, etc.), Accessibility of employment and education, Better accessibility of public transport	More noise and pollution, Lack of nature and quiet places, Higher crime rate	In the countryside	5	Yes, older parental age improves children's mental health	3	I don't know	1	Improving the affordability of mental health care, Raising awareness of mental health through campaigns	Always
19	1	South Moravian Region	3	3	2	Economic conditions (e.g., financial stress), Academic or work pressure, Family relationships, Loneliness and social isolation	Rural environment	Greater social and cultural opportunities (cinemas, concerts, cafés, etc.), Accessibility of employment and education, Better accessibility of public transport	Higher levels of stress and pace of life, More noise and pollution, Higher crime rates	In a city	5	No, parental age has no effect	2	No, maternal age has no effect on her mental health	3	Greater support in schools and universities, Creation of a better preventive care system	Often
21	1	Moravian-Silesian Region	3	0	0	Economic conditions (e.g., financial stress), Academic or work pressure, Family relationships, Loneliness and social isolation	Urban environment	Greater social and cultural opportunities (cinemas, concerts, cafés, etc.), Accessibility of employment and education, Opportunity to establish more social contacts	Higher levels of stress and pace of life, Lack of nature and quiet places, Higher crime rates, Greater competitive pressure in the job market	A combination of urban and rural living	5	Yes, older parental age improves children's mental health	2	Yes, a younger age (up to 21) may negatively affect her mental health	3	Improving access to specialist care (more therapists, shorter waiting times), improving the affordability of mental health care, raising awareness of mental health through campaigns, greater support in schools and universities, creating a better preventive care system.	Often
23	2	South Moravian Region	2	0	1	Economic conditions (e.g., financial stress), Academic or work pressure, Family relationships, Loneliness and social isolation	Urban environment	Greater social and cultural opportunities (cinemas, concerts, cafés, etc.), better access to public transport, opportunity to make more social contacts	Higher levels of stress and pace of life, Greater anonymity and feelings of loneliness, Greater competitive pressure in the job market	A combination of urban and rural living	4	Yes, older parental age improves children's mental health	2	Yes, a younger age (up to 21) may negatively affect her mental health	2	Improved access to specialist care (more therapists, shorter waiting times), Improved affordability of mental health care	Often
23	1	Moravian-Silesian Region	4	0	1	Economic conditions (e.g., financial stress), Academic or work pressure, Family relationships, Loneliness and social isolation	Rural environment	Easier access to medical and mental health care, Greater social and cultural opportunities (cinemas, concerts, cafés, etc.), Greater anonymity and privacy		A combination of urban and rural living	5	Yes, older parental age improves children's mental health	2	Yes, a younger age (up to 21) may negatively affect her mental health	2	Improving the affordability of mental health care, Greater support in schools and universities, Creating a better preventive care system	Occasionally
22	1	Olomouc Region	4	0	0	Economic conditions (e.g., financial stress), Study or work pressure, Family relationships, Loneliness and social isolation, Crime and feelings of insecurity	Urban environment	Easier access to medical and mental health care, Accessibility of employment and education, Better accessibility of public transport	More noise and pollution, Lack of nature and quiet places, Higher crime rate	A combination of urban and rural living	5	No, parental age has no effect	4	No, maternal age has no effect on her mental health	1	Raising awareness of mental health through campaigns, Greater support in schools and universities	Often
21	2	Moravian-Silesian Region	5	0	2	Economic conditions (e.g., financial stress), Study or work pressure, Family relationships, Loneliness and social isolation, Crime and feelings of insecurity	Rural environment	Easier access to medical and mental health care, Greater social and cultural opportunities (cinemas, concerts, cafés, etc.), Accessibility of employment and education, Better accessibility of public transport, Opportunity to establish more social contacts	Higher levels of stress and pace of life, Higher crime rates	A combination of urban and rural living	4	No, parental age has no effect	1	No, maternal age has no effect on her mental health	2	Improving access to specialist care (more therapists, shorter waiting times), Raising awareness of mental health through campaigns, Creating a better preventive care system	Never
18	1	South Moravian Region	4	0	2	Economic conditions (e.g., financial stress), Study or work pressure, Family relationships, Loneliness and social isolation, Crime and feelings of insecurity	Urban environment	Easier access to medical and mental health care, Greater social and cultural opportunities (cinemas, concerts, cafés, etc.), Accessibility of employment and education, Greater anonymity and privacy, Better accessibility of public transport, Opportunity to establish more social contacts	Higher levels of stress and pace of life, More noise and pollution, Lack of nature and quiet places, Greater anonymity and feelings of loneliness, Higher crime rates, Greater competitive pressure in the job market	A combination of urban and rural living	5	Yes, younger parental age improves children's mental health	3	Yes, an older age (over 21) may positively affect her mental health	3	Improving access to specialist care (more therapists, shorter waiting times), improving the affordability of mental health care, raising awareness of mental health through campaigns, greater support in schools and universities, creating a better preventive care system.	Often
22	1	Moravian-Silesian Region	3	0	0	Economic conditions (e.g., financial stress), Academic or work pressure, Family relationships, Loneliness and social isolation, Death of loved ones	Mixed (on the outskirts of a city or in small towns)	Greater social and cultural opportunities (cinemas, concerts, cafés, etc.), Accessibility of employment and education, Better accessibility of public transport, Opportunity to establish more social contacts		A combination of urban and rural living	5	No, parental age has no effect	1	Yes, an older age (over 21) may positively affect her mental health	2	More support at schools and universities, more support in terms of learning how to take care of your mental health yourself. People always help themselves the most, even if they seek external help. I am with myself 100% of the time and I should learn to take care of myself.	Often
22	2	Moravian-Silesian Region	4	0	0	Economic conditions (e.g., financial stress), Study or work pressure, Health, illness	Rural environment	Greater social and cultural opportunities (cinemas, concerts, cafés, etc.), Opportunity to establish more social contacts	More noise and pollution, Lack of nature and quiet places, Higher crime rate	In the countryside	5	No, parental age has no effect	2	No, maternal age has no effect on her mental health	4	Improving the affordability of mental health care, Raising awareness of mental health through campaigns	Occasionally
24	2	Moravian-Silesian Region	2	4	2	Economic conditions (e.g., financial stress), Study or work pressure, Health condition	Urban environment	Easier access to medical and mental health care, Greater social and cultural opportunities (cinemas, concerts, cafés, etc.), Accessibility of employment and education	Higher levels of stress and pace of life, More noise and pollution, Lack of nature and quiet places	A combination of urban and rural living	4	Yes, younger parental age improves children's mental health	3	Yes, a younger age (up to 21) may negatively affect her mental health	2	Raising awareness of mental health through campaigns, Greater support in schools and universities	Often
19	1	Zlín Region	3	2	1	Combination of multiple factors	Urban environment	Greater social and cultural opportunities (cinemas, concerts, cafés, etc.), Accessibility of employment and education, Greater anonymity and privacy, Better accessibility of public transport	Higher crime rate	I have no preference	4	No, parental age has no effect	2	No, maternal age has no effect on her mental health	5	Raising awareness of mental health through campaigns, Greater support in schools and universities	Always
21	2	Moravian-Silesian Region	1	0	1	Loneliness	Rural environment	Easier access to medical and mental health care, Greater social and cultural opportunities (cinemas, concerts, cafés, etc.), Accessibility of employment and education, Opportunity to establish more social contacts	Higher levels of stress and pace of life, Lack of nature and quiet places, Greater anonymity and feelings of loneliness, Higher crime rates	A combination of urban and rural living	5	I don't know	5	Yes, a younger age (up to 21) may negatively affect her mental health	1	Improved access to specialist care (more therapists, shorter waiting times), improved affordability of mental health care, greater support in schools and universities, creation of a better preventive care system.	Always
20	2	South Moravian Region	2	0	0	Loneliness and social isolation, I don't really know	Urban environment	Accessibility of employment and education, Better accessibility of public transport		In the countryside	2	I don't know	2	I don't know	3	Improve access to specialist care (more therapists, shorter waiting times), privatize and strongly deregulate education, the labor market, and healthcare, and generally move closer to respecting negative rights.	Never
22	1	Moravian-Silesian Region	4	0	0	Family relationships	Urban environment	Greater social and cultural opportunities (cinemas, concerts, cafés, etc.), Accessibility of employment and education, Greater anonymity and privacy	More noise and pollution, higher crime rates	A combination of urban and rural living	3	Yes, older parental age improves children's mental health	3	Yes, a younger age (up to 21) may negatively affect her mental health	2	Improved access to specialist care (more therapists, shorter waiting times), greater support in schools and universities, creation of a better preventive care system.	Occasionally
26	1	Zlín Region	4	0	0	Family relationships	Mixed (on the outskirts of a city or in small towns)	Greater social and cultural opportunities (cinemas, concerts, cafés, etc.), Accessibility of employment and education		A combination of urban and rural living	5	Yes, younger parental age improves children's mental health	2	Yes, an older age (over 21) may positively affect her mental health	3	Improved access to specialist care (more therapists, shorter waiting times), Improved affordability of mental health care	Often
23	2	Moravian-Silesian Region	4	0	0	Family relationships, loneliness, and social isolation	Urban environment	Greater social and cultural opportunities (cinemas, concerts, cafés, etc.), Accessibility of employment and education, Opportunity to establish more social contacts	Higher levels of stress and pace of life, Higher crime rates	A combination of urban and rural living	3	No, parental age has no effect	2	No, maternal age has no effect on her mental health	2	Improving access to specialist care (more therapists, shorter waiting times), improving the affordability of mental health care, raising awareness of mental health through campaigns, greater support in schools and universities, creating a better preventive care system.	Often
21	1	Vysočina Region	3	2	2	Family relationships, loneliness, and social isolation	Rural environment	Greater social and cultural opportunities (cinemas, concerts, cafés, etc.), Opportunity to establish more social contacts	Higher levels of stress and pace of life, Lack of nature and quiet places	A combination of urban and rural living	2	I don't know	1	Yes, a younger age (up to 21) may negatively affect her mental health	3	Improving the affordability of mental health care, Creating a better preventive care system	Often
22	1	Capital City of Prague	4	0	0	Family relationships, loneliness, and social isolation	Urban environment	Easier access to medical and mental health care, Greater social and cultural opportunities (cinemas, concerts, cafés, etc.), Accessibility of employment and education, Greater anonymity and privacy, Better accessibility of public transport	Higher levels of stress and pace of life, More noise and pollution	In a city	4	I don't know	2	I don't know	3	Improving the affordability of mental health care, Raising awareness of mental health through campaigns, Greater support in schools and universities	Never
20	2	South Moravian Region	5	0	2	Family relationships, loneliness, and social isolation	Urban environment	I don't think that living in the city affects my mental state in any way.	I don't think it has any effect.	I have no preference	3	No, parental age has no effect	2	Yes, a younger age (up to 21) may negatively affect her mental health	2	The basis of a healthy soul is a healthy body, so I wouldn't go down the route of therapists and the like, because once a therapist is needed, I would say it's too late. It's better to go down the route of prevention, i.e., an active lifestyle, simply doing something productive and not just sitting around watching Netflix.	Always
23	1	Moravian-Silesian Region	4	0	0	Family relationships, Loneliness and social isolation, Crime and feelings of insecurity	Urban environment	Easier access to medical and mental health care, Greater social and cultural opportunities (cinemas, concerts, cafés, etc.), Accessibility of employment and education	Higher crime rates, greater competitive pressure in the labor market	A combination of urban and rural living	5	Yes, older parental age improves children's mental health	2	Yes, a younger age (up to 21) may negatively affect her mental health	4	Improving the affordability of mental health care, Creating a better preventive care system	Never
24	2	Moravian-Silesian Region	1	3	1	Family relationships, Loneliness and social isolation, Inadequate access to healthcare	Mixed (on the outskirts of a city or in small towns)	Easier access to medical and mental health care, Greater social and cultural opportunities (cinemas, concerts, cafés, etc.), Opportunity to establish more social contacts	Higher levels of stress and pace of life, More noise and pollution, Lack of nature and quiet places	In the countryside	4	Yes, older parental age improves children's mental health	2	Yes, a younger age (up to 21) may negatively affect her mental health	2	Improving access to professional care (more therapists, shorter waiting times), Raising awareness of mental health through campaigns	Often
23	1	Olomouc Region	3	0	2	Family relationships, Loneliness and social isolation, Inadequate access to healthcare	Urban environment	Easier access to medical and mental health care, Greater social and cultural opportunities (cinemas, concerts, cafés, etc.), Greater anonymity and privacy	Higher levels of stress and pace of life, Higher crime rates, Greater competitive pressure in the labor market	A combination of urban and rural living	5	I don't know	3	Yes, a younger age (up to 21) may negatively affect her mental health	2	Improving access to professional care (more therapists, shorter waiting times), Raising awareness of mental health through campaigns	Occasionally
22	1	Moravian-Silesian Region	4	1	2	Family relationships, loneliness and social isolation, social relationships (friends)	Urban environment	Greater social and cultural opportunities (cinemas, concerts, cafés, etc.), better access to public transport, opportunity to make more social contacts	Higher levels of stress and pace of life, Lack of nature and quiet places, Greater anonymity and feelings of loneliness	A combination of urban and rural living	4	Yes, younger parental age improves children's mental health	3	No, maternal age has no effect on her mental health	2	Raising awareness of mental health through campaigns, Greater support in schools and universities, Creating a better preventive care system	Often
19	2	Olomouc Region	5	0	0	weakness	Rural environment	Easier access to medical and mental health care	More noise and pollution, Lack of nature and quiet places, Higher crime rate	In the countryside	5	No, parental age has no effect	2	No, maternal age has no effect on her mental health	4	Creating a better preventive care system	Never
22	2	Central Bohemian Region	3	3	1	Social media and technology	Urban environment		Higher levels of stress and pace of life, More noise and pollution, Greater anonymity and feelings of loneliness	A combination of urban and rural living	4	I don't know	3	I don't know	1	Improving access to specialist care (more therapists, shorter waiting times), improving the affordability of mental health care, raising awareness of mental health through campaigns	Often
23	1	Zlín Region	3	0	1	Social media and technology	Mixed (on the outskirts of a city or in small towns)	Greater social and cultural opportunities (cinemas, concerts, cafés, etc.), Accessibility of employment and education	More noise and pollution, Lack of nature and quiet places, Higher crime rate	A combination of urban and rural living	4	Yes, younger parental age improves children's mental health	3	Yes, an older age (over 21) may positively affect her mental health	3	Creating a better preventive care system	Always
20	2	Moravian-Silesian Region	5	0	0	Social media and technology, Crime and feelings of insecurity, excess free time and propaganda	Urban environment	Access to employment and education	Higher levels of stress and pace of life, Lack of nature and quiet places, Higher crime rates	I have no preference	3	Yes, older parental age improves children's mental health	5	Yes, a younger age (up to 21) may negatively affect her mental health	3	Above all, stop looking for enemies around you and focus on the truly important internal priorities of the state.	Always
20	1	Moravian-Silesian Region	5	0	0	Social media and technology, Loneliness and social isolation	Mixed (on the outskirts of a city or in small towns)	Greater social and cultural opportunities (cinemas, concerts, cafés, etc.), Accessibility of employment and education, Opportunity to establish more social contacts		A combination of urban and rural living	5	Yes, younger parental age improves children's mental health	3	No, maternal age has no effect on her mental health	4	Raising awareness of mental health through campaigns,	Always
23	2	Zlín Region	4	0	2	Social media and technology, Loneliness and social isolation	Rural environment	Greater social and cultural opportunities (cinemas, concerts, cafés, etc.), greater anonymity and privacy	There may be nothing to do in the apartment, there is a lack of practical activities.	In the countryside	1	Yes, older parental age improves children's mental health	1	No, maternal age has no effect on her mental health	3	Don't take yourself so seriously, nowadays every other person is mentally ill, which is completely irrelevant.	Never
22	1	Moravian-Silesian Region	1	3	2	Social media and technology, Loneliness and social isolation	Mixed (on the outskirts of a city or in small towns)		Higher levels of stress and pace of life, Greater anonymity and feelings of loneliness, Greater competitive pressure in the job market	In the countryside	4	Yes, older parental age improves children's mental health	1	Yes, a younger age (up to 21) may negatively affect her mental health	3	Improving access to specialist care (more therapists, shorter waiting times), creating a better preventive care system	Occasionally
22	2	Moravian-Silesian Region	4	0	0	Social media and technology, Loneliness and social isolation	Mixed (on the outskirts of a city or in small towns)	Easier access to medical and mental health care, Accessibility of employment and education, Better accessibility of public transport, Opportunity to establish more social contacts		I have no preference	4	I don't know	2	Yes, a younger age (up to 21) may negatively affect her mental health	1	Raising awareness of mental health through campaigns, Greater support in schools and universities	Often
26	1	Moravian-Silesian Region	4	0	0	Social media and technology, Loneliness and social isolation	Rural environment	Easier access to medical and mental health care, Greater social and cultural opportunities (cinemas, concerts, cafés, etc.)	Higher levels of stress and pace of life, Lack of nature and quiet places	A combination of urban and rural living	4	No, parental age has no effect	1	No, maternal age has no effect on her mental health	4	Raising awareness of mental health through campaigns	Always
22	1	Moravian-Silesian Region	5	1	1	Social media and technology, Loneliness and social isolation	Urban environment		Higher levels of stress and pace of life, More noise and pollution, Greater anonymity and feelings of loneliness	In a city	5	No, parental age has no effect	2	Yes, a younger age (up to 21) may negatively affect her mental health	2	Improving access to specialist care (more therapists, shorter waiting times), creating a better preventive care system	Occasionally
25	2	South Moravian Region	4	0	3	Social media and technology, Loneliness and social isolation	Urban environment	Greater social and cultural opportunities (cinemas, concerts, cafés, etc.), Accessibility of employment and education	Higher levels of stress and pace of life, greater anonymity and feelings of loneliness	In a city	5	No, parental age has no effect	3	Yes, an older age (over 21) may positively affect her mental health	2	Improved access to professional care (more therapists, shorter waiting times), Greater support in schools and universities	Occasionally
24	2	Moravian-Silesian Region	5	0	0	Social media and technology, Loneliness and social isolation, The future	Mixed (on the outskirts of a city or in small towns)	Greater social and cultural opportunities (cinemas, concerts, cafés, etc.), better access to public transport, opportunity to make more social contacts	More noise and pollution, Lack of nature and quiet places, Greater anonymity and feelings of loneliness	In a city	2	I don't know	2	I don't know	3	Raising awareness of mental health through campaigns	Often
21	1	Zlín Region	3	5	2	Social media and technology, Loneliness and social isolation, Crime and feelings of insecurity	Mixed (on the outskirts of a city or in small towns)	Greater social and cultural opportunities (cinemas, concerts, cafés, etc.), Greater anonymity and privacy, Opportunity to establish more social contacts	More noise and pollution, Lack of nature and quiet places, Greater competitive pressure in the job market	A combination of urban and rural living	4	Yes, younger parental age improves children's mental health	2	Yes, an older age (over 21) may positively affect her mental health	1	Raising awareness of mental health through campaigns, Greater support in schools and universities	Often
21	2	Moravian-Silesian Region	5	0	0	Social media and technology, Loneliness and social isolation, Procrastination	Mixed (on the outskirts of a city or in small towns)	Greater social and cultural opportunities (cinemas, concerts, cafés, etc.), Greater anonymity and privacy, Opportunity to establish more social contacts		In the countryside	1	Yes, older parental age improves children's mental health	1	I don't know	3	Creating a better preventive care system, Socialization	Never
24	2	Zlín Region	5	0	0	Social media and technology, Loneliness and social isolation, Overthinking minor and insignificant "problems"	Mixed (on the outskirts of a city or in small towns)	Greater social and cultural opportunities (cinemas, concerts, cafés, etc.), Accessibility of employment and education, Opportunity to establish more social contacts	Higher levels of stress and pace of life, Lack of nature and quiet places, Greater competitive pressure in the job market	A combination of urban and rural living	2	Yes, older parental age improves children's mental health	1	Yes, a younger age (up to 21) may negatively affect her mental health	3		Never
23	2	Moravian-Silesian Region	5	0	2		Rural environment	More social and cultural opportunities (cinemas, concerts, cafés, etc.), Better public transport accessibility, Everything is close by	Higher levels of stress and pace of life, More noise and pollution, Lack of nature and quiet places	In the countryside	4	No, parental age has no effect	3	No, maternal age has no effect on her mental health	3	Creating leisure activities where young people will not have access to the internet, or turning off social networks for a certain period of time.	Often
24	2	Moravian-Silesian Region	4	5	2	Social media and technology, Family relationships	Urban environment		Higher levels of stress and pace of life, More noise and pollution, Lack of nature and quiet places	In the countryside	5	I don't know	2	Yes, a younger age (up to 21) may negatively affect her mental health	1	Raising awareness of mental health through campaigns, Greater support in schools and universities	Occasionally
22	2	Moravian-Silesian Region	2	0	0	Social media and technology, Family relationships	Rural environment	Greater anonymity and privacy, Opportunity to establish more social contacts	Higher levels of stress and pace of life, More noise and pollution, Lack of nature and quiet places, Higher crime rates	A combination of urban and rural living	4	No, parental age has no effect	2	Yes, a younger age (up to 21) may negatively affect her mental health	3	Improved access to specialist care (more therapists, shorter waiting times)	Often

Age	Gender	Region of Permanent Residence:	Q1. How would you rate your current mental state? (1 = Very poor, 5 = Very good)	Q2. If you sought professional help for your mental health (e.g., psychologist, psychiatrist), how would you rate the quality of these services? (1 = Very dissatisfied, 5 = Very satisfied)	Q3. Do you think mental health care is easily accessible in your city/region?	Q4: Which factors do you think affect the mental health of young people aged 18–25 the most? (select the 3 most important)	Q5. Do you live in an urban or rural environment?	Q6. What benefits of living in the city do you think have a positive impact on mental health? (select the 3 most important)	Q7. What disadvantages of living in the city do you think deteriorate mental health? (select the 3 most important)	Q8. If you could choose, where would you prefer to live in terms of mental health?	Q9. How important is support from family and friends to you in dealing with mental health problems? (1 = Not important, 5 = Very important)	Q10. Do you think the age at which people enter parenthood can affect the mental health of their children?	Q11. How much of an impact do you think crime in your region has on your mental health? (1 = No effect, 5 = Very strong effect)	Q12. Do you think the age of the first-time mother can affect her mental health?	Q13.How would you rate the level of general awareness and education about mental health in secondary schools and colleges? (1 = Very poor, 5 = Very good)	Q14. Which of the following do you think would be most helpful in improving mental health care for young people in your region? (select up to 2 options)	Q15. How often do you feel you have to look after your mental health on your own, without the help of professionals or family?
25	2	Moravian-Silesian Region	5	0	3	Social media and technology, Family relationships	Rural environment	Greater social and cultural opportunities (cinemas, concerts, cafés, etc.), better access to public transport, opportunity to make more social contacts	Higher levels of stress and pace of life, Lack of nature and quiet places, Greater anonymity and feelings of loneliness	In the countryside	5	No, parental age has no effect	3	No, maternal age has no effect on her mental health	2		Occasionally
20	1	Moravian-Silesian Region	4	0	2	Social media and technology, Family relationships	Rural environment	Easier access to medical and mental health care	Higher levels of stress and pace of life, More noise and pollution, Lack of nature and quiet places	A combination of urban and rural living	5	Yes, older parental age improves children's mental health	3	Yes, a younger age (up to 21) may negatively affect her mental health	3	Creating a better preventive care system	Occasionally
23	1	Moravian-Silesian Region	4	0	3	Social media and technology, Family relationships	Urban environment		Higher levels of stress and pace of life, Lack of nature and quiet places, Higher crime rates	A combination of urban and rural living	4	No, parental age has no effect	2	Yes, a younger age (up to 21) may negatively affect her mental health	2	Raising awareness of mental health through campaigns, Creating a better preventive care system	Often
20	2	Moravian-Silesian Region	5	0	0	Social media and technology, Family relationships, Crime and feelings of insecurity	Mixed (on the outskirts of a city or in small towns)	Easier access to medical and mental health care, Greater social and cultural opportunities (cinemas, concerts, cafés, etc.), Opportunity to establish more social contacts		I have no proference	1	Yes, younger parental age improves children's mental health	2	Yes, a younger age (up to 21) may negatively affect her mental health	4	Raising awareness of mental health through campaigns	Never
19	1	Moravian-Silesian Region	4	3	2	Social media and technology, Family relationships, Loneliness and social isolation	Mixed (on the outskirts of a city or in small towns)	Greater social and cultural opportunities (cinemas, concerts, cafés, etc.), Accessibility of employment and education, Opportunity to establish more social contacts	Higher levels of stress and pace of life, Lack of nature and quiet places, Higher crime rates	A combination of urban and rural living	4	I don't know	2	Yes, a younger age (up to 21) may negatively affect her mental health	1	Greater support in schools and universities, Creation of a better preventive care system	Always
20	1	Moravian-Silesian Region	4	0	2	Social media and technology, Family relationships, Loneliness and social isolation	Urban environment	Greater social and cultural opportunities (cinemas, concerts, cafés, etc.), Greater anonymity and privacy, Opportunity to establish more social contacts		In a city	5	Yes, older parental age improves children's mental health	1	Yes, a younger age (up to 21) may negatively affect her mental health	3	Improving the affordability of mental health care, Greater support in schools and universities	Often
23	1	Moravian-Silesian Region	4	2	1	Social media and technology, Family relationships, Loneliness and social isolation	Rural environment	Easier access to medical and mental health care	Higher levels of stress and pace of life, More noise and pollution, Lack of nature and quiet places, Greater anonymity and feelings of loneliness	In the countryside	2	I don't know	2	Yes, a younger age (up to 21) may negatively affect her mental health	1	Improved access to specialist care (more therapists, shorter waiting times), improved affordability of mental health care, greater support in schools and universities, creation of a better preventive care system.	Always
19	2	Moravian-Silesian Region	4	0	0	Social media and technology, Family relationships, Loneliness and social isolation	Mixed (on the outskirts of a city or in small towns)		Higher levels of stress and pace of life, Lack of nature and quiet places, Greater anonymity and feelings of loneliness	A combination of urban and rural living	4	Yes, older parental age improves children's mental health	2	Yes, a younger age (up to 21) may negatively affect her mental health	3	Raising awareness of mental health through campaigns	Never
20	1	Moravian-Silesian Region	3	4	2	Social media and technology, Family relationships, Loneliness and social isolation	Rural environment	Easier access to medical and mental health care, Greater social and cultural opportunities (cinemas, concerts, cafés, etc.), Greater anonymity and privacy	More noise and pollution, Lack of nature and quiet places, Greater anonymity and feelings of loneliness	A combination of urban and rural living	5	Yes, younger parental age improves children's mental health	2	Yes, a younger age (up to 21) may negatively affect her mental health	3	Improving the affordability of mental health care, Raising awareness of mental health through campaigns	Often
20	2	Moravian-Silesian Region	5	0	3	Social media and technology, Family relationships, Loneliness and social isolation	Rural environment	Greater social and cultural opportunities (cinemas, concerts, cafés, etc.), Greater anonymity and privacy, Opportunity to establish more social contacts	Higher levels of stress and pace of life, More noise and pollution, Lack of nature and quiet places	In the countryside	4	Yes, older parental age improves children's mental health	1	Yes, a younger age (up to 21) may negatively affect her mental health	2	Raising awareness of mental health through campaigns, Greater support in schools and universities	Never
20	1	Moravian-Silesian Region	4	0	0	Social media and technology, Family relationships, Loneliness and social isolation	Mixed (on the outskirts of a city or in small towns)	Greater social and cultural opportunities (cinemas, concerts, cafés, etc.), Accessibility of employment and education, Opportunity to establish more social contacts	Higher levels of stress and pace of life, More noise and pollution, Lack of nature and quiet places	A combination of urban and rural living	5	I don't know	2	Yes, a younger age (up to 21) may negatively affect her mental health	3	Improved access to professional care (more therapists, shorter waiting times), Greater support in schools and universities	Often
24	1	Moravian-Silesian Region	4	4	1	Social media and technology, Family relationships, Loneliness and social isolation	Rural environment	Greater social and cultural opportunities (cinemas, concerts, cafés, etc.), Accessibility of employment and education, Opportunity to establish more social contacts	Higher levels of stress and pace of life, More noise and pollution, Lack of nature and quiet places	In the countryside	5	Yes, younger parental age improves children's mental health	4	Yes, a younger age (up to 21) may negatively affect her mental health	2	Improved access to specialist care (more therapists, shorter waiting times), Improved affordability of mental health care	Occasionally
26	1	Moravian-Silesian Region	4	0	0	Social media and technology, Family relationships, Loneliness and social isolation	Rural environment	I cannot judge	Higher levels of stress and pace of life, More noise and pollution, Greater anonymity and feelings of loneliness	In the countryside	4	No, parental age has no effect	2	No, maternal age has no effect on her mental health	3	Raising awareness of mental health through campaigns, Greater support in schools and universities	Always
24	2	Olomouc Region	4	0	3	Social media and technology, Family relationships, Loneliness and social isolation	Urban environment	Greater social and cultural opportunities (cinemas, concerts, cafés, etc.), Accessibility of employment and education, Opportunity to establish more social contacts	Higher levels of stress and pace of life, More noise and pollution, Lack of nature and quiet places	A combination of urban and rural living	5	Yes, younger parental age improves children's mental health	1	Yes, a younger age (up to 21) may negatively affect her mental health	3	Raising awareness of mental health through campaigns, Greater support in schools and universities, Creating a better preventive care system	Occasionally
23	1	Moravian-Silesian Region	5	0	1	Social media and technology, Family relationships, Loneliness and social isolation	Urban environment	Greater social and cultural opportunities (cinemas, concerts, cafés, etc.), Accessibility of employment and education, Better accessibility of public transport	Higher levels of stress and pace of life, More noise and pollution, Lack of nature and quiet places, Greater anonymity and feelings of loneliness	A combination of urban and rural living	5	No, parental age has no effect	2	No, maternal age has no effect on her mental health	3	Improved access to specialist care (more therapists, shorter waiting times), Improved affordability of mental health care	Always
26	2	Moravian-Silesian Region	4	0	1	Social media and technology, Family relationships, Loneliness and social isolation	Urban environment	Easier access to medical and mental health care, Greater social and cultural opportunities (cinemas, concerts, cafés, etc.), Accessibility of employment and education	Higher levels of stress and pace of life, More noise and pollution, Higher crime rates	In a city	5	Yes, younger parental age improves children's mental health	3	No, maternal age has no effect on her mental health	3	Raising awareness of mental health through campaigns, Greater support in schools and universities	Always
22	1	Moravian-Silesian Region	4	4	3	Social media and technology, Family relationships, Loneliness and social isolation	Mixed (on the outskirts of a city or in small towns)	Easier access to medical and mental health care, Greater social and cultural opportunities (cinemas, concerts, cafés, etc.), Opportunity to establish more social contacts	Higher levels of stress and pace of life, More noise and pollution, Greater anonymity and feelings of loneliness	In the countryside	5	Yes, younger parental age improves children's mental health	3	I don't know	2	Raising awareness of mental health through campaigns, Greater support in schools and universities	Occasionally
22	2	Moravian-Silesian Region	3	0	2	Social media and technology, Family relationships, Loneliness and social isolation	Urban environment	Easier access to medical and mental health care, Greater social and cultural opportunities (cinemas, concerts, cafés, etc.), Opportunity to establish more social contacts	Higher levels of stress and pace of life, Lack of nature and quiet places, Greater anonymity and feelings of loneliness	A combination of urban and rural living	5	Yes, older parental age improves children's mental health	3	Yes, a younger age (up to 21) may negatively affect her mental health	2	Improving access to professional care (more therapists, shorter waiting times), Raising awareness of mental health through campaigns	Always
20	1	Moravian-Silesian Region	1	4	2	Social media and technology, Family relationships, Loneliness and social isolation	Mixed (on the outskirts of a city or in small towns)	Easier access to medical and mental health care, Greater anonymity and privacy, Better public transport accessibility	Higher levels of stress and pace of life, Greater anonymity and feelings of loneliness, Higher crime rates	A combination of urban and rural living	5	No, parental age has no effect	3	Yes, a younger age (up to 21) may negatively affect her mental health	1	Improved access to professional care (more therapists, shorter waiting times), Greater support in schools and universities	Often
20	1	Olomouc Region	4	0	1	Social media and technology, Family relationships, Loneliness and social isolation	Urban environment	Greater social and cultural opportunities (cinemas, concerts, cafés, etc.), Accessibility of employment and education, Better accessibility of public transport	Higher levels of stress and pace of life, Lack of nature and quiet places, Greater anonymity and feelings of loneliness	In a city	4	Yes, older parental age improves children's mental health	1	Yes, a younger age (up to 21) may negatively affect her mental health	2	Improved access to specialist care (more therapists, shorter waiting times), Improved affordability of mental health care	Occasionally
19	1	Moravian-Silesian Region	3	3	2	Social media and technology, Family relationships, Loneliness and social isolation	Mixed (on the outskirts of a city or in small towns)	Easier access to medical and mental health care, Greater social and cultural opportunities (cinemas, concerts, cafés, etc.), Opportunity to establish more social contacts	Higher levels of stress and pace of life, More noise and pollution, Lack of nature and quiet places	A combination of urban and rural living	4	No, parental age has no effect	1	Yes, a younger age (up to 21) may negatively affect her mental health	3	Improved access to specialist care (more therapists, shorter waiting times), Improved affordability of mental health care	Often
20	1	Moravian-Silesian Region	5	4	0	Social media and technology, Family relationships, Loneliness and social isolation	Rural environment	Greater social and cultural opportunities (cinemas, concerts, cafés, etc.), Accessibility of employment and education, Opportunity to establish more social contacts	Higher levels of stress and pace of life, More noise and pollution, Lack of nature and quiet places, Greater anonymity and feelings of loneliness, Greater competitive pressure in the job market	A combination of urban and rural living	5	No, parental age has no effect	1	No, maternal age has no effect on her mental health	1	Raising awareness of mental health through campaigns, Greater support in schools and universities, Creating a better preventive care system	Often
22	1	Moravian-Silesian Region	4	0	0	Social media and technology, Family relationships, Loneliness and social isolation	Mixed (on the outskirts of a city or in small towns)	Greater social and cultural opportunities (cinemas, concerts, cafés, etc.), Accessibility of employment and education, Better accessibility of public transport	Higher levels of stress and pace of life, Greater anonymity and feelings of loneliness, Greater competitive pressure in the job market	A combination of urban and rural living	4	Yes, older parental age improves children's mental health	2	Yes, an older age (over 21) may positively affect her mental health	3	Raising awareness of mental health through campaigns, Greater support in schools and universities	Occasionally
21	2	Moravian-Silesian Region	5	0	1	Social media and technology, Family relationships, Loneliness and social isolation	Urban environment	Greater social and cultural opportunities (cinemas, concerts, cafés, etc.), Accessibility of employment and education, Opportunity to establish more social contacts		In the countryside	5	I don't know	2	I don't know	3	Improved access to specialist care (more therapists, shorter waiting times), Improved affordability of mental health care	Occasionally
22	2	Moravian-Silesian Region	2	0	1	Social media and technology, Family relationships, Loneliness and social isolation	Urban environment	Greater social and cultural opportunities (cinemas, concerts, cafés, etc.), Accessibility of employment and education, Better accessibility of public transport	More noise and pollution	I have no proference	1	I don't know	1	I don't know	3	Ban the internet	Always
21	2	Moravian-Silesian Region	5	0	1	Social media and technology, Family relationships, Loneliness and social isolation	Mixed (on the outskirts of a city or in small towns)	Easier access to medical and mental health care, Greater social and cultural opportunities (cinemas, concerts, cafés, etc.), Opportunity to establish more social contacts	More noise and pollution, Lack of nature and quiet places, Higher crime rate	In the countryside	5	Yes, older parental age improves children's mental health	3	No, maternal age has no effect on her mental health	4	Improving access to specialist care (more therapists, shorter waiting times), creating a better preventive care system	Occasionally
26	2	Moravian-Silesian Region	5	0	0	Social media and technology, Family relationships, Loneliness and social isolation	Mixed (on the outskirts of a city or in small towns)	Greater social and cultural opportunities (cinemas, concerts, cafés, etc.), Greater anonymity and privacy, Opportunity to establish more social contacts	Higher levels of stress and pace of life, Higher crime rates, Greater competitive pressure in the labor market	I have no proference	4	No, parental age has no effect	2	I don't know	3	Improving the affordability of mental health care, Creating a better preventive care system	Never
26	1	Moravian-Silesian Region	5	0	3	Social media and technology, Family relationships, Loneliness and social isolation	Urban environment	Greater social and cultural opportunities (cinemas, concerts, cafés, etc.), Accessibility of employment and education, Opportunity to establish more social contacts	Higher levels of stress and pace of life, Greater anonymity and feelings of loneliness, Greater competitive pressure in the job market	A combination of urban and rural living	1	Yes, older parental age improves children's mental health	3	Yes, an older age (over 21) may positively affect her mental health	3	Improving the affordability of mental health care, Greater support in schools and universities	Occasionally
21	1	South Moravian Region	5	0	2	Social media and technology, Family relationships, Loneliness and social isolation	Urban environment	Easier access to medical and mental health care, Greater social and cultural opportunities (cinemas, concerts, cafés, etc.), Accessibility of employment and education, Opportunity to establish more social contacts		A combination of urban and rural living	5	Yes, older parental age improves children's mental health	1	Yes, a younger age (up to 21) may negatively affect her mental health	1	Improving access to specialist care (more therapists, shorter waiting times), improving the affordability of mental health care, raising awareness of mental health through campaigns	Never
19	2	South Moravian Region	2	0	0	Social media and technology, Family relationships, Loneliness and social isolation	Mixed (on the outskirts of a city or in small towns)	Greater social and cultural opportunities (cinemas, concerts, cafés, etc.), Accessibility of employment and education, Greater anonymity and privacy	Higher levels of stress and pace of life, Lack of nature and quiet places, Greater competitive pressure in the job market	In the countryside	4	Yes, older parental age improves children's mental health	2	Yes, a younger age (up to 21) may negatively affect her mental health	2	Raising awareness of mental health through campaigns, Greater support in schools and universities	Often
20	1	South Bohemian Region	2	4	2	Social media and technology, Family relationships, Loneliness and social isolation	Mixed (on the outskirts of a city or in small towns)	Easier access to medical and mental health care, Greater social and cultural opportunities (cinemas, concerts, cafés, etc.), Greater anonymity and privacy	Higher levels of stress and pace of life, More noise and pollution, Lack of nature and quiet places	In a city	2	Yes, older parental age improves children's mental health	1	Yes, a younger age (up to 21) may negatively affect her mental health	3	Improving the affordability of mental health care, Creating a better preventive care system	Often
20	2	Vysočina Region	4	0	0	Social media and technology, Family relationships, Loneliness and social isolation	Urban environment	Greater social and cultural opportunities (cinemas, concerts, cafés, etc.), Accessibility of employment and education, Opportunity to establish more social contacts	More noise and pollution, Lack of nature and quiet places	In the countryside	4	I don't know	1	I don't know	1	Greater support in schools and universities, Creation of a better preventive care system	Always
22	2	South Moravian Region	5	0	0	Social media and technology, Family relationships, Loneliness and social isolation	Urban environment	Greater social and cultural opportunities (cinemas, concerts, cafés, etc.), Accessibility of employment and education		In a city	4	No, parental age has no effect	2	I don't know	3	Improving the affordability of mental health care, Raising awareness of mental health through campaigns	Occasionally
24	2	South Moravian Region	3	0	0	Social media and technology, Family relationships, Loneliness and social isolation	Urban environment	Easier access to medical and mental health care, Greater social and cultural opportunities (cinemas, concerts, cafés, etc.), Opportunity to establish more social contacts	Higher levels of stress and pace of life, Lack of nature and quiet places, Greater competitive pressure in the job market	A combination of urban and rural living	5	Yes, younger parental age improves children's mental health	1	Yes, a younger age (up to 21) may negatively affect her mental health	2	Raising awareness of mental health through campaigns, Creating a better preventive care system	Occasionally
24	2	Zlín Region	5	0	3	Social media and technology, Family relationships, Loneliness and social isolation	Urban environment	Greater social and cultural opportunities (cinemas, concerts, cafés, etc.), Accessibility of employment and education, Better accessibility of public transport, Opportunity to establish more social contacts	Higher levels of stress and pace of life, Lack of nature and quiet places, Greater anonymity and feelings of loneliness, Higher crime rates	A combination of urban and rural living	5	Yes, younger parental age improves children's mental health	1	Yes, an older age (over 21) may positively affect her mental health	5	introduction of compulsory physical education throughout the entire period of study,	Occasionally
20	2	Zlín Region	5	0	0	Social media and technology, Family relationships, Loneliness and social isolation	Urban environment	Greater social and cultural opportunities (cinemas, concerts, cafés, etc.), Accessibility of employment and education, Opportunity to establish more social contacts		A combination of urban and rural living	1	I don't know	1	Yes, an older age (over 21) may positively affect her mental health	3	Greater support in schools and universities, Creation of a better preventive care system	Occasionally
21	2	Hradec Králové Region	5	3	2	Social media and technology, Family relationships, Loneliness and social isolation	Mixed (on the outskirts of a city or in small towns)	Greater social and cultural opportunities (cinemas, concerts, cafés, etc.), Accessibility of employment and education, Opportunity to establish more social contacts		A combination of urban and rural living	5	No, parental age has no effect	1	No, maternal age has no effect on her mental health	3	Greater support in schools and universities, Creation of a better preventive care system	Occasionally
20	1	Vysočina Region	4	0	2	Social media and technology, Family relationships, Loneliness and social isolation	Rural environment	Greater social and cultural opportunities (cinemas, concerts, cafés, etc.), Accessibility of employment and education, Greater anonymity and privacy	Higher levels of stress and pace of life, Lack of nature and quiet places	In the countryside	5	I don't know	1	Yes, an older age (over 21) may positively affect her mental health	3	Improving access to professional care (more therapists, shorter waiting times), Raising awareness of mental health through campaigns, Greater support in schools and universities	Often
21	2	Moravian-Silesian Region	4	0	1	Social media and technology, Family relationships, Loneliness and social isolation	Urban environment	Easier access to medical and mental health care, Greater social and cultural opportunities (cinemas, concerts, cafés, etc.), Accessibility of employment and education	More noise and pollution, Lack of nature and quiet places, Greater anonymity and feelings of loneliness	A combination of urban and rural living	4	Yes, younger parental age improves children's mental health	2	Yes, an older age (over 21) may positively affect her mental health	2	Raising awareness of mental health through campaigns, Creating a better preventive care system	Occasionally
21	1	South Moravian Region	2	4	1	Social media and technology, Family relationships, Loneliness and social isolation	Urban environment	Easier access to medical and mental health care, Greater social and cultural opportunities (cinemas, concerts, cafés, etc.), Accessibility of employment and education	Higher levels of stress and pace of life, Greater anonymity and feelings of loneliness, Greater competitive pressure in the job market	A combination of urban and rural living	5	Yes, younger parental age improves children's mental health	2	Yes, a younger age (up to 21) may negatively affect her mental health	2	Improving access to specialist care (more therapists, shorter waiting times), creating a better preventive care system	Never
19	1	Central Bohemian Region	3	0	1	Social media and technology, Family relationships, Loneliness and social isolation	Mixed (on the outskirts of a city or in small towns)	Easier access to medical and mental health care, Greater social and cultural opportunities (cinemas, concerts, cafés, etc.), Greater anonymity and privacy		A combination of urban and rural living	5	I don't know	2	I don't know	3	Improving access to professional care (more therapists, shorter waiting times), Raising awareness of mental health through campaigns	Often
22	2	Ústí nad Labem Region	3	0	0	Social media and technology, Family relationships, Loneliness and social isolation	Urban environment	Easier access to medical and mental health care, Greater social and cultural opportunities (cinemas, concerts, cafés, etc.), Better public transport accessibility	Higher levels of stress and pace of life, More noise and pollution, Greater anonymity and feelings of loneliness	A combination of urban and rural living	5	No, parental age has no effect	2	I don't know	3	Improving the affordability of mental health care, Creating a better preventive care system	Occasionally
20	2	South Moravian Region	5	0	0	Social media and technology, Family relationships, Loneliness and social isolation	Urban environment	Greater social and cultural opportunities (cinemas, concerts, cafés, etc.), Accessibility of employment and education, Opportunity to establish more social contacts	Higher levels of stress and pace of life, More noise and pollution, Lack of nature and quiet places	In a city	2	Yes, younger parental age improves children's mental health	1	Yes, a younger age (up to 21) may negatively affect her mental health	2	Improving the affordability of mental health care, Creating a better preventive care system	Never
22	1	Moravian-Silesian Region	5	5	2	Social media and technology, Family relationships, Loneliness and social isolation	Urban environment	Greater social and cultural opportunities (cinemas, concerts, cafés, etc.), Accessibility of employment and education	Higher levels of stress and pace of life, More noise and pollution, Lack of nature and quiet places, Higher crime rates, Greater competitive pressure in the job market	A combination of urban and rural living	5	No, parental age has no effect	3	Yes, a younger age (up to 21) may negatively affect her mental health	2	Greater support in schools and universities, Creation of a better preventive care system	Often
21	1	Central Bohemian Region	2	4	0	Social media and technology, Family relationships, Loneliness and social isolation	Urban environment	Easier access to medical and mental health care, Greater social and cultural opportunities (cinemas, concerts, cafés, etc.), Accessibility of employment and education, Better accessibility of public transport, Opportunity to establish more social contacts	Higher levels of stress and pace of life, More noise and pollution, Lack of nature and quiet places, Higher crime rates	A combination of urban and rural living	5	I don't know	2	Yes, a younger age (up to 21) may negatively affect her mental health	3	Improved access to specialist care (more therapists, shorter waiting times), greater support in schools and universities, creation of a better preventive care system.	Often
19	2	South Moravian Region	4	5	3	Social media and technology, Family relationships, Loneliness and social isolation	Rural environment	Greater social and cultural opportunities (cinemas, concerts, cafés, etc.), Greater anonymity and privacy, Opportunity to establish more social contacts	Higher levels of stress and pace of life, More noise and pollution, Lack of nature and quiet places	A combination of urban and rural living	4	I don't know	2	Yes, a younger age (up to 21) may negatively affect her mental health	2	Raising awareness of mental health through campaigns, Greater support in schools and universities	Always
26	2	Olomouc Region	4	0	0	Social media and technology, Family relationships, Loneliness and social isolation	Rural environment	Greater social and cultural opportunities (cinemas, concerts, cafés, etc.), Accessibility of employment and education, Better accessibility of public transport	Higher levels of stress and pace of life, More noise and pollution, Lack of nature and quiet places	A combination of urban and rural living	4	I don't know	2	I don't know	3	Improving the affordability of mental health care, Greater support in schools and universities	Often
19	2	South Moravian Region	2	4	1	Social media and technology, Family relationships, Loneliness and social isolation, Inadequate access to healthcare	Urban environment	Easier access to medical and mental health care, Greater social and cultural opportunities (cinemas, concerts, cafés, etc.), Opportunity to establish more social contacts	Lack of nature and quiet places, Greater anonymity and feelings of loneliness	In a city	5	Yes, younger parental age improves children's mental health	2	No, maternal age has no effect on her mental health	1	Improving the affordability of mental health care, Raising awareness of mental health through campaigns, Greater support in schools and universities	Always

Age	Gender	Region of Permanent Residence:	Q1. How would you rate your current mental state? (1 = Very poor, 5 = Very good)	Q2. If you sought professional help for your mental health (e.g., psychologist, psychiatrist), how would you rate the quality of these services? (1 = Very dissatisfied, 5 = Very satisfied)	Q3. Do you think mental health care is easily accessible in your city/region?	Q4: Which factors do you think affect the mental health of young people aged 18–25 the most? (select the 3 most important)	Q5. Do you live in an urban or rural environment?	Q6. What benefits of living in the city do you think have a positive impact on mental health? (select the 3 most important)	Q7. What disadvantages of living in the city do you think deteriorate mental health? (select the 3 most important)	Q8. If you could choose, where would you prefer to live in terms of mental health?	Q9. How important is support from family and friends to you in dealing with mental health problems? (1 = Not important, 5 = Very important)	Q10. Do you think the age at which people enter parenthood can affect the mental health of their children?	Q11. How much of an impact do you think crime in your region has on your mental health? (1 = No effect, 5 = Very strong effect)	Q12. Do you think the age of the first-time mother can affect her mental health?	Q13.How would you rate the level of general awareness and education about mental health in secondary schools and colleges? (1 = Very poor, 5 = Very good)	Q14. Which of the following do you think would be most helpful in improving mental health care for young people in your region? (select up to 2 options)	Q15. How often do you feel you have to look after your mental health on your own, without the help of professionals or family?
23	2	Moravian-Silesian Region	4	0	2	Social media and technology, Family relationships, Loneliness and social isolation, Weak willpower, nihilism, victim mentality	Urban environment	Easier access to medical and mental health care, Greater social and cultural opportunities (cinemas, concerts, cafés, etc.), Opportunity to establish more social contacts	More noise and pollution, Lack of nature and quiet places, Coming into contact with criminals, drug addicts, Roma people	In a city	3	I don't know	4	I don't know	4	Building structures and public spaces that look attractive/harmonious. In other words, those that were built 80 years ago and earlier.	Occasionally
20	1	Moravian-Silesian Region	4	3	2	Social media and technology, Family relationships, Eating disorders	Urban environment	Easier access to medical and mental health care, Greater social and cultural opportunities (cinemas, concerts, cafés, etc.), Greater anonymity and privacy	Lack of nature and quiet places, Higher crime rate, Greater competitive pressure in the labor market	In a city	2	No, parental age has no effect	1	I don't know	3	Raising awareness of mental health through campaigns, Creating a better preventive care system	Often
20	1	Moravian-Silesian Region	4	3	2	Social media and technology, Family relationships, Eating disorders	Urban environment	Easier access to medical and mental health care, Greater social and cultural opportunities (cinemas, concerts, cafés, etc.), Greater anonymity and privacy	Lack of nature and quiet places, Greater competitive pressure in the job market	In a city	2	No, parental age has no effect	1	I don't know	3	Raising awareness of mental health through campaigns, Greater support in schools and universities	Always
20	2	Moravian-Silesian Region	4	0	0	Social media and technology, Social pressure (society's expectations)	Mixed (on the outskirts of a city or in small towns)	Greater social and cultural opportunities (cinemas, concerts, cafés, etc.), Greater anonymity and privacy, Opportunity to establish more social contacts	Higher levels of stress and pace of life, Lack of nature and quiet places, Greater need to fit in	A combination of urban and rural living	2	I don't know	1	Yes, an older age (over 21) may positively affect her mental health	2	Raising awareness of mental health through campaigns, Greater support in schools and universities, Creating a better preventive care system	Always
20	1	Moravian-Silesian Region	5	0	0	Social media and technology, Study or work pressure	Rural environment	Greater social and cultural opportunities (cinemas, concerts, cafés, etc.), Opportunity to establish more social contacts		A combination of urban and rural living	5	Yes, younger parental age improves children's mental health	1	Yes, a younger age (up to 21) may negatively affect her mental health	1	Raising awareness of mental health through campaigns, Greater support in schools and universities, Creating a better preventive care system	Always
22	1	Olomouc Region	1	0	2	Social media and technology, Study or work pressure	Mixed (on the outskirts of a city or in small towns)	Greater social and cultural opportunities (cinemas, concerts, cafés, etc.), Accessibility of employment and education, Greater anonymity and privacy	Higher levels of stress and pace of life, More noise and pollution, Lack of nature and quiet places	In the countryside	4	I don't know	1	No, maternal age has no effect on her mental health	2	Raising awareness of mental health through campaigns, Greater support in schools and universities	Often
23	1	Olomouc Region	3	0	2	Social media and technology, Study or work pressure	Urban environment	Greater social and cultural opportunities (cinemas, concerts, cafés, etc.), Accessibility of employment and education, Better accessibility of public transport, Opportunity to establish more social contacts	Lack of nature and quiet places	In a city	5	Yes, older parental age improves children's mental health	3	Yes, a younger age (up to 21) may negatively affect her mental health	1	Greater support in schools and universities	Always
19	1	Moravian-Silesian Region	3	0	0	Social media and technology, Study or work pressure	Rural environment	Greater social and cultural opportunities (cinemas, concerts, cafés, etc.), better access to public transport	Higher levels of stress and pace of life, More noise and pollution	In the countryside	2	I don't know	2	I don't know	3	Raising awareness of mental health through campaigns, Greater support in schools and universities	Occasionally
20	2	Moravian-Silesian Region	4	0	3	Social media and technology, Study or work pressure	Mixed (on the outskirts of a city or in small towns)	Greater social and cultural opportunities (cinemas, concerts, cafés, etc.), Greater anonymity and privacy, Better access to public transport	Higher levels of stress and pace of life, More noise and pollution, Lack of nature and quiet places, Greater competitive pressure in the job market	A combination of urban and rural living	5	No, parental age has no effect	3	No, maternal age has no effect on her mental health	2	Raising awareness of mental health through campaigns, Creating a better preventive care system	Occasionally
26	1	Olomouc Region	4	3	2	Social media and technology, Study or work pressure	Urban environment	Greater social and cultural opportunities (cinemas, concerts, cafés, etc.), greater anonymity and privacy	Higher levels of stress and pace of life, More noise and pollution, Lack of nature and quiet places	In the countryside	3	No, parental age has no effect	1	No, maternal age has no effect on her mental health	3	Raising awareness of mental health through campaigns, Greater support in schools and universities	Occasionally
19	1	Pardubice Region	3	0	1	Social media and technology, Study or work pressure	Mixed (on the outskirts of a city or in small towns)		Higher levels of stress and pace of life, More noise and pollution, Lack of nature and quiet places, Higher crime rates	A combination of urban and rural living	5	Yes, younger parental age improves children's mental health	4	I don't know	2	Greater support in schools and universities, Creation of a better preventive care system	Often
21	1	Moravian-Silesian Region	3	0	2	Social media and technology, Study or work pressure, Crime and feeling unsafe	Urban environment	Greater anonymity and privacy, Better access to public transport, Opportunity to establish more social contacts	Higher levels of stress and pace of life, Lack of nature and quiet places, Higher crime rates	In the countryside	5	No, parental age has no effect	3	No, maternal age has no effect on her mental health	2	Greater support in schools and universities, Creation of a better preventive care system	Often
22	1	Moravian-Silesian Region	3	5	2	Social media and technology, Study or work pressure, Inadequate access to healthcare	Urban environment			A combination of urban and rural living	5	No, parental age has no effect	2	No, maternal age has no effect on her mental health	2	Improving access to professional care (more therapists, shorter waiting times), Raising awareness of mental health through campaigns, Greater support in schools and universities	Always
20	1	Olomouc Region	2	0	1	Social media and technology, Study or work pressure, Loneliness and social isolation	Rural environment	Easier access to medical and mental health care, Greater social and cultural opportunities (cinemas, concerts, cafés, etc.), Accessibility of employment and education	Higher levels of stress and pace of life, More noise and pollution, Lack of nature and quiet places	In the countryside	3	I don't know	3	Yes, an older age (over 21) may positively affect her mental health	2	Improving access to professional care (more therapists, shorter waiting times), Raising awareness of mental health through campaigns	Often
23	1	Pardubice Region	4	0	1	Social media and technology, Study or work pressure, Loneliness and social isolation	Urban environment	Greater social and cultural opportunities (cinemas, concerts, cafés, etc.), better access to public transport, sports facilities	Higher levels of stress and pace of life, More noise and pollution, Greater competitive pressure in the job market	A combination of urban and rural living	5	Yes, younger parental age improves children's mental health	1	No, maternal age has no effect on her mental health	2	Greater support in schools and universities, Creation of a better preventive care system	Occasionally
22	1	Moravian-Silesian Region	3	0	2	Social media and technology, Study or work pressure, Loneliness and social isolation	Urban environment	Easier access to medical and mental health care, Greater social and cultural opportunities (cinemas, concerts, cafés, etc.), Accessibility of employment and education	Higher levels of stress and pace of life, Lack of nature and quiet places, Greater competitive pressure in the job market	A combination of urban and rural living	4	No, parental age has no effect	3	No, maternal age has no effect on her mental health	4	Improving the affordability of mental health care, Greater support in schools and universities	Often
22	1	Olomouc Region	3	5	1	Social media and technology, Study or work pressure, Loneliness and social isolation	Urban environment	Easier access to medical and mental health care, Greater social and cultural opportunities (cinemas, concerts, cafés, etc.), Opportunity to establish more social contacts	Higher levels of stress and pace of life, Lack of nature and quiet places	A combination of urban and rural living	5	No, parental age has no effect	1	I don't know	2	Improved access to specialist care (more therapists, shorter waiting times), greater support in schools and universities, creation of a better preventive care system.	Occasionally
19	2	Moravian-Silesian Region	3	2	1	Social media and technology, Study or work pressure, Loneliness and social isolation	Urban environment	Easier access to medical and mental health care, Greater social and cultural opportunities (cinemas, concerts, cafés, etc.), Accessibility of employment and education	Higher levels of stress and pace of life, Greater anonymity and feelings of loneliness, Greater competitive pressure in the job market	A combination of urban and rural living	4	I don't know	2	I don't know	2	Improved access to specialist care (more therapists, shorter waiting times), Improved affordability of mental health care	Often
24	1	Moravian-Silesian Region	3	0	0	Social media and technology, Study or work pressure, Loneliness and social isolation	Rural environment	Greater social and cultural opportunities (cinemas, concerts, cafés, etc.), Accessibility of employment and education, Opportunity to establish more social contacts		In the countryside	4	Yes, older parental age improves children's mental health	3	Yes, an older age (over 21) may positively affect her mental health	2	Raising awareness of mental health through campaigns, Creating a better preventive care system	Always
22	1	Moravian-Silesian Region	3	0	2	Social media and technology, Study or work pressure, Loneliness and social isolation	Rural environment	Easier access to medical and mental health care, Greater social and cultural opportunities (cinemas, concerts, cafés, etc.), Opportunity to establish more social contacts	Higher levels of stress and pace of life, Lack of nature and quiet places, Greater competitive pressure in the job market	A combination of urban and rural living	5	Yes, older parental age improves children's mental health	4	Yes, a younger age (up to 21) may negatively affect her mental health	2	Improved access to specialist care (more therapists, shorter waiting times), improved affordability of mental health care, greater support in schools and universities	Often
22	1	Moravian-Silesian Region	3	0	2	Social media and technology, Study or work pressure, Loneliness and social isolation	Urban environment	Greater social and cultural opportunities (cinemas, concerts, cafés, etc.)	Lack of nature and quiet places, Higher crime rate	In the countryside	4	Yes, older parental age improves children's mental health	2	I don't know	2	Greater support in schools and universities, Creation of a better preventive care system	Often
21	1	Olomouc Region	3	2	2	Social media and technology, Study or work pressure, Loneliness and social isolation	Urban environment	Easier access to medical and mental health care, Greater social and cultural opportunities (cinemas, concerts, cafés, etc.), Greater anonymity and privacy, Better public transport accessibility	Higher levels of stress and pace of life, More noise and pollution, Lack of nature and quiet places	A combination of urban and rural living	3	I don't know	2	Yes, a younger age (up to 21) may negatively affect her mental health	2	Improving the affordability of mental health care, Raising awareness of mental health through campaigns, Greater support in schools and universities, Creating a better preventive care system	Often
20	1	Moravian-Silesian Region	3	0	1	Social media and technology, Study or work pressure, Loneliness and social isolation	Mixed (on the outskirts of a city or in small towns)	Easier access to medical and mental health care, Greater social and cultural opportunities (cinemas, concerts, cafés, etc.), Accessibility of employment and education	Higher levels of stress and pace of life, More noise and pollution, Lack of nature and quiet places	A combination of urban and rural living	3	I don't know	3	I don't know	3	Improving access to specialist care (more therapists, shorter waiting times), creating a better preventive care system	Never
21	2	Moravian-Silesian Region	4	2	2	Social media and technology, Study or work pressure, Loneliness and social isolation	Urban environment	Greater social and cultural opportunities (cinemas, concerts, cafés, etc.), Accessibility of employment and education, Better accessibility of public transport	Higher levels of stress and pace of life, Greater anonymity and feelings of loneliness, Higher crime rates	In a city	4	I don't know	2	I don't know	3	Improving access to specialist care (more therapists, shorter waiting times), creating a better preventive care system	Occasionally
20	2	Moravian-Silesian Region	5	0	0	Social media and technology, Study or work pressure, Loneliness and social isolation	Mixed (on the outskirts of a city or in small towns)	Greater social and cultural opportunities (cinemas, concerts, cafés, etc.), Accessibility of employment and education, Opportunity to establish more social contacts	Higher levels of stress and pace of life, More noise and pollution, Lack of nature and quiet places	A combination of urban and rural living	3	I don't know	1	I don't know	3	Creating a better preventive care system	Often
19	1	Moravian-Silesian Region	5	0	0	Social media and technology, Study or work pressure, Loneliness and social isolation	Mixed (on the outskirts of a city or in small towns)	Easier access to medical and mental health care, Greater social and cultural opportunities (cinemas, concerts, cafés, etc.), Greater anonymity and privacy	Higher levels of stress and pace of life, Greater anonymity and feelings of loneliness, Greater competitive pressure in the job market	In a city	4	No, parental age has no effect	3	I don't know	1	Greater support in schools and universities, Creation of a better preventive care system	Often
22	2	South Moravian Region	3	0	0	Social media and technology, Study or work pressure, Loneliness and social isolation	Urban environment	Greater social and cultural opportunities (cinemas, concerts, cafés, etc.), Accessibility of employment and education, Opportunity to establish more social contacts	Higher levels of stress and pace of life, Greater anonymity and feelings of loneliness, Greater competitive pressure in the job market	In the countryside	3	I don't know	1	I don't know	3	Improving the affordability of mental health care, Raising awareness of mental health through campaigns	Occasionally
20	1	Moravian-Silesian Region	2	0	0	Social media and technology, Study or work pressure, Loneliness and social isolation	Rural environment	Greater social and cultural opportunities (cinemas, concerts, cafés, etc.), better access to public transport, opportunity to make more social contacts		A combination of urban and rural living	2	I don't know	1	Yes, a younger age (up to 21) may negatively affect her mental health	2	Improving the affordability of mental health care, Raising awareness of mental health through campaigns, Creating a better preventive care system	Often
21	2	Karlovy Vary Region	3	0	0	Social media and technology, Study or work pressure, Loneliness and social isolation	Mixed (on the outskirts of a city or in small towns)	Greater social and cultural opportunities (cinemas, concerts, cafés, etc.), Accessibility of employment and education, Better accessibility of public transport	Higher levels of stress and pace of life, More noise and pollution, Greater anonymity and feelings of loneliness	A combination of urban and rural living	5	I don't know	1	Yes, a younger age (up to 21) may negatively affect her mental health	2	Raising awareness of mental health through campaigns, Greater support in schools and universities, Creating a better preventive care system	Occasionally
21	2	Moravian-Silesian Region	3	0	1	Social media and technology, Study or work pressure, Loneliness and social isolation	Urban environment	Greater social and cultural opportunities (cinemas, concerts, cafés, etc.), Greater anonymity and privacy, Opportunity to establish more social contacts	Higher levels of stress and pace of life, Lack of nature and quiet places	A combination of urban and rural living	2	Yes, younger parental age improves children's mental health	2	No, maternal age has no effect on her mental health	1	Raising awareness about mental health through campaigns, Avoiding toxic environments	Occasionally
21	2	Moravian-Silesian Region	4	0	0	Social media and technology, Study or work pressure, Loneliness and social isolation	Mixed (on the outskirts of a city or in small towns)	Accessibility of employment and education, Better accessibility of public transport	Higher levels of stress and pace of life, More noise and pollution, Lack of nature and quiet places	In the countryside	5	I don't know	1	Yes, a younger age (up to 21) may negatively affect her mental health	3		Often
22	2	Pardubice Region	3	0	0	Social media and technology, Study or work pressure, Loneliness and social isolation	Urban environment	Greater social and cultural opportunities (cinemas, concerts, cafés, etc.), Accessibility of employment and education, Better accessibility of public transport	Lack of nature and quiet places, Greater anonymity and feelings of loneliness, Higher crime rates	A combination of urban and rural living	5	Yes, younger parental age improves children's mental health	1	Yes, a younger age (up to 21) may negatively affect her mental health	1	Raising awareness of mental health through campaigns, Greater support in schools and universities	Always
24	2	Pardubice Region	3	0	0	Social media and technology, Study or work pressure, Loneliness and social isolation	Mixed (on the outskirts of a city or in small towns)	Easier access to medical and mental health care, Greater social and cultural opportunities (cinemas, concerts, cafés, etc.), Accessibility of employment and education	Higher levels of stress and pace of life, More noise and pollution, Higher crime rates	A combination of urban and rural living	5	Yes, older parental age improves children's mental health	2	Yes, a younger age (up to 21) may negatively affect her mental health	2	Raising awareness of mental health through campaigns, Greater support in schools and universities	Occasionally
22	1	Olomouc Region	4	0	0	Social media and technology, Study or work pressure, Loneliness and social isolation	Mixed (on the outskirts of a city or in small towns)	Easier access to medical and mental health care, Greater social and cultural opportunities (cinemas, concerts, cafés, etc.), Opportunity to establish more social contacts	Higher levels of stress and pace of life, Lack of nature and quiet places, Greater competitive pressure in the job market	A combination of urban and rural living	5	Yes, older parental age improves children's mental health	2	Yes, a younger age (up to 21) may negatively affect her mental health	3	Creating a better preventive care system	Often
22	1	Moravian-Silesian Region	4	4	2	Social media and technology, Study or work pressure, Loneliness and social isolation	Rural environment	Greater social and cultural opportunities (cinemas, concerts, cafés, etc.), Accessibility of employment and education, Better accessibility of public transport	Higher levels of stress and pace of life, More noise and pollution, Lack of nature and quiet places	In the countryside	5	Yes, older parental age improves children's mental health	2	Yes, a younger age (up to 21) may negatively affect her mental health	2	Improving the affordability of mental health care, Greater support in schools and universities	Often
24	1	South Bohemian Region	4	0	0	Social media and technology, Study or work pressure, Loneliness and social isolation	Rural environment	Greater social and cultural opportunities (cinemas, concerts, cafés, etc.), Accessibility of employment and education, Better accessibility of public transport	Higher levels of stress and pace of life, More noise and pollution, Greater competitive pressure in the job market	In the countryside	5	I don't know	2	Yes, a younger age (up to 21) may negatively affect her mental health	3	Raising awareness of mental health through campaigns, Greater support in schools and universities, Creating a better preventive care system	Occasionally
25	1	Ústí nad Labem Region	3	0	2	Social media and technology, Study or work pressure, Loneliness and social isolation	Urban environment	Easier access to medical and mental health care, Greater social and cultural opportunities (cinemas, concerts, cafés, etc.), Accessibility of employment and education	More noise and pollution, Lack of nature and quiet places, Greater anonymity and feelings of loneliness	In the countryside	5	I don't know	2	Yes, a younger age (up to 21) may negatively affect her mental health	2	Creating a better preventive care system	Often
23	2	Moravian-Silesian Region	3	0	0	Social media and technology, Study or work pressure, Loneliness and social isolation	Mixed (on the outskirts of a city or in small towns)	Greater social and cultural opportunities (cinemas, concerts, cafés, etc.), Accessibility of employment and education, Greater anonymity and privacy	Higher levels of stress and pace of life, More noise and pollution, Lack of nature and quiet places	In the countryside	3	I don't know	2	I don't know	2	Greater support in schools and universities, Creation of a better preventive care system	Occasionally
19	2	Moravian-Silesian Region	3	0	0	Social media and technology, Study or work pressure, Loneliness and social isolation	Urban environment	Greater social and cultural opportunities (cinemas, concerts, cafés, etc.), Accessibility of employment and education, Better accessibility of public transport	Higher levels of stress and pace of life, More noise and pollution, Lack of nature and quiet places	A combination of urban and rural living	3	No, parental age has no effect	1	Yes, a younger age (up to 21) may negatively affect her mental health	3	Improving the affordability of mental health care, Greater support in schools and universities	Occasionally
19	1	Moravian-Silesian Region	3	0	1	Social media and technology, Study or work pressure, Loneliness and social isolation	Urban environment	Greater social and cultural opportunities (cinemas, concerts, cafés, etc.), Accessibility of employment and education, Better accessibility of public transport	More noise and pollution, Lack of nature and quiet places, Higher crime rate	In the countryside	5	No, parental age has no effect	3	No, maternal age has no effect on her mental health	3	Improved access to specialist care (more therapists, shorter waiting times), Improved affordability of mental health care	Often
20	2	Moravian-Silesian Region	5	0	0	Social media and technology, Study or work pressure, Loneliness and social isolation	Rural environment	Greater social and cultural opportunities (cinemas, concerts, cafés, etc.), better access to public transport, opportunity to make more social contacts	Higher levels of stress and pace of life, More noise and pollution, Lack of nature and quiet places	In the countryside	4	I don't know	1	I don't know	2	Improving the affordability of mental health care, Greater support in schools and universities	Occasionally
19	1	Moravian-Silesian Region	3	2	2	Social media and technology, Study or work pressure, Loneliness and social isolation	Urban environment	Greater social and cultural opportunities (cinemas, concerts, cafés, etc.), Accessibility of employment and education, Greater anonymity and privacy	Higher levels of stress and pace of life, Lack of nature and quiet places, Greater competitive pressure in the job market	A combination of urban and rural living	5	Yes, older parental age improves children's mental health	2	Yes, a younger age (up to 21) may negatively affect her mental health	4	Improving access to specialist care (more therapists, shorter waiting times), creating a better preventive care system	Often
20	1	Moravian-Silesian Region	4	0	2	Social media and technology, Study or work pressure, Loneliness and social isolation	Rural environment	Greater social and cultural opportunities (cinemas, concerts, cafés, etc.), better access to public transport, opportunity to make more social contacts	Higher levels of stress and pace of life, More noise and pollution, Lack of nature and quiet places	In the countryside	5	No, parental age has no effect	1	I don't know	2	Raising awareness of mental health through campaigns, Creating a better preventive care system	Often
21	2	Moravian-Silesian Region	3	3	2	Social media and technology, Study or work pressure, Loneliness and social isolation	Urban environment	Easier access to medical and mental health care, Greater social and cultural opportunities (cinemas, concerts, cafés, etc.), Accessibility of employment and education	Higher levels of stress and pace of life, More noise and pollution, Lack of nature and quiet places	A combination of urban and rural living	5	Yes, older parental age improves children's mental health	3	Yes, a younger age (up to 21) may negatively affect her mental health	2	Improving access to professional care (more therapists, shorter waiting times), Raising awareness of mental health through campaigns	Often
20	1	Zlín Region	3	1	1	Social media and technology, Study or work pressure, Loneliness and social isolation	Rural environment	Greater social and cultural opportunities (cinemas, concerts, cafés, etc.), Accessibility of employment and education, Opportunity to establish more social contacts	Higher levels of stress and pace of life, More noise and pollution, Lack of nature and quiet places	A combination of urban and rural living	5	Yes, older parental age improves children's mental health	4	Yes, a younger age (up to 21) may negatively affect her mental health	2	Improving access to professional care (more therapists, shorter waiting times), Raising awareness of mental health through campaigns	Often
22	2	Moravian-Silesian Region	1	0	1	Social media and technology, Study or work pressure, Loneliness and social isolation	Rural environment	Easier access to medical and mental health care, Accessibility of employment and education, Better accessibility of public transport	More noise and pollution, Lack of nature and quiet places, Higher crime rate	I have no proference	2	I don't know	1	I don't know	1	Improved access to specialist care (more therapists, shorter waiting times), Improved affordability of mental health care	Always
20	1	Moravian-Silesian Region	3	0	2	Social media and technology, Study or work pressure, Loneliness and social isolation	Mixed (on the outskirts of a city or in small towns)	Greater social and cultural opportunities (cinemas, concerts, cafés, etc.), better access to public transport, opportunity to make more social contacts	Higher levels of stress and pace of life, More noise and pollution, Lack of nature and quiet places	In the countryside	4	Yes, older parental age improves children's mental health	2	Yes, a younger age (up to 21) may negatively affect her mental health	3	Improving access to specialist care (more therapists, shorter waiting times), creating a better preventive care system	Occasionally
21	1	Olomouc Region	4	3	2	Social media and technology, Study or work pressure, Loneliness and social isolation	Rural environment	Easier access to medical and mental health care, Accessibility of employment and education, Better accessibility of public transport	Higher levels of stress and pace of life, More noise and pollution, Higher crime rates	In the countryside	4	Yes, older parental age improves children's mental health	3	Yes, a younger age (up to 21) may negatively affect her mental health	2	Improved access to professional care (more therapists, shorter waiting times), Greater support in schools and universities	Often

Age	Gender	Region of Permanent Residence:	Q1. How would you rate your current mental state? (1 = Very poor, 5 = Very good)	Q2. If you sought professional help for your mental health (e.g., psychologist, psychiatrist), how would you rate the quality of these services? (1 = Very dissatisfied, 5 = Very satisfied)	Q3. Do you think mental health care is easily accessible in your city/region?	Q4: Which factors do you think affect the mental health of young people aged 18–25 the most? (select the 3 most important)	Q5. Do you live in an urban or rural environment?	Q6. What benefits of living in the city do you think have a positive impact on mental health? (select the 3 most important)	Q7. What disadvantages of living in the city do you think deteriorate mental health? (select the 3 most important)	Q8. If you could choose, where would you prefer to live in terms of mental health?	Q9. How important is support from family and friends to you in dealing with mental health problems? (1 = Not important, 5 = Very important)	Q10. Do you think the age at which people enter parenthood can affect the mental health of their children?	Q11. How much of an impact do you think crime in your region has on your mental health? (1 = No effect, 5 = Very strong effect)	Q12. Do you think the age of the first-time mother can affect her mental health?	Q13.How would you rate the level of general awareness and education about mental health in secondary schools and colleges? (1 = Very poor, 5 = Very good)	Q14. Which of the following do you think would be most helpful in improving mental health care for young people in your region? (select up to 2 options)	Q15. How often do you feel you have to look after your mental health on your own, without the help of professionals or family?
19	1	Moravian-Silesian Region	4	0	0	Social media and technology, Study or work pressure, Loneliness and social isolation	Rural environment	Easier access to medical and mental health care, Greater social and cultural opportunities (cinemas, concerts, cafés, etc.), Accessibility of employment and education	Higher levels of stress and pace of life, More noise and pollution, Lack of nature and quiet places	In the countryside	4	I don't know	1	I don't know	3	Improving the affordability of mental health care, Creating a better preventive care system	Occasionally
21	1	Pardubice Region	5	0	0	Social media and technology, Study or work pressure, Loneliness and social isolation	Mixed (on the outskirts of a city or in small towns)	Greater social and cultural opportunities (cinemas, concerts, cafés, etc.), Greater anonymity and privacy, Better access to public transport	Higher levels of stress and pace of life, More noise and pollution, Lack of nature and quiet places	I have no proference	5	Yes, older parental age improves children's mental health	3	I don't know	4	Raising awareness of mental health through campaigns, Greater support in schools and universities, Creating a better preventive care system	Occasionally
21	1	Moravian-Silesian Region	3	0	0	Social media and technology, Study or work pressure, Loneliness and social isolation	Mixed (on the outskirts of a city or in small towns)	Greater social and cultural opportunities (cinemas, concerts, cafés, etc.), Accessibility of employment and education, Better accessibility of public transport	Higher levels of stress and pace of life, More noise and pollution, Lack of nature and quiet places	A combination of urban and rural living	5	I don't know	3	Yes, a younger age (up to 21) may negatively affect her mental health	2	Greater support in schools and universities, Creation of a better preventive care system	Often
21	1	Moravian-Silesian Region	3	0	0	Social media and technology, Study or work pressure, Loneliness and social isolation	Mixed (on the outskirts of a city or in small towns)	Accessibility of employment and education, Greater anonymity and privacy, Opportunity to establish more social contacts	Higher levels of stress and pace of life, Lack of nature and quiet places, Higher crime rates	A combination of urban and rural living	5	I don't know	4	Yes, a younger age (up to 21) may negatively affect her mental health	2	Greater support in schools and universities, Creation of a better preventive care system	Often
22	2	Moravian-Silesian Region	5	0	0	Social media and technology, Study or work pressure, Loneliness and social isolation	Urban environment	Greater social and cultural opportunities (cinemas, concerts, cafés, etc.), Accessibility of employment and education, Greater anonymity and privacy	Higher levels of stress and pace of life, More noise and pollution, Higher crime rates	In a city	5	No, parental age has no effect	4	Yes, a younger age (up to 21) may negatively affect her mental health	2	Raising awareness of mental health through campaigns, Greater support in schools and universities	Never
22	2	Moravian-Silesian Region	3	0	0	Social media and technology, Study or work pressure, Loneliness and social isolation	Mixed (on the outskirts of a city or in small towns)	Greater anonymity and privacy, Better access to public transport, Opportunity to establish more social contacts	Higher levels of stress and pace of life, More noise and pollution, Lack of nature and quiet places	A combination of urban and rural living	3	I don't know	4	Yes, a younger age (up to 21) may negatively affect her mental health	3	Improved access to specialist care (more therapists, shorter waiting times), Improved affordability of mental health care	Never
20	1	Moravian-Silesian Region	3	0	2	Social media and technology, Study or work pressure, Loneliness and social isolation	Urban environment		Higher levels of stress and pace of life, Lack of nature and quiet places, Greater competitive pressure in the job market	A combination of urban and rural living	5	I don't know	1	Yes, a younger age (up to 21) may negatively affect her mental health	1	Improved access to professional care (more therapists, shorter waiting times), Greater support in schools and universities	Often
22	2	Moravian-Silesian Region	3	0	2	Social media and technology, Study or work pressure, Loneliness and social isolation	Rural environment	Greater social and cultural opportunities (cinemas, concerts, cafés, etc.), Accessibility of employment and education, Opportunity to establish more social contacts	More noise and pollution, Lack of nature and quiet places	In a city	4	No, parental age has no effect	2	Yes, a younger age (up to 21) may negatively affect her mental health	2	Raising awareness of mental health through campaigns, Greater support in schools and universities	Often
26	1	Moravian-Silesian Region	4	0	0	Social media and technology, Study or work pressure, Loneliness and social isolation	Rural environment	Greater social and cultural opportunities (cinemas, concerts, cafés, etc.), Accessibility of employment and education, Better accessibility of public transport		In the countryside	4	Yes, older parental age improves children's mental health	1	I don't know	2	Raising awareness of mental health through campaigns, Creating a better preventive care system	Occasionally
21	2	Olomouc Region	4	0	0	Social media and technology, Study or work pressure, Loneliness and social isolation	Mixed (on the outskirts of a city or in small towns)	Easier access to medical and mental health care, Greater social and cultural opportunities (cinemas, concerts, cafés, etc.), Opportunity to establish more social contacts	Higher levels of stress and pace of life, Lack of nature and quiet places, Greater anonymity and feelings of loneliness	A combination of urban and rural living	4	I don't know	1	Yes, a younger age (up to 21) may negatively affect her mental health	2	Raising awareness of mental health through campaigns, Greater support in schools and universities	Occasionally
19	1	Moravian-Silesian Region	3	4	0	Social media and technology, Study or work pressure, Loneliness and social isolation	Urban environment	Easier access to medical and mental health care, Greater social and cultural opportunities (cinemas, concerts, cafés, etc.), Opportunity to establish more social contacts	Higher levels of stress and pace of life, More noise and pollution, Greater competitive pressure in the job market	I have no proference	5	No, parental age has no effect	2	I don't know	1	Improving the affordability of mental health care, Raising awareness of mental health through campaigns, Greater support in schools and universities	Occasionally
22	1	Moravian-Silesian Region	5	0	0	Social media and technology, Study or work pressure, Loneliness and social isolation	Rural environment	Greater social and cultural opportunities (cinemas, concerts, cafés, etc.), Accessibility of employment and education, Opportunity to establish more social contacts	More noise and pollution, Lack of nature and quiet places, Greater anonymity and feelings of loneliness	In the countryside	5	No, parental age has no effect	2	No, maternal age has no effect on her mental health	2	Greater support in schools and universities, Creation of a better preventive care system	Occasionally
19	1	South Moravian Region	3	4	1	Social media and technology, Study or work pressure, Loneliness and social isolation	Mixed (on the outskirts of a city or in small towns)	Greater anonymity and privacy, Better access to public transport	More noise and pollution, Lack of nature and quiet places	A combination of urban and rural living	4	Yes, older parental age improves children's mental health	2	Yes, a younger age (up to 21) may negatively affect her mental health	2	Improved access to professional care (more therapists, shorter waiting times), Greater support in schools and universities	Often
26	1	Vysočina Region	5	5	2	Social media and technology, Study or work pressure, Loneliness and social isolation	Rural environment	Greater social and cultural opportunities (cinemas, concerts, cafés, etc.), Greater anonymity and privacy, Opportunity to establish more social contacts		A combination of urban and rural living	5	No, parental age has no effect	1	Yes, an older age (over 21) may positively affect her mental health	3	Improved access to specialist care (more therapists, shorter waiting times), Improved affordability of mental health care	Occasionally
22	1	South Moravian Region	2	3	2	Social media and technology, Study or work pressure, Loneliness and social isolation	Mixed (on the outskirts of a city or in small towns)	Easier access to medical and mental health care, Greater social and cultural opportunities (cinemas, concerts, cafés, etc.), Accessibility of employment and education	Higher levels of stress and pace of life, Greater anonymity and feelings of loneliness, Greater competitive pressure in the job market	A combination of urban and rural living	5	Yes, older parental age improves children's mental health	2	Yes, a younger age (up to 21) may negatively affect her mental health	2	Improved access to specialist care (more therapists, shorter waiting times), Improved affordability of mental health care	Always
25	2	Vysočina Region	3	0	1	Social media and technology, Study or work pressure, Loneliness and social isolation	Urban environment	Easier access to medical and mental health care, Greater social and cultural opportunities (cinemas, concerts, cafés, etc.), Accessibility of employment and education		A combination of urban and rural living	5	No, parental age has no effect	2	I don't know	3	Improving access to specialist care (more therapists, shorter waiting times), creating a better preventive care system	Often
21	2	Karlovy Vary Region	2	0	0	Social media and technology, Study or work pressure, Loneliness and social isolation	Urban environment	Greater social and cultural opportunities (cinemas, concerts, cafés, etc.), Accessibility of employment and education, Opportunity to establish more social contacts	More noise and pollution, Lack of nature and quiet places, Greater competitive pressure in the job market	I have no proference	1	I don't know	1	I don't know	4	Raising awareness of mental health through campaigns, Greater support in schools and universities	Always
20	2	Hradec Králové Region	3	4	2	Social media and technology, Study or work pressure, Loneliness and social isolation	Urban environment	Greater social and cultural opportunities (cinemas, concerts, cafés, etc.), better access to public transport, opportunity to make more social contacts		In a city	5	Yes, older parental age improves children's mental health	2	Yes, a younger age (up to 21) may negatively affect her mental health	2	Improving access to specialist care (more therapists, shorter waiting times), creating a better preventive care system	Occasionally
22	1	South Moravian Region	4	0	0	Social media and technology, Study or work pressure, Loneliness and social isolation	Urban environment	Easier access to medical and mental health care, Greater social and cultural opportunities (cinemas, concerts, cafés, etc.), Accessibility of employment and education	Higher levels of stress and pace of life, Greater anonymity and feelings of loneliness, Greater competitive pressure in the job market	In a city	5	I don't know	3	Yes, a younger age (up to 21) may negatively affect her mental health	1	Raising awareness of mental health through campaigns, Greater support in schools and universities	Always
21	1	Zlín Region	3	3	1	Social media and technology, Study or work pressure, Loneliness and social isolation	Urban environment	Accessibility of employment and education, Greater anonymity and privacy, Better accessibility of public transport	Higher levels of stress and pace of life, More noise and pollution, Lack of nature and quiet places	In the countryside	5	No, parental age has no effect	2	Yes, an older age (over 21) may positively affect her mental health	1	Improved access to specialist care (more therapists, shorter waiting times), Improved affordability of mental health care	Often
26	1	South Bohemian Region	4	0	0	Social media and technology, Study or work pressure, Loneliness and social isolation	Urban environment		More noise and pollution, Lack of nature and quiet places, Greater anonymity and feelings of loneliness	A combination of urban and rural living	4	I don't know	3	Yes, a younger age (up to 21) may negatively affect her mental health	4	Improving access to specialist care (more therapists, shorter waiting times), creating a better preventive care system	Often
20	2	South Moravian Region	5	3	2	Social media and technology, Study or work pressure, Loneliness and social isolation	Mixed (on the outskirts of a city or in small towns)	Greater social and cultural opportunities (cinemas, concerts, cafés, etc.), Accessibility of employment and education, Opportunity to establish more social contacts	Higher levels of stress and pace of life, Lack of nature and quiet places, Greater anonymity and feelings of loneliness	A combination of urban and rural living	3	No, parental age has no effect	4	No, maternal age has no effect on her mental health	2	Improved access to specialist care (more therapists, shorter waiting times), Improved affordability of mental health care	Often
20	2	South Moravian Region	4	0	2	Social media and technology, Study or work pressure, Loneliness and social isolation	Mixed (on the outskirts of a city or in small towns)	Greater social and cultural opportunities (cinemas, concerts, cafés, etc.), Greater anonymity and privacy, Opportunity to establish more social contacts	Higher levels of stress and pace of life, Higher crime rates, Greater competitive pressure in the labor market	A combination of urban and rural living	3	I don't know	1	Yes, a younger age (up to 21) may negatively affect her mental health	2	Greater support in schools and universities, Creation of a better preventive care system	Occasionally
19	1	Zlín Region	5	0	0	Social media and technology, Study or work pressure, Loneliness and social isolation	Urban environment	Greater social and cultural opportunities (cinemas, concerts, cafés, etc.), Accessibility of employment and education, Better accessibility of public transport	Higher levels of stress and pace of life, More noise and pollution, Lack of nature and quiet places	In a city	4	I don't know	2	Yes, a younger age (up to 21) may negatively affect her mental health	3	Improved access to specialist care (more therapists, shorter waiting times)	Occasionally
19	2	Central Bohemian Region	3	0	0	Social media and technology, Study or work pressure, Loneliness and social isolation	Urban environment	Greater social and cultural opportunities (cinemas, concerts, cafés, etc.), better access to public transport, opportunity to make more social contacts	Higher levels of stress and pace of life, More noise and pollution, Lack of nature and quiet places	In the countryside	3	Yes, older parental age improves children's mental health	1	I don't know	3		Often
20	1	Moravian-Silesian Region	4	0	2	Social media and technology, Study or work pressure, Loneliness and social isolation	Urban environment	Greater social and cultural opportunities (cinemas, concerts, cafés, etc.), Accessibility of employment and education, Opportunity to establish more social contacts	Higher levels of stress and	In a city	5	Yes, older parental age improves children's mental health	1	Yes, a younger age (up to 21) may negatively affect her mental health	2	Improving the affordability of mental health care, Greater support in schools and universities, Creating a better preventive care system	Occasionally
20	1	Zlín Region	3	0	2	Social media and technology, Study or work pressure, Loneliness and social isolation	Urban environment	Greater social and cultural opportunities (cinemas, concerts, cafés, etc.), Accessibility of employment and education, Opportunity to establish more social contacts	Higher levels of stress and pace of life, Lack of nature and quiet places, Greater competitive pressure in the job market	A combination of urban and rural living	5	I don't know	2	Yes, a younger age (up to 21) may negatively affect her mental health	2	Greater support in schools and universities, Creation of a better preventive care system	Often
20	1	Pardubice Region	4	0	0	Social media and technology, Study or work pressure, Loneliness and social isolation	Rural environment	Greater social and cultural opportunities (cinemas, concerts, cafés, etc.), Accessibility of employment and education, Opportunity to establish more social contacts	More noise and pollution, Lack of nature and quiet places, Greater anonymity and feelings of loneliness	A combination of urban and rural living	5	I don't know	1	Yes, an older age (over 21) may positively affect her mental health	3	Raising awareness of mental health through campaigns, Creating a better preventive care system	Occasionally
21	2	South Moravian Region	4	0	2	Social media and technology, Study or work pressure, Loneliness and social isolation	Urban environment	Greater social and cultural opportunities (cinemas, concerts, cafés, etc.), Accessibility of employment and education, Opportunity to establish more social contacts	Higher levels of stress and pace of life, More noise and pollution, Lack of nature and quiet places	A combination of urban and rural living	3	Yes, older parental age improves children's mental health	2	Yes, an older age (over 21) may positively affect her mental health	4	Raising awareness of mental health through campaigns, Creating a better preventive care system	Often
21	1	Central Bohemian Region	2	2	1	Social media and technology, Study or work pressure, Loneliness and social isolation	Urban environment	Greater social and cultural opportunities (cinemas, concerts, cafés, etc.), better access to public transport, opportunity to make more social contacts	Higher levels of stress and pace of life, More noise and pollution, Greater anonymity and feelings of loneliness	A combination of urban and rural living	5	Yes, younger parental age improves children's mental health	1	Yes, an older age (over 21) may positively affect her mental health	2	Improved access to specialist care (more therapists, shorter waiting times), improved affordability of mental health care, greater support in schools and universities	Often
21	1	Ústí nad Labem Region	3	0	0	Social media and technology, Study or work pressure, Loneliness and social isolation	Urban environment	Greater social and cultural opportunities (cinemas, concerts, cafés, etc.), Greater anonymity and privacy, Better access to public transport	Higher levels of stress and pace of life, greater competitive pressure in the labor market	A combination of urban and rural living	4	Yes, younger parental age improves children's mental health	1	No, maternal age has no effect on her mental health	2	Improving access to specialist care (more therapists, shorter waiting times), improving the affordability of mental health care, creating a better preventive care system	Occasionally
25	2	Central Bohemian Region	4	5	2	Social media and technology, Study or work pressure, Loneliness and social isolation	Mixed (on the outskirts of a city or in small towns)	Easier access to medical and mental health care, Greater social and cultural opportunities (cinemas, concerts, cafés, etc.), Accessibility of employment and education	Higher levels of stress and pace of life, Lack of nature and quiet places, Greater competitive pressure in the job market	A combination of urban and rural living	5	I don't know	3	I don't know	2	Improved access to specialist care (more therapists, shorter waiting times), Improved affordability of mental health care	Occasionally
18	1	South Moravian Region	4	0	2	Social media and technology, Study or work pressure, Loneliness and social isolation	Urban environment	Easier access to medical and mental health care, Greater social and cultural opportunities (cinemas, concerts, cafés, etc.), Greater anonymity and privacy	Higher levels of stress and pace of life, Lack of nature and quiet places, Greater competitive pressure in the job market	A combination of urban and rural living	4	I don't know	3	Yes, a younger age (up to 21) may negatively affect her mental health	3	Improving the affordability of mental health care, Raising awareness of mental health through campaigns	Never
21	1	South Moravian Region	3	4	2	Social media and technology, Study or work pressure, Loneliness and social isolation	Urban environment	Easier access to medical and mental health care, Greater social and cultural opportunities (cinemas, concerts, cafés, etc.), Opportunity to establish more social contacts	Higher levels of stress and pace of life, Lack of nature and quiet places, Greater anonymity and feelings of loneliness	A combination of urban and rural living	4	Yes, older parental age improves children's mental health	1	Yes, a younger age (up to 21) may negatively affect her mental health	2	Improving the affordability of mental health care, Raising awareness of mental health through campaigns	Occasionally
21	1	Moravian-Silesian Region	3	4	2	Social media and technology, Study or work pressure, Loneliness and social isolation	Urban environment	Easier access to medical and mental health care, Greater social and cultural opportunities (cinemas, concerts, cafés, etc.), Opportunity to establish more social contacts	Higher levels of stress and pace of life, Lack of nature and quiet places, Higher crime rates	In the countryside	5	Yes, younger parental age improves children's mental health	2	Yes, a younger age (up to 21) may negatively affect her mental health	2	Improving access to specialist care (more therapists, shorter waiting times), improving the affordability of mental health care, raising awareness of mental health through campaigns	Often
20	1	Moravian-Silesian Region	4	3	2	Social media and technology, Study or work pressure, Loneliness and social isolation	Mixed (on the outskirts of a city or in small towns)	Greater social and cultural opportunities (cinemas, concerts, cafés, etc.), Accessibility of employment and education, Better accessibility of public transport	Higher levels of stress and pace of life, More noise and pollution, Lack of nature and quiet places	In the countryside	4	Yes, younger parental age improves children's mental health	1	No, maternal age has no effect on her mental health	2	Improving the affordability of mental health care, Greater support in schools and universities	Occasionally
22	2	Moravian-Silesian Region	2	4	0	Social media and technology, Study or work pressure, Loneliness and social isolation	Urban environment	Greater social and cultural opportunities (cinemas, concerts, cafés, etc.), Accessibility of employment and education, Better accessibility of public transport	Higher levels of stress and pace of life, More noise and pollution, Higher crime rates	A combination of urban and rural living	4	No, parental age has no effect	1	No, maternal age has no effect on her mental health	2	Greater support in schools and universities, Creation of a better preventive care system	Often
21	2	Moravian-Silesian Region	5	0	3	Social media and technology, Study or work pressure, Loneliness and social isolation	Mixed (on the outskirts of a city or in small towns)	Easier access to medical and mental health care, Greater social and cultural opportunities (cinemas, concerts, cafés, etc.), Accessibility of employment and education	Higher levels of stress and pace of life, Lack of nature and quiet places, Higher crime rates	A combination of urban and rural living	5	Yes, younger parental age improves children's mental health	1	No, maternal age has no effect on her mental health	3	Raising awareness of mental health through campaigns, Greater support in schools and universities	Often
23	1	Moravian-Silesian Region	4	0	0	Social media and technology, Study or work pressure, Loneliness and social isolation	Urban environment	Greater social and cultural opportunities (cinemas, concerts, cafés, etc.), Accessibility of employment and education, Greater anonymity and privacy	Higher levels of stress and pace of life, More noise and pollution, Higher crime rates	A combination of urban and rural living	4	I don't know	4	I don't know	2	Greater support in schools and universities, Creation of a better preventive care system	Often
22	1	Moravian-Silesian Region	3	0	2	Social media and technology, Study or work pressure, Loneliness and social isolation	Rural environment	Easier access to medical and mental health care, Accessibility of employment and education, Better accessibility of public transport	Higher levels of stress and pace of life, More noise and pollution, Lack of nature and quiet places	In the countryside	5	Yes, younger parental age improves children's mental health	4	Yes, a younger age (up to 21) may negatively affect her mental health	2	Improved access to professional care (more therapists, shorter waiting times), Greater support in schools and universities	Often
22	1	South Moravian Region	2	0	2	Social media and technology, Study or work pressure, Loneliness and social isolation	Mixed (on the outskirts of a city or in small towns)	Greater social and cultural opportunities (cinemas, concerts, cafés, etc.), Accessibility of employment and education, Greater anonymity and privacy	Higher levels of stress and pace of life, Lack of nature and quiet places, Higher crime rates	A combination of urban and rural living	5	Yes, older parental age improves children's mental health	4	Yes, a younger age (up to 21) may negatively affect her mental health	3	Raising awareness of mental health through campaigns, Greater support in schools and universities	Often
22	2	South Moravian Region	4	3	3	Social media and technology, Study or work pressure, Loneliness and social isolation	Urban environment	Greater social and cultural opportunities (cinemas, concerts, cafés, etc.), Accessibility of employment and education, Better accessibility of public transport	More noise and pollution, Lack of nature and quiet places, Greater anonymity and feelings of loneliness	A combination of urban and rural living	3	I don't know	1	I don't know	3	-	Often
20	2	South Moravian Region	5	0	0	Social media and technology, Study or work pressure, Loneliness and social isolation, Inadequate access to healthcare	Urban environment	Easier access to medical and mental health care, Greater social and cultural opportunities (cinemas, concerts, cafés, etc.), Accessibility of employment and education, Better accessibility of public transport, Opportunity to establish more social contacts	Lack of nature and quiet places, Higher crime rate, Greater competitive pressure in the labor market	A combination of urban and rural living	5	I don't know	3	Yes, a younger age (up to 21) may negatively affect her mental health	3	Improving access to specialist care (more therapists, shorter waiting times), improving the affordability of mental health care, raising awareness of mental health through campaigns, greater support in schools and universities	Occasionally
22	2	Vysočina Region	4	0	0	Social media and technology, Study or work pressure, Loneliness and social isolation, Thinking about the future, I won't find a job, I won't have anyone to start a family with, I won't be able to take care of a family... but I also see environmental factors lately, e.g.: I can't have a child [illegible]	Rural environment	Greater social and cultural opportunities (cinemas, concerts, cafés, etc.), Accessibility of employment and education, It doesn't have many advantages for me	More noise and pollution, Lack of nature and quiet places, Greater anonymity and feelings of loneliness, Higher crime rates	In the countryside	3	I don't know	1	Yes, a younger age (up to 21) may negatively affect her mental health	3	Improving the affordability of mental health care, Raising awareness of mental health through campaigns, Greater support in schools and universities	Often
19	2	Vysočina Region	4	0	0		Mixed (on the outskirts of a city or in small towns)	Opportunity to establish more social contacts	More noise and pollution, Lack of nature and quiet places, Greater anonymity and feelings of loneliness	A combination of urban and rural living	3	Yes, older parental age improves children's mental health	1	Yes, a younger age (up to 21) may negatively affect her mental health	2	Ban on social media for children under 15. It is very important for parents to limit their children's use of computers, cell phones, etc., which provide more "dopamine" than playing in the real world. Mandatory (semi-mandatory) clubs for socialization within the school.	Always
22	2	Zlín Region	3	5	1	Social media and technology, Study or work pressure, Family relationships	Urban environment	Greater social and cultural opportunities (cinemas, concerts, cafés, etc.), Accessibility of employment and education, Greater anonymity and privacy	Higher levels of stress and pace of life, More noise and pollution, Lack of nature and quiet places	In the countryside	3	No, parental age has no effect	2	No, maternal age has no effect on her mental health	2		Occasionally
19	1	Moravian-Silesian Region	5	0	1	Social media and technology, Study or work pressure, Family relationships	Rural environment	Greater social and cultural opportunities (cinemas, concerts, cafés, etc.), better access to public transport, -	Lack of nature and quiet places, Higher crime rate, -	In the countryside	5	No, parental age has no effect	3	Yes, a younger age (up to 21) may negatively affect her mental health	1	Don't spend time on social media, but go for a run, for example.	Occasionally
19	1	Moravian-Silesian Region	4	0	2	Social media and technology, Study or work pressure, Family relationships	Urban environment	Accessibility of employment and education, Greater anonymity and privacy, Opportunity to establish more social contacts	Higher levels of stress and pace of life, Lack of nature and quiet places, Greater competitive pressure in the job market	A combination of urban and rural living	4	Yes, older parental age improves children's mental health	3	Yes, a younger age (up to 21) may negatively affect her mental health	4	Improving access to specialist care (more therapists, shorter waiting times), creating a better preventive care system	Occasionally

Age	Gender	Region of Permanent Residence:	Q1. How would you rate your current mental state? (1 = Very poor, 5 = Very good)	Q2. If you sought professional help for your mental health (e.g., psychologist, psychiatrist), how would you rate the quality of these services? (1 = Very dissatisfied, 5 = Very satisfied)	Q3. Do you think mental health care is easily accessible in your city/region?	Q4: Which factors do you think affect the mental health of young people aged 18–25 the most? (select the 3 most important)	Q5. Do you live in an urban or rural environment?	Q6. What benefits of living in the city do you think have a positive impact on mental health? (select the 3 most important)	Q7. What disadvantages of living in the city do you think deteriorate mental health? (select the 3 most important)	Q8. If you could choose, where would you prefer to live in terms of mental health?	Q9. How important is support from family and friends to you in dealing with mental health problems? (1 = Not important, 5 = Very important)	Q10. Do you think the age at which people enter parenthood can affect the mental health of their children?	Q11. How much of an impact do you think crime in your region has on your mental health? (1 = No effect, 5 = Very strong effect)	Q12. Do you think the age of the first-time mother can affect her mental health?	Q13.How would you rate the level of general awareness and education about mental health in secondary schools and colleges? (1 = Very poor, 5 = Very good)	Q14. Which of the following do you think would be most helpful in improving mental health care for young people in your region? (select up to 2 options)	Q15. How often do you feel you have to look after your mental health on your own, without the help of professionals or family?
20	2	Moravian-Silesian Region	4	0	2	Social media and technology, Study or work pressure, Family relationships	Mixed (on the outskirts of a city or in small towns)	Easier access to medical and mental health care, Greater social and cultural opportunities (cinemas, concerts, cafés, etc.), Accessibility of employment and education	More noise and pollution, Lack of nature and quiet places, Greater anonymity and feelings of loneliness	A combination of urban and rural living	5	No, parental age has no effect	2	I don't know	3	Greater support in schools and universities, Creation of a better preventive care system	Occasionally
26	1	Central Bohemian Region	4	3	2	Social media and technology, Study or work pressure, Family relationships	Mixed (on the outskirts of a city or in small towns)	Greater social and cultural opportunities (cinemas, concerts, cafés, etc.), Accessibility of employment and education, Greater anonymity and privacy	Higher levels of stress and pace of life, Lack of nature and quiet places, Greater competitive pressure in the job market	A combination of urban and rural living	4	Yes, younger parental age improves children's mental health	1	Yes, a younger age (up to 21) may negatively affect her mental health	3	Improved access to specialist care (more therapists, shorter waiting times), Improved affordability of mental health care	Often
26	1	Moravian-Silesian Region	4	5	2	Social media and technology, Study or work pressure, Family relationships	Rural environment	Greater social and cultural opportunities (cinemas, concerts, cafés, etc.), Accessibility of employment and education, Opportunity to establish more social contacts	Higher levels of stress and pace of life, More noise and pollution, Lack of nature and quiet places	In the countryside	4	No, parental age has no effect	2	I don't know	2	Improving access to professional care (more therapists, shorter waiting times), Raising awareness of mental health through campaigns	Occasionally
19	1	Olomouc Region	2	0	0	Social media and technology, Study or work pressure, Family relationships	Rural environment	Greater social and cultural opportunities (cinemas, concerts, cafés, etc.), Greater anonymity and privacy, Better access to public transport	Higher levels of stress and pace of life, More noise and pollution, Lack of nature and quiet places	In the countryside	4	Yes, younger parental age improves children's mental health	3	Yes, a younger age (up to 21) may negatively affect her mental health	1	Improved access to professional care (more therapists, shorter waiting times), Greater support in schools and universities	Always
22	1	Moravian-Silesian Region	3	0	0	Social media and technology, Study or work pressure, Family relationships	Mixed (on the outskirts of a city or in small towns)	Greater social and cultural opportunities (cinemas, concerts, cafés, etc.), Accessibility of employment and education, Better accessibility of public transport	Higher levels of stress and pace of life, More noise and pollution, Greater competitive pressure in the job market	In the countryside	5	Yes, older parental age improves children's mental health	2	Yes, a younger age (up to 21) may negatively affect her mental health	3	Improving access to professional care (more therapists, shorter waiting times), Raising awareness of mental health through campaigns, Greater support in schools and universities	Often
22	1	Olomouc Region	2	0	0	Social media and technology, Study or work pressure, Family relationships	Mixed (on the outskirts of a city or in small towns)	Easier access to medical and mental health care, Accessibility of employment and education	Higher levels of stress and pace of life, Lack of nature and quiet places	A combination of urban and rural living	5	Yes, older parental age improves children's mental health	3	Yes, a younger age (up to 21) may negatively affect her mental health	2	Improved access to specialist care (more therapists, shorter waiting times), greater support in schools and universities, creation of a better preventive care system.	Occasionally
22	1	Moravian-Silesian Region	2	0	0	Social media and technology, Study or work pressure, Family relationships	Urban environment	Easier access to medical and mental health care, Accessibility of employment and education	Lack of nature and quiet places	A combination of urban and rural living	5	I don't know	2	No, maternal age has no effect on her mental health	1	Improving access to specialist care (more therapists, shorter waiting times), improving the affordability of mental health care, raising awareness of mental health through campaigns, greater support in schools and universities, creating a better preventive care system.	Always
26	1	Moravian-Silesian Region	3	0	1	Social media and technology, Study or work pressure, Family relationships	Mixed (on the outskirts of a city or in small towns)		Higher levels of stress and pace of life, More noise and pollution, Lack of nature and quiet places	I have no preference	1	Yes, older parental age improves children's mental health	2	Yes, an older age (over 21) may positively affect her mental health	2	Improved access to professional care (more therapists, shorter waiting times), Greater support in schools and universities	Often
21	1	Moravian-Silesian Region	3	0	0	Social media and technology, Study or work pressure, Family relationships	Mixed (on the outskirts of a city or in small towns)	Greater social and cultural opportunities (cinemas, concerts, cafés, etc.), Accessibility of employment and education, Better accessibility of public transport	More noise and pollution, Lack of nature and quiet places, Higher crime rate	A combination of urban and rural living	5	I don't know	3	I don't know	3	Improving access to specialist care (more therapists, shorter waiting times), creating a better preventive care system	Occasionally
19	1	Moravian-Silesian Region	3	0	2	Social media and technology, Study or work pressure, Family relationships	Urban environment	Greater social and cultural opportunities (cinemas, concerts, cafés, etc.), better access to public transport, opportunity to make more social contacts	Higher levels of stress and pace of life, More noise and pollution, Lack of nature and quiet places	A combination of urban and rural living	4	No, parental age has no effect	2	Yes, a younger age (up to 21) may negatively affect her mental health	2	Improving the affordability of mental health care, Greater support in schools and universities	Often
20	1	Moravian-Silesian Region	2	0	2	Social media and technology, Study or work pressure, Family relationships	Mixed (on the outskirts of a city or in small towns)	Greater social and cultural opportunities (cinemas, concerts, cafés, etc.), Accessibility of employment and education, Greater anonymity and privacy	More noise and pollution, Lack of nature and quiet places, Greater competitive pressure in the job market	A combination of urban and rural living	4	I don't know	2	Yes, a younger age (up to 21) may negatively affect her mental health	2	Improved access to professional care (more therapists, shorter waiting times), Greater support in schools and universities	Often
20	1	Moravian-Silesian Region	2	0	0	Social media and technology, Study or work pressure, Family relationships	Rural environment	Greater social and cultural opportunities (cinemas, concerts, cafés, etc.), better access to public transport		In the countryside	5	No, parental age has no effect	1	I don't know	3	Raising awareness of mental health through campaigns	Occasionally
21	1	Olomouc Region	5	0	0	Social media and technology, Study or work pressure, Family relationships	Rural environment	Easier access to medical and mental health care, Greater social and cultural opportunities (cinemas, concerts, cafés, etc.), Accessibility of employment and education	Lack of nature and quiet places, Greater anonymity and feelings of loneliness, Greater competitive pressure in the job market	In the countryside	5	Yes, older parental age improves children's mental health	2	No, maternal age has no effect on her mental health	1	Improving the affordability of mental health care, Creating a better preventive care system	Often
22	1	Zlín Region	3	0	1	Social media and technology, Study or work pressure, Family relationships	Mixed (on the outskirts of a city or in small towns)	Greater social and cultural opportunities (cinemas, concerts, cafés, etc.), Greater anonymity and privacy, Better access to public transport	Higher levels of stress and pace of life, More noise and pollution, Lack of nature and quiet places	A combination of urban and rural living	5	I don't know	2	I don't know	2	Improved access to professional care (more therapists, shorter waiting times), Greater support in schools and universities	Often
22	2	Moravian-Silesian Region	3	5	2	Social media and technology, Study or work pressure, Family relationships	Rural environment	Greater social and cultural opportunities (cinemas, concerts, cafés, etc.), better access to public transport	Higher levels of stress and pace of life, More noise and pollution, Lack of nature and quiet places	In the countryside	5	No, parental age has no effect	1	No, maternal age has no effect on her mental health	1	Raising awareness of mental health through campaigns, Creating a better preventive care system	Occasionally
24	2	Olomouc Region	1	0	1	Social media and technology, Study or work pressure, Family relationships	Rural environment	Easier access to medical and mental health care, Greater social and cultural opportunities (cinemas, concerts, cafés, etc.)	Higher levels of stress and pace of life, More noise and pollution, Lack of nature and quiet places	A combination of urban and rural living	5	Yes, younger parental age improves children's mental health	1	Yes, an older age (over 21) may positively affect her mental health	1	Improving access to professional care (more therapists, shorter waiting times), Raising awareness of mental health through campaigns	Never
23	1	Olomouc Region	4	0	2	Social media and technology, Study or work pressure, Family relationships	Urban environment		Higher levels of stress and pace of life, More noise and pollution, Lack of nature and quiet places, Higher crime rates	A combination of urban and rural living	5	Yes, younger parental age improves children's mental health	1	Yes, a younger age (up to 21) may negatively affect her mental health	2	Improving access to professional care (more therapists, shorter waiting times), Raising awareness of mental health through campaigns, Greater support in schools and universities, Creating a better preventive care system	Occasionally
19	1	Moravian-Silesian Region	2	0	2	Social media and technology, Study or work pressure, Family relationships	Rural environment	Easier access to medical and mental health care, Greater social and cultural opportunities (cinemas, concerts, cafés, etc.), Opportunity to establish more social contacts	Higher levels of stress and pace of life, More noise and pollution, Lack of nature and quiet places	In the countryside	5	I don't know	3	Yes, an older age (over 21) may positively affect her mental health	3	Improving access to professional care (more therapists, shorter waiting times), Raising awareness of mental health through campaigns	Often
20	1	Pardubice Region	2	4	0	Social media and technology, Study or work pressure, Family relationships	Urban environment	Easier access to medical and mental health care, Greater social and cultural opportunities (cinemas, concerts, cafés, etc.), Accessibility of employment and education	Higher levels of stress and pace of life, More noise and pollution, Lack of nature and quiet places	In a city	4	I don't know	2	I don't know	3	Improved access to professional care (more therapists, shorter waiting times), Greater support in schools and universities	Never
19	2	Moravian-Silesian Region	3	0	2	Social media and technology, Study or work pressure, Family relationships	Rural environment	Easier access to medical and mental health care, Greater social and cultural opportunities (cinemas, concerts, cafés, etc.), Opportunity to establish more social contacts	Higher levels of stress and pace of life, Lack of nature and quiet places, Greater anonymity and feelings of loneliness	In the countryside	4	Yes, older parental age improves children's mental health	1	Yes, an older age (over 21) may positively affect her mental health	4	Improving access to specialist care (more therapists, shorter waiting times), creating a better preventive care system	Never
20	1	Zlín Region	4	2	2	Social media and technology, Study or work pressure, Family relationships	Mixed (on the outskirts of a city or in small towns)	Easier access to medical and mental health care, Greater social and cultural opportunities (cinemas, concerts, cafés, etc.), Accessibility of employment and education	Higher levels of stress and pace of life, More noise and pollution, Greater competitive pressure in the job market	In the countryside	5	No, parental age has no effect	1	Yes, a younger age (up to 21) may negatively affect her mental health	2	Improved access to specialist care (more therapists, shorter waiting times), greater support in schools and universities, creation of a better preventive care system.	Occasionally
24	1	Zlín Region	3	0	0	Social media and technology, Study or work pressure, Family relationships	Mixed (on the outskirts of a city or in small towns)	None of the above	None of this makes sense.	A combination of urban and rural living	4	No, parental age has no effect	1	No, maternal age has no effect on her mental health	1	Improved access to professional care (more therapists, shorter waiting times), Greater support in schools and universities	Always
22	1	Moravian-Silesian Region	2	0	2	Social media and technology, Study or work pressure, Family relationships	Rural environment	Greater social and cultural opportunities (cinemas, concerts, cafés, etc.), Accessibility of employment and education, Greater anonymity and privacy	More noise and pollution, Lack of nature and quiet places, Higher crime rate	A combination of urban and rural living	5	Yes, younger parental age improves children's mental health	2	Yes, a younger age (up to 21) may negatively affect her mental health	2	Improving the affordability of mental health care, Greater support in schools and universities	Always
23	2	Moravian-Silesian Region	4	0	0	Social media and technology, Study or work pressure, Family relationships	Rural environment	Greater social and cultural opportunities (cinemas, concerts, cafés, etc.), Greater anonymity and privacy, Opportunity to establish more social contacts	Lack of nature and quiet places, Greater anonymity and feelings of loneliness, Higher crime rates	I have no preference	5	I don't know	3	I don't know	2	Improved access to professional care (more therapists, shorter waiting times), Greater support in schools and universities	Never
20	2	Capital City of Prague	4	0	0	Social media and technology, Study or work pressure, Family relationships	Urban environment	Greater social and cultural opportunities (cinemas, concerts, cafés, etc.), Accessibility of employment and education, Opportunity to establish more social contacts	Higher levels of stress and pace of life, greater anonymity and feelings of loneliness	In a city	5	I don't know	1	I don't know	2	Creating a better preventive care system	Often
21	2	Capital City of Prague	4	0	2	Social media and technology, Study or work pressure, Family relationships	Urban environment	Greater social and cultural opportunities (cinemas, concerts, cafés, etc.), Accessibility of employment and education, Greater anonymity and privacy	Higher levels of stress and pace of life, More noise and pollution, Lack of nature and quiet places	A combination of urban and rural living	4	I don't know	2	I don't know	2	Greater support in schools and universities, Creation of a better preventive care system	Occasionally
23	1	Moravian-Silesian Region	4	0	0	Social media and technology, Study or work pressure, Family relationships	Urban environment	Greater social and cultural opportunities (cinemas, concerts, cafés, etc.), Accessibility of employment and education, Better accessibility of public transport	Higher levels of stress and pace of life, More noise and pollution, Lack of nature and quiet places	A combination of urban and rural living	4	I don't know	2	Yes, a younger age (up to 21) may negatively affect her mental health	3	Improving the affordability of mental health care, Raising awareness of mental health through campaigns	Occasionally
24	1	Moravian-Silesian Region	4	0	0	Social media and technology, Study or work pressure, Family relationships	Mixed (on the outskirts of a city or in small towns)	Easier access to medical and mental health care, Greater social and cultural opportunities (cinemas, concerts, cafés, etc.), Better public transport accessibility	Higher levels of stress and pace of life, Greater anonymity and feelings of loneliness, Greater competitive pressure in the job market	A combination of urban and rural living	4	Yes, older parental age improves children's mental health	1	Yes, a younger age (up to 21) may negatively affect her mental health	3	Improving the affordability of mental health care, Raising awareness of mental health through campaigns	Often
23	1	South Bohemian Region	3	0	0	Social media and technology, Study or work pressure, Family relationships	Rural environment	Easier access to medical and mental health care, Greater social and cultural opportunities (cinemas, concerts, cafés, etc.), Accessibility of employment and education		In the countryside	4	No, parental age has no effect	1	Yes, a younger age (up to 21) may negatively affect her mental health	1	Improved access to professional care (more therapists, shorter waiting times), Greater support in schools and universities	Often
21	2	Moravian-Silesian Region	5	0	0	Social media and technology, Study or work pressure, Family relationships	Mixed (on the outskirts of a city or in small towns)	Easier access to medical and mental health care, Greater social and cultural opportunities (cinemas, concerts, cafés, etc.)	More noise and pollution, greater anonymity and feelings of loneliness	In the countryside	5	Yes, older parental age improves children's mental health	3	Yes, an older age (over 21) may positively affect her mental health	1	Improving the affordability of mental health care, Raising awareness of mental health through campaigns	Always
21	2	Moravian-Silesian Region	5	0	2	Social media and technology, Study or work pressure, Family relationships	Urban environment		Lack of nature and quiet places, Greater anonymity and feelings of loneliness	A combination of urban and rural living	3	Yes, older parental age improves children's mental health	1	No, maternal age has no effect on her mental health	3	Improved access to specialist care (more therapists, shorter waiting times), improved affordability of mental health care, greater support in schools and universities, creation of a better preventive care system.	Never
25	1	Moravian-Silesian Region	4	0	0	Social media and technology, Study or work pressure, Family relationships	Rural environment	Greater social and cultural opportunities (cinemas, concerts, cafés, etc.), Accessibility of employment and education, Better accessibility of public transport	Higher levels of stress and pace of life, More noise and pollution, Higher crime rates	In the countryside	5	No, parental age has no effect	3	Yes, a younger age (up to 21) may negatively affect her mental health	2	Improved access to professional care (more therapists, shorter waiting times), Greater support in schools and universities	Occasionally
21	2	Moravian-Silesian Region	2	1	1	Social media and technology, Study or work pressure, Family relationships	Mixed (on the outskirts of a city or in small towns)	Easier access to medical and mental health care, Greater social and cultural opportunities (cinemas, concerts, cafés, etc.), Accessibility of employment and education, Better accessibility of public transport, Opportunity to establish more social contacts	Higher levels of stress and pace of life, More noise and pollution, Lack of nature and quiet places, Higher crime rates	In the countryside	4	Yes, older parental age improves children's mental health	3	Yes, a younger age (up to 21) may negatively affect her mental health	2	Improved access to specialist care (more therapists, shorter waiting times), Improved affordability of mental health care	Always
21	1	Moravian-Silesian Region	5	0	0	Social media and technology, Study or work pressure, Family relationships	Urban environment	Greater social and cultural opportunities (cinemas, concerts, cafés, etc.), Accessibility of employment and education, Opportunity to establish more social contacts	Higher levels of stress and pace of life, Lack of nature and quiet places, Greater competitive pressure in the job market	A combination of urban and rural living	4	No, parental age has no effect	1	I don't know	4	Improving the affordability of mental health care, Greater support in schools and universities, Creating a better preventive care system	Occasionally
21	1	Moravian-Silesian Region	3	0	3	Social media and technology, Study or work pressure, Family relationships	Mixed (on the outskirts of a city or in small towns)	Easier access to medical and mental health care, Greater social and cultural opportunities (cinemas, concerts, cafés, etc.), Opportunity to establish more social contacts	More noise and pollution, Lack of nature and quiet places, Greater competitive pressure in the job market	A combination of urban and rural living	3	Yes, older parental age improves children's mental health	1	Yes, a younger age (up to 21) may negatively affect her mental health	3	Improving the affordability of mental health care, Creating a better preventive care system	Occasionally
21	1	Moravian-Silesian Region	4	4	2	Social media and technology, Study or work pressure, Family relationships	Urban environment	Easier access to medical and mental health care, Greater social and cultural opportunities (cinemas, concerts, cafés, etc.), Opportunity to establish more social contacts		I have no preference	3	I don't know	3	I don't know	1	Improved access to specialist care (more therapists, shorter waiting times), greater support in schools and universities, creation of a better preventive care system.	Often
25	1	Moravian-Silesian Region	2	0	2	Social media and technology, Study or work pressure, Family relationships	Urban environment	Greater social and cultural opportunities (cinemas, concerts, cafés, etc.), Accessibility of employment and education, Opportunity to establish more social contacts	Higher levels of stress and pace of life, Lack of nature and quiet places, Higher crime rates	A combination of urban and rural living	4	I don't know	4	No, maternal age has no effect on her mental health	2	Improved access to professional care (more therapists, shorter waiting times), Greater support in schools and universities	Often
19	1	Moravian-Silesian Region	2	0	2	Social media and technology, Study or work pressure, Family relationships	Urban environment	Easier access to medical and mental health care, Greater social and cultural opportunities (cinemas, concerts, cafés, etc.), Greater anonymity and privacy	Higher levels of stress and pace of life, More noise and pollution, Lack of nature and quiet places	A combination of urban and rural living	4	No, parental age has no effect	3	No, maternal age has no effect on her mental health	2	Raising awareness of mental health through campaigns, Greater support in schools and universities	Occasionally
21	1	Olomouc Region	4	0	0	Social media and technology, Study or work pressure, Family relationships	Rural environment	Easier access to medical and mental health care, Greater social and cultural opportunities (cinemas, concerts, cafés, etc.)	Higher levels of stress and pace of life, More noise and pollution, Lack of nature and quiet places	In the countryside	5	I don't know	3	I don't know	2	Greater support in schools and universities, talking about it in family education, etc.	Occasionally
19	2	Moravian-Silesian Region	5	0	0	Social media and technology, Study or work pressure, Family relationships	Rural environment		Higher levels of stress and pace of life, More noise and pollution, Lack of nature and quiet places	In the countryside	5	I don't know	3	Yes, a younger age (up to 21) may negatively affect her mental health	3	Raising awareness of mental health through campaigns, Greater support in schools and universities	Never
21	1	Moravian-Silesian Region	4	0	2	Social media and technology, Study or work pressure, Family relationships	Urban environment	Greater social and cultural opportunities (cinemas, concerts, cafés, etc.), Greater anonymity and privacy, Opportunity to establish more social contacts	Higher levels of stress and pace of life, Higher crime rates, Greater competitive pressure in the labor market	In the countryside	5	No, parental age has no effect	3	No, maternal age has no effect on her mental health	2	Improved access to professional care (more therapists, shorter waiting times), Greater support in schools and universities	Occasionally
19	1	Olomouc Region	4	0	2	Social media and technology, Study or work pressure, Family relationships	Urban environment	Easier access to medical and mental health care, Greater social and cultural opportunities (cinemas, concerts, cafés, etc.), Better public transport accessibility	More noise and pollution, Lack of nature and quiet places, Higher crime rate	In a city	4	Yes, older parental age improves children's mental health	2	Yes, a younger age (up to 21) may negatively affect her mental health	2	Improving the affordability of mental health care, Greater support in schools and universities	Occasionally
20	2	Olomouc Region	5	0	0	Social media and technology, Study or work pressure, Family relationships	Urban environment	Greater social and cultural opportunities (cinemas, concerts, cafés, etc.), Accessibility of employment and education, Better accessibility of public transport	Higher levels of stress and pace of life, More noise and pollution, Lack of nature and quiet places	In a city	4	No, parental age has no effect	3	Yes, a younger age (up to 21) may negatively affect her mental health	2	Improving the affordability of mental health care, Raising awareness of mental health through campaigns	Occasionally
21	1	Moravian-Silesian Region	4	0	0	Social media and technology, Study or work pressure, Family relationships	Urban environment	Greater social and cultural opportunities (cinemas, concerts, cafés, etc.), Accessibility of employment and education, Better accessibility of public transport	More noise and pollution, Lack of nature and quiet places, Higher crime rate	A combination of urban and rural living	5	I don't know	3	Yes, a younger age (up to 21) may negatively affect her mental health	1	Raising awareness of mental health through campaigns, Greater support in schools and universities	Often
26	2	Moravian-Silesian Region	3	4	2	Social media and technology, Study or work pressure, Family relationships	Urban environment	Greater anonymity and privacy	Higher levels of stress and pace of life, More noise and pollution, Lack of nature and quiet places	A combination of urban and rural living	2	Yes, older parental age improves children's mental health	1	Yes, a younger age (up to 21) may negatively affect her mental health	1	Raising awareness of mental health through campaigns, Creating a better preventive care system	Often
20	1	Ústí nad Labem Region	3	0	1	Social media and technology, Study or work pressure, Family relationships	Mixed (on the outskirts of a city or in small towns)	Easier access to medical and mental health care, Greater social and cultural opportunities (cinemas, concerts, cafés, etc.), Opportunity to establish more social contacts	Higher levels of stress and pace of life, More noise and pollution, Lack of nature and quiet places, Greater anonymity and feelings of loneliness	In the countryside	5	No, parental age has no effect	2	Yes, a younger age (up to 21) may negatively affect her mental health	2	Improving access to specialist care (more therapists, shorter waiting times), improving the affordability of mental health care, raising awareness of mental health through campaigns, greater support in schools and universities	Often
21	1	South Moravian Region	3	5	2	Social media and technology, Study or work pressure, Family relationships	Mixed (on the outskirts of a city or in small towns)	Easier access to medical and mental health care, Greater social and cultural opportunities (cinemas, concerts, cafés, etc.), Better public transport accessibility, Opportunity to establish more social contacts	Higher levels of stress and pace of life, More noise and pollution, Lack of nature and quiet places	A combination of urban and rural living	4	I don't know	2	I don't know	3	Improved access to specialist care (more therapists, shorter waiting times), greater support in schools and universities, creation of a better preventive care system.	Often
22	1	South Moravian Region	4	0	2	Social media and technology, Study or work pressure, Family relationships	Urban environment	Easier access to medical and mental health care, Better public transport accessibility, Opportunity to establish more social contacts	Higher levels of stress and pace of life, More noise and pollution, Lack of nature and quiet places	A combination of urban and rural living	5	I don't know	2	Yes, a younger age (up to 21) may negatively affect her mental health	2	Improved access to professional care (more therapists, shorter waiting times), Greater support in schools and universities	Occasionally

Age	Gender	Region of Permanent Residence:	Q1. How would you rate your current mental state? (1 = Very poor, 5 = Very good)	Q2. If you sought professional help for your mental health (e.g., psychologist, psychiatrist), how would you rate the quality of these services? (1 = Very dissatisfied, 5 = Very satisfied)	Q3. Do you think mental health care is easily accessible in your city/region?	Q4: Which factors do you think affect the mental health of young people aged 18–25 the most? (select the 3 most important)	Q5. Do you live in an urban or rural environment?	Q6. What benefits of living in the city do you think have a positive impact on mental health? (select the 3 most important)	Q7. What disadvantages of living in the city do you think deteriorate mental health? (select the 3 most important)	Q8. If you could choose, where would you prefer to live in terms of mental health?	Q9. How important is support from family and friends to you in dealing with mental health problems? (1 = Not important, 5 = Very important)	Q10. Do you think the age at which people enter parenthood can affect the mental health of their children?	Q11. How much of an impact do you think crime in your region has on your mental health? (1 = No effect, 5 = Very strong effect)	Q12. Do you think the age of the first-time mother can affect her mental health?	Q13.How would you rate the level of general awareness and education about mental health in secondary schools and colleges? (1 = Very poor, 5 = Very good)	Q14. Which of the following do you think would be most helpful in improving mental health care for young people in your region? (select up to 2 options)	Q15. How often do you feel you have to look after your mental health on your own, without the help of professionals or family?
20	1	South Moravian Region	5	0	2	Social media and technology, Study or work pressure, Family relationships	Mixed (on the outskirts of a city or in small towns)	Greater social and cultural opportunities (cinemas, concerts, cafés, etc.), Accessibility of employment and education, Opportunity to establish more social contacts	More noise and pollution, Lack of nature and quiet places, Greater competitive pressure in the job market	A combination of urban and rural living	5	Yes, older parental age improves children's mental health	2	No, maternal age has no effect on her mental health	1	Raising awareness of mental health through campaigns, Greater support in schools and universities	Always
19	2	South Moravian Region	4	0	0	Social media and technology, Study or work pressure, Family relationships	Rural environment	Greater social and cultural opportunities (cinemas, concerts, cafés, etc.), Accessibility of employment and education, Better accessibility of public transport		In the countryside	3	I don't know	2	I don't know	3	Greater support in schools and universities, Creation of a better preventive care system	Often
23	2	South Moravian Region	4	5	2	Social media and technology, Study or work pressure, Family relationships	Urban environment	Greater social and cultural opportunities (cinemas, concerts, cafés, etc.), Accessibility of employment and education, Better accessibility of public transport	More noise and pollution, Lack of nature and quiet places, Greater anonymity and feelings of loneliness	A combination of urban and rural living	2	I don't know	1	I don't know	2	Greater support in schools and universities, Creation of a better preventive care system	Often
23	1	Vysočina Region	2	0	0	Social media and technology, Study or work pressure, Family relationships	Rural environment	Greater social and cultural opportunities (cinemas, concerts, cafés, etc.), Accessibility of employment and education, Opportunity to establish more social contacts	Higher levels of stress and pace of life, Lack of nature and quiet places, Greater anonymity and feelings of loneliness	A combination of urban and rural living	3	I don't know	2	Yes, a younger age (up to 21) may negatively affect her mental health	3	Greater support in schools and universities, Creation of a better preventive care system	Always
21	1	Vysočina Region	3	0	2	Social media and technology, Study or work pressure, Family relationships	Urban environment	Easier access to medical and mental health care, Greater social and cultural opportunities (cinemas, concerts, cafés, etc.), Accessibility of employment and education, Better accessibility of public transport, Opportunity to establish more social contacts	Higher levels of stress and pace of life, More noise and pollution	In a city	2	Yes, younger parental age improves children's mental health	1	Yes, a younger age (up to 21) may negatively affect her mental health	1	Improving access to professional care (more therapists, shorter waiting times), Raising awareness of mental health through campaigns, Greater support in schools and universities	Often
19	1	South Moravian Region	4	0	2	Social media and technology, Study or work pressure, Family relationships	Urban environment	Greater social and cultural opportunities (cinemas, concerts, cafés, etc.), Accessibility of employment and education, Better accessibility of public transport	Higher levels of stress and pace of life, Lack of nature and quiet places, Greater competitive pressure in the job market	In a city	4	I don't know	2	I don't know	3	Greater support in schools and universities, Creation of a better preventive care system	Occasionally
26	2	South Bohemian Region	5	0	0	Social media and technology, Study or work pressure, Family relationships	Rural environment	Easier access to medical and mental health care, Greater social and cultural opportunities (cinemas, concerts, cafés, etc.), Accessibility of employment and education	Higher levels of stress and pace of life, lack of nature and quiet places, people in big cities don't have the same opportunities as people in the countryside, where they can work at home in their gardens on their small farms, which is not their main source of income, which in my experience helps to reduce stress, because you're not rushing anywhere and you can clear your head.	A combination of urban and rural living	5	No, parental age has no effect	1	No, maternal age has no effect on her mental health	1	Raising awareness of mental health through campaigns, Greater support in schools and universities, Educating people that improving their condition in the short term with addictive substances (alcohol, marijuana, cocaine, etc.) may ultimately worsen their condition after short-term relief.	Always
19	1	Capital City of Prague	4	0	3	Social media and technology, Study or work pressure, Family relationships	Urban environment	Greater social and cultural opportunities (cinemas, concerts, cafés, etc.), Accessibility of employment and education, Better accessibility of public transport	Higher levels of stress and pace of life, More noise and pollution, Greater anonymity and feelings of loneliness	I have no proference	4	I don't know	3	Yes, a younger age (up to 21) may negatively affect her mental health	4	Improving the affordability of mental health care, Creating a better preventive care system	Often
20	1	South Moravian Region	3	0	1	Social media and technology, Study or work pressure, Family relationships	Mixed (on the outskirts of a city or in small towns)	Greater social and cultural opportunities (cinemas, concerts, cafés, etc.), Accessibility of employment and education, Greater anonymity and privacy	Higher levels of stress and pace of life, Lack of nature and quiet places, Greater competitive pressure in the job market	In the countryside	5	Yes, older parental age improves children's mental health	2	Yes, a younger age (up to 21) may negatively affect her mental health	1	Improving access to specialist care (more therapists, shorter waiting times), creating a better preventive care system	Often
22	1	Moravian-Silesian Region	3	0	2	Social media and technology, Study or work pressure, Family relationships	Rural environment	Greater social and cultural opportunities (cinemas, concerts, cafés, etc.), Greater anonymity and privacy, Better access to public transport	Higher levels of stress and pace of life, More noise and pollution, Lack of nature and quiet places	A combination of urban and rural living	4	Yes, older parental age improves children's mental health	1	Yes, a younger age (up to 21) may negatively affect her mental health	2	Raising awareness of mental health through campaigns, Creating a better preventive care system	Often
21	1	Pardubice Region	4	4	1	Social media and technology, Study or work pressure, Family relationships	Rural environment	Greater social and cultural opportunities (cinemas, concerts, cafés, etc.), Accessibility of employment and education, Better accessibility of public transport	More noise and pollution, Lack of nature and quiet places, Higher crime rate	In the countryside	5	Yes, younger parental age improves children's mental health	2	Yes, a younger age (up to 21) may negatively affect her mental health	3	Improved access to specialist care (more therapists, shorter waiting times), greater support in schools and universities, creation of a better preventive care system.	Occasionally
19	1	South Moravian Region	4	0	0	Social media and technology, Study or work pressure, Family relationships	Urban environment	Easier access to medical and mental health care, Greater social and cultural opportunities (cinemas, concerts, cafés, etc.), Accessibility of employment and education, Better accessibility of public transport	Higher levels of stress and pace of life, More noise and pollution, Lack of nature and quiet places, Greater competitive pressure in the job market	In the countryside	5	No, parental age has no effect	1	No, maternal age has no effect on her mental health	2	Raising awareness of mental health through campaigns, Greater support in schools and universities	Occasionally
20	1	South Bohemian Region	3	0	0	Social media and technology, Study or work pressure, Family relationships	Mixed (on the outskirts of a city or in small towns)	Easier access to medical and mental health care, Greater social and cultural opportunities (cinemas, concerts, cafés, etc.), Accessibility of employment and education, Better accessibility of public transport	Lack of nature and quiet places	A combination of urban and rural living	5	Yes, younger parental age improves children's mental health	2	Yes, an older age (over 21) may positively affect her mental health	5	Greater support in schools and universities	Occasionally
20	1	Olomouc Region	4	0	0	Social media and technology, Study or work pressure, Family relationships	Rural environment	Greater social and cultural opportunities (cinemas, concerts, cafés, etc.), Accessibility of employment and education, Opportunity to establish more social contacts	More noise and pollution, Lack of nature and quiet places, Higher crime rate	A combination of urban and rural living	5	No, parental age has no effect	3	No, maternal age has no effect on her mental health	3	Improving the affordability of mental health care, Raising awareness of mental health through campaigns	Occasionally
22	1	South Moravian Region	4	3	2	Social media and technology, Study or work pressure, Family relationships	Urban environment	Greater social and cultural opportunities (cinemas, concerts, cafés, etc.), Accessibility of employment and education, Opportunity to establish more social contacts	Higher levels of stress and pace of life, More noise and pollution, Greater competitive pressure in the job market	In the countryside	5	Yes, younger parental age improves children's mental health	1	Yes, an older age (over 21) may positively affect her mental health	3	Raising awareness of mental health through campaigns, Greater support in schools and universities	Often
21	1	Pardubice Region	4	4	3	Social media and technology, Study or work pressure, Family relationships	Urban environment	Greater social and cultural opportunities (cinemas, concerts, cafés, etc.), Accessibility of employment and education, Greater anonymity and privacy	Higher levels of stress and	In a city	5	Yes, younger parental age improves children's mental health	2	Yes, a younger age (up to 21) may negatively affect her mental health	2	Improving access to professional care (more therapists, shorter waiting times), Raising awareness of mental health through campaigns, Greater support in schools and universities	Never
22	2	Olomouc Region	4	0	2	Social media and technology, Study or work pressure, Family relationships	Urban environment	Easier access to medical and mental health care, Greater social and cultural opportunities (cinemas, concerts, cafés, etc.), Greater anonymity and privacy	More noise and pollution	In a city	5	No, parental age has no effect	1	I don't know	3	Raising awareness of mental health through campaigns, Greater support in schools and universities, Creating a better preventive care system	Often
22	1	South Bohemian Region	4	0	2	Social media and technology, Study or work pressure, Family relationships	Rural environment	Greater social and cultural opportunities (cinemas, concerts, cafés, etc.), Accessibility of employment and education, Greater anonymity and privacy	Higher levels of stress and pace of life, Lack of nature and quiet places, Greater competitive pressure in the job market	A combination of urban and rural living	5	I don't know	3	Yes, a younger age (up to 21) may negatively affect her mental health	2	Greater support in schools and universities, Creation of a better preventive care system	Occasionally
21	1	South Moravian Region	3	5	2	Social media and technology, Study or work pressure, Family relationships	Urban environment	Easier access to medical and mental health care, Greater social and cultural opportunities (cinemas, concerts, cafés, etc.), Opportunity to establish more social contacts	Higher levels of stress and pace of life, More noise and pollution, Lack of nature and quiet places	A combination of urban and rural living	4	No, parental age has no effect	5	No, maternal age has no effect on her mental health	4	Improved access to specialist care (more therapists, shorter waiting times), Improved affordability of mental health care	Always
22	1	Moravian-Silesian Region	4	0	2	Social media and technology, Study or work pressure, Family relationships	Rural environment	Greater social and cultural opportunities (cinemas, concerts, cafés, etc.), Accessibility of employment and education, Opportunity to establish more social contacts	More noise and pollution, Lack of nature and quiet places, Greater anonymity and feelings of loneliness	In the countryside	5	No, parental age has no effect	3	Yes, a younger age (up to 21) may negatively affect her mental health	3	Improving access to specialist care (more therapists, shorter waiting times), Raising awareness of mental health through campaigns, Creating a better preventive care system	Occasionally
21	1	Zlín Region	2	3	1	Social media and technology, Study or work pressure, Family relationships	Urban environment	Easier access to medical and mental health care, Greater social and cultural opportunities (cinemas, concerts, cafés, etc.), Opportunity to establish more social contacts	More noise and pollution, Lack of nature and quiet places, Greater competitive pressure in the job market	In a city	3	No, parental age has no effect	1	No, maternal age has no effect on her mental health	1	Improved access to specialist care (more therapists, shorter waiting times), Improved affordability of mental health care	Always
19	1	Moravian-Silesian Region	3	2	2	Social media and technology, Study or work pressure, Family relationships, Crime and feeling unsafe	Rural environment	Greater social and cultural opportunities (cinemas, concerts, cafés, etc.), better access to public transport		A combination of urban and rural living	5	Yes, younger parental age improves children's mental health	3	I don't know	3	Raising awareness of mental health through campaigns, Greater support in schools and universities, Creating a better preventive care system	Occasionally
23	1	Moravian-Silesian Region	4	0	3	Social media and technology, Study or work pressure, Family relationships, Crime and feeling unsafe	Rural environment	Greater social and cultural opportunities (cinemas, concerts, cafés, etc.), Accessibility of employment and education, Better accessibility of public transport	Higher levels of stress and pace of life, More noise and pollution, Lack of nature and quiet places, Higher crime rates, Greater competitive pressure in the job market	In the countryside	5	No, parental age has no effect	1	No, maternal age has no effect on her mental health	2	Raising awareness of mental health through campaigns, Greater support in schools and universities	Occasionally
23	1	Moravian-Silesian Region	4	5	2	Social media and technology, Study or work pressure, Family relationships, Crime and feeling unsafe	Urban environment	Greater social and cultural opportunities (cinemas, concerts, cafés, etc.), Accessibility of employment and education, Better accessibility of public transport	Higher levels of stress and pace of life, More noise and pollution, Lack of nature and quiet places, Higher crime rates	In the countryside	5	Yes, older parental age improves children's mental health	5	No, maternal age has no effect on her mental health	2	Zlepšení dostupnosti odborné péče (více terapeutů, kratší čekací doby), Zvýšení povědomí o duševním zdraví prostřednictvím kampaní, Větší podpora na školách a univerzitách, Vytvoření lepšího systému preventivní péče	Often
22	1	South Bohemian Region	5	0	2	Social media and technology, Study or work pressure, Family relationships, Inadequate access to healthcare	Urban environment	Easier access to medical and mental health care, Greater anonymity and privacy	Higher levels of stress and pace of life, More noise and pollution, Greater competitive pressure in the job market	A combination of urban and rural living	4	I don't know	2	Yes, a younger age (up to 21) may negatively affect her mental health	1	Improved access to specialist care (more therapists, shorter waiting times), improved affordability of mental health care, greater support in schools and universities	Always
19	1	Moravian-Silesian Region	4	0	0	Social media and technology, Study or work pressure, Family relationships, Loneliness and social isolation	Mixed (on the outskirts of a city or in small towns)	Greater social and cultural opportunities (cinemas, concerts, cafés, etc.), Accessibility of employment and education, Better accessibility of public transport	Higher levels of stress and pace of life, More noise and pollution, Lack of nature and quiet places, Higher crime rates, Greater competitive pressure in the job market	A combination of urban and rural living	4	I don't know	2	Yes, a younger age (up to 21) may negatively affect her mental health	2	Improving access to professional care (more therapists, shorter waiting times), Raising awareness of mental health through campaigns, Greater support in schools and universities	Often
24	2	Moravian-Silesian Region	4	0	0	Social media and technology, Study or work pressure, Family relationships, Loneliness and social isolation	Mixed (on the outskirts of a city or in small towns)	Greater anonymity and privacy	Higher levels of stress and pace of life, Lack of nature and quiet places, Greater anonymity and feelings of loneliness	In the countryside	3	Yes, older parental age improves children's mental health	2	I don't know	2	Greater support in schools and universities, Creation of a better preventive care system	Often
19	1	Moravian-Silesian Region	3	0	2	Social media and technology, Study or work pressure, Family relationships, Loneliness and social isolation	Rural environment	Easier access to medical and mental health care, Greater social and cultural opportunities (cinemas, concerts, cafés, etc.), Greater anonymity and privacy	Higher levels of stress and pace of life, More noise and pollution, Lack of nature and quiet places	In the countryside	4	I don't know	1	Yes, a younger age (up to 21) may negatively affect her mental health	2	Raising awareness of mental health through campaigns, Greater support in schools and universities, Creating a better preventive care system	Often
20	1	Moravian-Silesian Region	3	0	2	Social media and technology, Study or work pressure, Family relationships, Loneliness and social isolation	Mixed (on the outskirts of a city or in small towns)	Greater social and cultural opportunities (cinemas, concerts, cafés, etc.), better access to public transport	More noise and pollution, higher crime rates	In the countryside	4	Yes, younger parental age improves children's mental health	3	Yes, an older age (over 21) may positively affect her mental health	1	Improving access to professional care (more therapists, shorter waiting times), Raising awareness of mental health through campaigns, Greater support in schools and universities, Creating a better preventive care system	Often
23	1	Moravian-Silesian Region	4	1	0	Social media and technology, Study or work pressure, Family relationships, Loneliness and social isolation	Rural environment	Greater social and cultural opportunities (cinemas, concerts, cafés, etc.), Accessibility of employment and education, Greater anonymity and privacy		A combination of urban and rural living	4	I don't know	3	I don't know	4	Raising awareness of mental health through campaigns	Occasionally
22	1	Moravian-Silesian Region	3	1	1	Social media and technology, Study or work pressure, Family relationships, Loneliness and social isolation	Urban environment	Better access to public transport, Opportunity to establish more social contacts	Higher levels of stress and pace of life, More noise and pollution, Greater anonymity and feelings of loneliness	A combination of urban and rural living	4	No, parental age has no effect	2	No, maternal age has no effect on her mental health	2	Improving access to specialist care (more therapists, shorter waiting times), improving the affordability of mental health care, creating a better preventive care system	Often
19	1	Moravian-Silesian Region	4	0	0	Social media and technology, Study or work pressure, Family relationships, Loneliness and social isolation	Urban environment	Easier access to medical and mental health care, Greater social and cultural opportunities (cinemas, concerts, cafés, etc.), Better public transport accessibility	Lack of nature and quiet places, Greater anonymity and feelings of loneliness	In a city	3	No, parental age has no effect	1	Yes, a younger age (up to 21) may negatively affect her mental health	2	Improving access to specialist care (more therapists, shorter waiting times), improving the affordability of mental health care, creating a better preventive care system	Occasionally
19	1	Moravian-Silesian Region	2	0	0	Social media and technology, Study or work pressure, Family relationships, Loneliness and social isolation	Rural environment	Easier access to medical and mental health care, Greater social and cultural opportunities (cinemas, concerts, cafés, etc.), Accessibility of employment and education, Greater anonymity and privacy	Higher levels of stress and pace of life, Lack of nature and quiet places	A combination of urban and rural living	4	I don't know	1	Yes, a younger age (up to 21) may negatively affect her mental health	2	Improving access to specialist care (more therapists, shorter waiting times), improving the affordability of mental health care, raising awareness of mental health through campaigns, creating a better preventive care system	Often
23	2	Moravian-Silesian Region	5	0	0	Social media and technology, Study or work pressure, Family relationships, Loneliness and social isolation	Mixed (on the outskirts of a city or in small towns)	Accessibility of employment and education, Greater anonymity and privacy, Opportunity to establish more social contacts	Higher levels of stress and pace of life, More noise and pollution, Lack of nature and quiet places	A combination of urban and rural living	1	Yes, younger parental age improves children's mental health	1	No, maternal age has no effect on her mental health	3		Occasionally
20	1	Zlín Region	3	0	1	Social media and technology, Study or work pressure, Family relationships, Loneliness and social isolation	Mixed (on the outskirts of a city or in small towns)	Accessibility of employment and education, Greater anonymity and privacy, Opportunity to establish more social contacts	Lack of nature and quiet places, Greater competitive pressure in the job market	A combination of urban and rural living	5	Yes, younger parental age improves children's mental health	3	No, maternal age has no effect on her mental health	4	Improved access to specialist care (more therapists, shorter waiting times), improved affordability of mental health care, greater support in schools and universities	Always
22	2	Zlín Region	2	3	2	Social media and technology, Study or work pressure, Family relationships, Loneliness and social isolation	Rural environment	Greater social and cultural opportunities (cinemas, concerts, cafés, etc.), Accessibility of employment and education, Opportunity to establish more social contacts	Higher levels of stress and pace of life, More noise and pollution, Lack of nature and quiet places	In the countryside	1	Yes, older parental age improves children's mental health	1	Yes, an older age (over 21) may positively affect her mental health	4	Improved access to specialist care (more therapists, shorter waiting times), Improved affordability of mental health care	Always
24	1	Olomouc Region	3	0	0	Social media and technology, Study or work pressure, Family relationships, Loneliness and social isolation	Rural environment	Greater social and cultural opportunities (cinemas, concerts, cafés, etc.), better access to public transport, opportunity to make more social contacts	Higher levels of stress and pace of life, More noise and pollution, Lack of nature and quiet places	A combination of urban and rural living	3	Yes, younger parental age improves children's mental health	2	Yes, a younger age (up to 21) may negatively affect her mental health	3	Raising awareness of mental health through campaigns, Greater support in schools and universities, Creating a better preventive care system	Often
22	1	Moravian-Silesian Region	4	0	0	Social media and technology, Study or work pressure, Family relationships, Loneliness and social isolation	Mixed (on the outskirts of a city or in small towns)	Easier access to medical and mental health care, Greater social and cultural opportunities (cinemas, concerts, cafés, etc.), Opportunity to establish more social contacts	Higher levels of stress and pace of life, More noise and pollution, Higher crime rates	In the countryside	4	Yes, older parental age improves children's mental health	2	I don't know	3	Improving access to professional care (more therapists, shorter waiting times), Raising awareness of mental health through campaigns	Occasionally
25	1	Olomouc Region	4	0	2	Social media and technology, Study or work pressure, Family relationships, Loneliness and social isolation	Rural environment	Greater social and cultural opportunities (cinemas, concerts, cafés, etc.), greater anonymity and privacy	Higher levels of stress and pace of life, Lack of nature and quiet places, Greater anonymity and feelings of loneliness	In the countryside	5	No, parental age has no effect	1	No, maternal age has no effect on her mental health	3	Greater support in schools and universities	Often
25	1	Moravian-Silesian Region	2	3	2	Social media and technology, Study or work pressure, Family relationships, Loneliness and social isolation	Mixed (on the outskirts of a city or in small towns)		More noise and pollution, Lack of nature and quiet places, Greater competitive pressure in the job market	In the countryside	4	I don't know	3	I don't know	2	Improved access to specialist care (more therapists, shorter waiting times), Improved affordability of mental health care	Always
22	1	Moravian-Silesian Region	1	0	0	Social media and technology, Study or work pressure, Family relationships, Loneliness and social isolation	Rural environment	Access to employment and education, Greater anonymity and privacy		A combination of urban and rural living	5	I don't know	2	I don't know	2	Raising awareness of mental health through campaigns, Greater support in schools and universities, Creating a better preventive care system	Occasionally
19	1	Olomouc Region	4	0	0	Social media and technology, Study or work pressure, Family relationships, Loneliness and social isolation	Rural environment	Easier access to medical and mental health care, Greater social and cultural opportunities (cinemas, concerts, cafés, etc.), Opportunity to establish more social contacts	Higher levels of stress and pace of life, More noise and pollution, Lack of nature and quiet places	A combination of urban and rural living	4	I don't know	2	Yes, a younger age (up to 21) may negatively affect her mental health	3	Raising awareness of mental health through campaigns, Greater support in schools and universities	Occasionally
21	1	Olomouc Region	5	0	0	Social media and technology, Study or work pressure, Family relationships, Loneliness and social isolation	Rural environment	Greater social and cultural opportunities (cinemas, concerts, cafés, etc.), Greater anonymity and privacy, Better access to public transport, Opportunity to make more social contacts	More noise and pollution, Lack of nature and quiet places	In the countryside	4	Yes, older parental age improves children's mental health	2	Yes, a younger age (up to 21) may negatively affect her mental health	3	Raising awareness of mental health through campaigns, Greater support in schools and universities, Creating a better preventive care system	Always
21	1	Moravian-Silesian Region	4	3	2	Social media and technology, Study or work pressure, Family relationships, Loneliness and social isolation	Urban environment			A combination of urban and rural living	5	No, parental age has no effect	3	Yes, a younger age (up to 21) may negatively affect her mental health	1	Improving the affordability of mental health care, Raising awareness of mental health through campaigns, Greater support in schools and universities, Creating a better preventive care system	Often
19	1	Olomouc Region	3	0	0	Social media and technology, Study or work pressure, Family relationships, Loneliness and social isolation	Urban environment	Greater social and cultural opportunities (cinemas, concerts, cafés, etc.), Accessibility of employment and education, Better accessibility of public transport	Higher levels of stress and pace of life, Lack of nature and quiet places, Greater anonymity and feelings of loneliness, Greater competitive pressure in the job market	In a city	5	Yes, younger parental age improves children's mental health	2	Yes, a younger age (up to 21) may negatively affect her mental health	2	Improving access to specialist care (more therapists, shorter waiting times), creating a better preventive care system	Occasionally
21	2	Zlín Region	4	0	0	Social media and technology, Study or work pressure, Family relationships, Loneliness and social isolation	Urban environment	Greater social and cultural opportunities (cinemas, concerts, cafés, etc.), better access to public transport, opportunity to make more social contacts	Higher levels of stress and pace of life, greater competitive pressure in the labor market	A combination of urban and rural living	5	Yes, younger parental age improves children's mental health	2	Yes, a younger age (up to 21) may negatively affect her mental health	3	Improving the affordability of mental health care, Greater support in schools and universities	Occasionally
22	1	Olomouc Region	4	0	1	Social media and technology, Study or work pressure, Family relationships, Loneliness and social isolation	Urban environment	Easier access to medical and mental health care, Greater social and cultural opportunities (cinemas, concerts, cafés, etc.), Accessibility of employment and education, Greater anonymity and privacy, Better accessibility of public transport, Opportunity to establish more social contacts	More noise and pollution, Lack of nature and quiet places	A combination of urban and rural living	5	No, parental age has no effect	2	No, maternal age has no effect on her mental health	2	Raising awareness of mental health through campaigns, Greater support in schools and universities, Creating a better preventive care system	Occasionally
20	1	Pardubice Region	2	0	2	Social media and technology, Study or work pressure, Family relationships, Loneliness and social isolation	Rural environment	Greater social and cultural opportunities (cinemas, concerts, cafés, etc.), Accessibility of employment and education, Opportunity to establish more social contacts		A combination of urban and rural living	5	I don't know	2	No, maternal age has no effect on her mental health	4	Improving access to professional care (more therapists, shorter waiting times), Raising awareness of mental health through campaigns	Occasionally

Age	Gender	Region of Permanent Residence:	Q1. How would you rate your current mental state? (1 = Very poor, 5 = Very good)	Q2. If you sought professional help for your mental health (e.g., psychologist, psychiatrist), how would you rate the quality of these services? (1 = Very dissatisfied, 5 = Very satisfied)	Q3. Do you think mental health care is easily accessible in your city/region?	Q4: Which factors do you think affect the mental health of young people aged 18–25 the most? (select the 3 most important)	Q5. Do you live in an urban or rural environment?	Q6. What benefits of living in the city do you think have a positive impact on mental health? (select the 3 most important)	Q7. What disadvantages of living in the city do you think deteriorate mental health? (select the 3 most important)	Q8. If you could choose, where would you prefer to live in terms of mental health?	Q9. How important is support from family and friends to you in dealing with mental health problems? (1 = Not important, 5 = Very important)	Q10. Do you think the age at which people enter parenthood can affect the mental health of their children?	Q11. How much of an impact do you think crime in your region has on your mental health? (1 = No effect, 5 = Very strong effect)	Q12. Do you think the age of the first-time mother can affect her mental health?	Q13.How would you rate the level of general awareness and education about mental health in secondary schools and colleges? (1 = Very poor, 5 = Very good)	Q14. Which of the following do you think would be most helpful in improving mental health care for young people in your region? (select up to 2 options)	Q15. How often do you feel you have to look after your mental health on your own, without the help of professionals or family?
23	2	Moravian-Silesian Region	2	0	1	Social media and technology, Study or work pressure, Family relationships, Loneliness and social isolation	Urban environment	Greater social and cultural opportunities (cinemas, concerts, cafés, etc.), better access to public transport	Lack of nature and quiet places	In the countryside	4	I don't know	1	Yes, a younger age (up to 21) may negatively affect her mental health	3	Improved access to specialist care (more therapists, shorter waiting times), improved affordability of mental health care, greater support in schools and universities	Often
19	1	South Moravian Region	5	4	3	Social media and technology, Study or work pressure, Family relationships, Loneliness and social isolation	Urban environment	Greater social and cultural opportunities (cinemas, concerts, cafés, etc.), Accessibility of employment and education, Greater anonymity and privacy	Higher levels of stress and pace of life, More noise and pollution, Lack of nature and quiet places	A combination of urban and rural living	5	Yes, older parental age improves children's mental health	2	Yes, a younger age (up to 21) may negatively affect her mental health	1	Greater support in schools and universities, Creation of a better preventive care system	Occasionally
22	1	South Moravian Region	4	0	2	Social media and technology, Study or work pressure, Family relationships, Loneliness and social isolation	Mixed (on the outskirts of a city or in small towns)	Better accessibility of public transport	Higher levels of stress and pace of life, More noise and pollution, Lack of nature and quiet places, Greater anonymity and feelings of loneliness, Higher crime rates, Greater competitive pressure in the job market	A combination of urban and rural living	5	Yes, younger parental age improves children's mental health	1	I don't know	2	Raising awareness of mental health through campaigns, Greater support in schools and universities	Never
21	1	South Moravian Region	2	0	0	Social media and technology, Study or work pressure, Family relationships, Loneliness and social isolation	Mixed (on the outskirts of a city or in small towns)	Greater social and cultural opportunities (cinemas, concerts, cafés, etc.), better access to public transport, opportunity to make more social contacts		A combination of urban and rural living	5	Yes, older parental age improves children's mental health	3	Yes, an older age (over 21) may positively affect her mental health	3	Greater support in schools and universities, Creation of a better preventive care system	Occasionally
21	1	Capital City of Prague	3	0	1	Social media and technology, Study or work pressure, Family relationships, Loneliness and social isolation	Urban environment	Greater social and cultural opportunities (cinemas, concerts, cafés, etc.), better access to public transport	More noise and pollution, Lack of nature and quiet places	In a city	5	No, parental age has no effect	1	Yes, a younger age (up to 21) may negatively affect her mental health	2	Improving the affordability of mental health care, Creating a better preventive care system	Occasionally
21	2	South Moravian Region	4	0	0	Social media and technology, Study or work pressure, Family relationships, Loneliness and social isolation	Urban environment	Access to employment and education, Opportunity to establish more social contacts		A combination of urban and rural living	3	Yes, younger parental age improves children's mental health	1	I don't know	3	Raising awareness of mental health through campaigns, Greater support in schools and universities, Creating a better preventive care system	Often
20	1	South Moravian Region	1	5	2	Social media and technology, Study or work pressure, Family relationships, Loneliness and social isolation	Rural environment	Easier access to medical and mental health care, Greater social and cultural opportunities (cinemas, concerts, cafés, etc.), Accessibility of employment and education	Higher levels of stress and pace of life, More noise and pollution, Lack of nature and quiet places	A combination of urban and rural living	4	No, parental age has no effect	3	Yes, a younger age (up to 21) may negatively affect her mental health	3	Improving the affordability of mental health care, Greater support in schools and universities	Occasionally
20	1	Pardubice Region	4	0	0	Social media and technology, Study or work pressure, Family relationships, Loneliness and social isolation	Urban environment	Easier access to medical and mental health care, Greater social and cultural opportunities (cinemas, concerts, cafés, etc.), Opportunity to establish more social contacts	Higher levels of stress and pace of life, Lack of nature and quiet places, Greater anonymity and feelings of loneliness	In a city	4	No, parental age has no effect	1	Yes, a younger age (up to 21) may negatively affect her mental health	2	Raising awareness of mental health through campaigns, Greater support in schools and universities	Never
21	1	Moravian-Silesian Region	4	0	1	Social media and technology, Study or work pressure, Family relationships, Loneliness and social isolation	Mixed (on the outskirts of a city or in small towns)	Easier access to medical and mental health care, Better availability of public transport	Higher levels of stress and pace of life, More noise and pollution, Lack of nature and quiet places, Greater anonymity and feelings of loneliness	In the countryside	5	Yes, younger parental age improves children's mental health	1	Yes, a younger age (up to 21) may negatively affect her mental health	3	Improving access to specialist care (more therapists, shorter waiting times), improving the affordability of mental health care, raising awareness of mental health through campaigns, creating a better preventive care system	Often
23	1	Vysočina Region	3	5	1	Social media and technology, Study or work pressure, Family relationships, Loneliness and social isolation	Mixed (on the outskirts of a city or in small towns)	Greater social and cultural opportunities (cinemas, concerts, cafés, etc.)	More noise and pollution, Lack of nature and quiet places	A combination of urban and rural living	5	Yes, older parental age improves children's mental health	1	I don't know	1		Often
22	1	Moravian-Silesian Region	4	0	0	Social media and technology, Study or work pressure, Family relationships, Loneliness and social isolation	Mixed (on the outskirts of a city or in small towns)	Easier access to medical and mental health care, Greater social and cultural opportunities (cinemas, concerts, cafés, etc.), Accessibility of employment and education, Better accessibility of public transport		A combination of urban and rural living	3	Yes, older parental age improves children's mental health	1	I don't know	2	Raising awareness of mental health through campaigns, Greater support in schools and universities, Creating a better preventive care system	Often
20	2	South Moravian Region	5	0	0	Social media and technology, Study or work pressure, Family relationships, Loneliness and social isolation	Urban environment	Easier access to medical and mental health care, Greater social and cultural opportunities (cinemas, concerts, cafés, etc.), Opportunity to establish more social contacts	Higher levels of stress and pace of life, Lack of nature and quiet places, Greater anonymity and feelings of loneliness	A combination of urban and rural living	4	Yes, older parental age improves children's mental health	1	Yes, a younger age (up to 21) may negatively affect her mental health	2	Raising awareness of mental health through campaigns, Creating a better preventive care system	Occasionally
19	1	South Moravian Region	4	0	2	Social media and technology, Study or work pressure, Family relationships, Loneliness and social isolation	Urban environment	Greater social and cultural opportunities (cinemas, concerts, cafés, etc.), Accessibility of employment and education, Greater anonymity and privacy	Higher levels of stress and pace of life, More noise and pollution, Greater competitive pressure in the job market	In a city	3	Yes, older parental age improves children's mental health	2	Yes, a younger age (up to 21) may negatively affect her mental health	3	Improved access to professional care (more therapists, shorter waiting times), Greater support in schools and universities	Occasionally
22	2	Moravian-Silesian Region	4	5	2	Social media and technology, Study or work pressure, Family relationships, Loneliness and social isolation	Urban environment	Greater social and cultural opportunities (cinemas, concerts, cafés, etc.), Accessibility of employment and education, Better accessibility of public transport, Opportunity to establish more social contacts	Higher levels of stress and pace of life, More noise and pollution, Lack of nature and quiet places	In the countryside	4	Yes, younger parental age improves children's mental health	1	I don't know	1	Improving access to specialist care (more therapists, shorter waiting times), improving the affordability of mental health care, raising awareness of mental health through campaigns, greater support in schools and universities, creating a better preventive care system.	Occasionally
23	1	South Moravian Region	4	0	0	Social media and technology, Study or work pressure, Family relationships, Loneliness and social isolation	Rural environment	Greater social and cultural opportunities (cinemas, concerts, cafés, etc.), Greater anonymity and privacy, Better access to public transport	More noise and pollution, Lack of nature and quiet places, Higher crime rate	In the countryside	5	No, parental age has no effect	2	No, maternal age has no effect on her mental health	2	Raising awareness of mental health through campaigns, Greater support in schools and universities	Never
21	1	Moravian-Silesian Region	2	3	0	Social media and technology, Study or work pressure, Family relationships, Loneliness and social isolation	Urban environment	Greater social and cultural opportunities (cinemas, concerts, cafés, etc.), Accessibility of employment and education, Better accessibility of public transport, Opportunity to establish more social contacts	Higher levels of stress and pace of life, More noise and pollution, Lack of nature and quiet places	A combination of urban and rural living	2	I don't know	1	Yes, an older age (over 21) may positively affect her mental health	1	Improved access to specialist care (more therapists, shorter waiting times), improved affordability of mental health care, greater support in schools and universities	Always
21	1	Moravian-Silesian Region	4	0	0	Social media and technology, Study or work pressure, Family relationships, Loneliness and social isolation	Urban environment	Greater social and cultural opportunities (cinemas, concerts, cafés, etc.), Opportunity to establish more social contacts	Higher levels of stress and pace of life, More noise and pollution, Lack of nature and quiet places, Higher crime rates	A combination of urban and rural living	3	No, parental age has no effect	2	No, maternal age has no effect on her mental health	2	Improving the affordability of mental health care, Greater support in schools and universities, Creating a better preventive care system	Often
21	1	Moravian-Silesian Region	4	0	2	Social media and technology, Study or work pressure, Family relationships, Loneliness and social isolation	Urban environment	Easier access to medical and mental health care, Greater social and cultural opportunities (cinemas, concerts, cafés, etc.), Accessibility of employment and education, Opportunity to establish more social contacts	Higher levels of stress and pace of life, More noise and pollution, Lack of nature and quiet places	A combination of urban and rural living	4	No, parental age has no effect	2	Yes, a younger age (up to 21) may negatively affect her mental health	1	Improving access to specialist care (more therapists, shorter waiting times), creating a better preventive care system	Often
20	2	Moravian-Silesian Region	2	3	1	Social media and technology, Study or work pressure, Family relationships, Loneliness and social isolation	Mixed (on the outskirts of a city or in small towns)	Greater social and cultural opportunities (cinemas, concerts, cafés, etc.), better access to public transport, opportunity to make more social contacts	Higher levels of stress and pace of life, greater competitive pressure in the labor market	A combination of urban and rural living	4	No, parental age has no effect	2	Yes, a younger age (up to 21) may negatively affect her mental health	2	Zlepšení dostupnosti odborné péče (více terapeutů, kratší čekací doby), Zvýšení povědomí o duševním zdraví prostřednictvím kampaní, Větší podpora na školách a univerzitách, Vytvoření lepšího systému preventivní péče	Often
19	2	Moravian-Silesian Region	4	0	0	Social media and technology, Study or work pressure, Family relationships, Loneliness and social isolation, I would say mainly the environment we find ourselves in, e.g. loneliness and social isolation. Even though I may [illegible]	Mixed (on the outskirts of a city or in small towns)	Greater social and cultural opportunities (cinemas, concerts, cafés, etc.), Accessibility of employment and education, Greater anonymity and privacy, Better accessibility of public transport, Opportunity to establish more social contacts	More noise and pollution, Lack of nature and quiet places, Greater competitive pressure in the job market	A combination of urban and rural living	5	I don't know	3	Yes, a younger age (up to 21) may negatively affect her mental health	4	For me, it's more of a big problem on social media, because we unconsciously compare ourselves to the masters in the field, and on the one hand, yes, it's great for people who are competitive, because it spurs them on to better results, but on the other hand, we look for partners based on who we like and we look for the elite in the field, and that's just difficult. Another problem is that we don't try to save relationships. When [illegible]	Always
25	1	Moravian-Silesian Region	5	4	2	Social media and technology, Study or work pressure, Family relationships, Loneliness and social isolation, Crime and feeling unsafe	Mixed (on the outskirts of a city or in small towns)	Easier access to medical and mental health care, Accessibility of employment and education, Greater anonymity and privacy		In the countryside	4	Yes, older parental age improves children's mental health	4	Yes, a younger age (up to 21) may negatively affect her mental health	1		Often
19	2	Moravian-Silesian Region	2	0	0	Social media and technology, Study or work pressure, Family relationships, Loneliness and social isolation, People worry unnecessarily about trivial matters, which only damages their mental health	Urban environment	Greater social and cultural opportunities (cinemas, concerts, cafés, etc.), Opportunity to establish more social contacts	Higher levels of stress and pace of life, More noise and pollution	I have no proference	5	Yes, younger parental age improves children's mental health	1	I don't know	2	Zlepšení finanční dostupnosti péče o duševní zdraví	Occasionally
22	2	Moravian-Silesian Region	3	0	0	Social media and technology, Study or work pressure, Family relationships, Health status	Rural environment	Greater social and cultural opportunities (cinemas, concerts, cafés, etc.), Accessibility of employment and education, Greater anonymity and privacy, Better accessibility of public transport	Lack of nature and quiet places	In the countryside	3	Yes, younger parental age improves children's mental health	2	Yes, a younger age (up to 21) may negatively affect her mental health	3	Greater support in schools and universities	Always
19	1	Moravian-Silesian Region	2	0	1	Social media and technology, Study or work pressure, Relationships	Urban environment	Greater social and cultural opportunities (cinemas, concerts, cafés, etc.), Accessibility of employment and education, Better accessibility of public transport	Higher levels of stress and pace of life, Higher crime rates, Greater competitive pressure in the labor market	A combination of urban and rural living	4	Yes, older parental age improves children's mental health	3	Yes, a younger age (up to 21) may negatively affect her mental health	2	Improved access to specialist care (more therapists, shorter waiting times), Improved affordability of mental health care	Often
21	2	Moravian-Silesian Region	2	0	2	Social media and technology, Study or work pressure, Health problems, Speaking in front of people	Mixed (on the outskirts of a city or in small towns)	Easier access to medical and mental health care, Greater social and cultural opportunities (cinemas, concerts, cafés, etc.), Better public transport accessibility	Higher levels of stress and pace of life, More noise and pollution, Lack of nature and quiet places, Greater anonymity and feelings of loneliness, Higher crime rates, Greater competitive pressure in the job market	In the countryside	5	No, parental age has no effect	2	No, maternal age has no effect on her mental health	1	Greater support in schools and universities, Creation of a better preventive care system	Often
20	2	Moravian-Silesian Region	2	0	0	Study or work pressure	Mixed (on the outskirts of a city or in small towns)	Better accessibility of public transport		In the countryside	4	I don't know	1	Yes, a younger age (up to 21) may negatively affect her mental health	3	Greater support in schools and universities	Always
20	2	Zlín Region	4	0	0	Study or work pressure	Mixed (on the outskirts of a city or in small towns)	Greater social and cultural opportunities (cinemas, concerts, cafés, etc.), better access to public transport, opportunity to make more social contacts	Higher levels of stress and pace of life, Lack of nature and quiet places, Greater competitive pressure in the job market	In the countryside	4	I don't know	1	Yes, a younger age (up to 21) may negatively affect her mental health	1	Raising awareness of mental health through campaigns, Creating a better preventive care system	Occasionally
25	1	Moravian-Silesian Region	3	0	1	Study or work pressure	Mixed (on the outskirts of a city or in small towns)	Easier access to medical and mental health care, Opportunity to establish more social contacts	Higher levels of stress and pace of life, Lack of nature and quiet places	In the countryside	4	I don't know	1	I don't know	1	Improving access to specialist care (more therapists, shorter waiting times), Raising awareness of mental health through campaigns, Creating a better preventive care system	Always
20	2	Moravian-Silesian Region	2	3	2	Study or work pressure	Rural environment	Greater social and cultural opportunities (cinemas, concerts, cafés, etc.), better access to public transport, opportunity to make more social contacts	Higher levels of stress and pace of life, More noise and pollution	In the countryside	5	I don't know	1	I don't know	4	Creating a better preventive care system	Never
20	1	South Moravian Region	5	0	2	Study or work pressure	Urban environment	Greater social and cultural opportunities (cinemas, concerts, cafés, etc.), better access to public transport, opportunity to make more social contacts	More noise and pollution	A combination of urban and rural living	4	I don't know	1	No, maternal age has no effect on her mental health	3	Improving access to specialist care (more therapists, shorter waiting times), improving the affordability of mental health care, raising awareness of mental health through campaigns, greater support in schools and universities, creating a better preventive care system.	Occasionally
19	1	Moravian-Silesian Region	2	0	1		Mixed (on the outskirts of a city or in small towns)	Greater social and cultural opportunities (cinemas, concerts, cafés, etc.), Accessibility of employment and education, Better accessibility of public transport	More noise and pollution, Lack of nature and quiet places, Higher crime rate	In a city	1	I don't know	4	Yes, a younger age (up to 21) may negatively affect her mental health	3	Improved access to specialist care (more therapists, shorter waiting times), Improved affordability of mental health care	Always
20	1	Moravian-Silesian Region	3	0	0	Study or work pressure, loneliness, and social isolation	Mixed (on the outskirts of a city or in small towns)	Greater social and cultural opportunities (cinemas, concerts, cafés, etc.), Accessibility of employment and education, Opportunity to establish more social contacts	Greater anonymity and feelings of loneliness, greater competitive pressure in the job market	A combination of urban and rural living	5	I don't know	2	I don't know	3	Creating a better preventive care system	Never
19	2	Zlín Region	1	0	0	Study or work pressure, loneliness, and social isolation	Urban environment	Greater social and cultural opportunities (cinemas, concerts, cafés, etc.), Accessibility of employment and education, Better accessibility of public transport	Higher levels of stress and pace of life, More noise and pollution, Lack of nature and quiet places, Greater anonymity and feelings of loneliness	A combination of urban and rural living	4	Yes, older parental age improves children's mental health	1	Yes, an older age (over 21) may positively affect her mental health	2	Raising awareness of mental health through campaigns, Greater support in schools and universities, Creating a better preventive care system	Always
23	2	Moravian-Silesian Region	5	0	0	Pressure from studies or work, loneliness and social isolation, crime and feelings of insecurity	Mixed (on the outskirts of a city or in small towns)	Easier access to medical and mental health care, Greater social and cultural opportunities (cinemas, concerts, cafés, etc.), Opportunity to establish more social contacts	Lack of nature and quiet places, Greater anonymity and feelings of loneliness, Greater competitive pressure in the job market	In the countryside	4	No, parental age has no effect	3	Yes, a younger age (up to 21) may negatively affect her mental health	3	Improved access to specialist care (more therapists, shorter waiting times), Improved affordability of mental health care	Never
19	1	Moravian-Silesian Region	2	0	1	Pressure from studies or work, loneliness and social isolation, crime and feelings of insecurity	Mixed (on the outskirts of a city or in small towns)	Easier access to medical and mental health care, Greater social and cultural opportunities (cinemas, concerts, cafés, etc.), Opportunity to establish more social contacts	Higher levels of stress and pace of life, Greater anonymity and feelings of loneliness, Greater competitive pressure in the job market	In the countryside	3	I don't know	4	Yes, a younger age (up to 21) may negatively affect her mental health	1	Greater support in schools and universities, Creation of a better preventive care system	Always
23	2	Moravian-Silesian Region	3	0	0	Pressure from studies or work, loneliness and social isolation, crime and feelings of insecurity	Urban environment	Greater social and cultural opportunities (cinemas, concerts, cafés, etc.), Accessibility of employment and education, Better accessibility of public transport		In a city	4	No, parental age has no effect	2	No, maternal age has no effect on her mental health	2	Raising awareness of mental health through campaigns	Never
25	2	Olomouc Region	2	3	1	Study or work pressure, loneliness and social isolation, insufficient access to healthcare	Rural environment	Easier access to medical and mental health care, Greater social and cultural opportunities (cinemas, concerts, cafés, etc.), Opportunity to establish more social contacts	Higher levels of stress and pace of life, Lack of nature and quiet places, Greater competitive pressure in the job market	In the countryside	3	Yes, older parental age improves children's mental health	1	Yes, a younger age (up to 21) may negatively affect her mental health	3	Improved access to professional care (more therapists, shorter waiting times), Greater support in schools and universities	Often
22	2	Moravian-Silesian Region	2	3	2	Study or work pressure, loneliness and social isolation, insufficient access to healthcare	Mixed (on the outskirts of a city or in small towns)	Easier access to medical and mental health care, Greater social and cultural opportunities (cinemas, concerts, cafés, etc.), Accessibility of employment and education	More noise and pollution, Lack of nature and quiet places, Higher crime rate	A combination of urban and rural living	5	No, parental age has no effect	1	No, maternal age has no effect on her mental health	1	Improving the affordability of mental health care, Creating a better preventive care system	Often
22	1	South Moravian Region	4	4	1	Study or work pressure, loneliness and social isolation, insufficient access to healthcare	Urban environment	Greater social and cultural opportunities (cinemas, concerts, cafés, etc.), Accessibility of employment and education, Better accessibility of public transport	Higher levels of stress and pace of life, More noise and pollution, Greater anonymity and feelings of loneliness	A combination of urban and rural living	5	I don't know	1	I don't know	5	Improved access to specialist care (more therapists, shorter waiting times), Improved affordability of mental health care	Occasionally
18	2	Moravian-Silesian Region	4	0	3	Study or work pressure, loneliness and social isolation, toxic relationships with people	Urban environment	Greater social and cultural opportunities (cinemas, concerts, cafés, etc.), Accessibility of employment and education, Opportunity to establish more social contacts	Higher levels of stress and pace of life, Higher crime rates, Greater competitive pressure in the labor market	I have no proference	5	I don't know	2	I don't know	2	Greater support in schools and universities, Creation of a better preventive care system	Often
20	2	Moravian-Silesian Region	2	0	1	Study or work pressure, Family relationships	Urban environment	Easier access to medical and mental health care, Greater social and cultural opportunities (cinemas, concerts, cafés, etc.), Accessibility of employment and education, Greater anonymity and privacy, Better accessibility of public transport	Higher levels of stress and pace of life, More noise and pollution, Lack of nature and quiet places	A combination of urban and rural living	4	Yes, older parental age improves children's mental health	2	Yes, a younger age (up to 21) may negatively affect her mental health	1	Improving access to specialist care (more therapists, shorter waiting times), improving the affordability of mental health care, raising awareness of mental health through campaigns, greater support in schools and universities	Always
21	1	Central Bohemian Region	4	0	1	Study or work pressure, Family relationships	Rural environment	Easier access to medical and mental health care, Greater social and cultural opportunities (cinemas, concerts, cafés, etc.)	Higher levels of stress and pace of life, More noise and pollution, Lack of nature and quiet places	In the countryside	4	Yes, younger parental age improves children's mental health	2	Yes, a younger age (up to 21) may negatively affect her mental health	2	Improving access to professional care (more therapists, shorter waiting times), Raising awareness of mental health through campaigns, Greater support in schools and universities	Always
20	1	South Moravian Region	3	4	1	Study or work pressure, family relationships, the cost of sessions with a psychologist—not everyone's parents will pay for it, and $30 per hour is really too much.	Rural environment		Higher levels of stress and pace of life, More noise and pollution, Lack of nature and quiet places, Greater competitive pressure in the job market	A combination of urban and rural living	5	Yes, younger parental age improves children's mental health	2	Yes, a younger age (up to 21) may negatively affect her mental health	2	Improving access to specialist care (more therapists, shorter waiting times), improving the affordability of mental health care, raising awareness of mental health through campaigns, greater support in schools and universities, creating a better preventive care system.	Always
19	2	Moravian-Silesian Region	4	5	3	Study or work pressure, Family relationships, Crime and feeling of danger	Mixed (on the outskirts of a city or in small towns)	Accessibility of employment and education, Greater anonymity and privacy, Better accessibility of public transport	Higher levels of stress and pace of life, More noise and pollution, Higher crime rates	In a city	5	Yes, younger parental age improves children's mental health	3	Yes, an older age (over 21) may positively affect her mental health	4	Improving access to professional care (more therapists, shorter waiting times), Raising awareness of mental health through campaigns	Occasionally
23	1	Moravian-Silesian Region	4	0	0	Study or work pressure, Family relationships, Inadequate access to healthcare	Mixed (on the outskirts of a city or in small towns)		Higher levels of stress and pace of life, More noise and pollution, Greater anonymity and feelings of loneliness	A combination of urban and rural living	5	No, parental age has no effect	2	No, maternal age has no effect on her mental health	2	Improved access to specialist care (more therapists, shorter waiting times), greater support in schools and universities, creation of a better preventive care system.	Always
22	2	Zlín Region	2	4	2	Study or work pressure, Family relationships, Inadequate access to healthcare	Rural environment	Easier access to medical and mental health care, Accessibility of employment and education, Opportunity to establish more social contacts	Higher levels of stress and pace of life, Lack of nature and quiet places, Greater competitive pressure in the job market	In the countryside	5	Yes, older parental age improves children's mental health	1	Yes, a younger age (up to 21) may negatively affect her mental health	1	Improving access to specialist care (more therapists, shorter waiting times), creating a better preventive care system	Often
20	1	Central Bohemian Region	2	0	1	Study or work pressure, Family relationships, Inadequate access to healthcare	Rural environment		Higher levels of stress and pace of life, Higher crime rates, Greater competitive pressure in the labor market	I have no proference	5	No, parental age has no effect	2	No, maternal age has no effect on her mental health	1	Improved access to specialist care (more therapists, shorter waiting times), Improved affordability of mental health care	Always
20	2	Moravian-Silesian Region	4	0	2	Study or work pressure, Family relationships, Loneliness and social isolation	Urban environment	Easier access to medical and mental health care, Greater social and cultural opportunities (cinemas, concerts, cafés, etc.), Opportunity to establish more social contacts	Higher levels of stress and pace of life, Higher crime rates, Greater competitive pressure in the labor market	A combination of urban and rural living	5	I don't know	3	I don't know	1	Improved access to specialist care (more therapists, shorter waiting times), improved affordability of mental health care, greater support in schools and universities	Often

Age	Gender	Region of Permanent Residence:	Q1. How would you rate your current mental state? (1 = Very poor, 5 = Very good)	Q2. If you sought professional help for your mental health (e.g., psychologist, psychiatrist), how would you rate the quality of these services? (1 = Very dissatisfied, 5 = Very satisfied)	Q3. Do you think mental health care is easily accessible in your city/region?	Q4: Which factors do you think affect the mental health of young people aged 18–25 the most? (select the 3 most important)	Q5. Do you live in an urban or rural environment?	Q6. What benefits of living in the city do you think have a positive impact on mental health? (select the 3 most important)	Q7. What disadvantages of living in the city do you think deteriorate mental health? (select the 3 most important)	Q8. If you could choose, where would you prefer to live in terms of mental health?	Q9. How important is support from family and friends to you in dealing with mental health problems? (1 = Not important, 5 = Very important)	Q10. Do you think the age at which people enter parenthood can affect the mental health of their children?	Q11. How much of an impact do you think crime in your region has on your mental health? (1 = No effect, 5 = Very strong effect)	Q12. Do you think the age of the first-time mother can affect her mental health?	Q13.How would you rate the level of general awareness and education about mental health in secondary schools and colleges? (1 = Very poor, 5 = Very good)	Q14. Which of the following do you think would be most helpful in improving mental health care for young people in your region? (select up to 2 options)	Q15. How often do you feel you have to look after your mental health on your own, without the help of professionals or family?
19	1	Moravian-Silesian Region	3	0	0	Study or work pressure, Family relationships, Loneliness and social isolation	Urban environment	Easier access to medical and mental health care, Accessibility of employment and education, Better accessibility of public transport	More noise and pollution, Higher crime rates, More competitive pressure in the job market	A combination of urban and rural living	2	I don't know	2	Yes, a younger age (up to 21) may negatively affect her mental health	1	Raising awareness of mental health through campaigns, Creating a better preventive care system	Often
19	1	Olomouc Region	3	0	0	Study or work pressure, Family relationships, Loneliness and social isolation	Rural environment	Greater social and cultural opportunities (cinemas, concerts, cafés, etc.), Accessibility of employment and education, Better accessibility of public transport	Higher levels of stress and pace of life, More noise and pollution, Lack of nature and quiet places	In the countryside	5	No, parental age has no effect	2	Yes, a younger age (up to 21) may negatively affect her mental health	3	Improving the affordability of mental health care, Creating a better preventive care system	Occasionally
19	1	Moravian-Silesian Region	2	3	1	Study or work pressure, Family relationships, Loneliness and social isolation	Urban environment	Accessibility of employment and education, Greater anonymity and privacy, Opportunity to establish more social contacts	Lack of nature and quiet places, Higher crime rate, Greater competitive pressure in the labor market	In the countryside	5	No, parental age has no effect	4	No, maternal age has no effect on her mental health	3	Raising awareness of mental health through campaigns, Creating a better preventive care system	Always
20	1	Moravian-Silesian Region	2	5	1	Study or work pressure, Family relationships, Loneliness and social isolation	Urban environment	Greater social and cultural opportunities (cinemas, concerts, cafés, etc.), Accessibility of employment and education, Opportunity to establish more social contacts	More noise and pollution, Lack of nature and quiet places, Greater anonymity and feelings of loneliness	In a city	5	Yes, older parental age improves children's mental health	2	No, maternal age has no effect on her mental health	1	Improving the affordability of mental health care, Raising awareness of mental health through campaigns	Occasionally
24	1	Moravian-Silesian Region	4	0	0	Study or work pressure, Family relationships, Loneliness and social isolation	Mixed (on the outskirts of a city or in small towns)	Greater social and cultural opportunities (cinemas, concerts, cafés, etc.), Opportunity to establish more social contacts	Lack of nature and quiet places	A combination of urban and rural living	3	No, parental age has no effect	2	No, maternal age has no effect on her mental health	1	Improving the affordability of mental health care, Raising awareness of mental health through campaigns, Greater support in schools and universities	Often
24	2	Moravian-Silesian Region	4	5	3	Study or work pressure, Family relationships, Loneliness and social isolation	Mixed (on the outskirts of a city or in small towns)	Easier access to medical and mental health care, Better public transport accessibility, Opportunity to establish more social contacts	Higher levels of stress and pace of life, Lack of nature and quiet places, Greater anonymity and feelings of loneliness	A combination of urban and rural living	5	Yes, older parental age improves children's mental health	1	Yes, a younger age (up to 21) may negatively affect her mental health	2	Improving the affordability of mental health care, Greater support in schools and universities	Often
22	1	Moravian-Silesian Region	3	0	0	Study or work pressure, Family relationships, Loneliness and social isolation	Urban environment	Easier access to medical and mental health care, Greater social and cultural opportunities (cinemas, concerts, cafés, etc.), Accessibility of employment and education, Opportunity to establish more social contacts	Higher levels of stress and pace of life, Lack of nature and quiet places, Greater anonymity and feelings of loneliness	A combination of urban and rural living	5	Yes, older parental age improves children's mental health	3	Yes, a younger age (up to 21) may negatively affect her mental health	2	Improving the affordability of mental health care, Raising awareness of mental health through campaigns, Greater support in schools and universities	Always
23	2	Olomouc Region	4	0	0	Study or work pressure, Family relationships, Loneliness and social isolation	Urban environment	Greater social and cultural opportunities (cinemas, concerts, cafés, etc.), Accessibility of employment and education, Greater anonymity and privacy	Higher levels of stress and pace of life, Greater anonymity and feelings of loneliness, Greater competitive pressure in the job market	In a city	5	No, parental age has no effect	1	Yes, a younger age (up to 21) may negatively affect her mental health	1	Raising awareness of mental health through campaigns, Greater support in schools and universities	Always
19	1	Moravian-Silesian Region	4	0	0	Study or work pressure, Family relationships, Loneliness and social isolation	Rural environment	Greater social and cultural opportunities (cinemas, concerts, cafés, etc.), Accessibility of employment and education, Opportunity to establish more social contacts		A combination of urban and rural living	5	Yes, older parental age improves children's mental health	2	I don't know	3	Improving access to specialist care (more therapists, shorter waiting times), Raising awareness of mental health through campaigns, Creating a better preventive care system	Occasionally
21	1	Moravian-Silesian Region	3	5	2	Study or work pressure, Family relationships, Loneliness and social isolation	Urban environment	Greater social and cultural opportunities (cinemas, concerts, cafés, etc.), Accessibility of employment and education, Greater anonymity and privacy	Higher levels of stress and pace of life, More noise and pollution, Lack of nature and quiet places	A combination of urban and rural living	4	No, parental age has no effect	1	I don't know	2	Improving the affordability of mental health care, Greater support in schools and universities	Always
23	1	Moravian-Silesian Region	3	0	0	Study or work pressure, Family relationships, Loneliness and social isolation	Urban environment	Greater social and cultural opportunities (cinemas, concerts, cafés, etc.), Accessibility of employment and education, Better accessibility of public transport	Higher levels of stress and pace of life, Higher crime rates, Greater competitive pressure in the labor market	In a city	5	No, parental age has no effect	5	I don't know	1	Greater support in schools and universities	Often
23	2	Zlín Region	3	0	0	Study or work pressure, Family relationships, Loneliness and social isolation	Rural environment	Greater social and cultural opportunities (cinemas, concerts, cafés, etc.), Accessibility of employment and education, Better accessibility of public transport	More noise and pollution, Lack of nature and quiet places, Greater anonymity and feelings of loneliness	In the countryside	5	Yes, older parental age improves children's mental health	2	Yes, a younger age (up to 21) may negatively affect her mental health	1	Raising awareness of mental health through campaigns, Greater support in schools and universities	Often
21	1	Pardubice Region	3	4	2	Study or work pressure, Family relationships, Loneliness and social isolation	Urban environment	Easier access to medical and mental health care, Greater social and cultural opportunities (cinemas, concerts, cafés, etc.), Greater anonymity and privacy	Higher levels of stress and pace of life, More noise and pollution, Lack of nature and quiet places	In the countryside	5	No, parental age has no effect	1	Yes, a younger age (up to 21) may negatively affect her mental health	1	Improving the affordability of mental health care, Greater support in schools and universities	Occasionally
22	1	Olomouc Region	3	0	0	Study or work pressure, Family relationships, Loneliness and social isolation	Rural environment	Easier access to medical and mental health care, Greater social and cultural opportunities (cinemas, concerts, cafés, etc.)	Higher levels of stress and pace of life, More noise and pollution, Lack of nature and quiet places, Greater competitive pressure in the job market	In the countryside	5	Yes, younger parental age improves children's mental health	2	Yes, an older age (over 21) may positively affect her mental health	3	Improved access to specialist care (more therapists, shorter waiting times), improved affordability of mental health care, greater support in schools and universities	Often
20	1	Zlín Region	4	0	0	Study or work pressure, Family relationships, Loneliness and social isolation	Rural environment		More noise and pollution, Lack of nature and quiet places, Higher crime rate	In the countryside	3	No, parental age has no effect	1	No, maternal age has no effect on her mental health	2	Greater support in schools and universities	Often
21	1	Moravian-Silesian Region	4	2	2	Study or work pressure, Family relationships, Loneliness and social isolation	Rural environment	Accessibility of employment and education, Better accessibility of public transport, Opportunity to establish more social contacts	Higher levels of stress and pace of life, More noise and pollution, Lack of nature and quiet places	In the countryside	5	Yes, older parental age improves children's mental health	2	Yes, a younger age (up to 21) may negatively affect her mental health	2	Improving the affordability of mental health care, Greater support in schools and universities, Creating a better preventive care system	Always
19	2	Moravian-Silesian Region	3	4	2	Study or work pressure, Family relationships, Loneliness and social isolation	Urban environment	Accessibility of employment and education, Better accessibility of public transport, Opportunity to establish more social contacts	Higher levels of stress and pace of life, Lack of nature and quiet places, Greater competitive pressure in the job market	In a city	4	I don't know	1	Yes, a younger age (up to 21) may negatively affect her mental health	2	Raising awareness of mental health through campaigns, Greater support in schools and universities	Often
21	1	Moravian-Silesian Region	4	0	0	Study or work pressure, Family relationships, Loneliness and social isolation	Urban environment	Greater social and cultural opportunities (cinemas, concerts, cafés, etc.), Greater anonymity and privacy, Better access to public transport	More noise and pollution, Lack of nature and quiet places, Greater competitive pressure in the job market	A combination of urban and rural living	4	No, parental age has no effect	3	Yes, a younger age (up to 21) may negatively affect her mental health	2	Creating a better preventive care system	Occasionally
21	1	Olomouc Region	4	0	0	Study or work pressure, Family relationships, Loneliness and social isolation	Rural environment	Easier access to medical and mental health care, Greater social and cultural opportunities (cinemas, concerts, cafés, etc.), Greater anonymity and privacy		In the countryside	5	I don't know	3	Yes, an older age (over 21) may positively affect her mental health	4	Improving access to specialist care (more therapists, shorter waiting times), creating a better preventive care system	Often
23	1	Moravian-Silesian Region	2	5	2	Study or work pressure, Family relationships, Loneliness and social isolation	Urban environment	Greater social and cultural opportunities (cinemas, concerts, cafés, etc.), Accessibility of employment and education, Better accessibility of public transport	More noise and pollution, Lack of nature and quiet places, Higher crime rate	In a city	5	I don't know	1	I don't know	3	Improved access to specialist care (more therapists, shorter waiting times), improved affordability of mental health care, greater support in schools and universities	Often
21	1	Moravian-Silesian Region	4	0	2	Study or work pressure, Family relationships, Loneliness and social isolation	Mixed (on the outskirts of a city or in small towns)	Easier access to medical and mental health care, Greater social and cultural opportunities (cinemas, concerts, cafés, etc.), Better public transport accessibility	Higher levels of stress and pace of life, Higher crime rates, Greater competitive pressure in the labor market	A combination of urban and rural living	5	No, parental age has no effect	3	Yes, a younger age (up to 21) may negatively affect her mental health	1	Improving access to specialist care (more therapists, shorter waiting times), creating a better preventive care system	Always
21	1	South Moravian Region	3	1	0	Study or work pressure, Family relationships, Loneliness and social isolation	Mixed (on the outskirts of a city or in small towns)	Greater social and cultural opportunities (cinemas, concerts, cafés, etc.), better access to public transport	Lack of nature and quiet places, Greater anonymity and feelings of loneliness	A combination of urban and rural living	3	No, parental age has no effect	3	No, maternal age has no effect on her mental health	1	Greater support in schools and universities, comfortable assistance for individuals	Often
21	2	Olomouc Region	4	0	1	Study or work pressure, Family relationships, Loneliness and social isolation	Rural environment	Greater social and cultural opportunities (cinemas, concerts, cafés, etc.), Accessibility of employment and education, Opportunity to establish more social contacts	More noise and pollution, Lack of nature and quiet places, Greater competitive pressure in the job market	In the countryside	5	No, parental age has no effect	2	Yes, a younger age (up to 21) may negatively affect her mental health	2	Greater support in schools and universities, Creation of a better preventive care system	Occasionally
20	1	Moravian-Silesian Region	4	5	2	Study or work pressure, Family relationships, Loneliness and social isolation	Mixed (on the outskirts of a city or in small towns)	Easier access to medical and mental health care, Greater social and cultural opportunities (cinemas, concerts, cafés, etc.), Accessibility of employment and education		A combination of urban and rural living	5	I don't know	2	Yes, a younger age (up to 21) may negatively affect her mental health	2	Improved access to specialist care (more therapists, shorter waiting times), Improved affordability of mental health care	Occasionally
21	2	South Moravian Region	4	0	0	Study or work pressure, Family relationships, Loneliness and social isolation	Mixed (on the outskirts of a city or in small towns)	Greater social and cultural opportunities (cinemas, concerts, cafés, etc.), Accessibility of employment and education, Better accessibility of public transport		A combination of urban and rural living	2	I don't know	1	I don't know	3	Improved access to professional care (more therapists, shorter waiting times), Greater support in schools and universities	Often
21	1	South Moravian Region	2	0	2	Study or work pressure, Family relationships, Loneliness and social isolation	Urban environment	Greater social and cultural opportunities (cinemas, concerts, cafés, etc.), Accessibility of employment and education, Better accessibility of public transport	More noise and pollution, Lack of nature and quiet places, Higher crime rate	A combination of urban and rural living	5	Yes, older parental age improves children's mental health	5	No, maternal age has no effect on her mental health	3	Greater support in schools and universities, Creation of a better preventive care system	Often
22	1	South Moravian Region	4	0	2	Study or work pressure, Family relationships, Loneliness and social isolation	Mixed (on the outskirts of a city or in small towns)	Greater social and cultural opportunities (cinemas, concerts, cafés, etc.), Accessibility of employment and education, Better accessibility of public transport	Higher levels of stress and pace of life, Lack of nature and quiet places, Greater competitive pressure in the job market	A combination of urban and rural living	5	I don't know	1	Yes, a younger age (up to 21) may negatively affect her mental health	2	Raising awareness of mental health through campaigns, Creating a better preventive care system	Often
21	2	South Moravian Region	3	0	2	Study or work pressure, Family relationships, Loneliness and social isolation	Mixed (on the outskirts of a city or in small towns)	Accessibility of employment and education, Better accessibility of public transport, Opportunity to establish more social contacts	More noise and pollution, Lack of nature and quiet places, Greater anonymity and feelings of loneliness	A combination of urban and rural living	4	Yes, older parental age improves children's mental health	1	Yes, a younger age (up to 21) may negatively affect her mental health	3	Improving access to professional care (more therapists, shorter waiting times), Raising awareness of mental health through campaigns	Often
23	1	Pilsen Region	4	4	2	Study or work pressure, Family relationships, Loneliness and social isolation	Mixed (on the outskirts of a city or in small towns)	Easier access to medical and mental health care, Greater social and cultural opportunities (cinemas, concerts, cafés, etc.), Opportunity to establish more social contacts	Higher levels of stress and pace of life, More noise and pollution, Lack of nature and quiet places, Greater anonymity and feelings of loneliness, Greater competitive pressure in the job market	In the countryside	5	No, parental age has no effect	2	Yes, a younger age (up to 21) may negatively affect her mental health	3	Improving access to specialist care (more therapists, shorter waiting times), improving the affordability of mental health care, raising awareness of mental health through campaigns, greater support in schools and universities, creating a better preventive care system.	Occasionally
22	1	South Moravian Region	4	0	0	Study or work pressure, Family relationships, Loneliness and social isolation	Rural environment	Greater social and cultural opportunities (cinemas, concerts, cafés, etc.), Accessibility of employment and education, Better accessibility of public transport	Higher levels of stress and pace of life, More noise and pollution, Lack of nature and quiet places	A combination of urban and rural living	5	No, parental age has no effect	2	Yes, a younger age (up to 21) may negatively affect her mental health	2	Improving the affordability of mental health care, Greater support in schools and universities	Always
19	1	Capital City of Prague	3	4	2	Study or work pressure, Family relationships, Loneliness and social isolation	Urban environment	Easier access to medical and mental health care, Accessibility of employment and education, Greater anonymity and privacy	Higher levels of stress and pace of life, More noise and pollution, Greater competitive pressure in the job market	A combination of urban and rural living	5	Yes, younger parental age improves children's mental health	2	Yes, a younger age (up to 21) may negatively affect her mental health	3	Improved access to specialist care (more therapists, shorter waiting times), Improved affordability of mental health care	Often
20	1	South Moravian Region	3	0	0	Study or work pressure, Family relationships, Loneliness and social isolation	Urban environment	Greater social and cultural opportunities (cinemas, concerts, cafés, etc.), Accessibility of employment and education, Opportunity to establish more social contacts	Higher levels of stress and pace of life, More noise and pollution, Greater anonymity and feelings of loneliness	A combination of urban and rural living	5	No, parental age has no effect	2	Yes, a younger age (up to 21) may negatively affect her mental health	3	Improved access to professional care (more therapists, shorter waiting times), Greater support in schools and universities	Occasionally
20	1	Vysočina Region	4	0	1	Study or work pressure, Family relationships, Loneliness and social isolation	Mixed (on the outskirts of a city or in small towns)	Greater social and cultural opportunities (cinemas, concerts, cafés, etc.), Accessibility of employment and education, Opportunity to establish more social contacts	Higher levels of stress and pace of life, More noise and pollution, Greater anonymity and feelings of loneliness	A combination of urban and rural living	5	Yes, older parental age improves children's mental health	1	Yes, a younger age (up to 21) may negatively affect her mental health	3	Improving access to specialist care (more therapists, shorter waiting times), improving the affordability of mental health care, creating a better preventive care system	Often
20	2	Olomouc Region	5	0	0	Study or work pressure, Family relationships, Loneliness and social isolation	Urban environment	Greater social and cultural opportunities (cinemas, concerts, cafés, etc.), Accessibility of employment and education, Opportunity to establish more social contacts	Higher levels of stress and pace of life, More noise and pollution, Greater anonymity and feelings of loneliness	A combination of urban and rural living	4	Yes, younger parental age improves children's mental health	2	Yes, a younger age (up to 21) may negatively affect her mental health	1	Raising awareness of mental health through campaigns, Greater support in schools and universities	Occasionally
20	2	South Moravian Region	3	4	1	Study or work pressure, Family relationships, Loneliness and social isolation	Rural environment	Easier access to medical and mental health care, Greater social and cultural opportunities (cinemas, concerts, cafés, etc.), Accessibility of employment and education	Higher levels of stress and pace of life, Lack of nature and quiet places, Greater anonymity and feelings of loneliness	A combination of urban and rural living	5	Yes, older parental age improves children's mental health	3	Yes, a younger age (up to 21) may negatively affect her mental health	3	Improved access to professional care (more therapists, shorter waiting times), Greater support in schools and universities	Occasionally
20	1	Moravian-Silesian Region	4	4	2	Study or work pressure, Family relationships, Loneliness and social isolation	Mixed (on the outskirts of a city or in small towns)	Greater social and cultural opportunities (cinemas, concerts, cafés, etc.), Accessibility of employment and education, Better accessibility of public transport	Higher levels of stress and pace of life, Lack of nature and quiet places, Greater anonymity and feelings of loneliness	A combination of urban and rural living	4	Yes, older parental age improves children's mental health	2	Yes, a younger age (up to 21) may negatively affect her mental health	2	Greater support in schools and universities, Creation of a better preventive care system	Often
20	1	South Moravian Region	3	4	3	Study or work pressure, Family relationships, Loneliness and social isolation	Urban environment	Greater social and cultural opportunities (cinemas, concerts, cafés, etc.), Accessibility of employment and education, Opportunity to establish more social contacts	Higher levels of stress and pace of life, Lack of nature and quiet places, Greater competitive pressure in the job market	A combination of urban and rural living	5	Yes, older parental age improves children's mental health	5	Yes, a younger age (up to 21) may negatively affect her mental health	3	Improved access to specialist care (more therapists, shorter waiting times), Improved affordability of mental health care	Often
20	2	South Moravian Region	3	0	0	Study or work pressure, Family relationships, Loneliness and social isolation	Urban environment	Greater social and cultural opportunities (cinemas, concerts, cafés, etc.), Accessibility of employment and education, Opportunity to establish more social contacts	Higher levels of stress and pace of life, Lack of nature and quiet places, Greater anonymity and feelings of loneliness	A combination of urban and rural living	4	Yes, younger parental age improves children's mental health	1	Yes, a younger age (up to 21) may negatively affect her mental health	3	Improved access to specialist care (more therapists, shorter waiting times), Improved affordability of mental health care	Often
21	2	Olomouc Region	5	0	0	Study or work pressure, Family relationships, Loneliness and social isolation	Urban environment	Greater social and cultural opportunities (cinemas, concerts, cafés, etc.), Accessibility of employment and education, Opportunity to establish more social contacts	Higher levels of stress and pace of life, Greater anonymity and feelings of loneliness, Greater competitive pressure in the job market	A combination of urban and rural living	4	Yes, older parental age improves children's mental health	1	Yes, a younger age (up to 21) may negatively affect her mental health	1	Raising awareness of mental health through campaigns, Greater support in schools and universities	Often
22	1	Moravian-Silesian Region	4	3	2	Study or work pressure, Family relationships, Loneliness and social isolation	Urban environment	Greater social and cultural opportunities (cinemas, concerts, cafés, etc.), Accessibility of employment and education, Better accessibility of public transport		A combination of urban and rural living	5	Yes, younger parental age improves children's mental health	2	Yes, a younger age (up to 21) may negatively affect her mental health	2	Improved access to specialist care (more therapists, shorter waiting times), improved affordability of mental health care, greater support in schools and universities	Often
20	1	Moravian-Silesian Region	2	4	1	Study or work pressure, Family relationships, Loneliness and social isolation	Mixed (on the outskirts of a city or in small towns)	Accessibility of employment and education, Better accessibility of public transport, Opportunity to establish more social contacts	More noise and pollution, Lack of nature and quiet places, Higher crime rate	In the countryside	5	I don't know	4	No, maternal age has no effect on her mental health	1	Improving access to specialist care (more therapists, shorter waiting times), creating a better preventive care system	Often
20	1	Moravian-Silesian Region	1	2	2	Study or work pressure, Family relationships, Loneliness and social isolation	Rural environment	Accessibility of employment and education, Greater anonymity and privacy, Better accessibility of public transport	Higher levels of stress and pace of life, More noise and pollution, Lack of nature and quiet places	In the countryside	4	Yes, younger parental age improves children's mental health	1	Yes, a younger age (up to 21) may negatively affect her mental health	1	Raising awareness of mental health through campaigns, Greater support in schools and universities	Often
22	2	Capital City of Prague	3	5	2	Study or work pressure, Family relationships, Loneliness and social isolation	Urban environment		Higher levels of stress and pace of life, More noise and pollution, Lack of nature and quiet places	A combination of urban and rural living	5	No, parental age has no effect	2	Yes, a younger age (up to 21) may negatively affect her mental health	2	Improved access to specialist care (more therapists, shorter waiting times), Improved affordability of mental health care	Always
23	2	Moravian-Silesian Region	4	0	0	Study or work pressure, Family relationships, Loneliness and social isolation	Urban environment	Greater social and cultural opportunities (cinemas, concerts, cafés, etc.), Accessibility of employment and education, Better accessibility of public transport, Opportunity to establish more social contacts	Higher levels of stress and pace of life, More noise and pollution, Greater competitive pressure in the job market	In a city	4	I don't know	3	Yes, an older age (over 21) may positively affect her mental health	2	Improving access to professional care (more therapists, shorter waiting times), Raising awareness of mental health through campaigns, Greater support in schools and universities, Creating a better preventive care system	Often
21	2	Olomouc Region	4	0	0	Pressure from studies or work, family relationships, loneliness and social isolation, being overly sensitive	Mixed (on the outskirts of a city or in small towns)	Greater social and cultural opportunities (cinemas, concerts, cafés, etc.), better access to public transport, opportunity to make more social contacts	Higher levels of stress and pace of life, Lack of nature and quiet places	A combination of urban and rural living	1	No, parental age has no effect	1	Yes, a younger age (up to 21) may negatively affect her mental health	3	Greater support in schools and universities, Creation of a better preventive care system	Always
21	2	Olomouc Region	3	0	0	Study or work pressure, Family relationships, Loneliness and social isolation, Crime and feeling unsafe	Rural environment	Greater social and cultural opportunities (cinemas, concerts, cafés, etc.), Greater anonymity and privacy, Opportunity to establish more social contacts	More noise and pollution, Lack of nature and quiet places, Higher crime rate	In the countryside	4	I don't know	4	I don't know	1	Creating a better preventive care system	Always
20	1	Zlín Region	5	0	0	Study or work pressure, Family relationships, Social life	Urban environment	Accessibility of employment and education, Greater anonymity and privacy, Opportunity to establish more social contacts	Higher levels of stress and pace of life, More noise and pollution, Greater competitive pressure in the job market	In the countryside	4	Yes, older parental age improves children's mental health	1	Yes, a younger age (up to 21) may negatively affect her mental health	3	Improving the affordability of mental health care, Creating a better preventive care system	Always
